# Importance of Precursor Adaptability in the Assembly of Molecular Organic Cages

**DOI:** 10.1021/acs.joc.2c02523

**Published:** 2023-02-02

**Authors:** Manuel Rondelli, Antonio H. Daranas, Tomás Martín

**Affiliations:** † Instituto de Productos Naturales y Agrobiología, Consejo Superior de Investigaciones Científicas (IPNA-CSIC), Avda. Astrofísico Francisco Sánchez, 3, 38206 La Laguna, Tenerife, Spain; ‡ Doctoral and Postgraduate School, University of La Laguna, Avda. Astrofísico Francisco Sánchez, 38203 La Laguna, Tenerife, Spain; § Instituto Universitario de Bio-Orgánica “Antonio González”, Universidad de La Laguna, Avda. Astrofísico Francisco Sánchez, 2, 38206 La Laguna, Tenerife, Spain

## Abstract

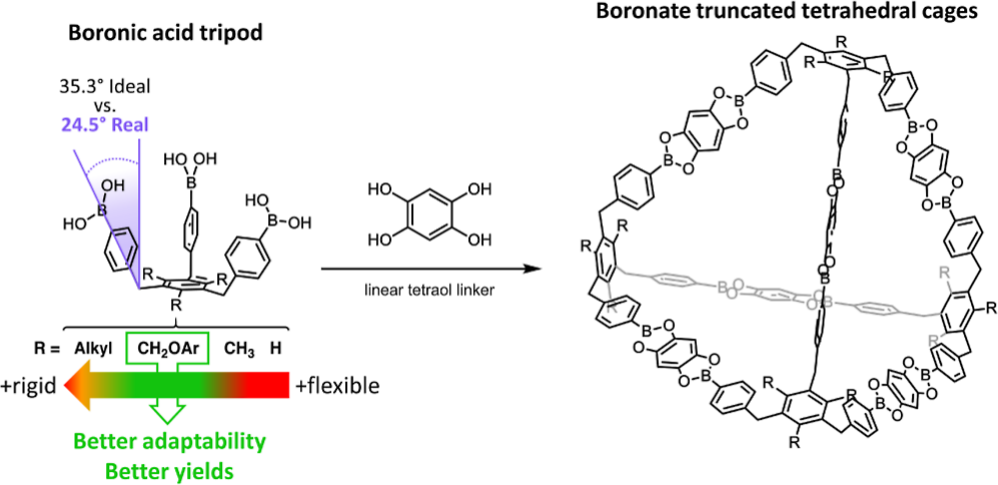

For molecular architectures based on dynamic covalent chemistry (DCvC), strict preorganization is a paradigmatic concept and the generally accepted strategy for their rational design. This results in the creation of highly rigid building blocks which are expected to fulfill the ideal geometry of the assembly, coming at a price that small geometric mismatches result in unpredicted and/or unproductive reaction outcomes. In this study, we show that feet of a tripodal platform have a great influence on the assembly of tetrahedral organic cages based on boronate ester formation. The aryl benzyl ether-functionalized building blocks perform significantly better than their alkyl-functionalized equivalents. Experimentally and using density functional theory geometry optimization of the cage structures, we prove that unexpectedly, this is not due to solubility but because of the enhanced capability of the aryl benzyl ether-functionalized building blocks to fit the ideal geometry of the assembly. This introduces the concept of building block adaptability to overcome geometrical mismatches in DCvC systems.

## Introduction

Nowadays, the rational design of complex molecular architectures such as molecular organic cages (MOCs) and covalent organic frameworks is based on a heuristic method known as the directional bonding approach.^1−3^ This means that for the prediction of the structural outcome, only the geometry, the directionality, and the topicity of the building blocks are taken into account.^4^ Although originally developed for non-covalent interactions in supramolecular chemistry, this approach has also been successfully employed in molecular systems based on dynamic covalent chemistry (DCvC).^5−15^ However, there are numerous surprising outcomes when applied to the synthesis of MOCs *via* DCvC. It has been shown that a wide range of factors such as the solvent,^16^ size of the building blocks,^17^ substituents of the building blocks,^18^ linker length,^19^ solubility,^20^ weak non-covalent interactions,^21^ pre-organization,^22^ and subtle geometrical features^4^ prevent the straight-forward prediction of the reaction outcome.

The main reason behind this unpredictability is the complex shape of the energetic landscape of reactions based on DCvC. The price for the stability of these systems is paid in kinetic bottlenecks and slow reaction rates^23,24^ that can lead to expected structures and/or intractable precipitates. To achieve reliable predictability and productive reactions, the energetic landscape of the reaction must neither be too flat (to insure that the thermodynamic minimum is reached within a reasonable time span, the so-called “Levinthal’s paradox”) nor be too steep (to avoid premature kinetic trapping).^4^ In other words, DCvC requires a smooth energetic landscape that in the first instance relies on the geometrical attributes of the building blocks. As a result, the geometry of the precursors in such systems is designed to be highly rigid and close to the ideal shape, to “accompany” the reaction pathway into the right direction and avoid kinetic trapping of undesired side-products.

However, two main limitations relate to this: (a) the ideal geometry cannot always be achieved due to the limitations of the organic chemical space, and (b) rigid structures are too sensitive toward geometric mismatches, preventing their adaptability to ideal geometries. In this context, the concept of adaptability can be associated with the ability of a molecule to undergo structural changes under unfavorable conditions by adjusting to them. Although ubiquitous in nature, this concept is scarcely applied in supramolecular chemistry. For multivalent host–guest complexes, it has been shown that the use of flexible spacers can significantly increase the binding by overcoming geometric mismatches through adaptability.^25^

**Figure 1:**
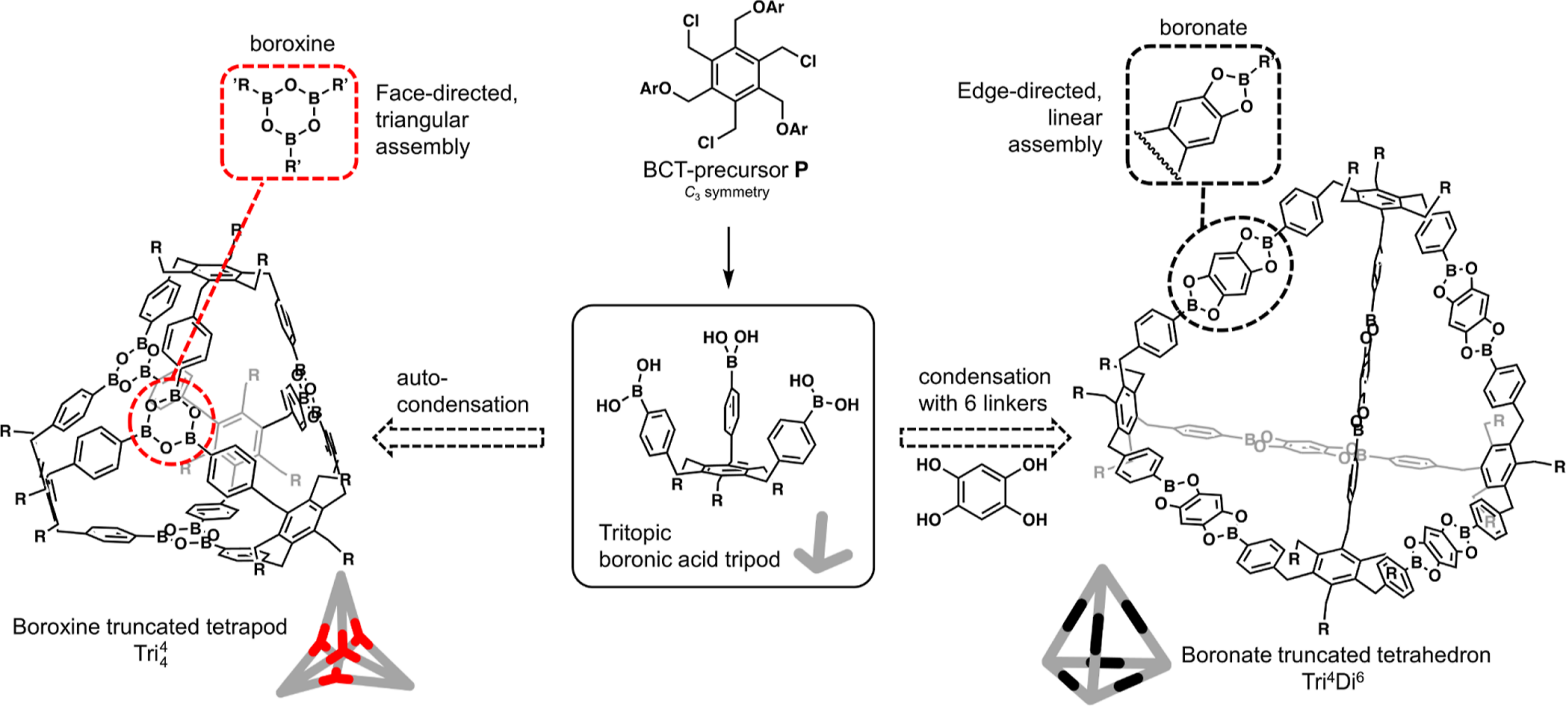
Our sterically geared, tritopic boronic acid tripod as a potential platform for the face-directed assembly of a boroxine-truncated tetrapod (left) and the edge-directed assembly of a boronate-truncated tetrahedral cage (right).

Here, we extend the concept of adaptability to DCvC by investigating the effects of precursor functionalization on the outcome of an MOC using boronic ester formation. Based on our previous studies toward the synthesis of functionalized benzocyclotrimers,^26−28^ we hypothesized that the sterically geared,^29,30^ 1,3,5/2,4,6-alternate substitution pattern of the *C*_3_-symmetric tripodal precursor **P** could be an ideal building block for the formation of a boroxine-truncated tetrapod with the topology Tri_4_^4^ by a face-directed assembly (Figure 1, left) and a boronate-truncated tetrahedron with the topology Tri^4^Di^6^ by an edge-directed assembly (Figure 1, right).^31^ Although the detailed geometrical analysis of the tripodal building block already anticipated potential problems in the formation of boroxine tetrapod Tri_4_^4^, the formation of boronate tetrahedron Tri^4^Di^6^ proved to be initially successful. However, when varying the substituents of the tripod, we observed unexpected differences in the yields for cage formation that did not match the “higher rigidity–higher yield” paradigm. Instead, we found that a certain degree of conformational flexibility of the building block is key to reaching maximum yields in the assembly of the anticipated cage.

## Results and Discussion

### Precursor Synthesis and Conformational Studies

**Scheme 1:**
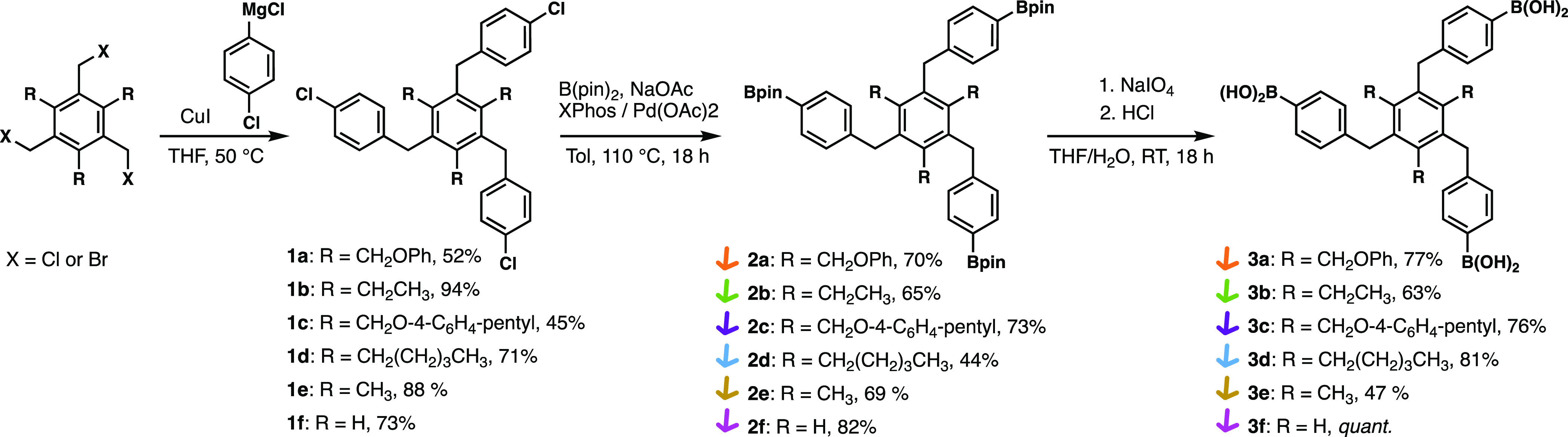
Synthesis of Triboronic Acid Precursors **3a–3f**

At first, we envisaged the synthesis and use of [2,4,6-tris(phenoxymethyl)]-triboronic acid **3a** as the potential building block (Scheme 1). Once we observed that cage formation was successful, we synthetized (2,4,6-triethylbenzene)-triboronic acid **3b**, with the hope to obtain crystals of a tetrahedral cage suitable for X-ray analysis. The unexpected differences in the yields of cages based on **3a** and **3b** lead us to perform a more thorough investigation including tripods **3c–3f**.

The synthetic sequence starts from 1,3,5-tris(bromomethyl)benzene or 1,3,5-tris(chloromethyl)benzene derivatives (Scheme 1). First, CuI-catalyzed coupling with 4-chlorophenylmagnesium bromide in tetrahydrofuran afforded the corresponding 1,3,5-tris(4-chlorobenzyl) derivative, which was subsequently subjected to the Miyaura borylation reaction with (Bpin)_2_. Finally, the pinacol esters were cleaved under oxidative conditions using NaIO_4_ to afford the corresponding boronic acid.

**Figure 2:**
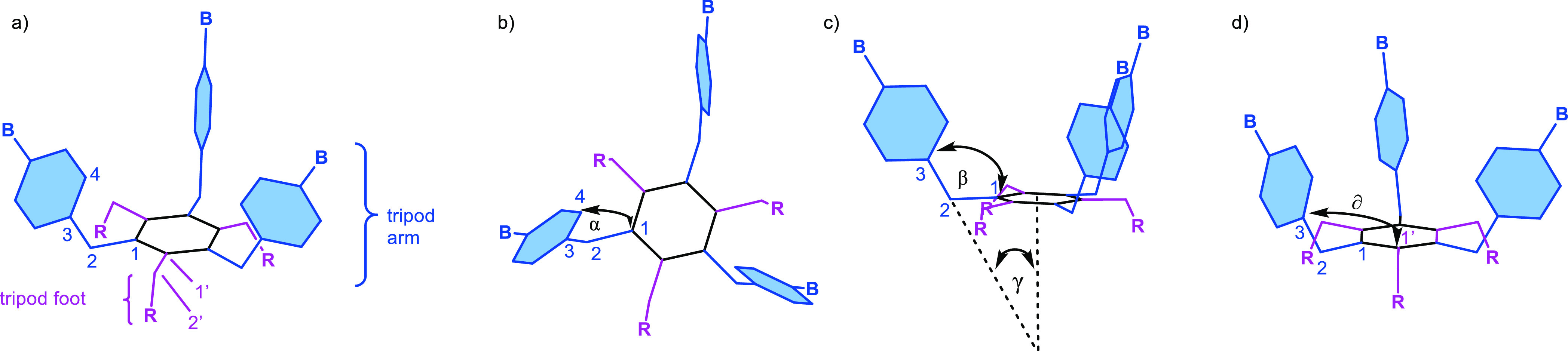
Numbering of atoms inside tripodal building blocks (a). Definition of dihedral angles α (b), angles β and γ (c), and dihedral angle δ (d). The angle γ is obtained by γ = β – 90°. The valency and the pinacol ester attached to the boron atoms have been omitted for clarity.

For a better understanding of the following conformational analysis, we introduce the terms “tripod arm” and “tripod foot” as depicted in Figure 2. The dihedral angle α is the absolute value of the angle defined by atoms 1-2-3-4, and δ is the dihedral angle enclosed by atoms 1′-1-2-3. The angle β is enclosed by atoms 1-2-3, and γ is is obtained by γ = β – 90°, being the angle between the tripod arm and the perpendicular to the central aromatic ring. Angles that are denoted with a bar (*e.g.*, α̅) refer to the value obtained from averaging the three values within one tripod.

**Figure 3:**
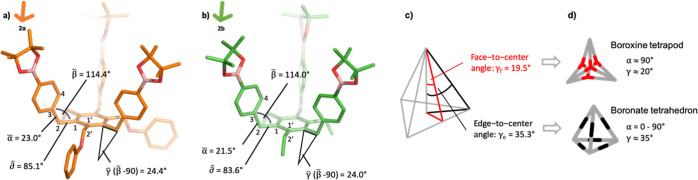
Geometrical analysis of DFT-optimized structures of **2a** (a) and **2b** (b); (c) ideal angles inside a regular tetrahedron; and (d) geometrical requirements for the formation of the boroxine tetrapod and boronate tetrahedron.

The conformational study started with crystals of **2a** and **2b** which were grown by slow cooling of a *n*-pentane/Et_2_O mixture and analyzed by X-ray analysis (see Section S9 in the Supporting Information). Both crystal structures showed deviation from the expected pure 1,3,5/2,4,6-alternated conformation pattern, which we attributed to packing effects in the crystal lattice and which has already been described in similar systems.^33,34^ Therefore, density functional theory (DFT) geometry optimization of **2a** and **2b** was performed (Figure 3. See Section S8.2 in the Supporting Information for details). To allow for comparison with the X-ray structures and to avoid artefacts resulting from intramolecular hydrogen bonding of the triboronic acids, the B-pin precursors were chosen. Despite the irregularity in the alternate conformation, the values for all angles (α, β, γ, and δ) obtained from DFT calculations are in good agreement with those obtained from X-ray analysis. Thus, the DFT structures were used for further analysis (Figure 3).

First, the angle γ for tripods **2a** (γ̅_2a_ = 24.4°) and **2b** (γ̅_2b_ = 24.0°) deviates only slightly from the face-to-center angle within a regular tetrahedron (γ_f_ = 19.5°), one requirement for the boroxine cage, face-directed assembly. Second, for both tripods, γ differs more significantly from the ideal edge-to-center angle γ_e_ = 35.3° of the boronate cage, edge-directed assembly (Figure 3). The previous data suggest that the boroxine assembly could be favored over the boronate assembly. However, for the boroxine assembly of tetrapod Tri_4_^4^, the dihedral angle α needs to be approximately 90° because the boroxine rings (having a planar trigonal arrangement) must be located on the faces of the tetrahedron. For both **2a** and **2b**, α lies in the range of 20° (probably as a consequence of the steric clash between the aromatic hydrogens of the 4-position and the neighboring benzylic hydrogens of the 2′-position). As a result, the boroxine Tri_4_^4^ assembly is likely to be inviable due to the large mismatch of α, despite the appropriated γ.

For the edge-directed assembly of tetrahedron Tri^4^Di^6^, the situation is the opposite: the assembly is independent of α due to its linear linker, but γ of the tripods differs approximately by 10° from the ideal angle. Last, the dihedral angles δ̅_2a_ = 85.1° and δ̅_2b_ = 83.6° indicate that the arms of the tripod are slightly tilted.

### Cage Synthesis

Based on the previous analyses, we first tried the construction of face-directed boroxine tetrapod Tri_4_^4^. Nevertheless, all our attempts to auto-condensate boronic acids **3a** and **3b** gave undefined ^1^H NMR spectra with broad or no signals. This agrees with the structural evaluation of **2a** and **2b** obtained from X-ray and DFT analysis, meaning that the dihedral angle α is too far from the 90° required value to enable face-directed assembly of a closed cage structure. In addition, we investigated boroxine tetrapod formation using building blocks **3c–3f**, but no significant cage formation was observed.

**Figure 4:**
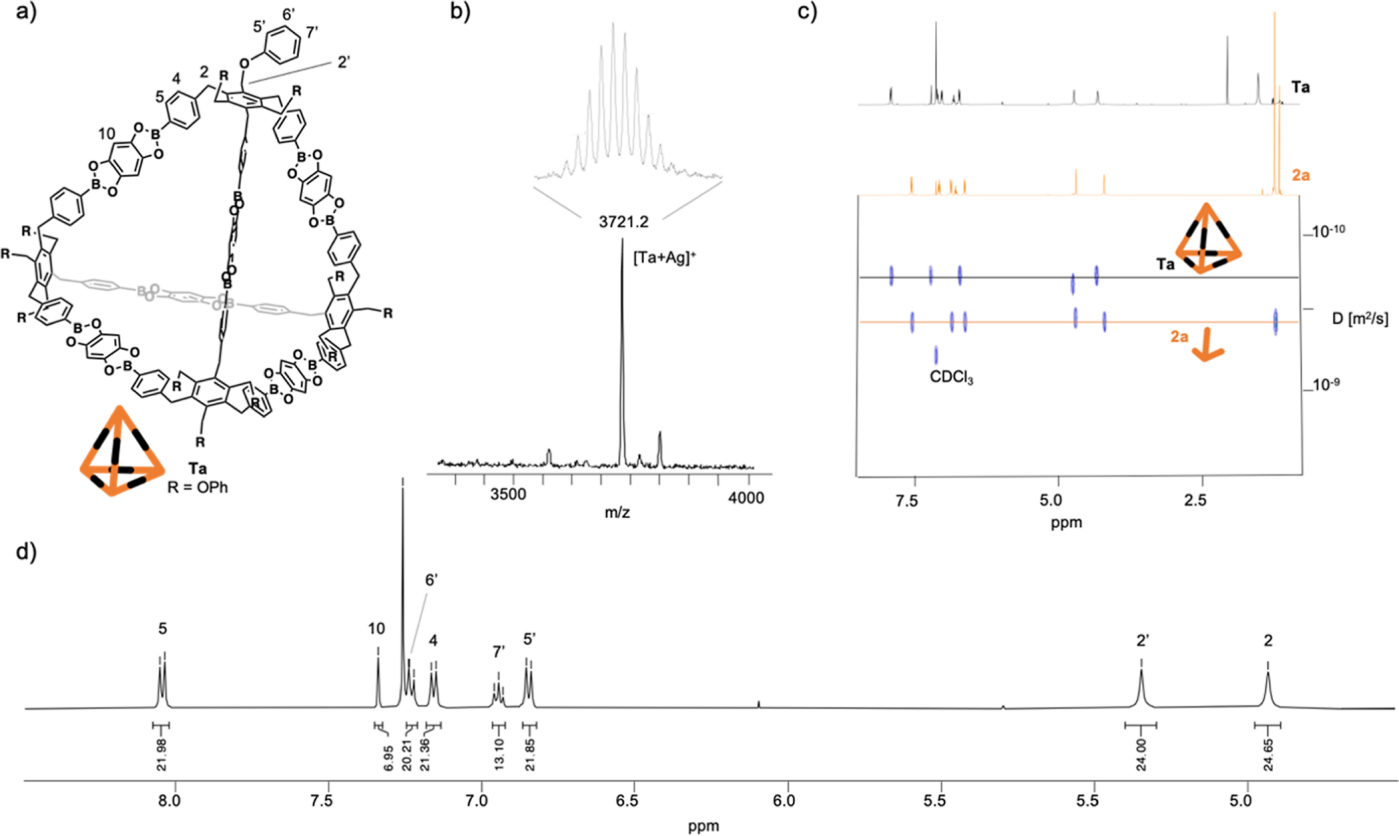
Characterization of boronate ester cage formation using **3a** and **THB**. (a) Structure of tetrahedral cage **Ta**. (b) Low- and high-resolution MALDI-TOF-MS of cage **Ta**. (c) ^1^H-DOSY-NMR (500 MHz, CDCl_3_) of a mixture of **Ta** and the corresponding Bpin precursor **2a**. Some signals corresponding to **Ta** and **2a** are omitted due to overlapping. (d) Excerpt of ^1^H NMR (400 MHz, CDCl_3_) of tetrahedral cage **Ta**.

From the previous results, we foresaw that changing the bond-forming geometry from a face-directed assembly (three components, boroxine) to an edge-directed assembly (two components, boronate ester) by using a linear linker would make the cage formation independent of the dihedral angle α. To probe this, we suspended **3a** and benzene-1,2,4,5-tetraol (**THB**) in a 4:6 ratio in CDCl_3_ using 2 equiv. of water for the boronic acid moiety^35^ and heated the mixture to 110 °C in a sealed pressure tube. The suspension gradually became a clear solution, and ^1^H NMR analysis after 72 h showed the expected spectrum of a Tri^4^Di^6^ cage **Ta** with *T*_*d*_ symmetry (Figure 4). Importantly, ^1^H-diffusion ordered spectroscopy (DOSY)-NMR showed the existence of a single species in this sample (see Figure S80 in the Supporting Information). Furthermore, an equimolar mixture of B-pin precursor **2a** (**2a** was chosen over **3a** due to its solubility in CDCl_3_) and cage **Ta** was prepared, and a ^1^H-DOSY NMR experiment was recorded. Data analysis confirmed the coexistence of two discrete species, with diffusion coefficients *D*_2a_ = 4.97 × 10^–10^ m^2^/s and *D*_Ta_ = 2.84 × 10^–10^ m^2^/s. Using a spherical-particle approximation and the Stokes–Einstein equation (see Section S7 in the Supporting Information), the volumes of the tripod **2a** and the cage **Ta** were calculated to be *V*_2a_ = 2420 Å^3^ and *V*_Ta_ = 12 932 Å^3^, respectively. These values are in excellent agreement with the volumes obtained from computational methods: *V*_2a_ = 2105 Å^3^ for **2a** and *V*_Ta_ = 11 445 Å^3^ for **Ta** (see Section S8.1 in the Supporting Information). The formation of tetrahedral cage **Ta** was further confirmed by matrix-assisted laser desorption/ionization-time of flight-mass spectrometry (MALDI-TOF-MS) (Figure 4). Unfortunately, all attempts to grow single crystals suitable for X-ray analysis failed.

Additionally, as described for other boronic ester cages,^15,36^ removal of the solvent under high vacuum resulted in the isolation of a white powder that proved to be impossible to re-dissolve, even after heating. However, as long as the product remains suspended in an organic solvent, it can easily be re-dissolved in CDCl_3_.

Reaction yields were calculated using an internal standard (1,1,2,2-tetrachloroethane) by quantitative ^1^H NMR.^37^ Every reaction was performed three times, and the average value showed that **Ta** was formed with a yield of 80 ± 2% (see Section S6 in the Supporting Information).

**Figure 5:**
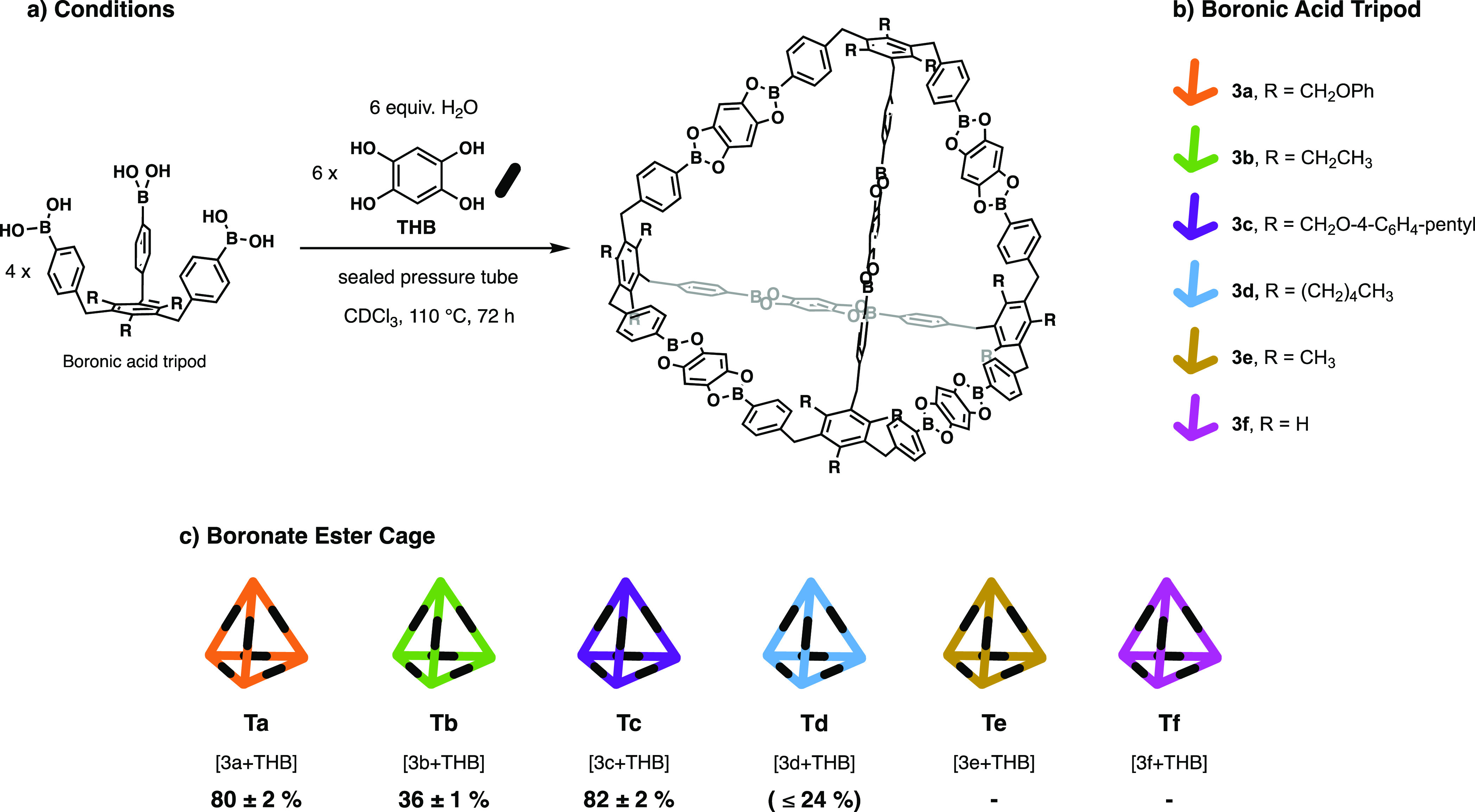
Synthesis of boronate ester tetrahedral cages. (a) General synthetic scheme for the formation of boronate ester cages. (b) Overview of boronic acid precursors used for the assembly of the cages. (c) Overview of reaction outcome of the cages. The yields were obtained from the average of three different reactions.

In an effort to obtain crystals suitable for X-ray analysis, we synthetized tripod **3b** and used it for cage formation with **THB**. Cage **Tb** was cleanly formed, but again, single crystals were not obtained. Interestingly, the average yield obtained for the assembly of **Tb** was 36 ± 1%, which was far below our expectations. During the reaction, a solid formed on the upper rim of the reaction mixture (see Figure S62 in the Supporting Information), which was analyzed by ^1^H NMR. While completely insoluble in CDCl_3_, the spectrum of the suspension in acetone-*d*_6_ shows only traces of **THB** (see Figure S63 in the Supporting Information). Since the boronic acid starting material is perfectly soluble in acetone-*d*_6_, we conclude that the precipitate consists of insoluble oligo/polymeric species (and traces of **THB**) that form during the course of the reaction and fall outside the equilibrium. Because the NMR spectrum of the reaction solution exclusively shows the presence of the product, the occurrence of the precipitate is in agreement with the low yields obtained for cage **Tb**.^38^ Considering this outcome, we decided to further investigate the role of the tripodal feet in the formation of our tetrahedral cage using tripods **3c**–**3f**. The results are summarized in Figure 5. Tripods **3e** and **3f** were synthetized to understand the effect of preorganization on cage assembly. The fact that cages **Te** and **Tf** did not form indicates that neither the hydrogen atom nor the methyl substituent in the 2,4,6-position induces sufficient steric gearing in the building block to direct the reaction toward cage assembly, which is similar to what was found in the formation of tetrahedral imine cages.^22^ Tripods **3c** and **3d** were synthetized to understand the influence of solubility on the assemblies while maintaining the geometrical attributes of **3a** and **3b**. Cage **Tc** formed with an average yield of 82 ± 2%. Therefore, the nearly identical yields in the formation of **Ta** and **Tc** indicate clearly that for this functionalization (aryl benzyl ether feet), the yield in cage formation is not determined by differences in solubility. In contrast, reaction with **3d** failed to produce a clean NMR spectrum, showing the presence of multiple and asymmetric species. Nevertheless, the MALDI-MS spectrum indicates that **Td** is formed, and integration of ^1^H NMR signals attributed to symmetrical cage **Td** shows that in all reactions, yields remained below or equal to 24%. Again, these results clearly demonstrate that the observed yield disparity in the formation of **Ta** and **Tb** is not a simple consequence of differences in solubility but that other factors must be at work. It should be noted that the tripod precursors **3a–3c** are practically insoluble in CDCl_3_ at room temperature; however, cages **Ta–Tc** are soluble even upon increasing the concentration at least 10-fold (from 0.005 to 0.05 M).

### Computational Studies and Yield Rationalization

The formation of boronic esters has been determined to be an entropically driven process, the driving force being the liberation of water molecules to the bulk solvent. Additionally, it is known that factors such as the functionality of the acid, the solvent, and the presence of coordinating donors can influence the condensation equilibrium.^39^ Furthermore, for the assembly of boronate cages, the conformation of the building blocks becomes a crucial factor.

Since for all cages investigated in this study, the amount of bonds (and hence the amount of water molecules) remains identical, the difference in the assembly efficiency among cages **Ta–f** must be a consequence of the tripodal feet of the building blocks.

Our results indicate that cage assembly is, in the first instance, governed by pre-organization of the building blocks: the tripods need sufficient steric gearing to efficiently assemble the cage. However, the question why cages **Ta** and **Tc** are formed with more than double the yield compared to **Tb** remained intriguing and unanswered. Solubility could be ruled out experimentally as the reason for this result because the pentyl-substituted tripod **3d** gave significantly worse results in the cage assembly than **3b**. To find an answer and keeping in mind that the tripod feet constitute the only difference between **3a** and **3b**, we performed an in-depth conformational analysis of tripods **2a** and **2b** (the Bpin precursors were used to compare with X-ray and NMR in CDCl_3_ and to avoid artifacts from intramolecular hydrogen bonding). For this, we conducted conformational searches (see Section S8.4 in the Supporting Information), molecular dynamics simulations (see Section S8.5 in the Supporting Information), and systematic energy scans associated with rotation of relevant dihedral angles (see Section S8.6 in the Supporting Information). Although all results pointed toward **2a** being conformationally slightly more loose than **2b**, none of these methods provided a conclusive explanation for our experimental observations.

**Figure 6:**
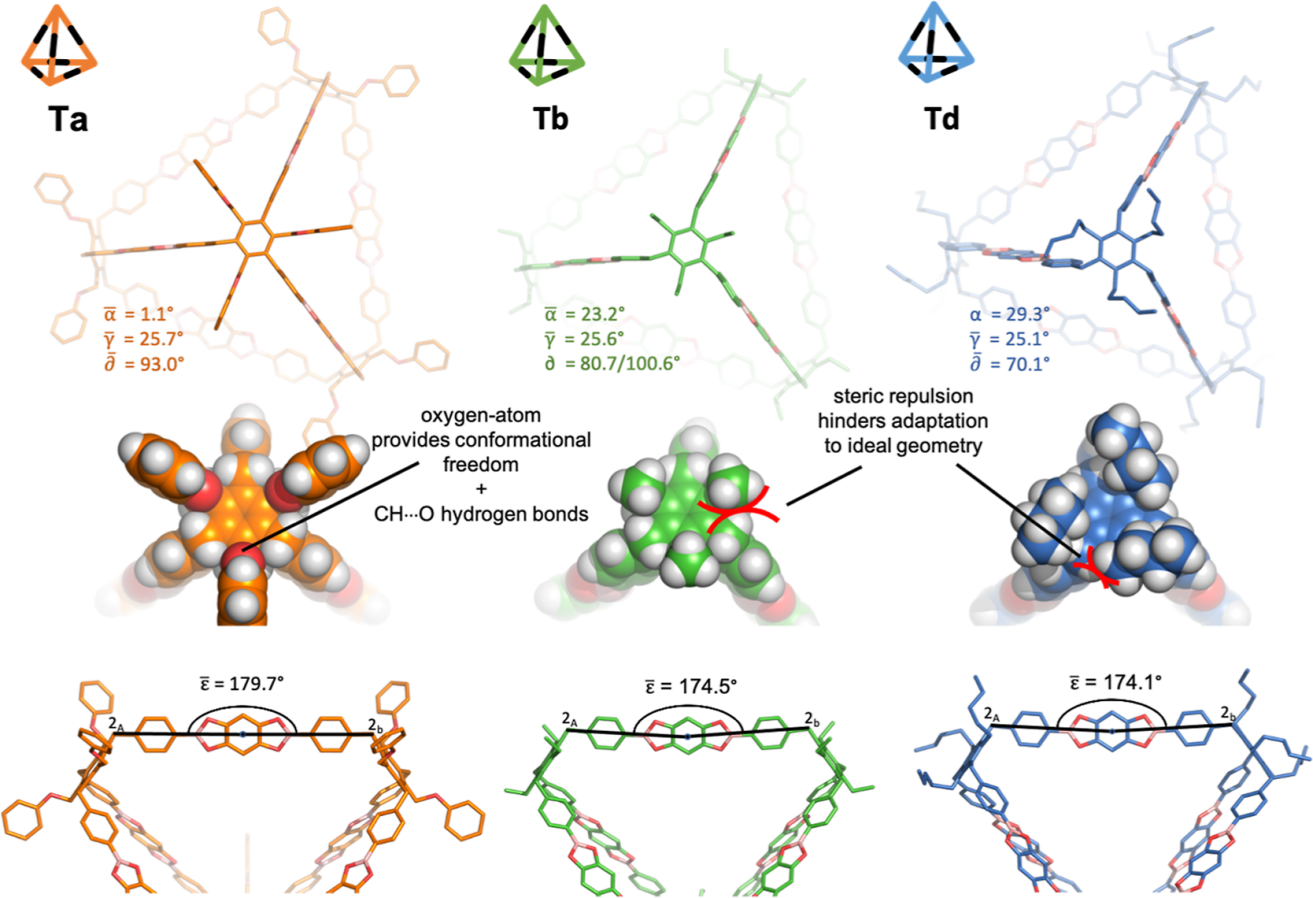
DFT-optimized structures of tetrahedral cages **Ta** (left), **Tb** (middle), and **Td** (right). The dihedral angle δ of **Tb** fluctuates between two vertices with approximately 80° and two vertices with approximately 100° and is thus given as the average of these two groups because the total average would result in approximately 90°, which would not reflect the reality.

Finally, we performed DFT geometry optimization of cages **Ta**, **Tb**, and **Td** at the B3LYP 6-31 level of theory (see Section S8.3 in the Supporting Information). **Tc** was not used due to redundancy with **Ta**. In the following, we refer to the tripod inside the tetrahedron as “vertex” (four per cage) and the connection between them as “edge” (six per cage). Additionally, ε refers to the strain angle of the tetrahedron edges, enclosed by (atom 2 of vertex A)—(centroid of **THB**)—(atom 2 of neighboring vertex B) (Figure 6). For each cage, all vertex angles α, β, γ, and δ and the strain angles ε were analyzed, and their average, standard deviation, range, and deviation from the values of the Bpin building blocks were calculated. A detailed overview can be found in Section S8.3 in the Supporting Information.

The calculations show that there is a significant structural difference between the cages (Figure 6). While **Ta** resembles nearly perfect *T*_*d*_ symmetry, the vertices of **Tb** and **Td** are twisted and tilted. These structural differences are clearly transferred to the values of the different angles. Therefore, the analysis of the strain angles ε, where ε̅_Ta_ = 179.7°, indicates that **Ta** is basically unstrained (|ε̅_Ta_ – 180°| = 0.3°), but **Tb** and **Td** show considerable strain with ε̅_Tb_ = 174.5° (|ε̅_Tb_ – 180°| = 5.3°) and ε̅_Td_ = 174.1° (|ε̅_Tc_ – 180°| = 5.9°). This trend is maintained also for angle γ, with γ̅_Ta_ = 25.7 ± 0° > γ̅_Tb_ = 25.6 ± 0.2° > γ̅_Tc_ = 25.1 ± 0°; however, it is less pronounced due to the intrinsic rigidity imposed by the sp^3^ hybridization of the C2 carbon atom. It is noteworthy that for tripod **3a**, γ goes from γ̅_2a_ = 24.4° to γ̅_Ta_ = 25.7° in cage **Ta**, while **3b** starts from γ̅_2b_ = 24.0° and changes to γ̅_Tb_ = 25.6° in cage **Tb**. We argue that the oxygen atoms of the phenyl benzyl ether feet enable **Ta** to adapt γ to the ideal tetrahedron angle (Figure 3) γ_e_ = 35.3° (within the sp^3^ limits) and at the same time adopt nearly perfect symmetrical structures with α̅_Ta_ = 1.1° and δ̅_Ta_ = 93.0°. This is because enough conformational freedom is provided to the benzylic hydrogen atoms of the 2-position of the tripod arm to undergo this adaptation. In addition, **Ta** is likely stabilized by a seam of bifurcated CH···O hydrogen bonds between the oxygen atoms of the phenyl benzyl ether and the benzylic hydrogens at the 2-position. The average values of the C–O distances and the CH–O dihedral angles of **Ta** are 3.35 Å and 127.9°, respectively, and thus lie within the range stipulated for this type of non-covalent interaction.^40^ On the other hand, in **Tb**, adaptation of γ_Tb_ comes at the cost of the tripodal arms being twisted out of symmetry, with α̅ turning from α̅_2b_ = 21.5° to α̅_Tb_ = 23.2° and δ tilting from δ̅_2b_ = 83.6° to δ̅_Tb_ = 80.7/100.6° (for δ̅_Tb_, we did not use the average because the tripod arms adapt angles of approximately 80° or approximately 100°; thus, the average would not reflect the conformational reality). This twist is due to steric clash of the benzylic hydrogens of the 2-position and the methyl hydrogens of 3′. The result is a cage structure that is less symmetrical and sterically less favorable, and it can be assumed that during the assembly process, the non-ideal geometry makes the last vertex unlikely to lock in to form the closed cage structure. For **Td**, this effect is clearly aggravated because the longer alkyl chain imposes more conformational restrictions compared to **Tb**.^41^ Thus, γ̅ of **Td** opens less, namely, from γ̅_2d_ = 24.0° to γ̅_Td_ = 25.1°, α̅ twists from α̅_2d_ = 23.0° to α̅_Td_ = 29.3°, and δ̅ tilts from δ̅_2d_ = 83.1° to δ̅_Td_ = 70.1° into an even more unfavorable geometry. All these results are in good agreement with the observed trend of the yields *Y*_Ta_ > *Y*_Tb_ > *Y*_Td_ (**Td** does not form cleanly).

**Figure 7:**
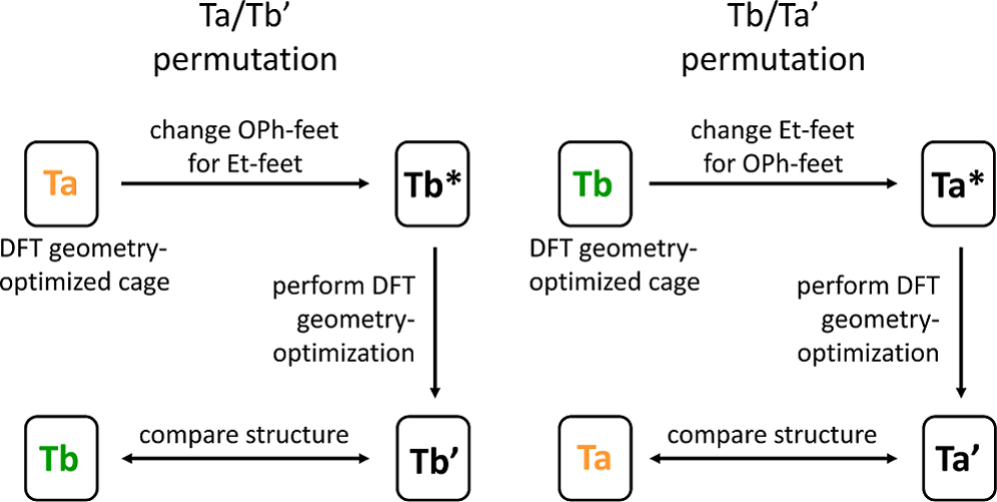
Schematic representation of the “structural permutations” performed for DFT geometry-optimized cages **Ta** and **Tb**. * denotes the permutated structures, and ′ denotes the permutated + optimized structures.

Finally, to confirm that the geometrical differences obtained for the optimized structures indeed arose from the differences in the tripodal feet/substituents, we performed structural “permutations” with cages **Ta** and **Tb** (Figure 7). For this, the geometry of cage **Ta** after DFT optimization was transformed into Tb* by changing the tripodal feet without performing any minimization/relaxation step. Then, the permutated structure Tb* was submitted to DFT geometry optimization to generate permutated + optimized **Tb′**. The same protocol was applied to **Tb** (to Ta* and then to **Ta′**). Analysis of the results showed nearly perfect agreement between **Tb**/**Tb′** pairs and **Ta**/**Ta′** pairs (see Tables S17/S18 and S19/S20 in the Supporting Information), demonstrating that there is an intrinsic difference in the cage structures depending on the tripod feet.

From a conceptual perspective, two aspects should be pointed out: (a) although the source of the described phenomena is small geometric differences of regions of the building blocks that do not actively participate in the reaction, the consequences determine the outcome of the assembly in a substantial way; (b) despite the fact that the rational lies in the geometrical attributes of the tripods, mere analysis of these building blocks could not provide a satisfying answer. Instead, the cage structures had to be analyzed to understand the full picture of the conformational behavior of the tripods and to assess their potential for adaptability, which proved to be crucial for the assembly process. The above-mentioned results demonstrate that conformational adaptability of the building blocks can be beneficial for DCvC systems and should be taken into account for the rational design of such systems, especially when building-block geometry is non-ideal for the assembly.

## Conclusions

In conclusion, we synthetized a series of boronic acid tripods as building blocks for the formation of MOCs based on boronic acid condensation. Through geometrical analysis of the building blocks, we explained why the formation of a boroxine tetrapod is unviable and solved this problem by using a linear linker to form a boronate tetrahedral cage. By varying the feet of the tripods, we observed that (a) a certain size of the tripodal substituents is necessary to induce steric gearing and enable cage formation and (b) that the tripods functionalized with aryl benzyl ethers gave significantly higher yields than those bearing alkyl chains. Through DFT, geometry optimization of the cage structures, and detailed angle analysis, we rationalized these results with the enhanced adaptability of the aryl benzyl ether-functionalized building blocks to fit the ideal geometry of the assembly. This highlights the profound effects that small geometrical features of the building blocks can have on DCvC systems and introduces the concept of building block adaptability to overcome geometrical mismatches in such systems. There are numerous examples where the feet of the molecular platforms are responsible for modulating the solubility of the building blocks and/or cage assembly.^20,42−44^ However, to the best of our knowledge, this is the first example where the feet modulate the adaptability of the building block and where this adaptability enhances yields in the cage assembly. This is opposed to the “higher rigidity–higher yield” principle that so far has been paradigmatic for DCvC systems.

The adaptability found in the aryl benzyl ether-functionalized tripod can be also extrapolated to modulate and improve the physico-chemical properties of receptors, sensors, capsules, and cages based on tripod scaffolds. Further studies to apply the detailed structural insights gained from this study on the assembly of more complex MOCs are currently underway in our laboratory.

## Data Availability

The data underlying this study are available in the published article and its Supporting Information.

## Supporting Information Available

Details for experimental procedures for the synthesis and characterization of the linear and tripod building blocks, synthesis and characterization of the tetrahedral cages, NMR spectra, DOSY experiments, molecular modeling, and crystallographic data (PDF)
