# Supplementary material for: Importance of Precursor Adaptability in the Assembly of Molecular Organic Cages
Source: J Org Chem. 2023 Feb 2;88(4):2113–21. doi: 10.1021/acs.joc.2c02523 (PMC9942191; doi:10.1021/acs.joc.2c02523)
Supplement: Supplementary file 1 — jo2c02523_si_001.pdf [file jo2c02523_si_001.pdf]

Supporting Information

Importance of precursor adaptability in the assembly of molecular organic cages

Manuel Rondelli,[a,b] Antonio H. Daranas [a,c,*], Tomás Martín [a,c,*]

[a] *Instituto de Productos Naturales y Agrobiología, Consejo Superior de Investigaciones Científicas (IPNA-CSIC), Avda. Astrofísico Francisco Sánchez, 3, 38206 La Laguna, Tenerife, Spain*
[b] *Doctoral and Postgraduate School, University of La Laguna, Avda. Astrofísico Francisco Sánchez, 38203 La Laguna, Tenerife, Spain*
[c] *Instituto Universitario de Bio-Orgánica "Antonio González", Universidad de La Laguna, Avda. Astrofísico Francisco Sánchez, 2, 38206 La Laguna, Tenerife, Spain*

*Email: adaranas@ipna.csic.es; tmartin@ipna.csic.es

Table of Contents

1. General Methods S3

2. Abbreviations S4

3. Synthesis of Building Blocks S5

4. NMR Spectra S18

5. Synthesis and Characterization of Tetrahedral Cages S41

6. Yield quantification S63

7. DOSY Experiments and Volume Approximation S67

8. Computational Calculations S76

8.1 Volume calculation of precursors and cages S76

8.2 DFT-optimized structures of tripodal building blocks S79

8.3 DFT-optimized structures of tetrahedral cages S81

8.4 Boltzmann distribution of tripods 2a and 2b S87

8.5 Molecular dynamics S89

8.6 Coordinate Scans S94

9. Crystallographic Data S97

10. Cartesian coordinates and Energies of molecular models based on force field calculations S101

11. Cartesian Coordinates and Energies of DFT optimized structures S123

12. References S149

1. General Methods

All reactions were performed under N_2 atmosphere in oven-dried glassware with magnetic stirring. For all reactions requiring heat, heated magnetic stirrers were used as a heat source, and Heat-On blocks were used to transfer heat to the round bottom flasks. Unless otherwise indicated, all reagents were purchased from commercial suppliers and used without any further purification. All solvents were purified by standard techniques or by a solvent purification system (PureSolv). Organic solutions were concentrated under reduced pressure on a rotary evaporator or an oil pump. Reactions were monitored through thin layer chromatography (TLC) on silica gel-precoated aluminum plates. Compounds were visualized by use of UV light, or by different stain solutions such as: vanillin with acetic and sulfuric acid in ethanol, potassium permanganate in sodium hydroxide aqueous solution, Ninhydrin solution or 2.5% phosphomolybdic acid in ethanol, and subsequent heating. Anhydrous sodium sulfate was used for drying solutions. Column chromatography were performed on silica gel, 60 Å and 0.2-0.5 mm. NMR spectra were recorded at 298 K on a Bruker NEO 500 spectrometer with a 5 mm broadband BBO cryo-probe equipped with a z-gradient unit with a maximum nominal gradient strength of 65.7 G cm^{-1}. ^{1}H and ^{13}C NMR chemical shifts were referenced to the $CHCl_3/CDCl_3$ solvent peak (δ_H 7.26 ppm and δ_C 77.0 ppm). The multiplicity of signals is abbreviated as follows: singlet (s), doublet (d), triplet (t), quartet (q), quintet (qui) and multiplet (m). Structural assignments were made with additional information from gCOSY, gHSQC, and gHMBC experiments. Quantitative ^{1}H-NMR measurements were done using calibrated 90 degrees pulses and relaxation delays of 30 s using 16.7 mM of 1,1,2,2-tetrachloroethane (TCE) as internal standard (for details see Chapter 6). DOSY experiments were performed at 298K using the double pulse gradient stimulated echo pulse sequence as implemented in the Bruker library (diffDste) using variable gradient pulse amplitudes. Gradients were varied using a linear scheme that started from 3.3 to 65.7 G/cm in 16 steps. Diffusion delays of 1 ms (little delta), 50 ms (big delta) and relaxation delays of 3 s were used. All DOSY spectra were processed using the diffusion analysis of dynamics center module available in Bruker Topspin 4.1 software. Low- and high-resolution mass spectra were recorded with TOF analyzer mass spectrometers (Waters LCT premier XE) by using electrospray ionization (ESI). Bruker Autoflex or Waters SYNAPT XS ion mobility Q-TOF mass spectrometer were used to acquired MALDI spectra. Infrared (FT-IR) spectra are reported in wavenumbers (cm^{-1}). Anhydrous $CDCl_3$ was prepared by adding dry 4 Å molecular sieves to a freshly opened bottle of $CDCl_3$ from VWR chemicals and leaving it at least for 48 hours before use.

2. Abbreviations

MALDI: matrix assisted laser desorption ionization
ESI: electro-spray ionization
ApCi: atmospheric pressure chemical ionization
TOF: time of flight
MS: mass spectrometry
TCE: 1,1,2,2,-tetrachloroethane
NMR: nuclear magnetic resonance
$CDCl_3$: deuterated chloroform
DCTB: *trans*-2-[3-(4-*tert*-Butylphenyl)-2-methyl-2-propenylidene]malononitrile
TFA: trifluoroacetate
HR: high-resolution
DOSY: diffusion-ordered spectroscopy
DFT: density functional theory
HF: Hartree-Fock
MM: molecular modeling
FF: force field

3. Synthesis of Building Blocks

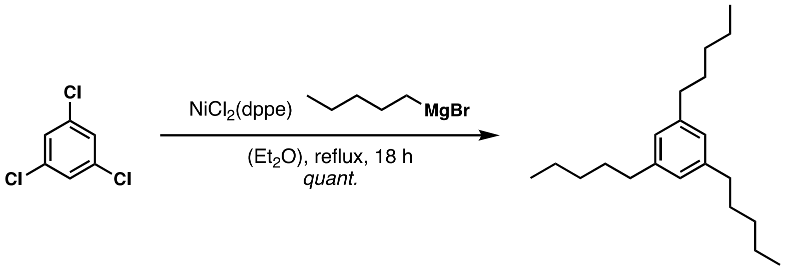

1,3,5-tripentylbenzene. According to the literature procedure,[1] a solution of pentylmagnesium bromide prepared from magnesium turnings (883 mg, 36.6 mmol) and pentyl bromide (3.0 mL, 24.3 mmol) in anhydrous diethyl ether (24.0 mL) was added at dropwise to a solution of 1,3,5-trichlorobenzene (708 mg, 3.9 mmol) and $NiCl_2$(dppe) (20.6 mg, 39.0 µmol) in anhydrous diethyl ether (8.0 mL). The resulting mixture was heated to reflux overnight. Upon completion, the reaction was quenched with aqueous 1N HCl (10 mL), diluted with water and the organic phase was extracted with CH_2Cl_2. The combined organic phases were dried over $NaSO_4$, filtered and concentrated *in vacuo*. The resulting oil was purified by column chromatography on silica gel (Hexane = 100%), yielding the product 1,3,5-tripentylbenzene as a colorless oil (1.12 g) in >99 % yield.
^{1}H NMR (400 MHz, $CDCl_3$) δ 6.81 (s, 3H), 2.57 – 2.50 (m, 6H), 1.60 (dt, *J* = 7.4, 15.3 Hz, 6H), 1.34 (dt, *J* = 2.6, 7.5 Hz, 6H), 0.94 – 0.83 (m, 12H).
^{13}C NMR{^{1}H} (126 MHz, $CDCl_3$) *δ* 142.7, 126.0, 36.1, 31.8, 31.5, 22.7, 14.2.
HRMS (ApCi): *m/z* calcd. for $C_{21}H_{37}$ $[M+H]^+$ 289.2895, found 289.2896.

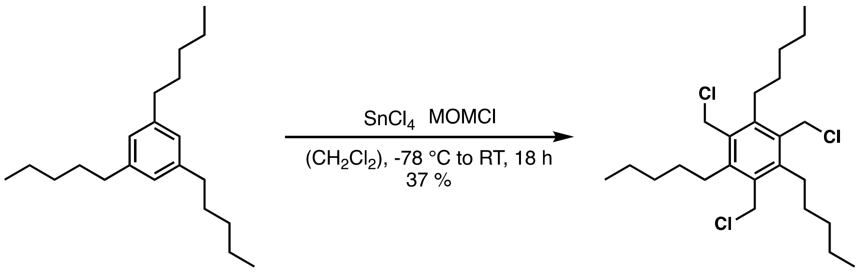

1,3,5-tris(chloromethyl)-2,4,6-tripentylbenzene. Following the literature procedure,[1] 1,3,5-tripentylbenzene (1.5 g, 5.20 mmol, 1.0 equiv.) was dissolved in 10 mL anhydrous CH_2Cl_2 (0.5 M) and MOMCl (5.92 mL, 78.0 mmol, 15.0 equiv.) was added. The resulting mixture was cooled to -78 °C and a 1.0 M solution of $SnCl_4$ in CH_2Cl_2 (62.4 mL, 62.5 mmol, 12.0 equiv.) was added dropwise. The reaction mixture was stirred 20 minutes at -78 °C and subsequently allowed to reach room temperature and stirred overnight. Upon completion, the reaction was carefully quenched with water and extracted with CH_2Cl_2. Purification by column chromatography (CH_2Cl_2/Hexane = 5/95) gave the product as a white, wax-like solid (830 mg, 37 %).
^{1}H NMR (400 MHz, $CDCl_3$) δ 4.65 (s, 1H), 2.90 – 2.72 (m, 1H), 1.67 – 1.56 (m, 1H), 1.53 – 1.46 (m, 1H), 1.45 – 1.37 (m, 1H), 0.95 (t, *J* = 7.2 Hz, 2H).
^{13}C NMR{^{1}H} (101 MHz, $CDCl_3$) δ 144.0, 132.8, 41.0, 32.7, 31.9, 29.9, 22.6, 14.2.
HRMS (ESI) *m/z* calcd. for $C_{24}H_{39}{}^{35}Cl_2{}^{37}ClAg$ $[M+Ag]^+$ 541.1139, found 541.1159.

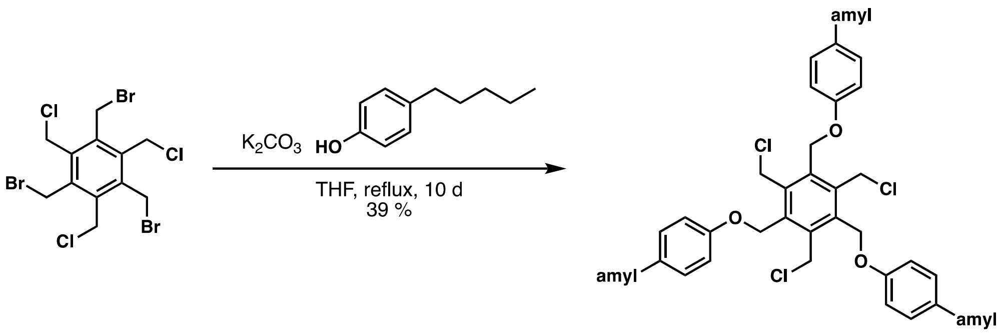

4,4',4''-(((2,4,6-Tris(chloromethyl)benzene-1,3,5-triyl)tris(methylene))tris(oxy))tris-(pentylbenzene). 1,3,5-Tris(bromomethyl)-2,4,6-tris(chloromethyl) benzene (3.0 g, 5.97 mmol, 1.0 equiv.) along with 4-pentylphenol (3.24 g, 3.37 mL, 19.71 mmol, 3.3 equiv.) were dissolved in dry THF (200 mL, 0.03 M) at 70 °C and anhydrous potassium carbonate (4.95 g, 35.8 mmol) was added. The reaction mixture was stirred at that temperature for 10 days. The reaction mixture was poured into water (400 mL) and extracted with dichloromethane. The combined organic solutions were dried over Na_2SO_4, and the resulting solution was concentrated and purified by column chromatography on silica gel (CH_2Cl_2/Hexane = 80/20 to 40/60), yielding the 4,4',4''-(((2,4,6-tris(chloromethyl)benzene-1,3,5-triyl)tris(methylene))tris(oxy))tris-(pentylbenzene) (1.76 g, 39 % yield) as a white solid.

^{1}H-NMR (400 MHz, $CDCl_3$) δ 7.19 (d, *J* = 8.3 Hz, 6H), 7.07 – 6.94 (m, 6H), 5.26 (s, 6H), 4.77 (s, 6H), 2.60 (t, *J* = 7.8 Hz, 6H), 1.63 (qui, *J* = 7.5 Hz, 6H), 1.41 – 1.31 (m, 12H), 0.92 (t, *J* = 7.0 Hz, 9H).

^{13}C-NMR{^{1}H} (101 MHz, $CDCl_3$) δ 156.6, 139.8, 136.8, 136.5, 129.7, 114.5, 63.0, 39.2, 35.2, 31.6, 31.6, 22.7, 14.2.

HRMS (ESI) *m/z* calcd. for $C_{45}H_{57}{}^{35}Cl_2{}^{37}ClO_3Na$ $[M+Na]^+$ 775.3241, found 775.3250.

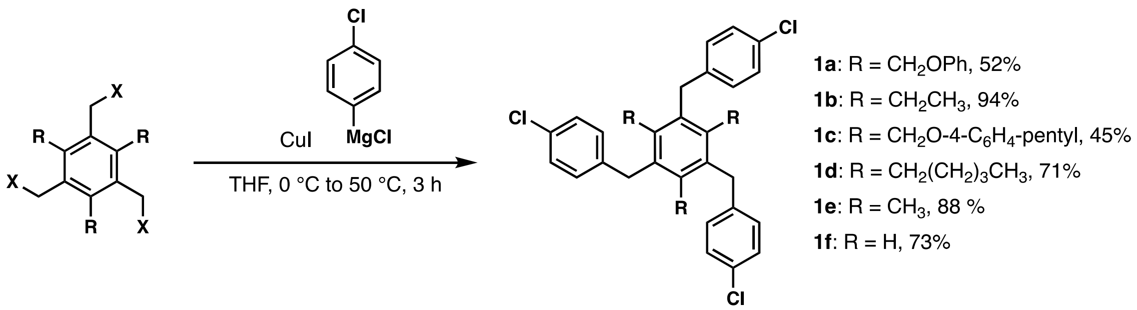

General Procedure B for the copper-catalyzed coupling of tripodal benzyl halides with 4-chlorophenylmagnesium bromide. According to the literature procedure[2] a solution of 4-chlorophenylmagnesium bromide in THF (1.0 M, 6.0 equiv.) was added at 0°C to a solution of 1,3,5-tris(halomethyl)benzene derivative (**1**) (1.0 equiv.) in THF (0.1 M) containing CuI (0.5 equiv.) under nitrogen over 30 min. The mixture was warmed up to room temperature and then stirred at 50°C for 1-3 h. The reaction was quenched with aqueous NH_4Cl and extracted three times with CH_2Cl_2. The combined organic layers were dried over anhydrous Na_2SO_4, filtered and concentrated *in vacuo*. The crude product was purified by flash chromatography on silica gel to yield the tris(chlorobenzene) as a white solid.

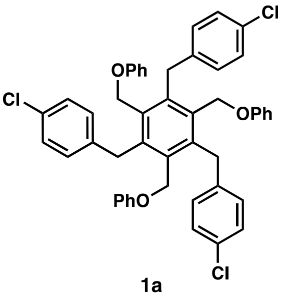

1a

4,4',4''-((2,4,6-Tris(phenoxymethyl)benzene-1,3,5-triyl)tris(methylene))tris (chlorobenzene) (1a). General procedure B was applied to (((2,4,6-tris(chloromethyl)benzene-1,3,5-triyl)tris(methylene))tris(oxy))tribenzene (536 mg, 0.99 mmol, 1.0 equiv.) in 10 mL THF with 4-chlorophenylmagnesium bromide in THF (1.0 M, 5.93 mL, 6.0 equiv.) and CuI (94.2 mg, 0.49 mmol, 0.5 equiv.) to obtain **1a** (428 mg, 52 % yield) as a white solid after purification by flash chromatography on silica gel (hexane/EtOAc = 9:1).

^{1}H-NMR (500 MHz, $CDCl_3$) δ 7.27 – 7.19 (m, 12H), 6.98 – 6.89 (m, 9H), 6.76 – 6.68 (m, 6H), 4.85 (s, 6H), 4.28 (s, 6H).

^{13}C-NMR{^{1}H} (126 MHz, $CDCl_3$) *δ* 158.6, 141.9, 138.8, 134.7, 132.1, 129.6, 129.5, 128.8, 121.3, 114.5, 64.6, 34.7.

HRMS (ESI) *m/z* calcd. for $C_{48}H_{39}{}^{35}Cl_2{}^{37}ClO_3Na$ $[M+Na]^+$ 793.1833, found 793.1817.

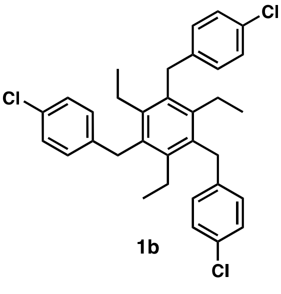

1b

4,4',4''-((2,4,6-Triethylbenzene-1,3,5-triyl)tris(methylene))tris(chlorobenzene) (1b). General procedure B was applied to 1,3,5-tris(bromomethyl)-2,4,6-triethylbenzene (2.00 g, 4.53 mmol, 1.0 equiv.) in 45 mL THF with 4-chlorophenylmagnesium bromide in THF (1.0 M, 27.2 mL, 6.0 equiv.) and CuI (431 mg, 2.27 mmol, 0.5 equiv.) to obtain **1b** (2.29 g, 94 % yield) as a white solid after purification by flash chromatography on silica gel (hexane/EtOAc = 98:2 to 95:5).
^{1}H-NMR (500 MHz, $CDCl_3$) δ 7.21 (d, J = 8.4 Hz, 6H), 6.90 (d, J = 8.2 Hz, 6H), 4.08 (s, 6H), 2.39 (q, J = 7.5 Hz, 6H), 1.04 (t, J = 7.4 Hz, 9H).
^{13}C-NMR{^{1}H} (126 MHz, $CDCl_3$) δ 141.5, 139.9, 133.8, 131.7, 129.1, 128.6, 34.1, 23.8, 15.3.
HRMS (ESI) *m/z* calcd. for $C_{33}H_{33}{}^{35}Cl_3$+^{109}Ag [M+Ag]$^+$ 643.0669, found 643.0684.

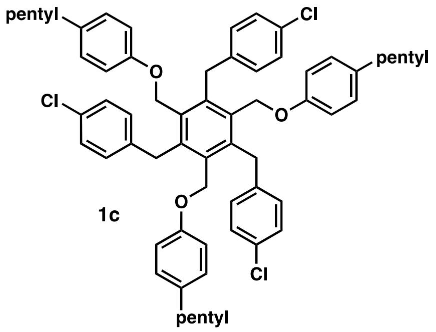

1c

4,4',4''-((2,4,6-Tris((4-pentylphenoxy)methyl)benzene-1,3,5-triyl)tris(methylene))-tris(chlorobenzene) (1c). General procedure B was applied to 4,4',4''-(((2,4,6-tris(chloromethyl)benzene-1,3,5-triyl)tris(methylene))tris(oxy))tris-(pentylbenzene) (1.24 g, 1.65 mmol, 1.0 equiv.) in 17 mL THF (0.1 M) with 4-chlorophenylmagnesium bromide in THF (1.0 M, 9.89 mL, 6.0 equiv.) and CuI (157 mg, 0.82 mmol, 0.5 equiv.) to obtain **1c** (720 mg, 45 % yield) as a white solid after purification by flash chromatography on silica gel (hexane/EtOAc = 98:2).
^{1}H-NMR (500 MHz, $CDCl_3$) δ 7.18 (d, J = 8.5 Hz, 6H), 7.01 (d, 6H), 6.93 (d, J = 8.3 Hz, 6H), 6.62 (d, J = 8.6 Hz, 6H), 4.80 (s, 6H), 4.25 (s, 6H), 2.51 (t, J = 7.7 Hz, 6H), 1.59 – 1.55 (m, 6H), 1.37 – 1.22 (m, 12H), 0.88 (t, J = 6.9 Hz, 9H).
^{13}C-NMR{^{1}H} (101 MHz, $CDCl_3$) δ 156.7, 141.8, 138.9, 135.8, 134.8, 132.0, 129.5, 129.4, 128.7, 114.3, 64.8, 35.2, 34.7, 31.6, 31.5, 22.7, 14.2.
HRMS (ESI) *m/z* calcd. for $C_{63}H_{69}O_3{}^{35}Cl_2{}^{37}Cl$+$NH_4$ [M+NH_4]$^+$ 998.4627, found 998.4627.

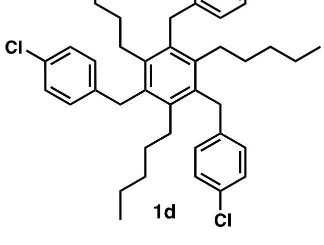

1d

4,4',4''-((2,4,6-tripentylbenzene-1,3,5-triyl)tris(methylene))tris(chlorobenzene) (1d). General procedure B was applied to 1,3,5-tris(chloromethyl)-2,4,6-tripentylbenzene (770 mg, 1.77 mmol, 1.0 equiv.) in 15 mL THF with 4-chlorophenylmagnesium bromide in THF (1.0 M, 10.7 mL, 6.0 equiv.) and CuI (169 mg, 887 µmol, 0.5 equiv.) to obtain

1d (842 mg, 71 % yield) as a colorless wax after purification by flash chromatography on silica gel (hexane = 100%).
^{1}H NMR (400 MHz, $CDCl_3$) *δ* 7.22 (d, *J* = 8.4 Hz, 6H), 6.90 (d, *J* = 8.3 Hz, 6H), 4.04 (s, 6H), 2.38 – 2.24 (m, 6H), 1.46 – 1.31 (m, 6H), 1.23 (q, *J* = 3.5 Hz, 12H), 0.89 – 0.75 (m, 9H).
^{13}C NMR{^{1}H} (101 MHz, $CDCl_3$) *δ* 140.3, 139.9, 134.1, 131.7, 129.1, 128.6, 34.4, 32.7, 31.1, 30.9, 22.5, 14.1.
HRMS (ESI) *m/z* calcd. for $C_{42}H_{51}{}^{35}Cl_2{}^{37}Cl_1{}^{107}Ag$ $[M+Ag]^+$ 769.2078, found 769.2104.

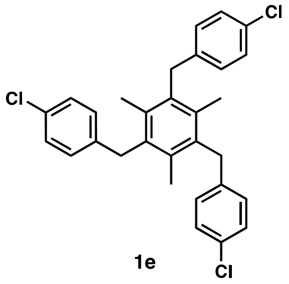

1e

4,4',4''-((2,4,6-Trimethylbenzene-1,3,5-triyl)tris(methylene))tris(chlorobenzene) (1e). General procedure B was applied to 1,3,5-tris(bromomethyl)-2,4,6-trimethylbenzene (487 mg, 1.22 mmol, 1.0 equiv.) in 12 mL THF (0.1 M) with 4-chlorophenylmagnesium bromide in THF (1.0 M, 7.32 mL, 6.0 equiv.) and CuI (116 mg, 0.61 mmol, 0.5 equiv.) to obtain **1e** (529 mg, 88 % yield) as a white solid after purification by flash chromatography on silica gel (hexane/EtOAc = 98:2).
^{1}H-NMR (500 MHz, $CDCl_3$) δ 7.23 (d, *J* = 8.4 Hz, 6H), 6.96 (d, *J* = 8.2 Hz, 6H), 4.11 (s, 6H), 2.13 (s, 9H).
^{13}C-NMR{^{1}H} (126 MHz, $CDCl_3$) δ 138.8, 135.0, 134.8, 131.6, 129.2, 128.7, 35.7, 16.9.
HRMS (ESI, AgTFA) *m/z* calcd. for $C_{30}H_{27}{}^{35}Cl_2{}^{37}Cl+NH_4$ $[M+Ag]^+$ 601.0179, found 601.0193.

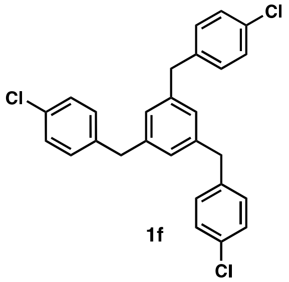

1f

1,3,5-Tris(4-chlorobenzyl)benzene (1f). General procedure B was applied to 1,3,5-tris(bromomethyl)benzene (1.00 g, 2.80 mmol, 1.0 equiv.) in 28 mL THF with 4-chlorophenylmagnesium bromide in THF (1.0 M, 16.8 mL, 6.0 equiv.) and CuI (267 mg, 1.40 mmol, 0.5 equiv.) to obtain tris(chlorobenzene) **1f** (921 mg, 73 % yield) as a white solid after purification by flash chromatography on silica gel (hexane/EtOAc = 98:2).
^{1}H-NMR (500 MHz, $CDCl_3$) *δ* 7.37 – 7.19 (m, 6H), 7.19 – 7.06 (m, 6H), 6.85 (s, 3H), 3.90 (s, 6H).
^{13}C-NMR{^{1}H} (126 MHz, $CDCl_3$) *δ* 141.3, 139.6, 132.0, 130.3, 128.7, 127.6, 41.2.
HRMS (ESI) *m/z* calcd. for $C_{27}H_{21}{}^{35}Cl_3+{}^{109}Ag$ $[M+Ag]^+$ 558.9756, found 558.9733.

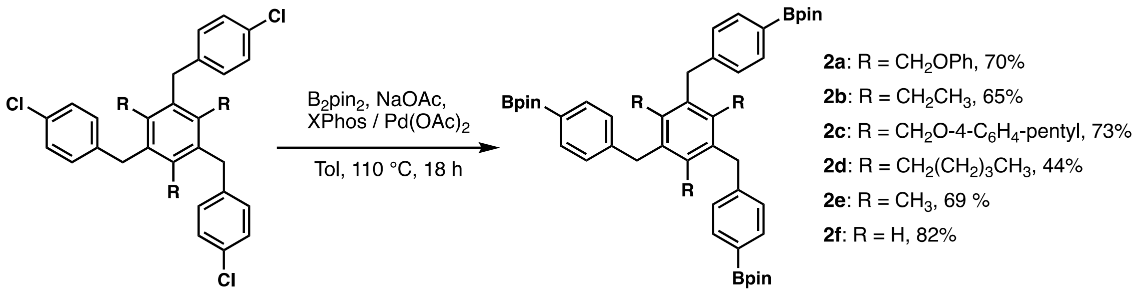

General procedure C for the palladium-catalyzed borylation of tris(chlorobenzenes). According to a modification of the literature procedure[3] a flame-dried round bottom flask equipped with a reflux condenser was charged with tris(chlorobenzene) (1.00 equiv.), B_2pin_2 (3.6 equiv.), anhydrous NaOAc (6.0 equiv.), $Pd(OAc)_2$ (0.1 equiv.) and XPhos (0.2 equiv.). The flask was evacuated and backfilled with nitrogen three times and anhydrous toluene (0.2 M) was added. The resulting mixture was heated to reflux for 18 h. The mixture was allowed to cool to room temperature, quenched with water and extracted three times with CH_2Cl_2. The combined organic layers were dried over anhydrous Na_2SO_4, filtered and concentrated *in vacuo*. The crude product was purified by flash chromatography on silica gel to yield the tris(pinacolboronate) as a white solid.

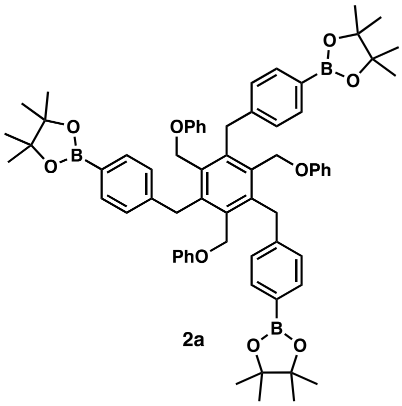

2a

2,2',2''-(((2,4,6-Tris(phenoxymethyl)benzene-1,3,5-triyl)tris(methylene))tris(benzene-4,1-diyl))tris(4,4,5,5-tetramethyl-1,3,2-dioxaborolane) (2a). General procedure C was applied to 4,4',4''-((2,4,6-tris(phenoxymethyl)benzene-1,3,5-triyl)tris(methylene))tris(chlorobenzene) (**1a**) (430 mg, 558 µmol, 1.00 equiv) using B_2pin_2 (510 mg, 2.01 mmol, 3.6 equiv.), anhydrous NaOAc (275 mg, 3.35 mmol, 6.0 equiv.), $Pd(OAc)_2$ (12.5 mg, 55.8 µmol, 0.1 equiv.) and XPhos (53.2 mg, 112 µmol, 0.2 equiv.) in 2.8 mL anhydrous toluene. Purification by flash chromatography on silica gel (hexane/EtOAc = 85:15) gave tris(pinacolboronate) **2a** (407 mg, 70 % yield) as a white solid.

^{1}H-NMR (500 MHz, $CDCl_3$) δ 7.68 (d, J = 8.0 Hz, 6H), 7.20 (dd, J = 8.7, 7.3 Hz, 6zH), 6.99 (dt, J = 8.0 Hz, 6H), 6.91 (dt, J = 7.5, 1.1 Hz, 3H), 6.76 (dd, J = 8.8, 1.1 Hz, 6H), 4.81 (s, 6H), 4.32 (s, 6H), 1.35 (s, 36H).

^{13}C-NMR{^{1}H} (126 MHz, $CDCl_3$) δ 158.9, 143.8, 141.9, 135.2, 134.6, 129.5, 127.5, 121.1, 114.6, 83.9, 83.7, 64.7, 35.3, 25.0.

^{1}H DOSY-NMR (500 MHz, $CDCl_3$): D = 5.13 ± 0.02 x 10^{-10} m^2/s.

HRMS (ESI) *m/z* calcd. for $C_{66}H_{75}{}^{11}B_3O_9Na$ $[M+Na]^+$ 1067.5588, found 1067.5630.

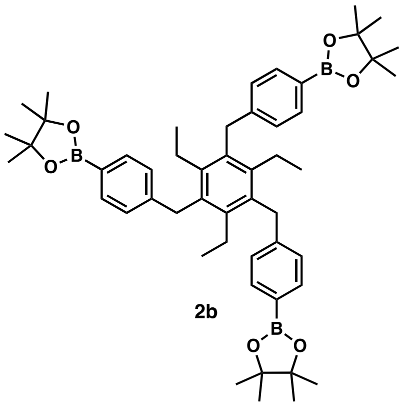

2b

2,2',2''-(((2,4,6-Triethylbenzene-1,3,5-triyl)tris(methylene))tris(benzene-4,1-diyl))tris(4,4,5,5-tetramethyl-1,3,2-dioxaborolane) (2b). General procedure C was applied to 4,4',4''-((2,4,6-triethylbenzene-1,3,5-triyl)tris(methylene))tris(chlorobenzene) (**1b**) (411 mg, 768 µmol, 1.0 equiv.) using B_2pin_2 (701 mg, 2.76 mmol, 3.6 equiv.), anhydrous NaOAc (277 mg, 4.60 mmol, 6.0 equiv.), $Pd(OAc)_2$ (17.2 mg, 76.7 µmol, 0.1 equiv.) and XPhos (73.1 mg, 153 µmol, 0.2 equiv.) in 3.8 mL anhydrous toluene. Purification by flash chromatography on silica gel (hexane/EtOAc = 9:1) gave tris(pinacolboronate) **2b** (403 mg, 65 % yield) as a white solid.

^{1}H-NMR (500 MHz, $CDCl_3$) δ 7.69 (d, J = 8.0 Hz, 6H), 7.00 (d, J = 7.5 Hz, 6H), 4.14 (s, 6H), 2.39 (q, J = 7.4 Hz, 6H), 1.34 (s, 36H), 1.08 (t, J = 7.4 Hz, 9H).

^{13}C-NMR{^{1}H} (126 MHz, $CDCl_3$) δ 144.8, 141.5, 135.5, 135.1, 133.8, 127.3, 83.7, 34.9, 25.0, 23.8, 15.3.

^{1}H DOSY-NMR (500 MHz, $CDCl_3$): $D = 5.71 \pm 0.03 \times 10^{-10}$ m^2/s.

HRMS (ESI) m/z calcd. for $C_{51}H_{69}{}^{11}B_3O_6Na$ $[M+Na]^+$ 833.5271, found 833.5273.

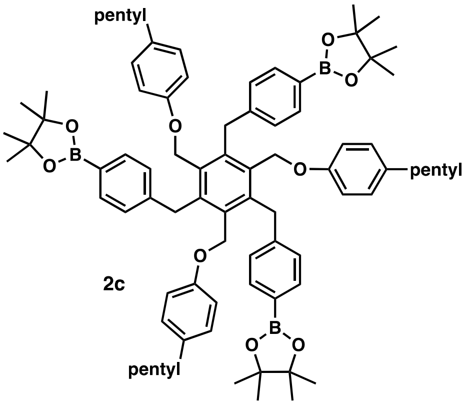

2c

2,2',2''-(((2,4,6-tris((4-pentylphenoxy)methyl)benzene-1,3,5-triyl)tris(methylene))tris(benzene-4,1-diyl))tris(4,4,5,5-tetramethyl-1,3,2-dioxaborolane) (2c). General procedure C was applied to 4,4',4''-((2,4,6-tris((4-pentylphenoxy)methyl)benzene-1,3,5-triyl)tris(methylene))-tris(chlorobenzene) (**1c**) (1.05 g, 1.07 mmol, 1.0 equiv.) using B_2pin_2 (978 mg, 3.85 mmol, 3.6 equiv.), anhydrous NaOAc (527 mg, 6.42 mmol, 6.0 equiv.), $Pd(OAc)_2$ (24.0 mg, 107.1 µmol, 0.1 equiv.), XPhos (102 mg, 214 µmol, 0.2 equiv.) in 5 mL (0.2 M) anhydrous toluene. Purification by flash chromatography on silica gel (hexane/EtOAc = 9:1) gave tris(pinacolboronate) **2c** (981 mg, 73 % yield) as a white solid.

^{1}H-NMR (400 MHz, $CDCl_3$) δ 7.74 (d, J = 8.0 Hz, 6H), 7.04 (d, J = 7.9 Hz, 6H), 7.01 (d, J = 8.6 Hz, 6H), 6.72 (d, J = 8.6 Hz, 6H), 4.93 (s, 6H), 4.37 (s, 6H), 3.27 (s, 6H), 2.47

(t, J = 7.7 Hz, 6H), 1.60 – 1.41 (m, 6H), 1.26 (pd, J = 2.1, 5.3 Hz, 12H), 0.82 (t, J = 6.9 Hz, 9H).
^{13}C-NMR{^{1}H} (101 MHz, $CDCl_3$) δ 157.8, 143.4, 142.6, 136.0, 135.5, 135.2, 132.1, 130.0, 127.9, 115.1, 65.7, 35.5, 35.4, 32.0, 32.0, 23.0, 14.2.
^{1}H DOSY-NMR (500 MHz, $CDCl_3$): $D = 4.09 \pm 0.05 \times 10^{-10}$ m^2/s.
HRMS (ESI) *m/z* calcd. for $C_{81}H_{105}{}^{11}B_3O_9Na$ [M+Na]$^+$ 1277.7935, found 1277.7961.

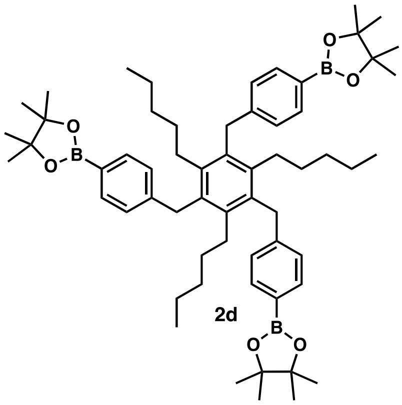

2,2',2''-(((2,4,6-tripentylbenzene-1,3,5-triyl)tris(methylene))tris(benzene-4,1-diyl))tris(4,4,5,5-tetramethyl-1,3,2-dioxaborolane) (2d). General procedure C was applied to 4,4',4''-((2,4,6-tripentylbenzene-1,3,5-triyl)tris(methylene))tris(chlorobenzene) (**1d**). (420 mg, 634 µmol, 1.0 equiv.) using B_2pin_2 (580 mg, 2.28 mmol, 3.6 equiv.), anhydrous NaOAc (312 mg, 3.81 mmol, 6.0 equiv.), $Pd(OAc)_2$ (14.2 mg, 63.4 µmol, 0.1 equiv.), XPhos (60.5 mg, 126.9 µmol, 0.2 equiv.) in 6 mL anhydrous toluene. Purification by flash chromatography on silica gel (hexane/EtOAc = 95:5) gave tris(pinacolboronate) **2d** (260.1 mg, 44 % yield) as a white solid.
^{1}H NMR (400 MHz, $CDCl_3$) δ 7.68 (d, J = 7.8 Hz, 6H), 6.98 (d, J = 7.8 Hz, 6H), 4.09 (s, 6H), 2.35 – 2.24 (m, 6H), 1.47 (d, J = 13.0 Hz, 6H), 1.33 (s, 36H), 1.21 (m, 12H), 0.88 – 0.77 (m, 9H).
^{13}C NMR{^{1}H} (126 MHz, $CDCl_3$) δ 144.9, 140.3, 135.1, 134.0, 127.3, 126.2, 83.7, 35.2, 32.6, 30.9, 25.0, 22.5, 14.1.
HRMS (ESI) *m/z* calcd. for $C_{60}H_{87}{}^{11}B_3O_6Na$ [M+Na]$^+$ 959.6680, found 959.6708.

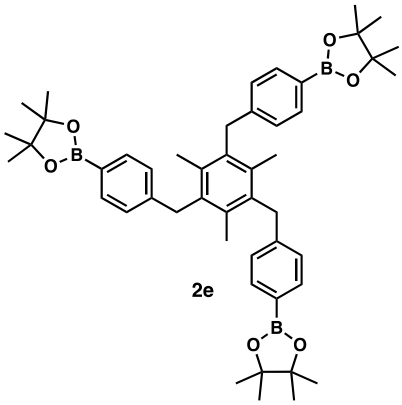

2,2',2''-(((2,4,6-Trimethylbenzene-1,3,5-triyl)tris(methylene))tris(benzene-4,1-diyl))tris(4,4,5,5-tetramethyl-1,3,2-dioxaborolane) (2e). General procedure C was applied to 4,4',4''-((2,4,6-trimethylbenzene-1,3,5-triyl)tris(methylene))tris(chlorobenzene) (**1e**) (451 mg, 913 µmol, 1.0 equiv.) using B_2pin_2 (834 mg, 3.29 mmol, 3.6

equiv.), anhydrous NaOAc (450 mg, 5.48 mmol, 6.0 equiv.), $Pd(OAc)_2$ (20.5 mg, 91.3 µmol, 0.1 equiv.), XPhos (87.1 mg, 182 µmol, 0.2 equiv.) in 5 mL anhydrous toluene. Purification by flash chromatography on silica gel (hexane/EtOAc = 9:1) gave tris(pinacolboronate) **2e** (601.5 mg, 69 % yield) as a white solid.
^{1}H-NMR (500 MHz, $CDCl_3$) δ 7.70 (d, *J* = 8.0 Hz, 6H), 7.04 (d, *J* = 7.8 Hz, 6H), 4.17 (s, 6H), 2.11 (s, 9H), 1.33 (s, 36H).
^{13}C-NMR{^{1}H} (126 MHz, $CDCl_3$) δ 144.0, 135.1, 135.0, 134.7, 127.4, 126.2, 83.7, 36.5, 25.0, 16.9.
HRMS (ESI) *m/z* calcd. for $C_{48}H_{63}{}^{11}B_3O_6Na$ $[M+Na]^+$ 791.4802, found 791.4844.

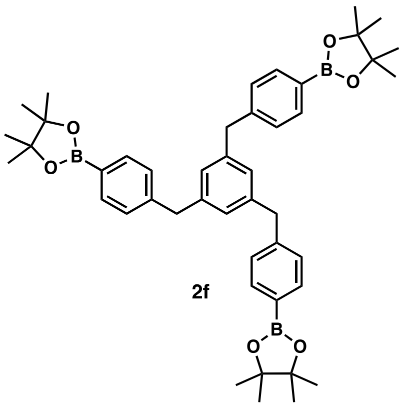

2f

1,3,5-Tris(4-(4,4,5,5-tetramethyl-1,3,2-dioxaborolan-2-yl)benzyl)benzene (2f). General procedure C was applied to 1,3,5-tris(4-chlorobenzyl)benzene (**1f**) (450 mg, 996 µmol, 1.0 equiv.) using B_2pin_2 (911 mg, 3.59 mmol, 3.6 equiv.), anhydrous NaOAc (490 mg, 5.98 mmol, 6.0 equiv.), $Pd(OAc)_2$ (22.4 mg, 99.6 µmol, 0.1 equiv.), XPhos (95.0 mg, 199 µmol, 0.2 equiv.) in 5 mL anhydrous toluene. Purification by flash chromatography on silica gel (hexane/EtOAc = 85:15) gave tris(pinacolboronate) **2f** (594 mg, 82 % yield) as a white solid.
^{1}H-NMR (500 MHz, $CDCl_3$) δ 7.72 (d, *J* = 8.0 Hz, 6H), 7.15 (d, *J* = 7.9 Hz, 6H), 6.83 (s, 3H), 3.88 (s, 6H), 1.34 (s, 36H).
^{13}C-NMR{^{1}H} (126 MHz, $CDCl_3$) δ 144.6, 141.2, 135.1, 128.5, 127.7, 83.8, 83.6, 42.1, 25.0.
HRMS (ESI) *m/z* calcd. for $C_{45}H_{57}{}^{11}B_3O_6Na$ $[M+Na]^+$ 749.4332, found 749.4336.

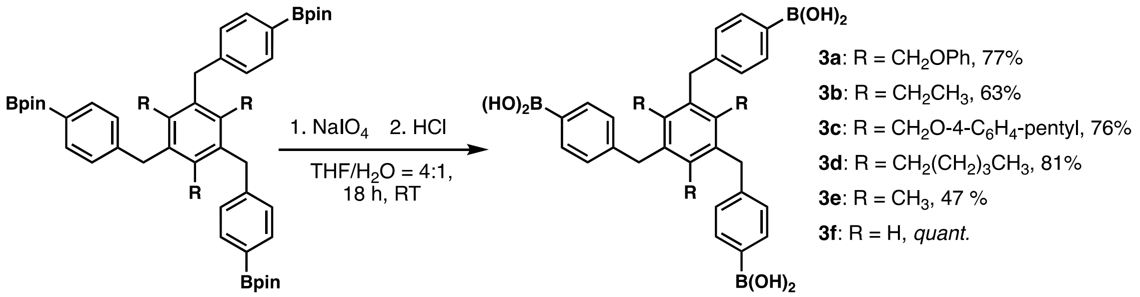

General procedure D for the deprotection of tris(pinacolboronates). According to the literature procedure,[4] tris(pinacolboronate) was dissolved in a 4:1 mixture of THF/H_2O (0.09 M:0.35 M) and $NaIO_4$ (12.0 equiv.) was added. The resulting mixture was stirred at room temperature for 30 min following addition of HCl (1.0 M, 3.0 equiv.) and additional stirring for 18 h. Upon completion of the reaction, water was added to dissolve the precipitated salts and the aqueous phase was extracted three times with EtOAc. The combined organic layers were dried over anhydrous Na_2SO_4, filtered and concentrated *in vacuo*. The crude product was purified by flash chromatography on silica gel to yield the tris(boronic acid) as an off-white solid. The NMR-spectra were recorder in acetone-d_6 with one drop of D_2O to facilitate solvation and prevent boronic acid condensation.

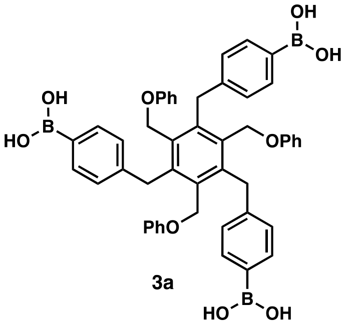

3a

(((2,4,6-Tris(phenoxymethyl)benzene-1,3,5-triyl)tris(methylene))tris(benzene-4,1-diyl))triboronic acid (3a). General procedure D was applied to 2,2',2''-(((2,4,6-tris(phenoxymethyl)benzene-1,3,5-triyl)tris(methylene))tris(benzene-4,1-diyl))tris(4,4,5,5-tetramethyl-1,3,2-dioxaborolane) (**2a**) (823 mg, 788 µmol, 1.0 equiv.) using $NaIO_4$ (2.02 g, 9.45 mmol, 12 equiv.), HCl (2.36 mL, 1.0 M, 2.36 mmol, 3.0 equiv.) in 11 mL THF/H_2O. Purification by flash chromatography on silica gel (CH_2Cl_2/MeOH = 95:5) gave triboronic acid **3a** (486 mg, 77 % yield) as an off-white solid.

^{1}H-NMR (500 MHz, acetone-d_6/D_2O) δ 7.73 (d, J = 8.0 Hz, 6H), 7.19 (td, J = 7.3, 2.1 Hz, 6H), 7.04 (d, J = 8.1 Hz, 6H), 6.88 (t, J = 7.3 Hz, 3H), 6.79 (d, J = 7.8 Hz, 6H), 4.98 (s, 6H), 4.37 (s, 6H), 3.27 (s, 6H).

^{13}C-NMR{^{1}H} (126 MHz, acetone-d_6/D_2O) δ 159.7, 143.3, 142.7, 135.3, 135.2, 130.2, 127.9, 121.8, 115.2, 65.5, 49.4, 35.5.

Elemental analysis (%) calcd for $C_{48}H_{45}B_3O_9$: C, 72.22; H, 5.68; found: C, 72.09; H, 5.77

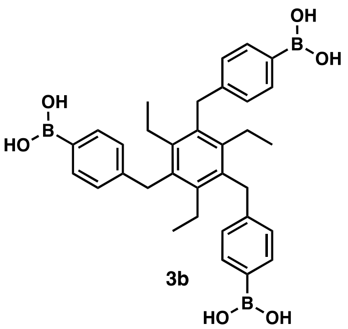

3b

(((2,4,6-Triethylbenzene-1,3,5-triyl)tris(methylene))tris(benzene-4,1-diyl))-triboronic acid (3b). General procedure D was applied to 2,2',2''-(((2,4,6-triethylbenzene-1,3,5-triyl)tris(methylene))tris(benzene-4,1-diyl))tris(4,4,5,5-tetramethyl-1,3,2-dioxaborolane) (**2b**) (403 mg, 497 µmol, 1.0 equiv.) using $NaIO_4$ (1.28 g, 5.97 mmol, 12 equiv.), HCl (1.49 mL, 1.0 M, 1.49 mmol, 3.0 equiv.) in 7 mL THF/H_2O. Purification by flash chromatography on silica gel (CH_2Cl_2/MeOH = 9:1) gave triboronic acid **3b** (175 mg, 63 % yield) as an off-white solid.

^{1}H-NMR (400 MHz, acetone-d_6/D_2O) δ 7.73 (d, J = 8.2 Hz, 6H), 7.02 (d, J = 8.3 Hz, 6H), 4.17 (s, 6H), 3.26 (s, 6H), 2.45 (q, J = 7.4 Hz, 6H), 1.00 (t, J = 7.4 Hz, 9H).

^{13}C-NMR{^{1}H} (126 MHz, acetone-d_6/D_2O) δ 144.5, 141.9, 135.1, 134.6, 127.7, 49.4, 35.2, 24.2, 15.4.

Elemental analysis (%) calcd for $C_{33}H_{39}B_3O_6$: C, 70.26; H, 6.97; found: C, 70.22; H, 7.32.

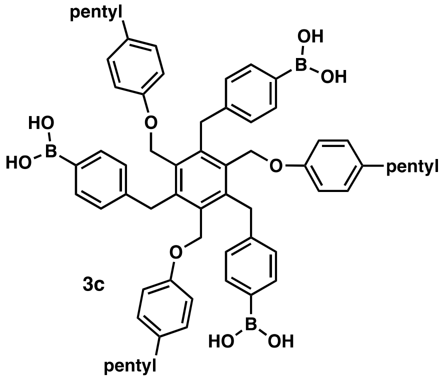

3c

(((2,4,6-Tris((4-pentylphenoxy)methyl)benzene-1,3,5-triyl)tris(methylene))tris-(benzene-4,1-diyl))triboronic acid (3c). General procedure C was applied to 2,2',2''-(((2,4,6-tris(phenoxymethyl)benzene-1,3,5-triyl)tris(methylene))tris-(benzene-4,1-diyl)) tris(4,4,5,5-tetramethyl-1,3,2-dioxaborolane) (**2c**) (981 mg, 782 µmol, 1.0 equiv.) using $NaIO_4$ (2.01 g, 9.38 mmol, 12 equiv.), HCl (2.34 mL, 1.0 M, 2.34 µmol, 3.0 equiv.) in 10 mL THF/H_2O. Purification by flash chromatography on silica gel (CH_2Cl_2/MeOH = 9:1) gave triboronic acid **3c** (596 mg, 76%*)* as an off-white solid.

^{1}H-NMR (400 MHz, acetone-d_6/D_2O) δ 7.74 (d, J = 8.0 Hz, 6H), 7.03 (dd, J = 8.2, 10.3 Hz, 12H), 6.79 – 6.66 (m, 6H), 4.93 (s, 6H), 4.37 (s, 6H), 3.27 (s, 6H), 2.47 (t, J = 7.7 Hz, 6H), 1.60 – 1.41 (m, 6H), 1.26 (pd, J = 2.1, 5.3 Hz, 12H), 0.82 (t, J = 6.9 Hz, 9H).

^{13}C NMR{^{1}H} (101 MHz, acetone-d_6/D_2O) δ 157.8, 143.4, 142.6, 136.0, 135.5, 135.2, 132.1, 130.0, 127.9, 115.1, 65.7, 35.5, 35.4, 32.0, 32.0, 23.0, 14.2.

Elemental analysis (%) calcd for $C_{63}H_{75}B_3O_9$: C, 75.02; H, 7.49; found: C, 75.35; H, 7.23.

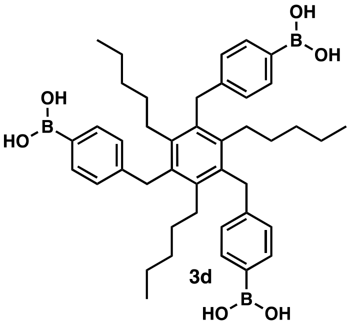

3d

(((2,4,6-Tripentylbenzene-1,3,5-triyl)tris(methylene))tris(benzene-4,1-diyl))triboronic acid (**3d**). General procedure D was applied to (**2d**) (225 mg, 240 µmol, 1.0 equiv.) using $NaIO_4$ (617 mg, 2.88 mmol, 12 equiv.), HCl (0.96 mL, 1.0 M, 0.96 mmol, 3.0 equiv.) in 4 mL THF/H_2O. Purification by flash chromatography on silica gel (CH_2Cl_2/MeOH = 95:5) gave triboronic acid **3d** (133.5 mg, 81 % yield) as a white solid.
^{1}H NMR (500 MHz, acetone-*d*$_6$/D_2O) *δ* 7.76 (d, *J* = 7.9 Hz, 6H), 7.04 (d, *J* = 7.8 Hz, 6H), 4.17 (s, 6H), 3.29 (s, 6H), 2.56 – 2.32 (m, 6H), 1.48 – 1.36 (m, 6H), 1.22 (dq, *J* = 4.4, 9.2 Hz, 12H), 0.83 – 0.73 (m, 8H).
^{13}C NMR{^{1}H} (126 MHz, acetone-*d*$_6$/D_2O) *δ* 144.7, 140.7, 135.2, 135.0, 131.8, 127.8, 35.6, 33.2, 31.6, 31.5, 22.9, 14.2.
Elemental analysis (%) calcd for $C_{42}H_{57}B_3O_6$: C, 73.07; H, 8.32; found: C, 73.04; H, 8.25.

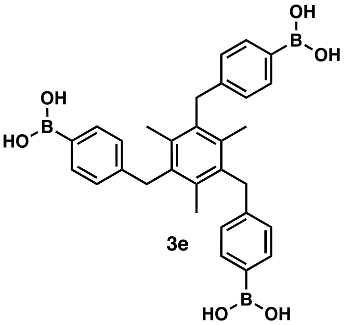

3e

(((2,4,6-Trimethylbenzene-1,3,5-triyl)tris(methylene))tris(benzene-4,1-diyl))triboronic acid (3e). General procedure C was applied to 2,2',2''-(((2,4,6-trimethylbenzene-1,3,5-triyl)tris(methylene))tris(benzene-4,1-diyl))tris(4,4,5,5-tetramethyl-1,3,2-dioxaborolane) (**2e**) (361 mg, 470 µmol, 1.0 equiv.) using $NaIO_4$ (1.21 g, 5.64 mmol, 12 equiv.), HCl (1.41 mL, 1.0 M, 1.41 µmol, 3.0 equiv.) in 7 mL THF/H_2O. The reaction was diluted with water and the white solid was filtrated, washed with water and dried at high vacuum. Double purification by flash chromatography on silica gel (CH_2Cl_2/MeOH = 95:5) gave triboronic acid **3e** (114 mg, 47 %*)* as an off-white solid.
^{1}H-NMR (400 MHz, acetone-*d*$_6$/D_2O) δ 7.76 (d, *J* = 8.0 Hz, 6H), 7.04 (d, *J* = 7.7 Hz, 6H), 4.18 (s, 6H), 3.28 (s, 6H), 2.14 (s, 9H).
^{13}C NMR{^{1}H} (126 MHz, acetone-*d*$_6$/D_2O) δ 143.6, 135.7, 135.2, 135.2, 131.7, 127.8, 36.7, 17.0.
Elemental analysis (%) calcd for $C_{63}H_{75}B_3O_9$: C, 69.03; H, 6.37; found: C, 69.14; H, 6.65.

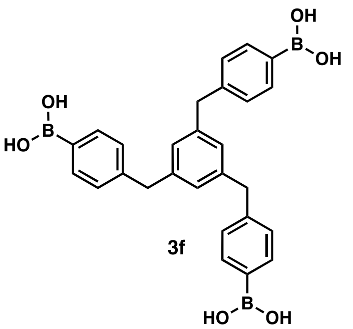

3f

((Benzene-1,3,5-triyltris(methylene))tris(benzene-4,1-diyl))triboronic acid (3f). General procedure C was applied to 1,3,5-tris(4-(4,4,5,5-tetramethyl-1,3,2-dioxaborolan-2-yl)benzyl)benzene (**2f**) (200 mg, 275 µmol, 1.0 equiv.) using $NaIO_4$ (706 mg, 3.30 mmol, 12 equiv.), HCl (826 µL, 1.0 M, 826 µmol, 3.0 equiv.) in 4 mL THF/H_2O. Work-up gave triboronic acid **3f** (133 mg, quantitative yield*)* as a white solid with sufficient purity to proceed without further purification.

^{1}H-NMR (500 MHz, acetone-*d*$_6$/D_2O) δ 7.74 (d, J = 8.0 Hz, 6H), 7.15 (d, J = 7.9 Hz, 6H), 6.96 (s, 3H), 3.88 (s, 6H), 3.27 (s, 6H).

^{13}C-NMR{^{1}H} (126 MHz, acetone-*d*$_6$/D_2O) δ 144.4, 142.3, 135.2, 128.7, 128.1, 49.4, 42.3.

Elemental analysis (%) calcd for $C_{27}H_{27}B_3O_6$: C, 67.57; H, 5.67; found: C, 67.58; H, 5.79

*side note: For the deprotection of the tris(pinacolboronates), purification by column chromatography is not ideal due to considerable loss of product in the column. However, in most of the cases it proved necessary due to remove small quantities of undesired side-products from the crude product. Alternatively, the crude product can be dissolved in EtOAc or CH_2Cl_2 and precipitated with *n*-pentane to afford the product after filtration and drying at high vacuum.

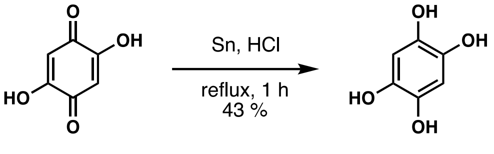

Benzene-1,2,4,5-tetraol (THB). Following the literature procedure[5] 2,5-dihydroxycyclohexa-2,5-diene-1,4-dione (3.20 g, 22.8 mmol, 1.0 equiv.) was dissolved in 70 mL *conc*. HCl and granulated Sn (3.25 g, 27.4 mmol, 1.2 equiv.) were carefully added and refluxed for 1 h. During this time, the reaction mixture turned from orange to black. After completion the reaction was filtered hot through a glass frit and after cooling to RT, it was left to crystalize at 0 °C over the weekend. The black crystal were recrystallized from THF to give benzene-1,2,4,5-tetraol (**THB**) (1.4 g, 43 % yield) as a light-grey solid.

^{1}H-NMR (500 MHz, DMSO-*d*$_6$) δ 7.94 (br s, 4H), 6.20 (s, 2H).

^{13}C-NMR{^{1}H} (126 MHz, DMSO-*d*$_6$) δ 137.0, 104.7.

HRMS (ESI) *m/z* calcd. for $C_6H_5O_4$ [M-H]$^-$ 141.0188, found 141.0186.

4. NMR Spectra

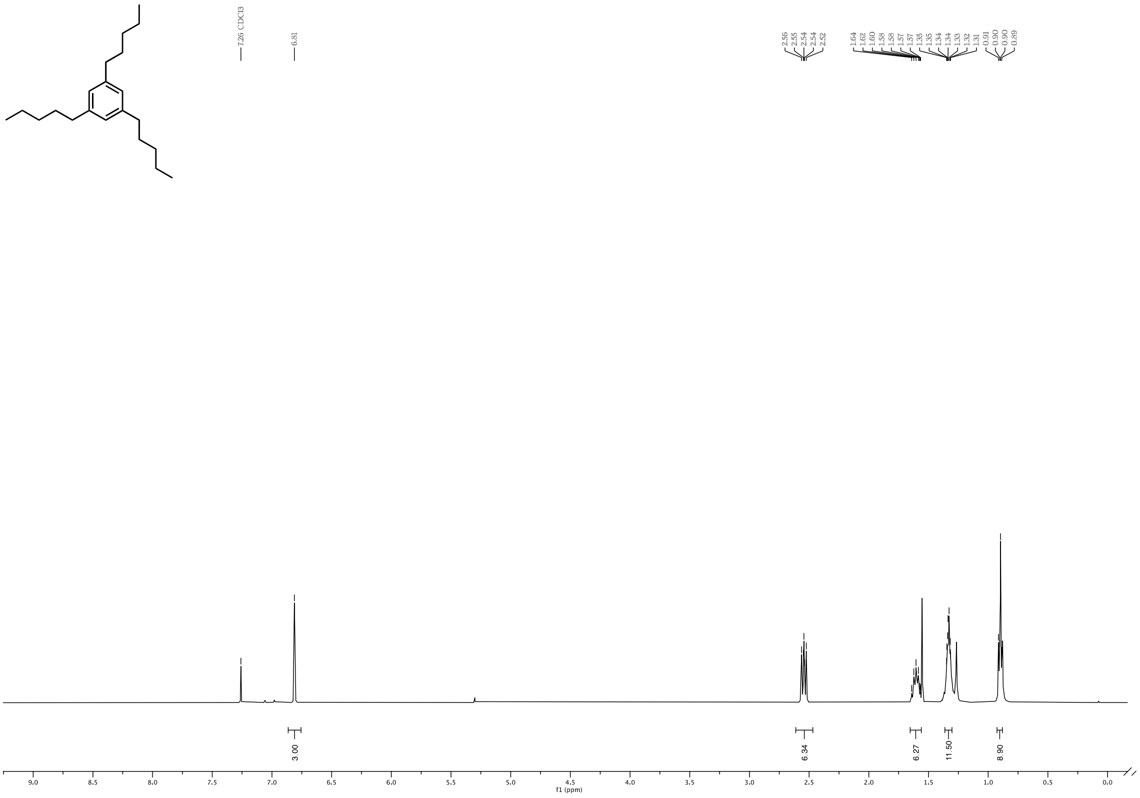

Figure S1: ^{1}H-NMR spectrum of 1,3,5-tripentylbenzene (400 MHz, $CDCl_3$).

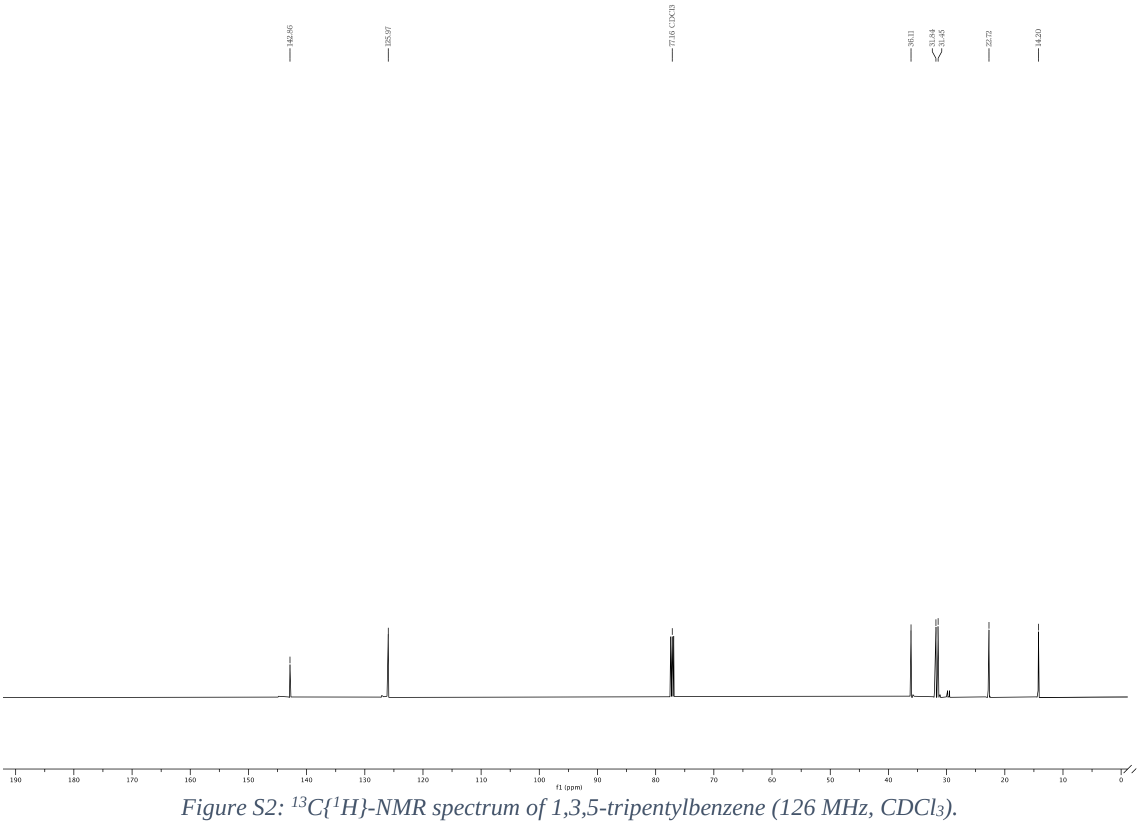

Figure S2: $^{13}C\{^{1}H\}$-NMR spectrum of 1,3,5-tripentylbenzene (126 MHz, $CDCl_3$).

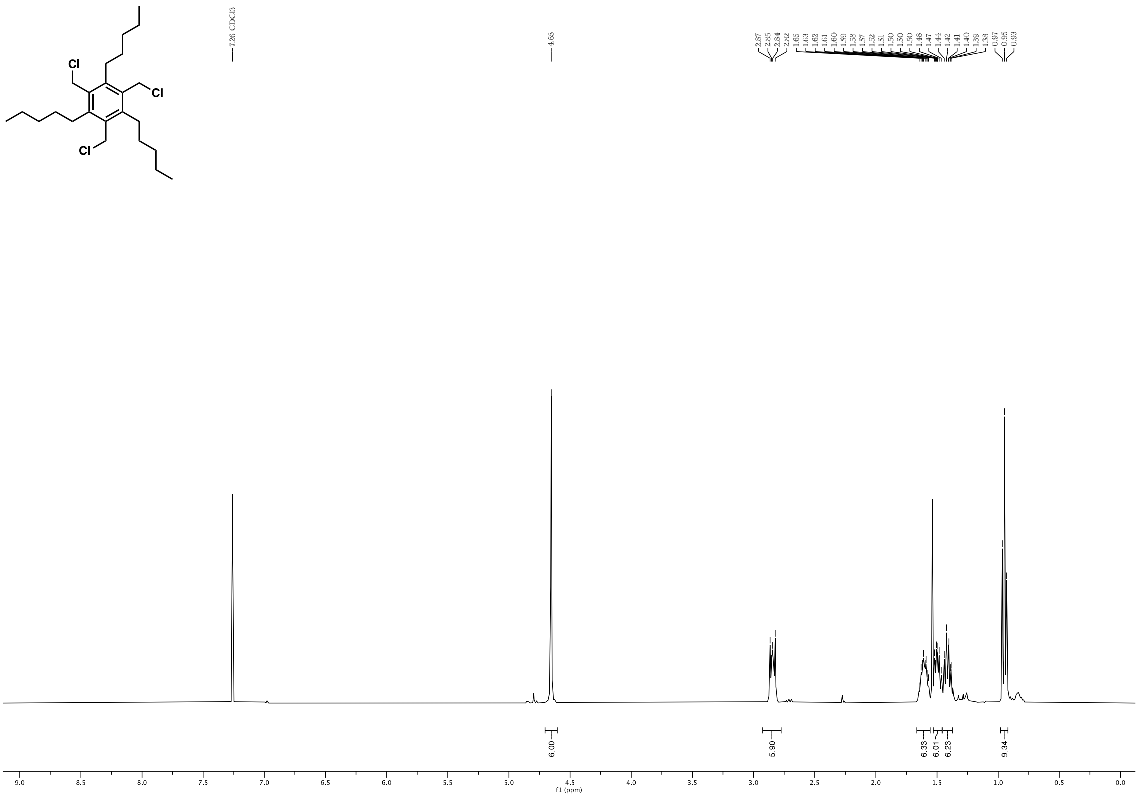

Figure S3: ^{1}H-NMR spectrum of 1,3,5-tris(chloromethyl)-2,4,6-tripentylbenzene (400 MHz, $CDCl_3$).

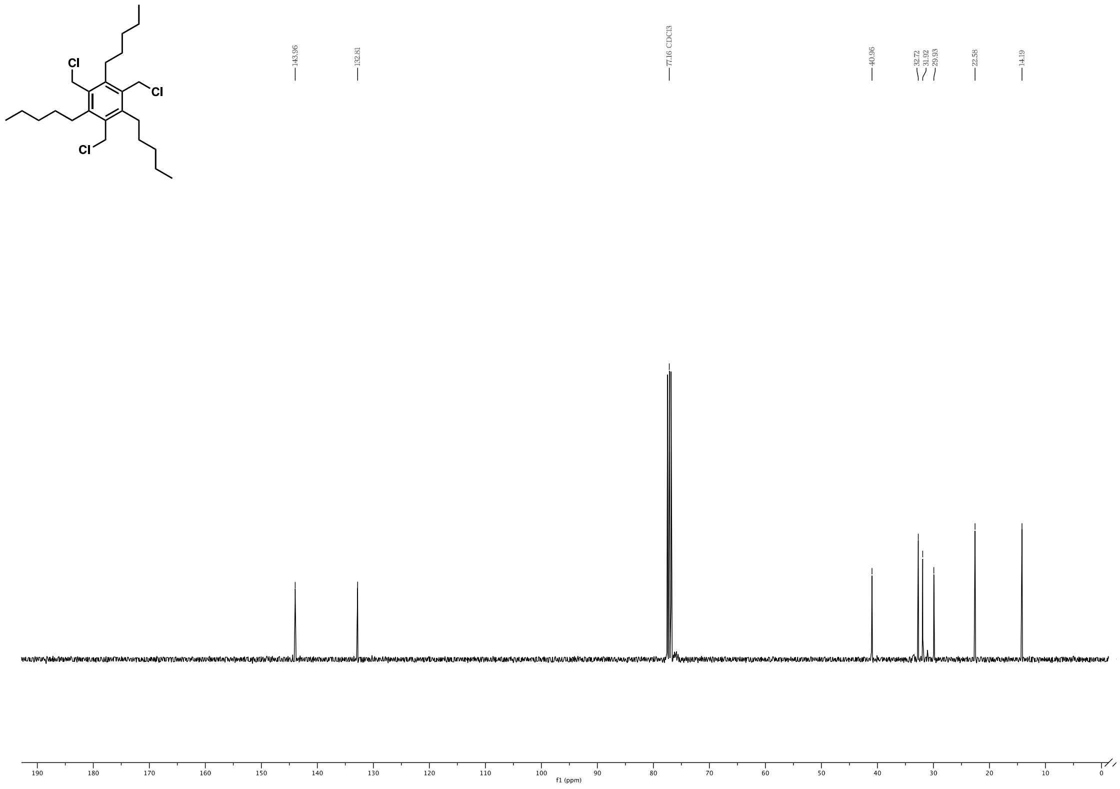

Figure S4: $^{13}C\{^{1}H\}$-NMR spectrum of 1,3,5-tris(chloromethyl)-2,4,6-tripentylbenzene (101 MHz, $CDCl_3$).

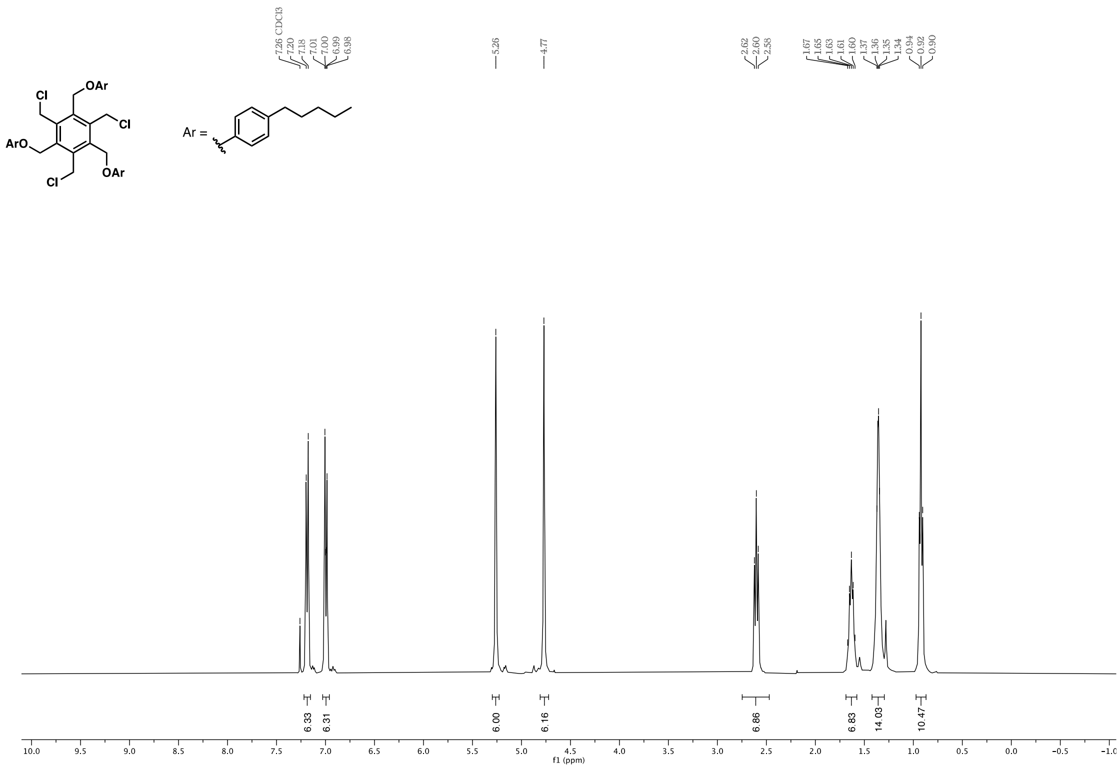

Figure S5: ^{1}H-NMR spectrum of 4,4',4''-(((2,4,6-tris(chloromethyl)benzene-1,3,5-triyl)tris(methylene))tris(oxy))tris(pentylbenzene)(400 MHz, $CDCl_3$).

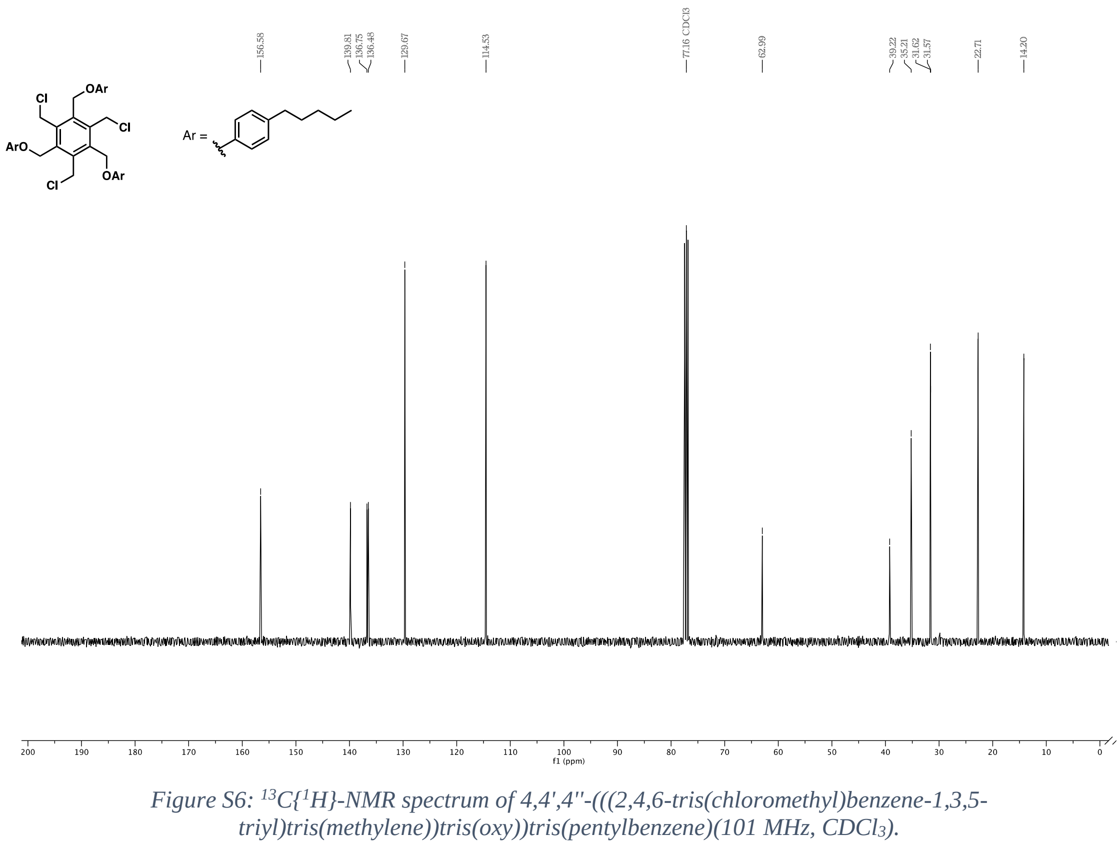

Figure S6: ^{13}C{^{1}H}-NMR spectrum of 4,4',4''-(((2,4,6-tris(chloromethyl)benzene-1,3,5-triyl)tris(methylene))tris(oxy))tris(pentylbenzene)(101 MHz, $CDCl_3$).

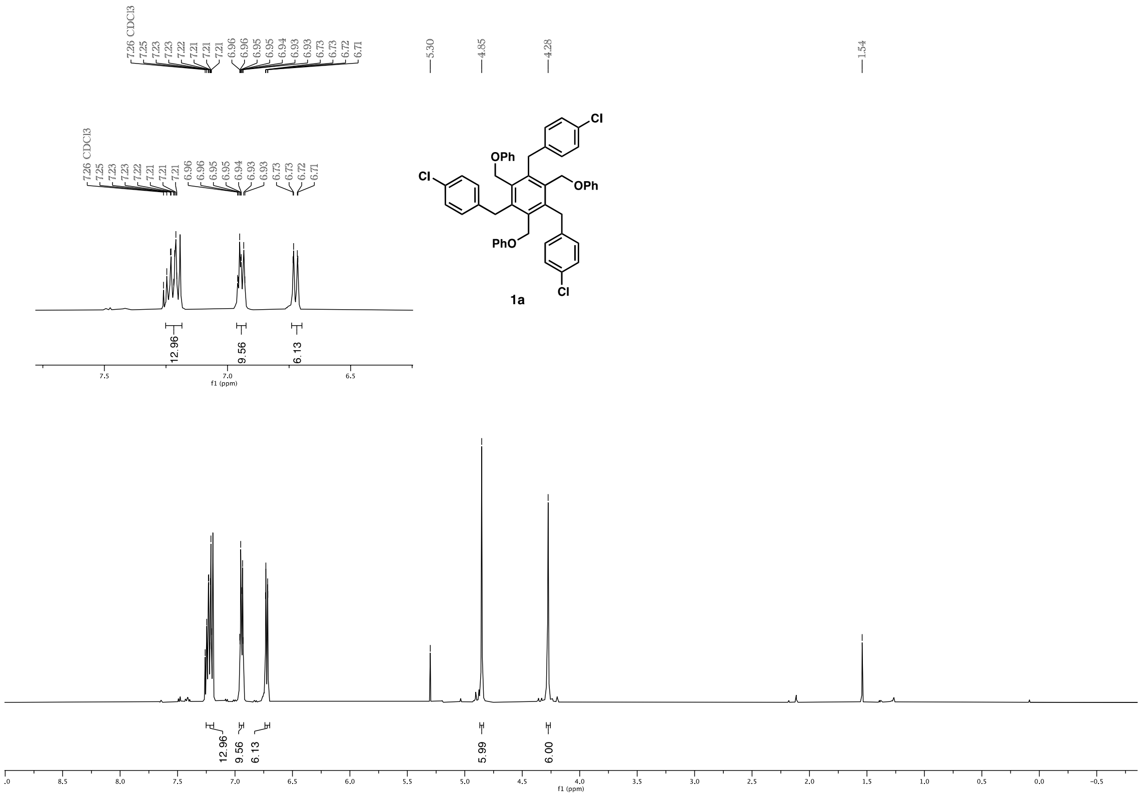

*Figure S7: ^{1}H-NMR spectrum of 4,4',4''-((2,4,6-Tris(phenoxymethyl)benzene-1,3,5-triyl)tris(methylene))tris (chlorobenzene) (**1a**), (500 MHz, $CDCl_3$)*

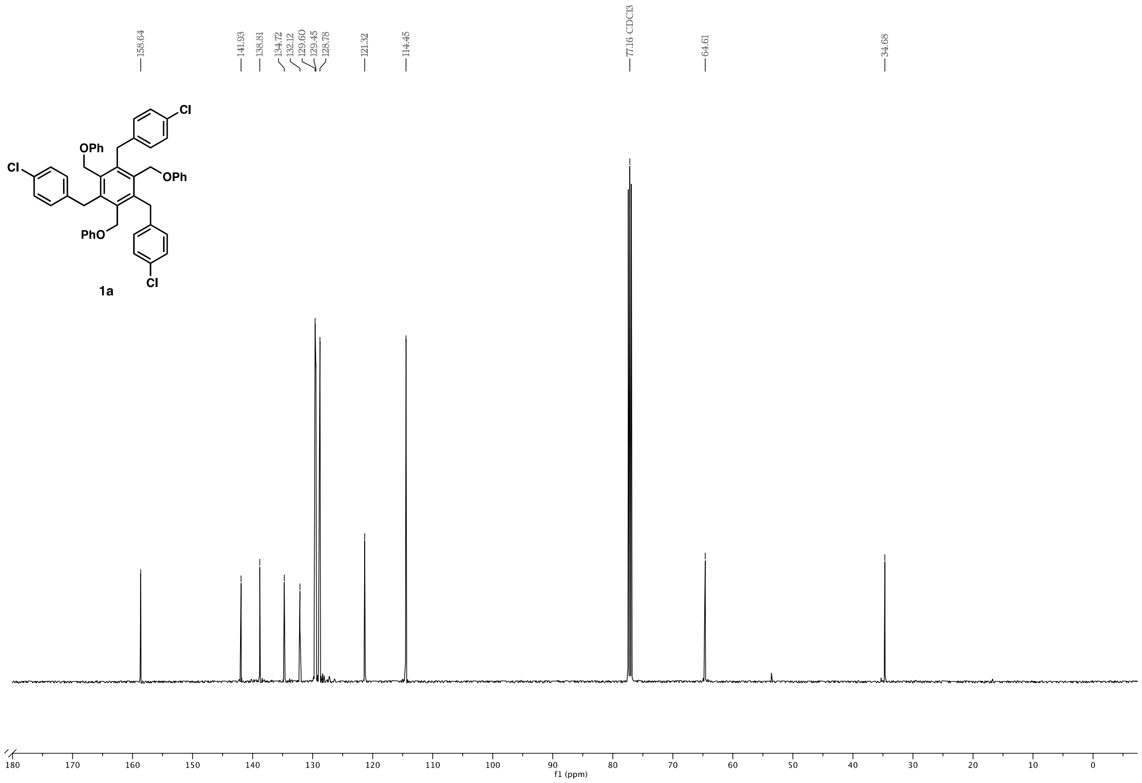

*Figure S8: ^{13}C{^{1}H}-NMR spectrum of 4,4',4''-((2,4,6-Tris(phenoxymethyl)benzene-1,3,5-triyl)tris(methylene))tris (chlorobenzene) (**1a**), (126 MHz, $CDCl_3$).*

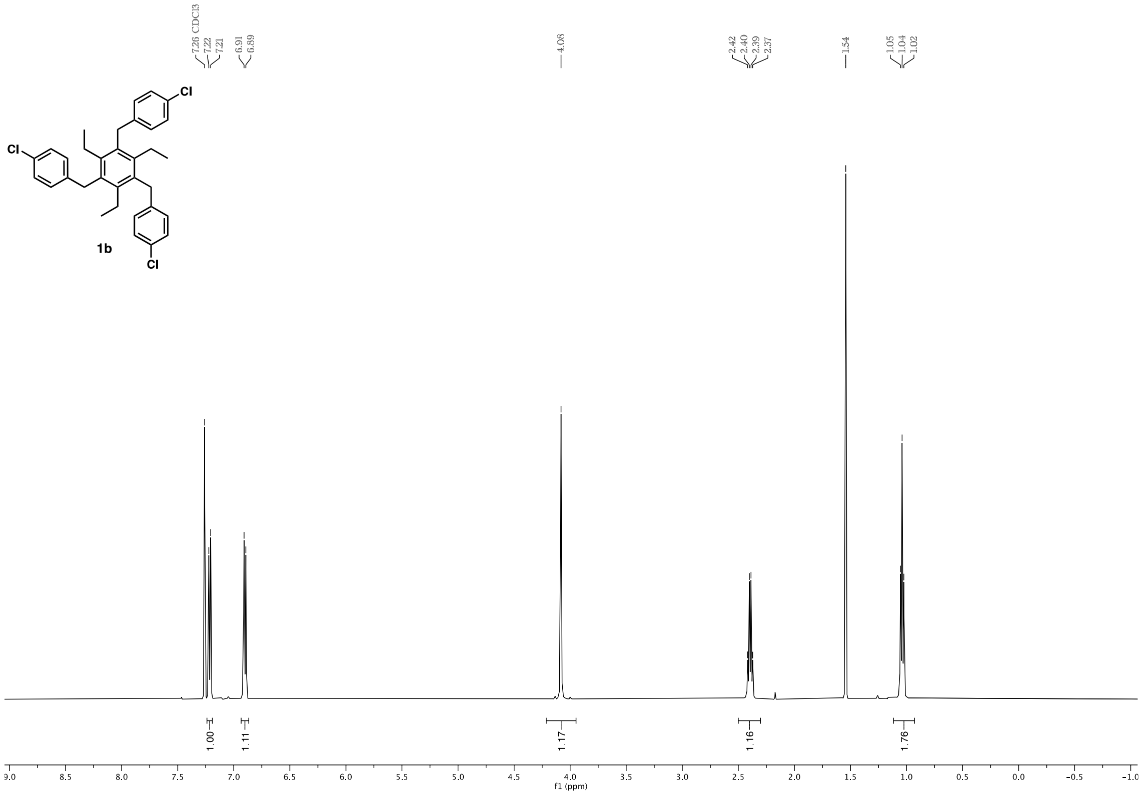

*Figure S9: ^{1}H-NMR spectrum of 4,4',4''-((2,4,6-triethylbenzene-1,3,5-triyl)tris(methylene))tris(chlorobenzene) (**1b**), (500 MHz, $CDCl_3$)*

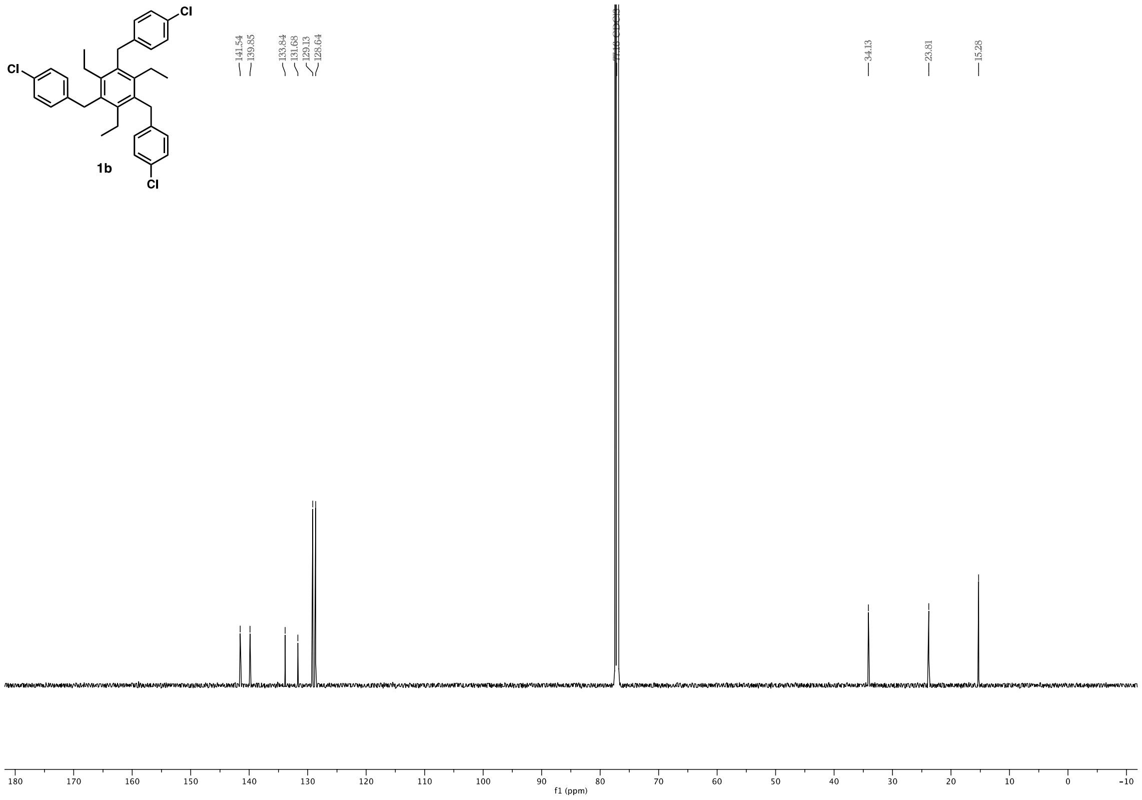

*Figure S10: ^{13}C{^{1}H}-NMR spectrum of 4,4',4''-((2,4,6-triethylbenzene-1,3,5-triyl)tris(methylene))tris(chlorobenzene) (**1b**), (126 MHz, $CDCl_3$).*

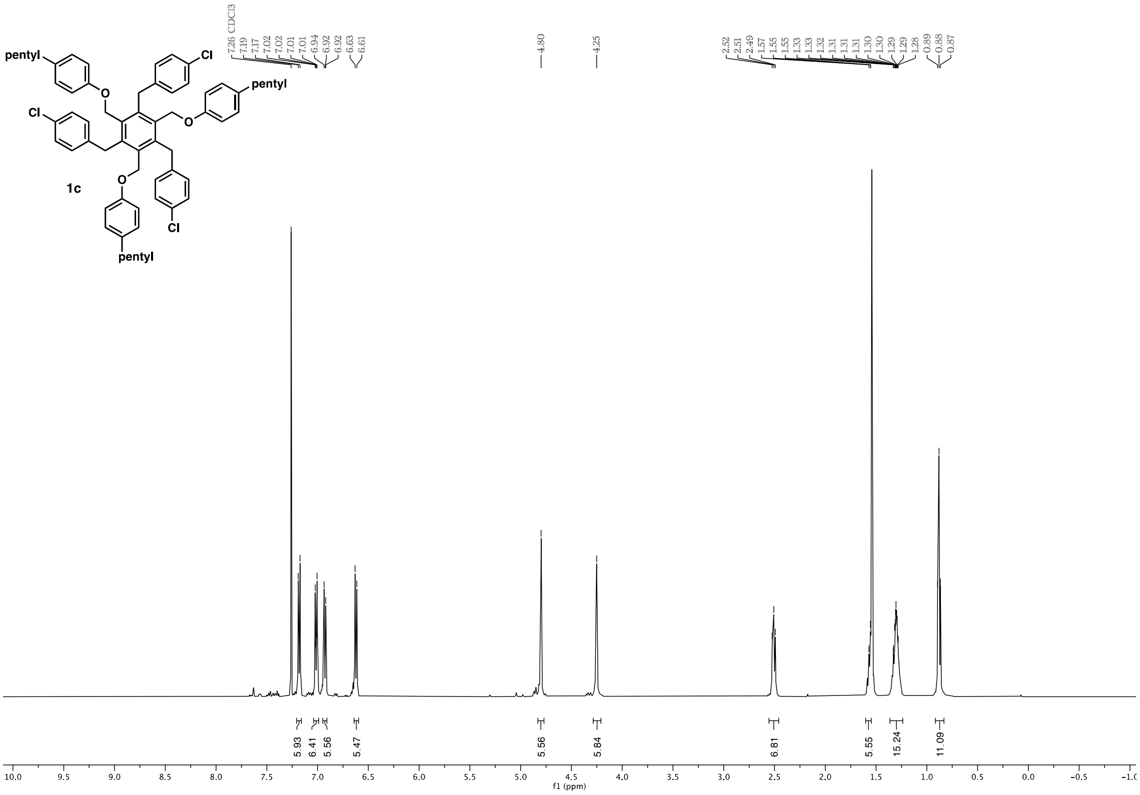

*Figure S11: ^{1}H-NMR spectrum of 4,4',4''-((2,4,6-tris((4-pentylphenoxy)methyl)benzene-1,3,5-triyl)tris(methylene))tris(chlorobenzene) (**1c**), (500 MHz, $CDCl_3$).*

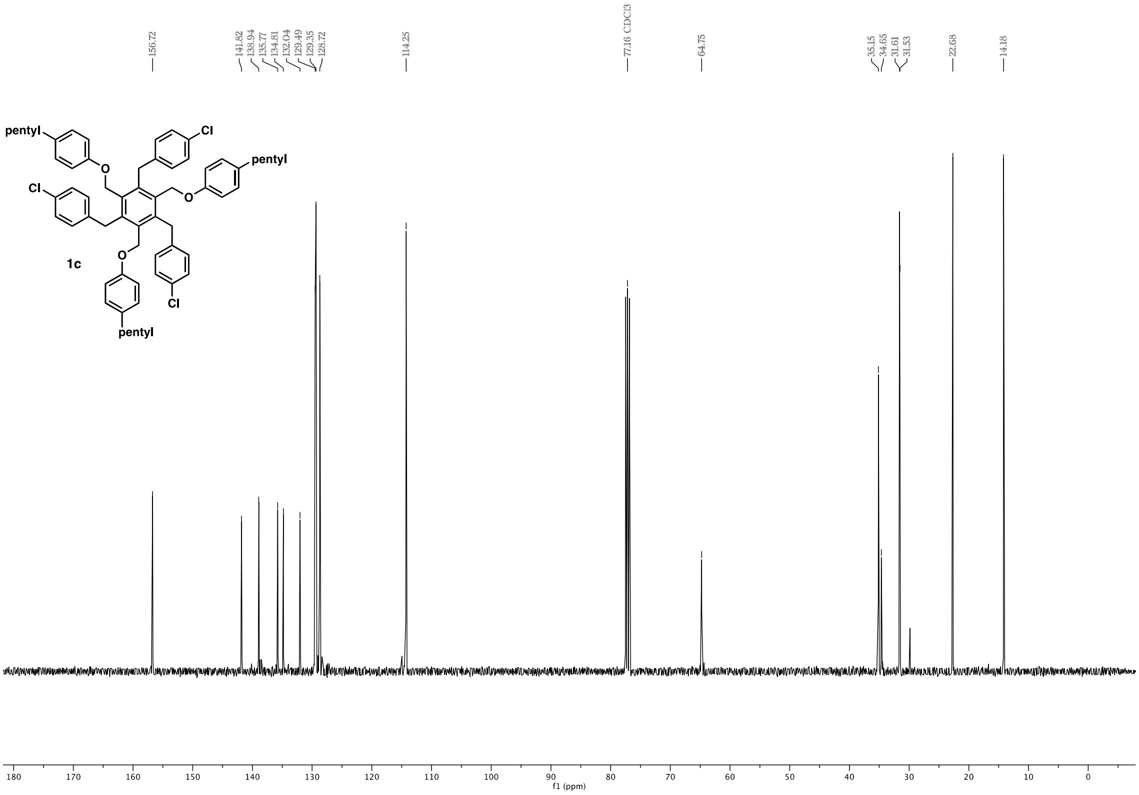

*Figure S12: $^{13}C\{^{1}H\}$-NMR spectrum of 4,4',4''-((2,4,6-tris((4-pentylphenoxy)methyl)benzene-1,3,5-triyl)tris(methylene))tris(chlorobenzene) (**1c**), (101 MHz, $CDCl_3$).*

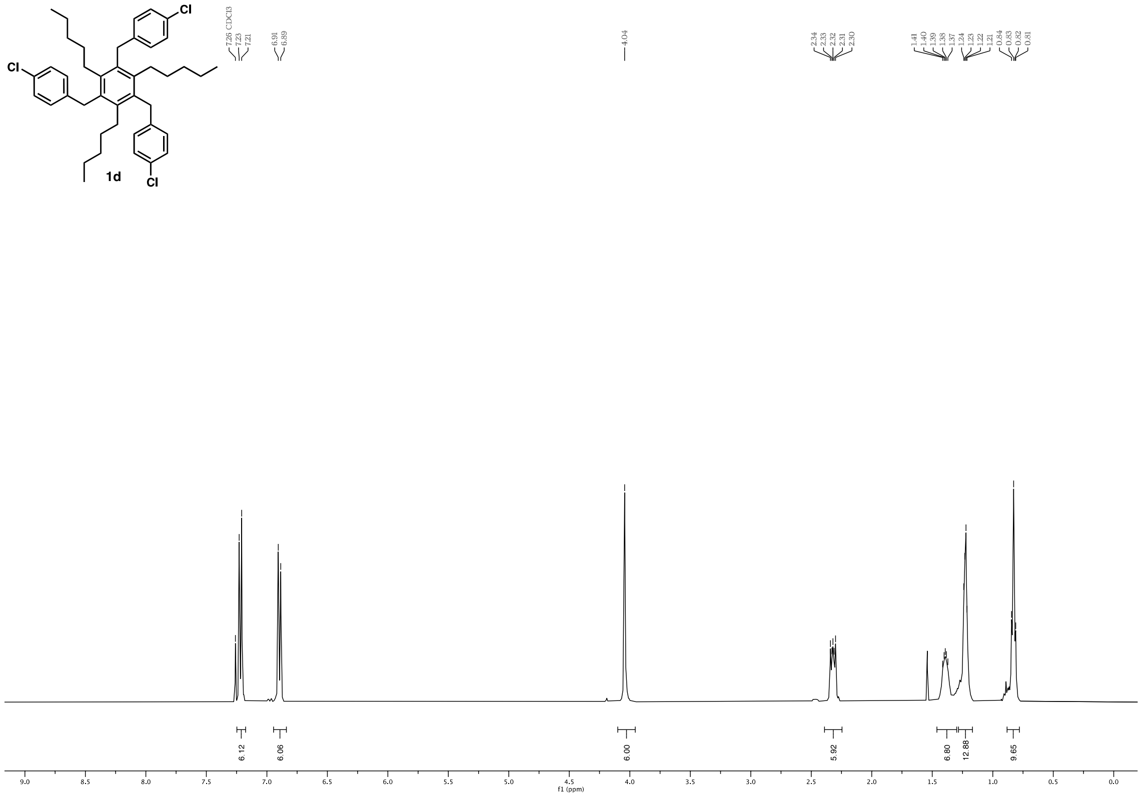

*Figure S13: ^{1}H-NMR spectrum of 4,4',4''-((2,4,6-tripentylbenzene-1,3,5-triyl)tris(methylene))tris(chlorobenzene) (**1d**), (400 MHz, $CDCl_3$).*

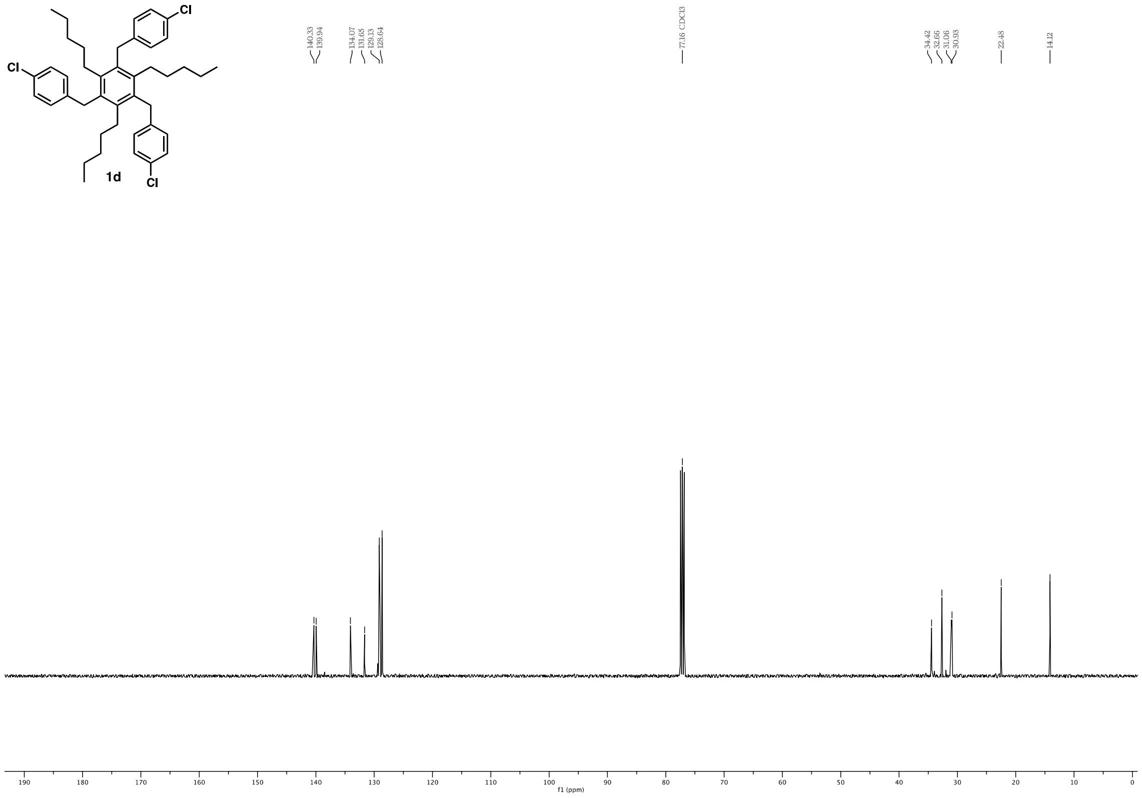

*Figure S14: $^{13}C\{^1H\}$-NMR spectrum of 4,4',4''-((2,4,6-tripentylbenzene-1,3,5-triyl)tris(methylene))tris(chlorobenzene) (**1d**), (101 MHz, $CDCl_3$).*

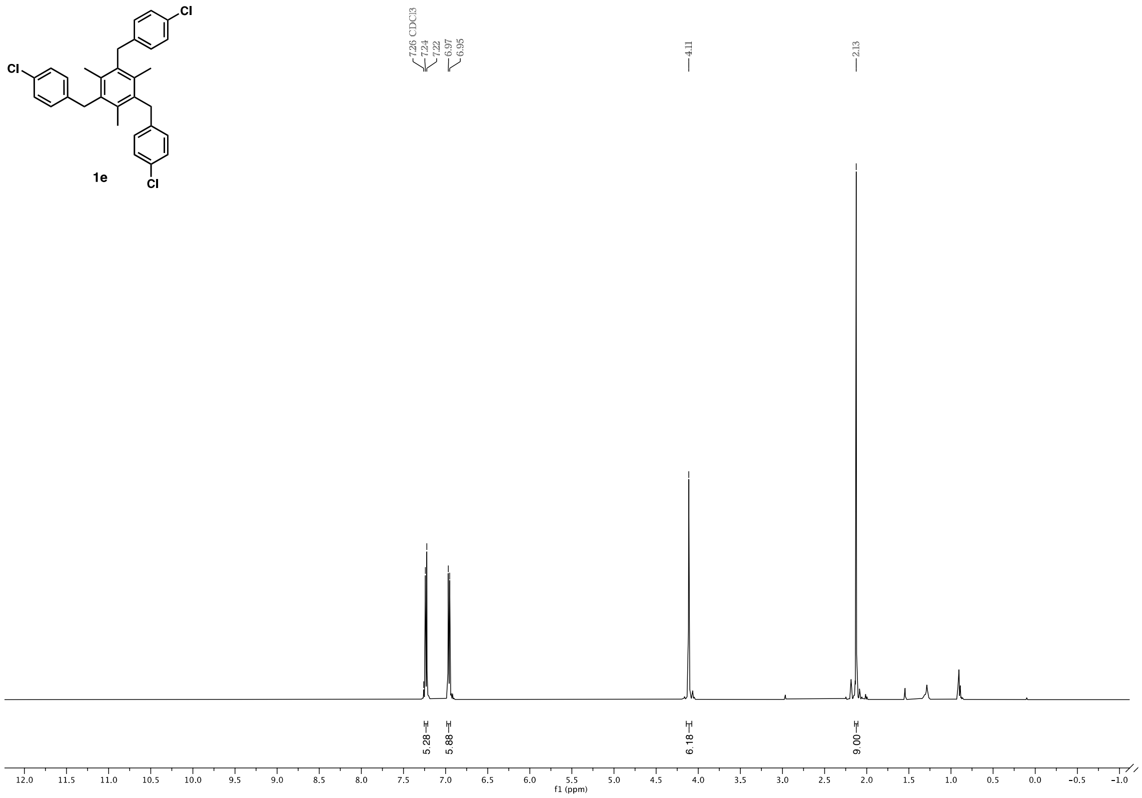

*Figure S15: ¹H-NMR spectrum of 4,4',4''-((2,4,6-trimethylbenzene-1,3,5-triyl)tris(methylene))tris(chlorobenzene) (**1e**), (500 MHz, $CDCl_3$).*

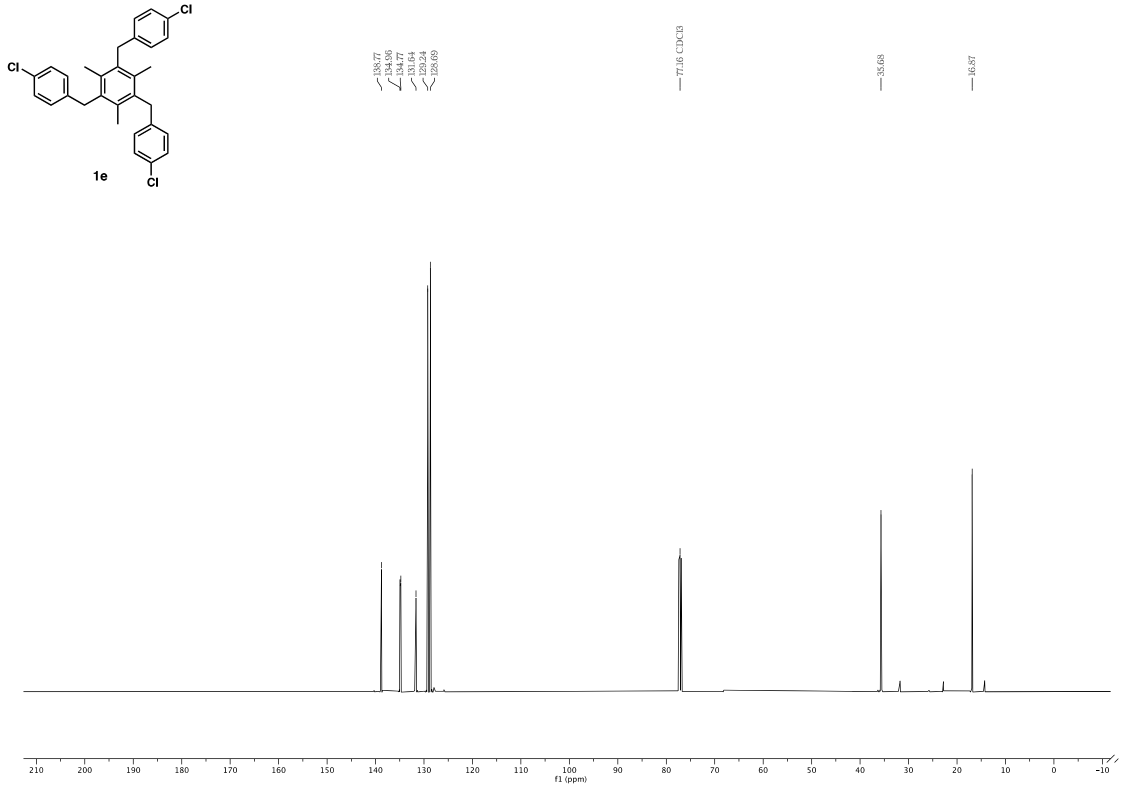

*Figure S16: $^{13}C\{^{1}H\}$-NMR spectrum of 4,4',4''-((2,4,6-trimethylbenzene-1,3,5-triyl)tris(methylene))tris(chlorobenzene) (**1e**), (126 MHz, $CDCl_3$).*

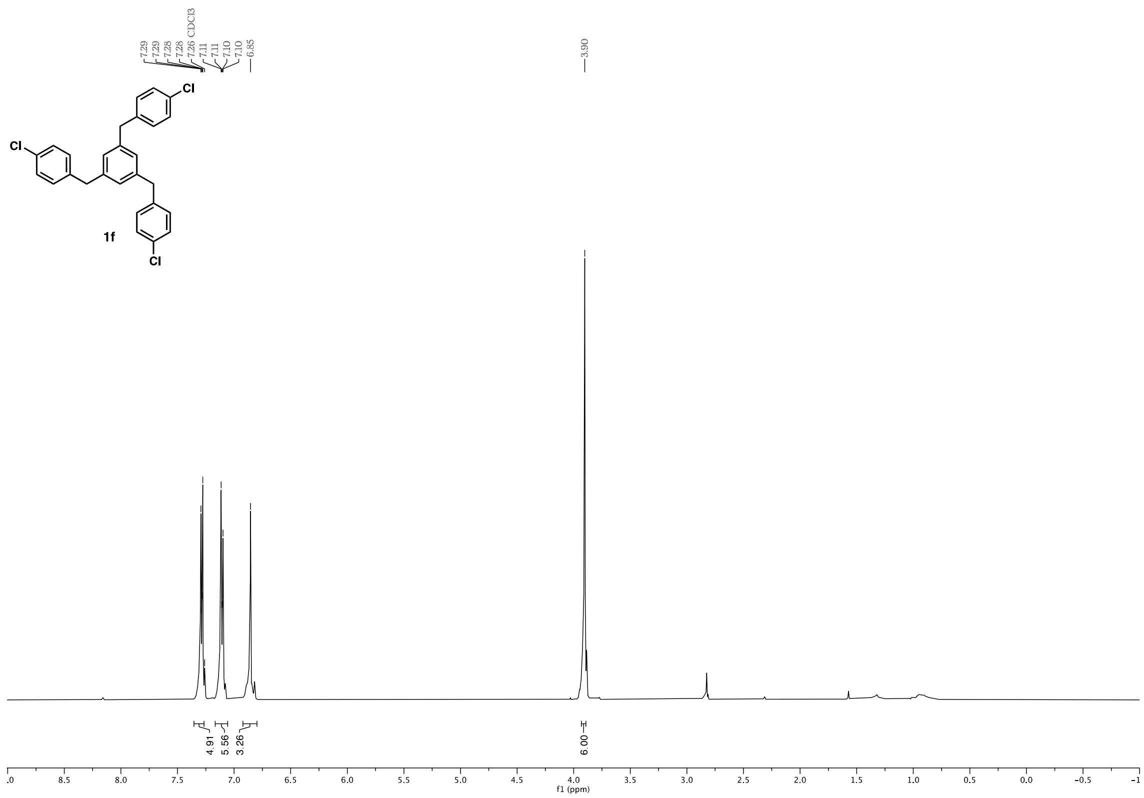

*Figure S17: ^{1}H-NMR spectrum of 1,3,5-tris(4-Chlorobenzyl)benzene (**1f**), (500 MHz, $CDCl_3$).*

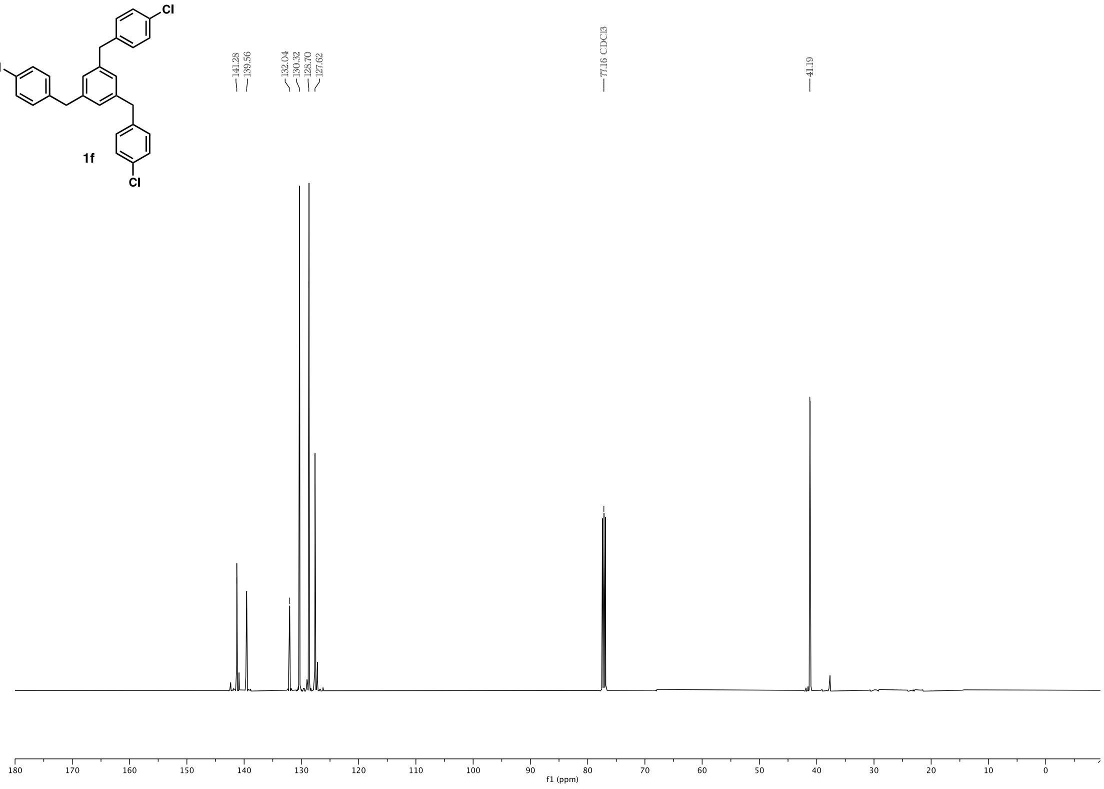

*Figure S18: ^{13}C{^{1}H}-NMR spectrum of 1,3,5-tris(4-Chlorobenzyl)benzene (**1f**) , (126 MHz, $CDCl_3$).*

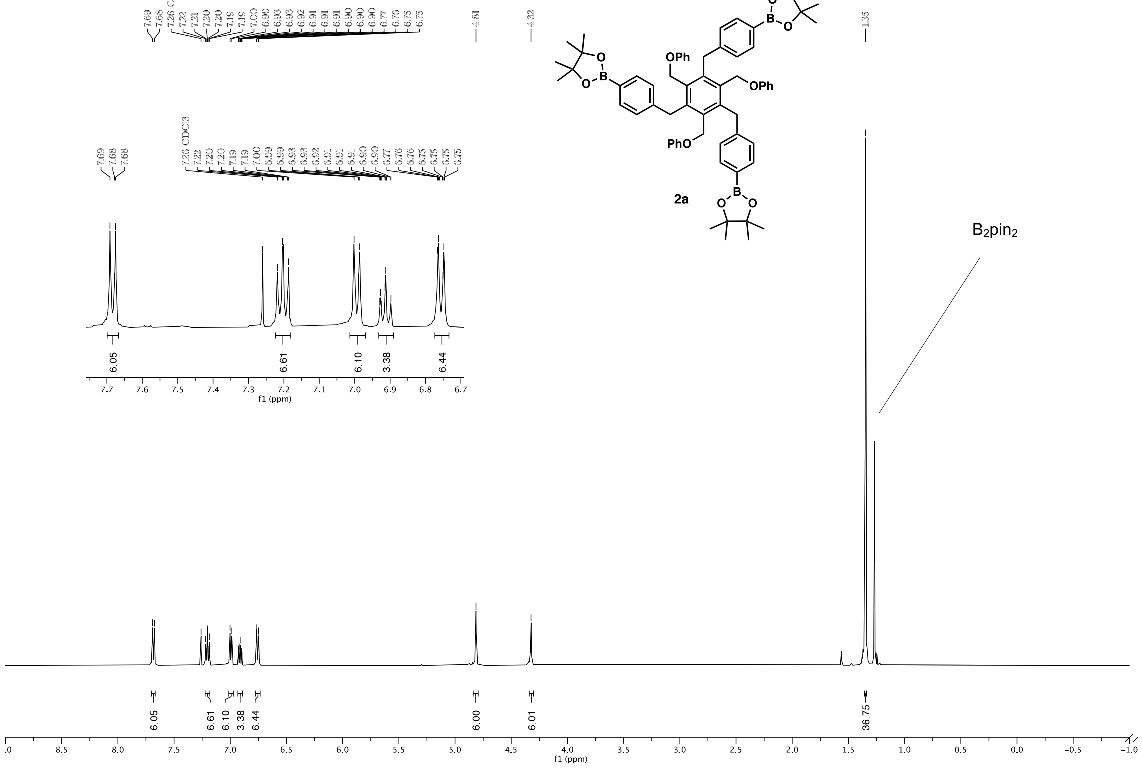

*Figure S19: ^{1}H-NMR spectrum of 2,2',2''-(((2,4,6-Tris(phenoxymethyl)benzene-1,3,5-triyl)tris(methylene))tris(benzene-4,1-diyl))tris(4,4,5,5-tetramethyl-1,3,2-dioxaborolane) (**2a**), (500 MHz, $CDCl_3$).*

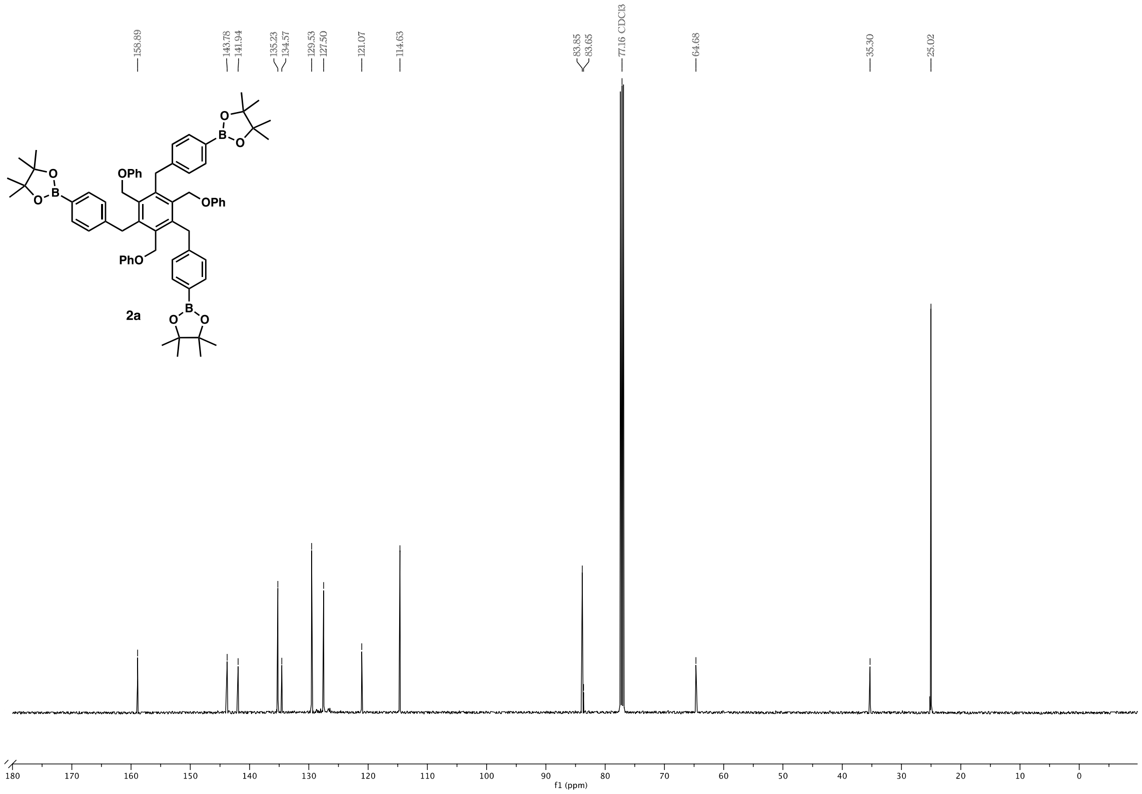

*Figure S20: $^{13}C\{^{1}H\}$-NMR spectrum of 2,2',2''-(((2,4,6-Tris(phenoxymethyl)benzene-1,3,5-triyl)tris(methylene))tris(benzene-4,1-diyl))tris(4,4,5,5-tetramethyl-1,3,2-dioxaborolane) (**2a**), (126 MHz, $CDCl_3$).*

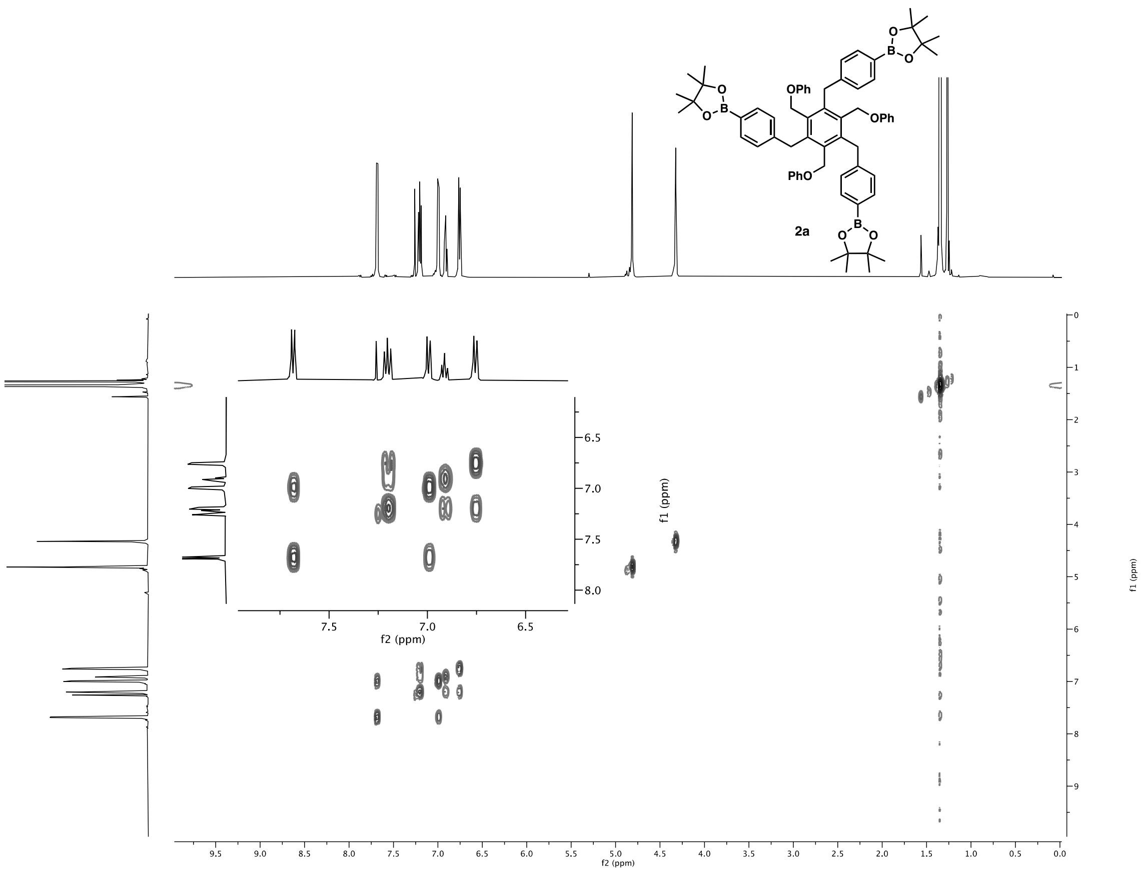

*Figure S21: COSY-NMR spectrum of 2,2',2''-(((2,4,6-Tris(phenoxymethyl)benzene-1,3,5-triyl)tris(methylene))tris(benzene-4,1-diyl))tris(4,4,5,5-tetramethyl-1,3,2-dioxaborolane) (**2a**), (500 MHz, $CDCl_3$).*

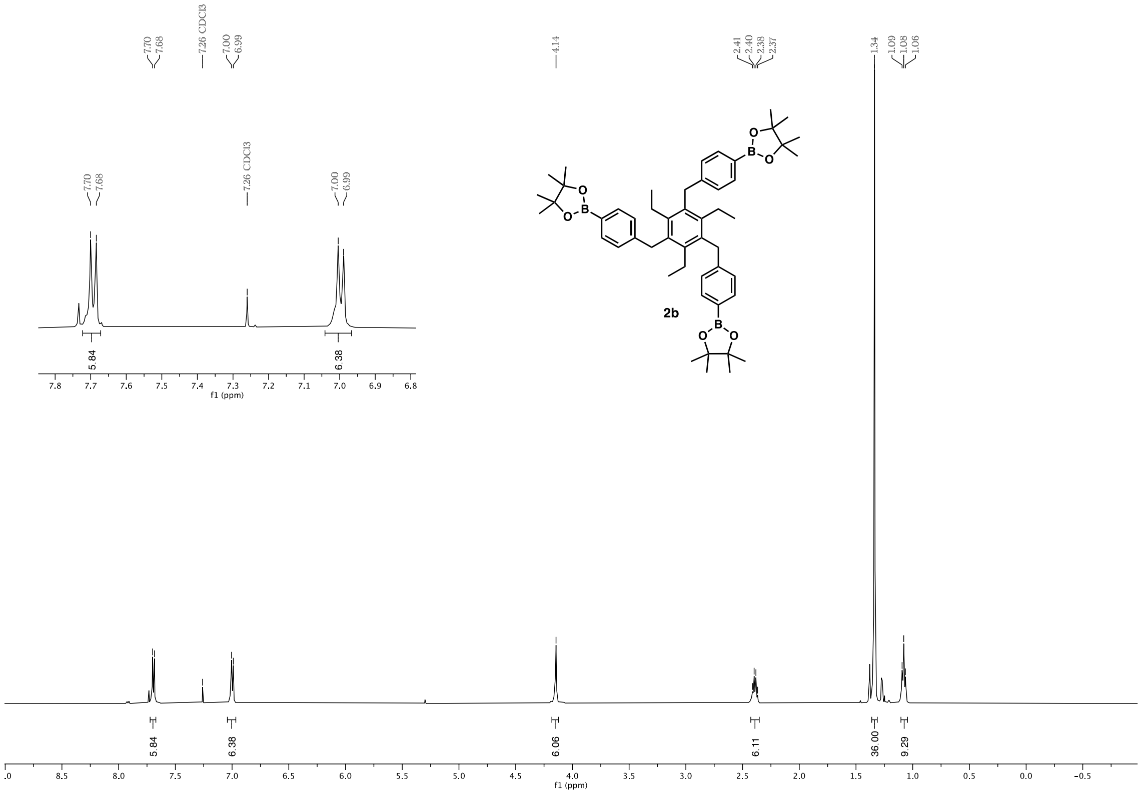

*Figure S22: ¹H-NMR spectrum of 2,2',2''-(((2,4,6-Triethylbenzene-1,3,5-triyl)tris(methylene))tris(benzene-4,1-diyl))tris(4,4,5,5-tetramethyl-1,3,2-dioxaborolane) (**2b**), (500 MHz, $CDCl_3$).*

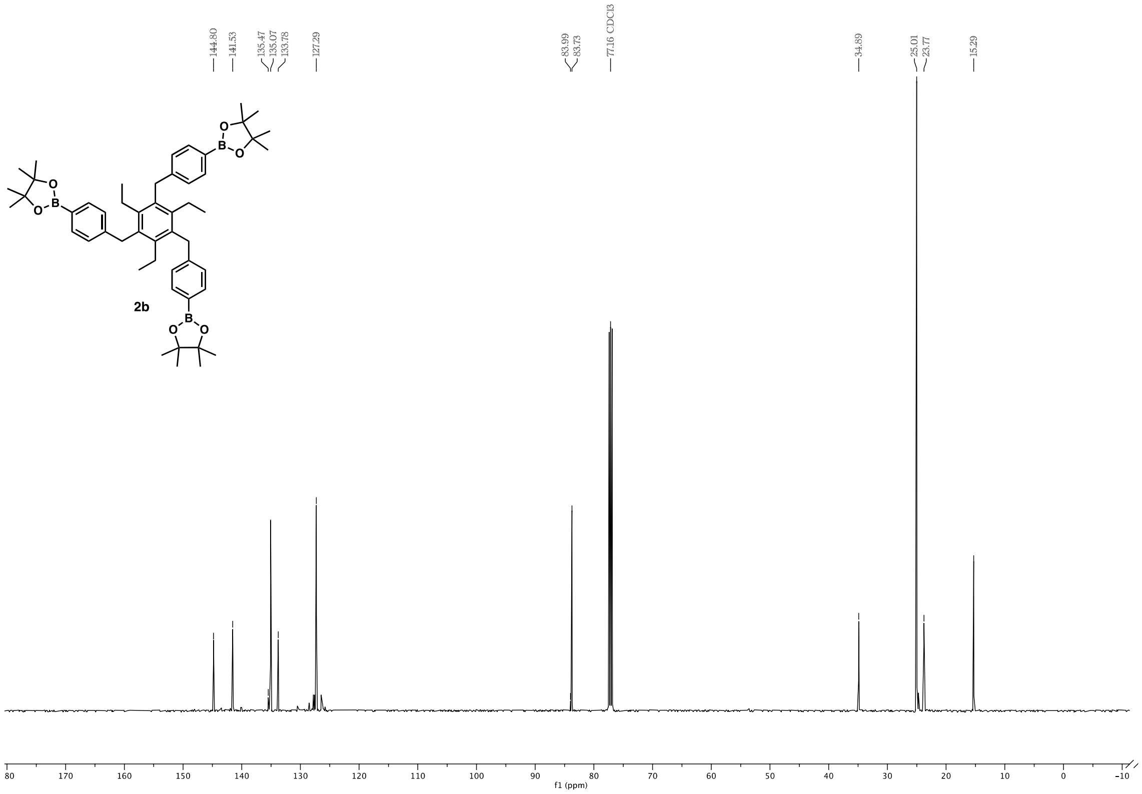

*Figure S23: $^{13}C\{^{1}H\}$-NMR spectrum of 2,2',2''-(((2,4,6-Triethylbenzene-1,3,5-triyl)tris(methylene))tris(benzene-4,1-diyl))tris(4,4,5,5-tetramethyl-1,3,2-dioxaborolane) (**2b**), (126 MHz, $CDCl_3$).*

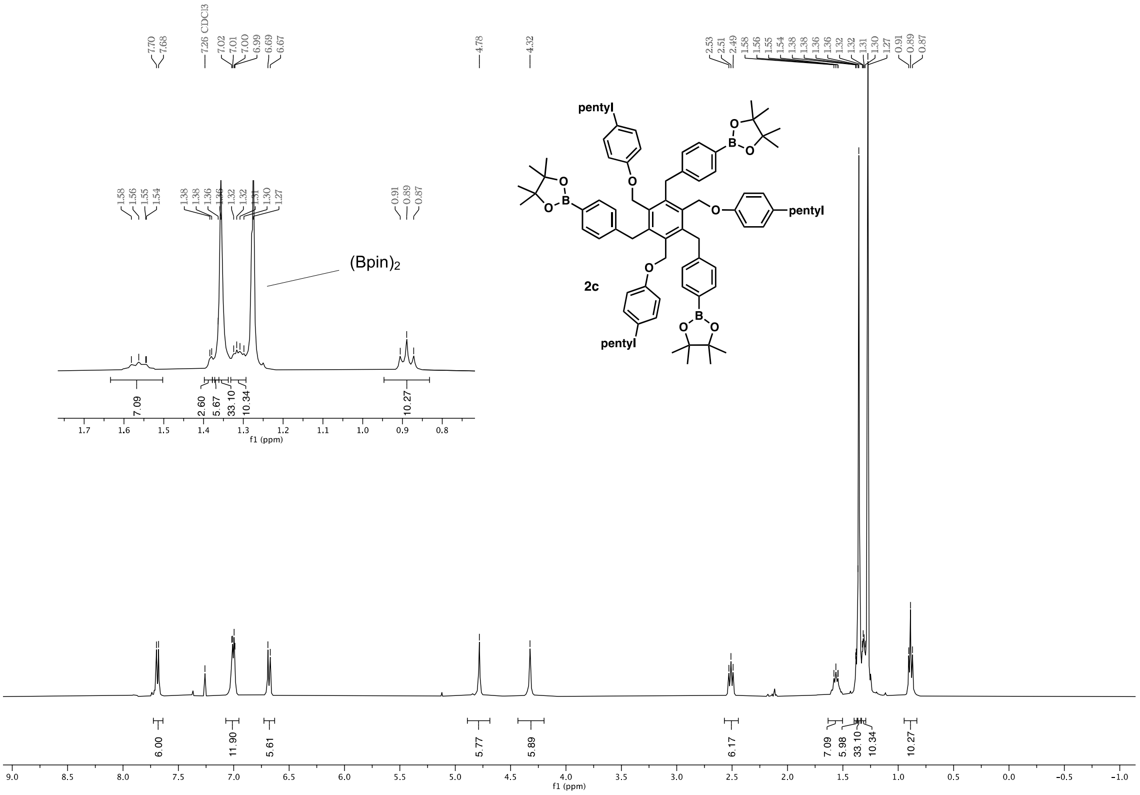

*Figure S24: ^{1}H-NMR spectrum of 2,2',2''-(((2,4,6-tris(phenoxymethyl)benzene-1,3,5-triyl)tris(methylene))tris(benzene-4,1-diyl))tris(4,4,5,5-tetramethyl-1,3,2-dioxaborolane) (**2c**), (400 MHz, $CDCl_3$).*

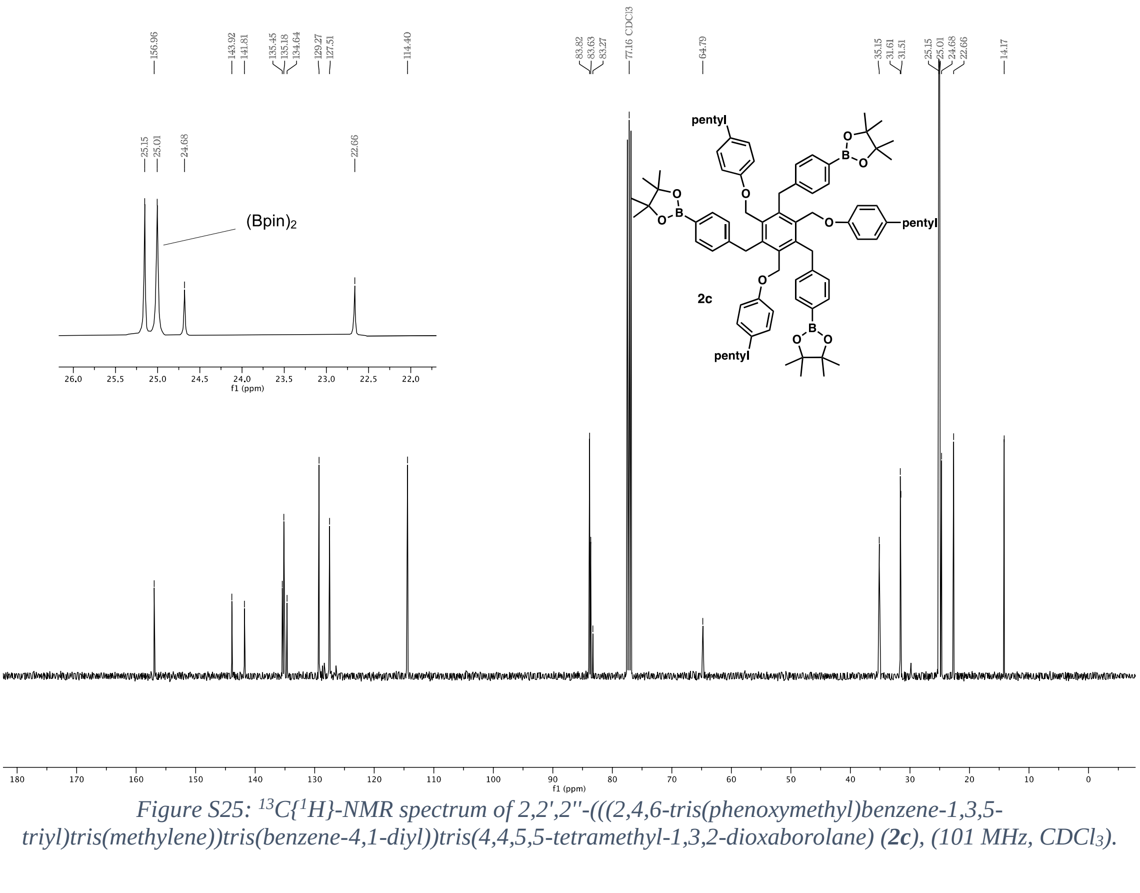

*Figure S25: $^{13}C\{^{1}H\}$-NMR spectrum of 2,2',2''-(((2,4,6-tris(phenoxymethyl)benzene-1,3,5-triyl)tris(methylene))tris(benzene-4,1-diyl))tris(4,4,5,5-tetramethyl-1,3,2-dioxaborolane) (**2c**), (101 MHz, $CDCl_3$).*

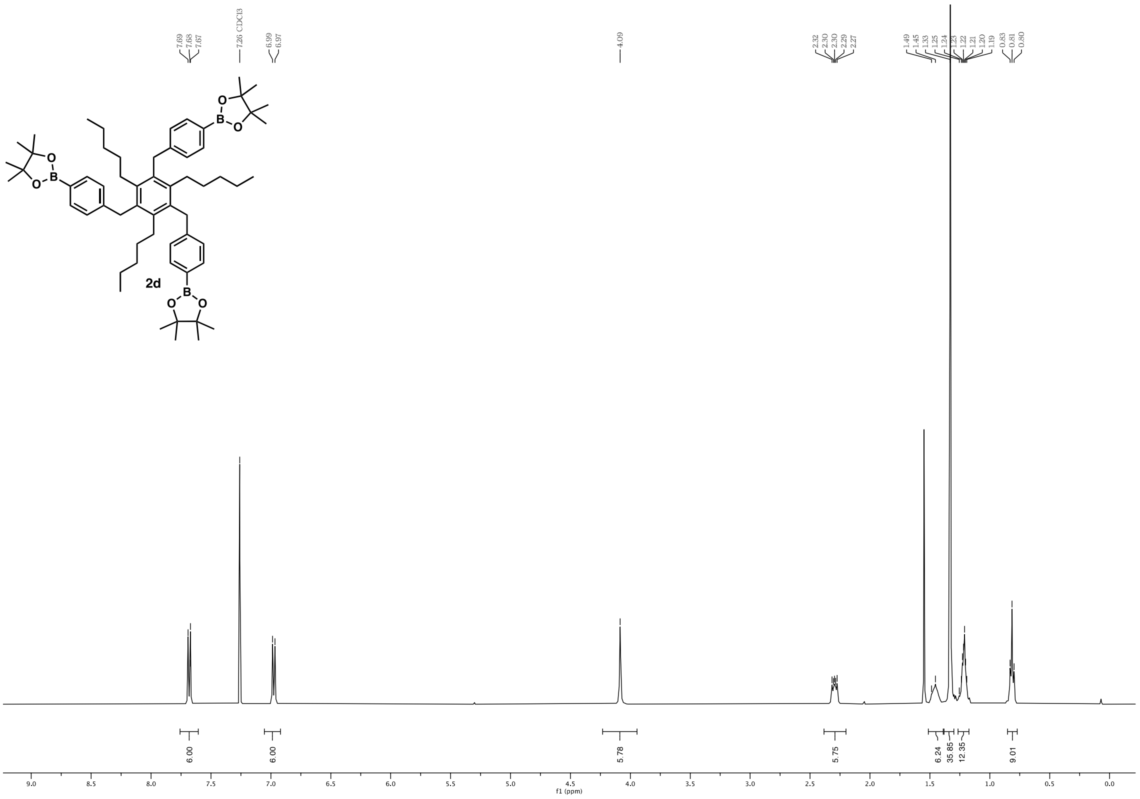

*Figure S26: ¹H-NMR spectrum of 2,2',2''-(((2,4,6-tripentylbenzene-1,3,5-triyl)tris(methylene))tris(benzene-4,1-diyl))tris(4,4,5,5-tetramethyl-1,3,2-dioxaborolane) (**2d**), (400 MHz, $CDCl_3$).*

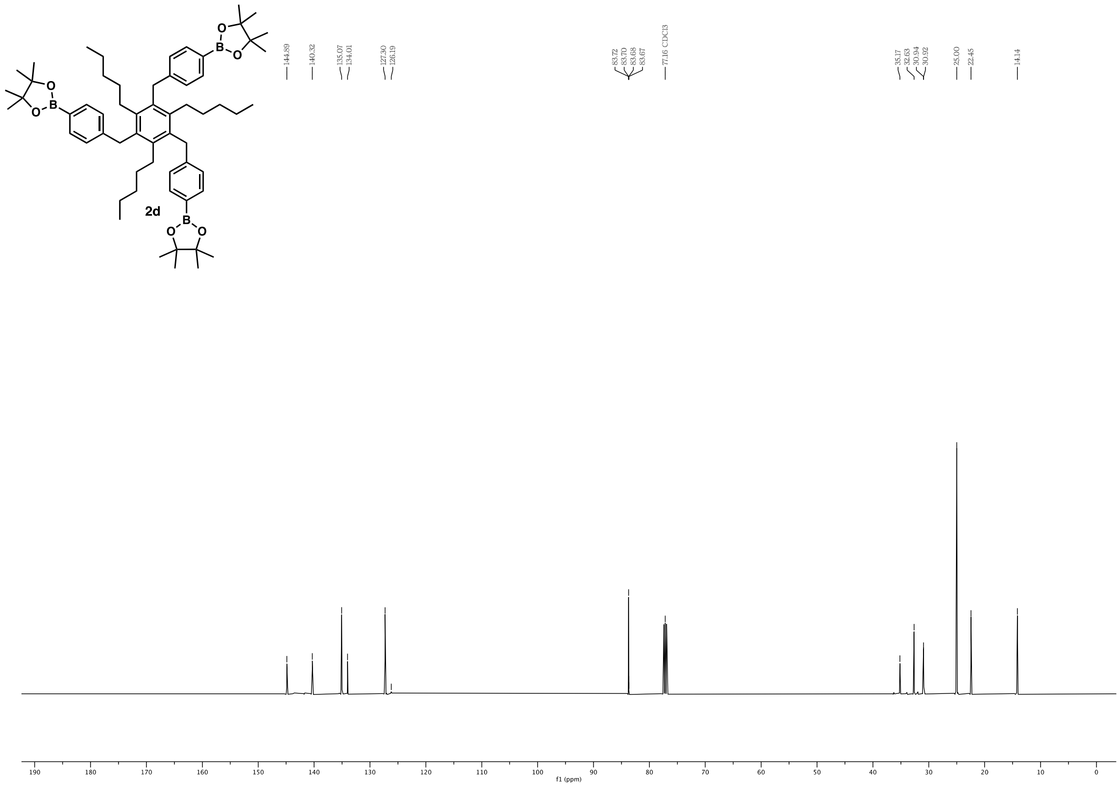

*Figure S27: ¹³C{¹H}-NMR spectrum of 2,2',2''-(((2,4,6-tripentylbenzene-1,3,5-triyl)tris(methylene))tris(benzene-4,1-diyl))tris(4,4,5,5-tetramethyl-1,3,2-dioxaborolane) (**2d**), (126 MHz, $CDCl_3$).*

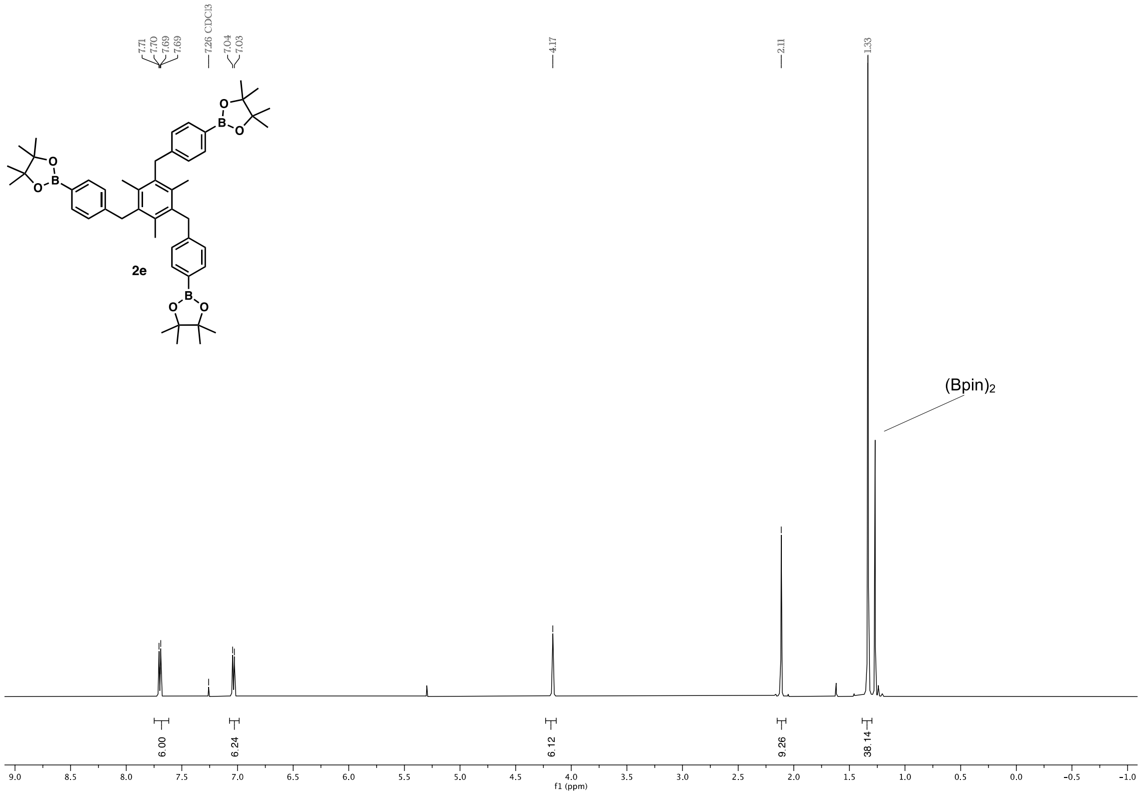

*Figure S28: ^{1}H-NMR spectrum of 2,2',2''-(((2,4,6-trimethylbenzene-1,3,5-triyl)tris(methylene))tris(benzene-4,1-diyl))tris(4,4,5,5-tetramethyl-1,3,2-dioxaborolane) (**2e**), (500 MHz, CDCl$_3$).*

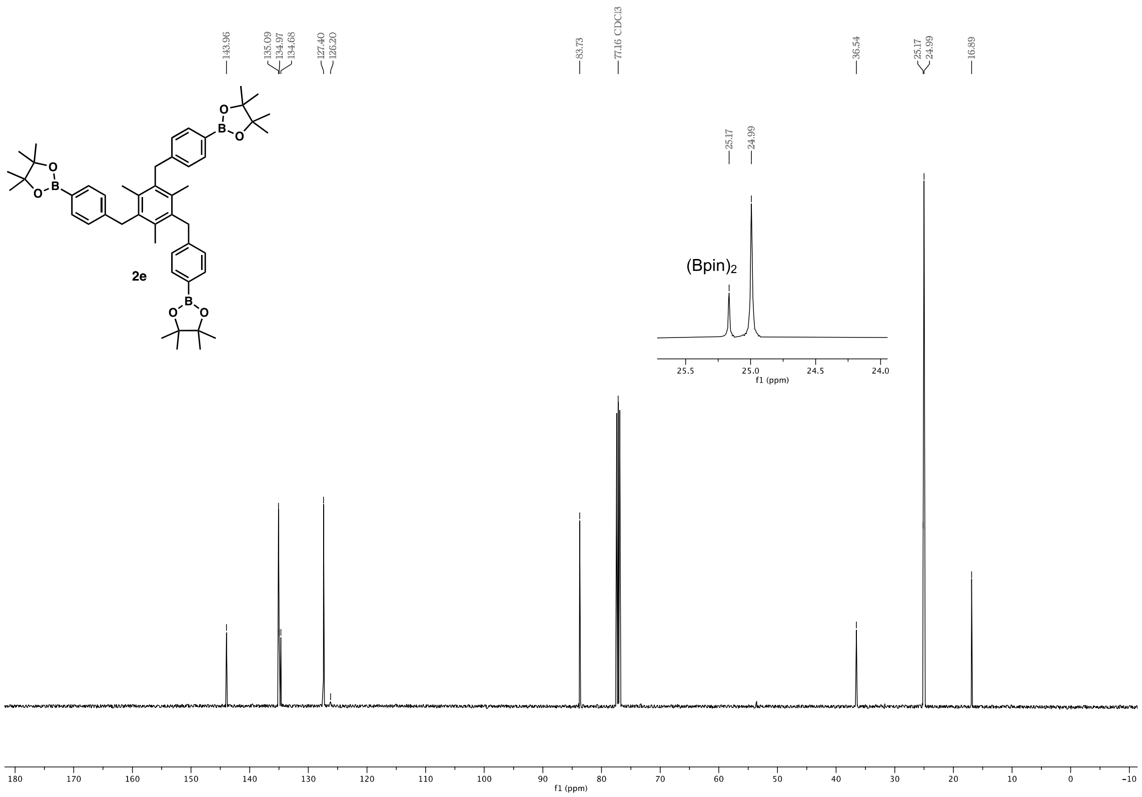

*Figure S29: ^{13}C{^{1}H}-NMR spectrum of 2,2',2''-(((2,4,6-trimethylbenzene-1,3,5-triyl)tris(methylene))tris(benzene-4,1-diyl))tris(4,4,5,5-tetramethyl-1,3,2-dioxaborolane) (**2e**), (126 MHz, CDCl$_3$).*

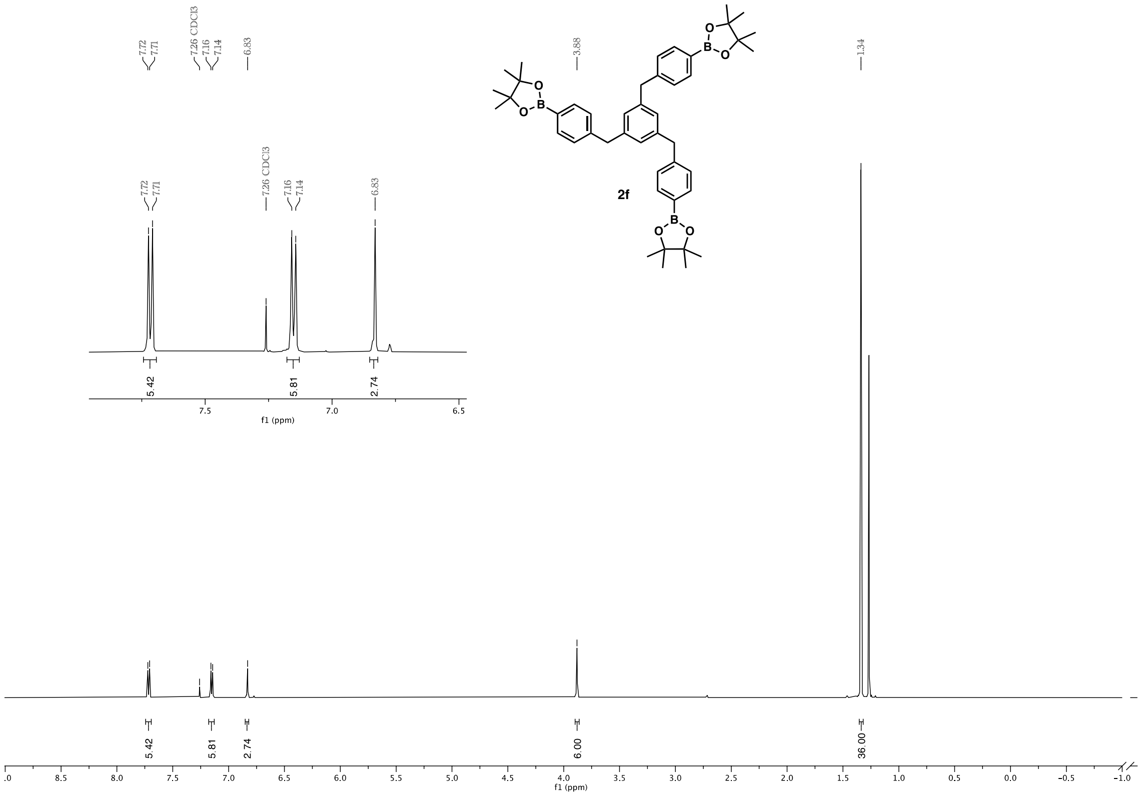

*Figure S30: ^{1}H-NMR spectrum of 1,3,5-Tris(4-(4,4,5,5-tetramethyl-1,3,2-dioxaborolan-2-yl)benzyl)benzene (**2f**), (500 MHz, $CDCl_3$).*

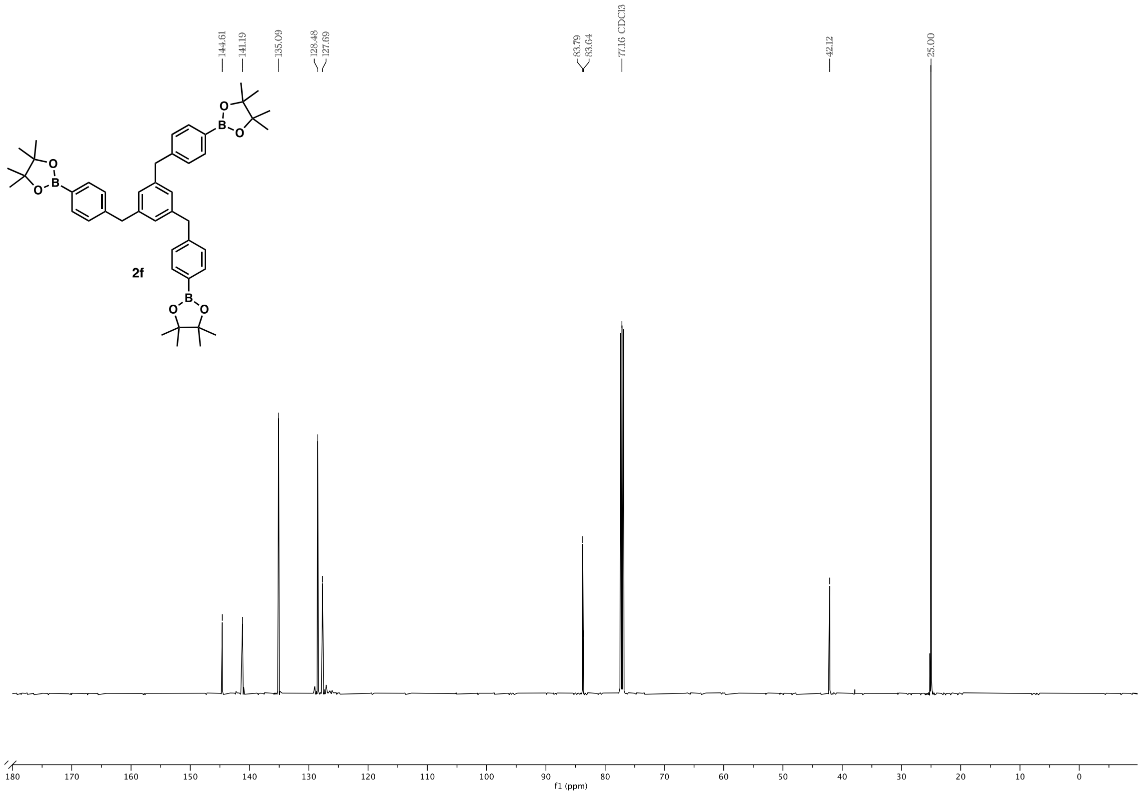

*Figure S31: $^{13}C\{^{1}H\}$-NMR spectrum of 1,3,5-Tris(4-(4,4,5,5-tetramethyl-1,3,2-dioxaborolan-2-yl)benzyl)benzene (**2f**), (126 MHz, $CDCl_3$).*

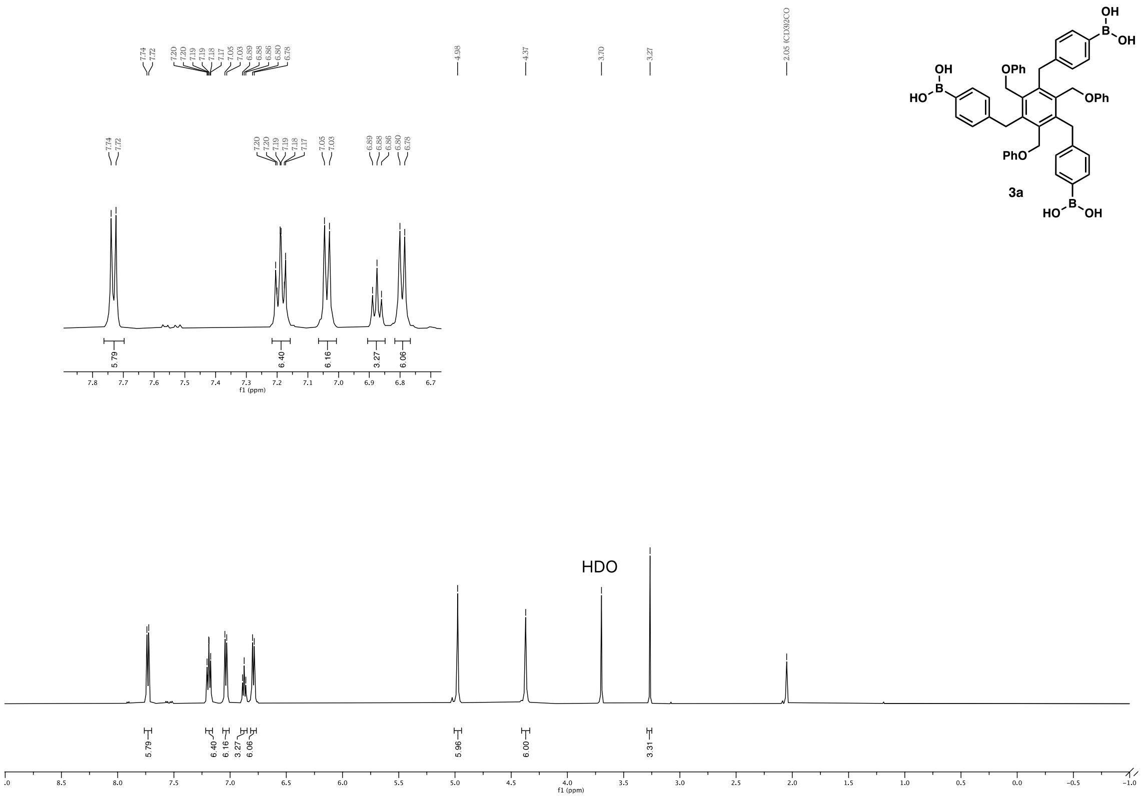

*Figure S32: ^{1}H-NMR spectrum of (((2,4,6-Tris(phenoxymethyl)benzene-1,3,5-triyl)tris(methylene))tris(benzene-4,1-diyl))triboronic acid (**3a**) in acetone-d$_6$ with few drops of D_2O (500 MHz)*

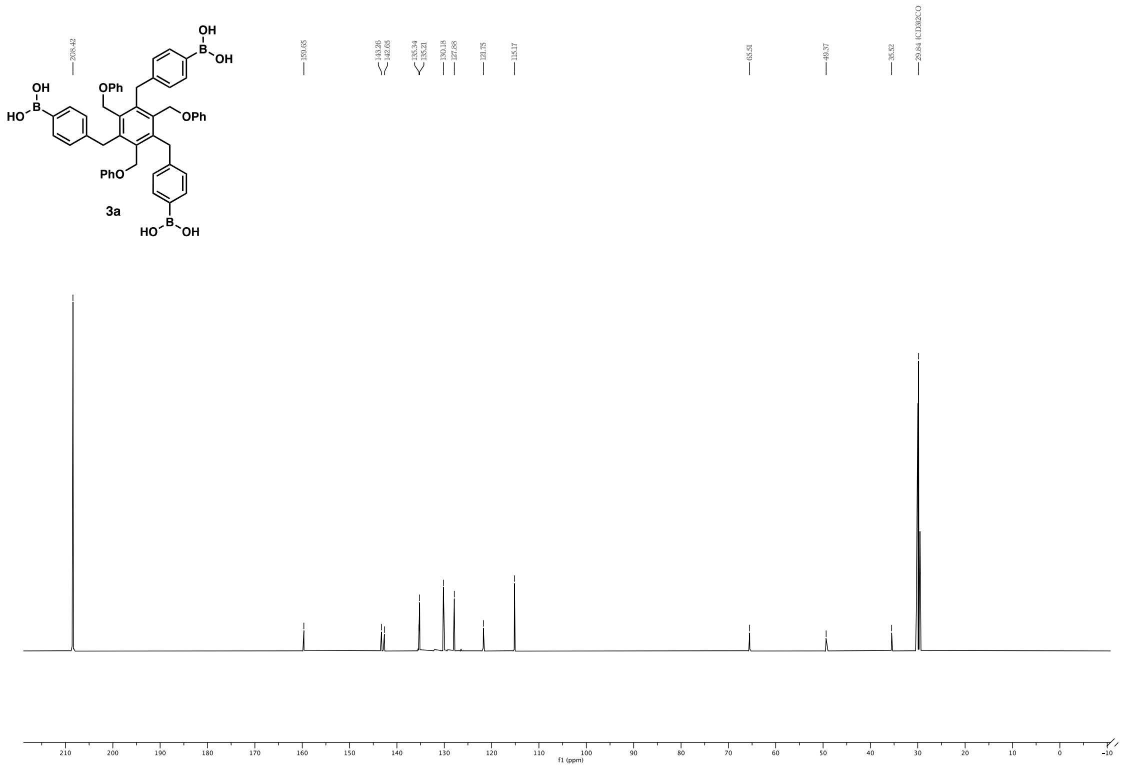

*Figure S33: ^{13}C{^{1}H}-NMR spectrum of (((2,4,6-Tris(phenoxymethyl)benzene-1,3,5-triyl)tris(methylene))tris(benzene-4,1-diyl))triboronic acid (**3a**) in acetone-d$_6$ with few drops of D_2O (126 MHz).*

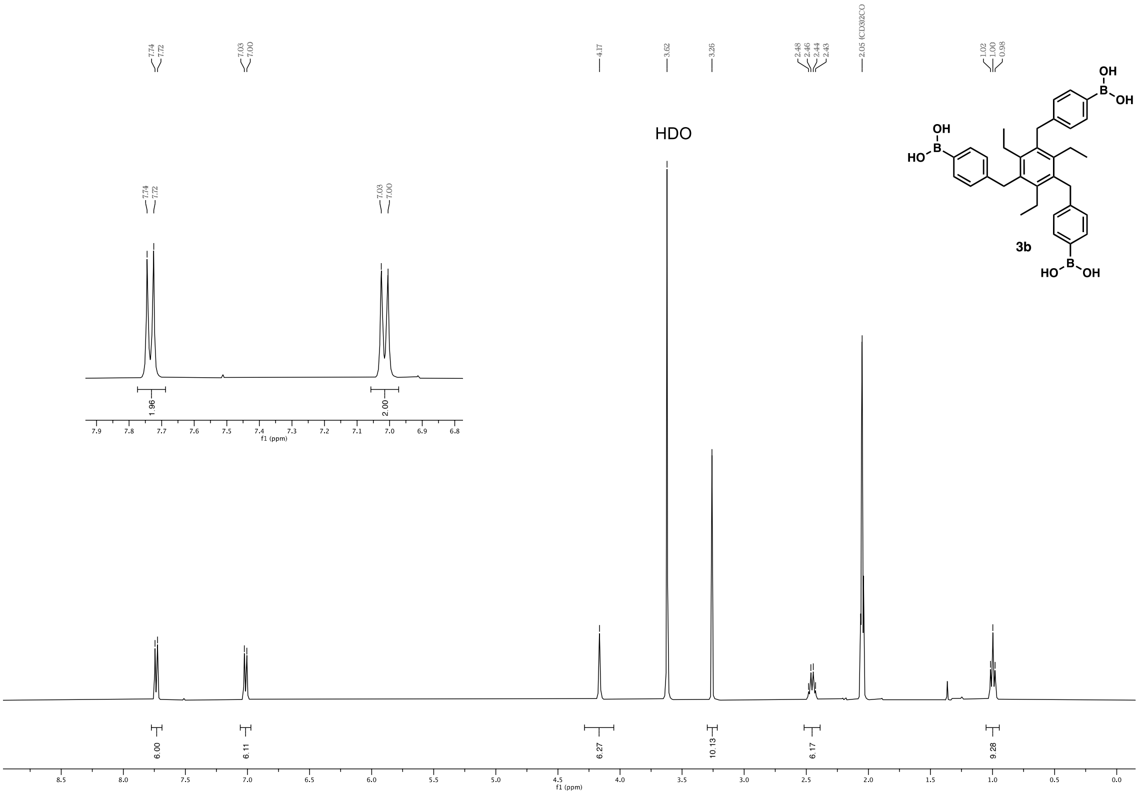

*Figure S34: ^{1}H-NMR spectrum of (((2,4,6-Triethylbenzene-1,3,5-triyl)tris(methylene))tris(benzene-4,1-diyl))triboronic acid (**3b**) in acetone-d$_6$ with few drops of D_2O (400 MHz).*

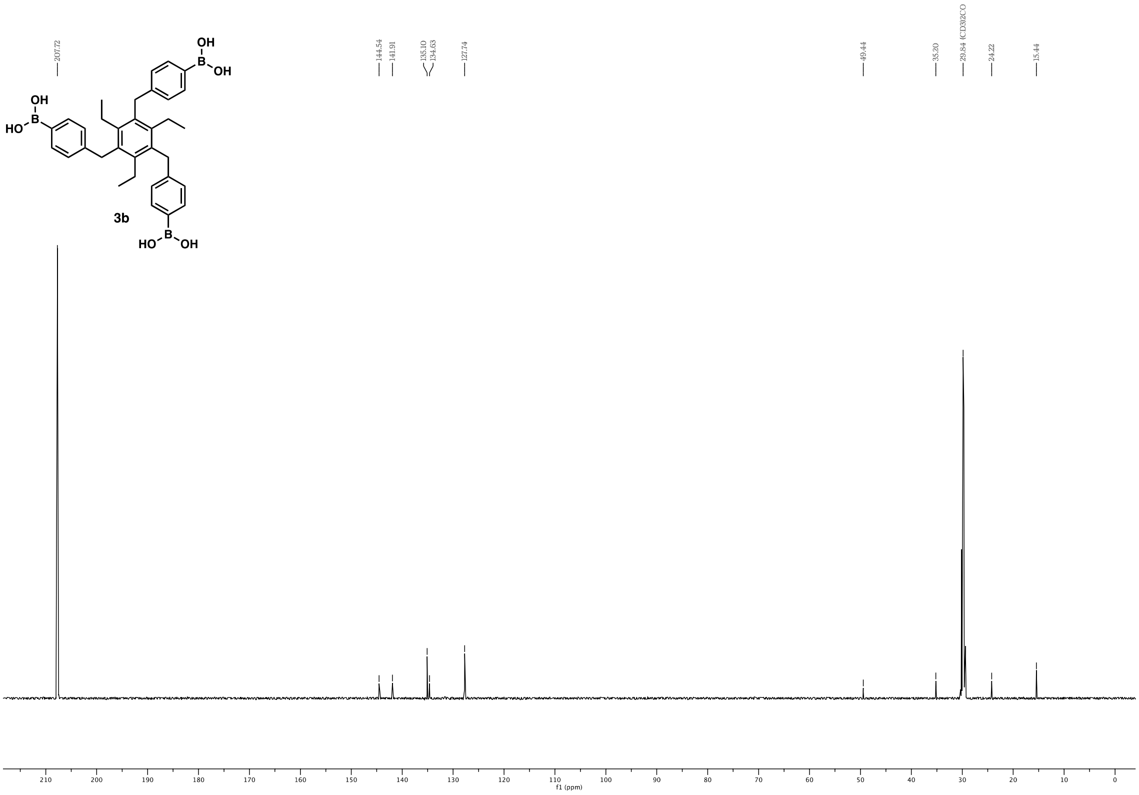

*Figure S35: $^{13}C\{^1H\}$-NMR spectrum of (((2,4,6-Triethylbenzene-1,3,5-triyl)tris(methylene))tris(benzene-4,1-diyl))triboronic acid (**3b**) in acetone-d$_6$ with few drops of D_2O (126 MHz).*

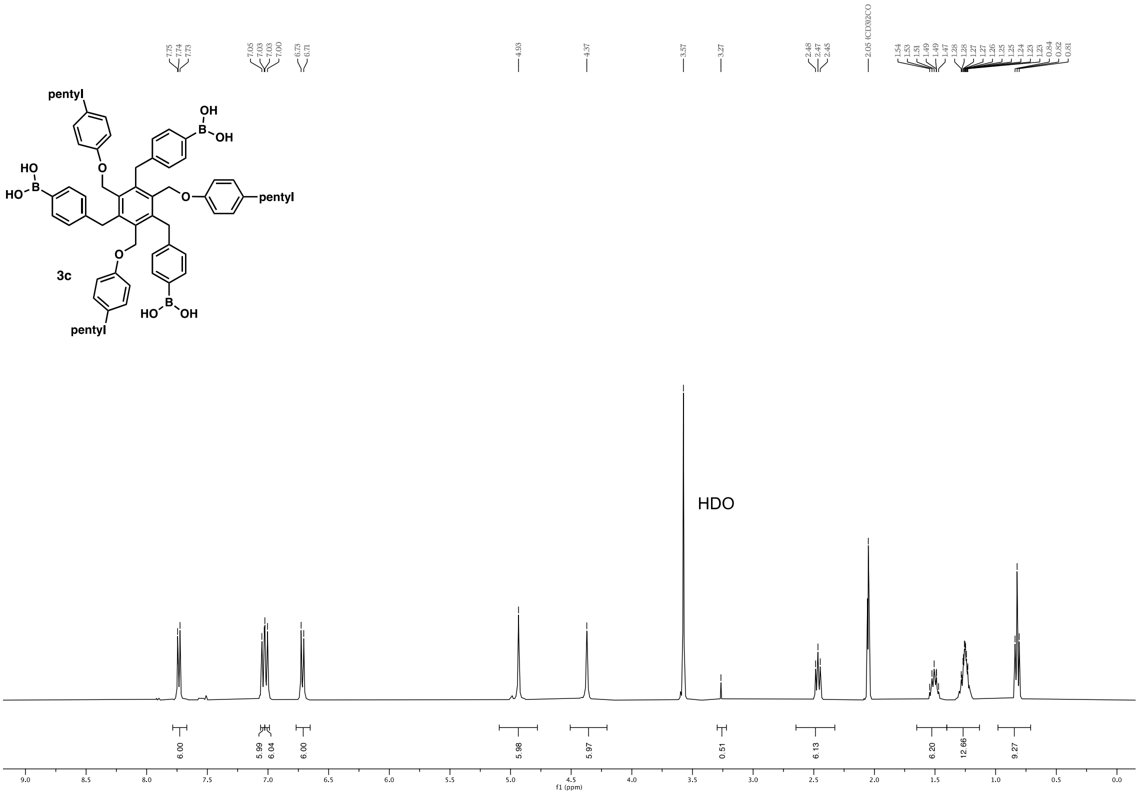

*Figure S36: ^{1}H-NMR spectrum of (((2,4,6-tris((4-pentylphenoxy)methyl)benzene-1,3,5-triyl)tris(methylene))tris(benzene-4,1-diyl))triboronic acid (**3c**) in acetone-d_6 with few drops of D_2O (400 MHz).*

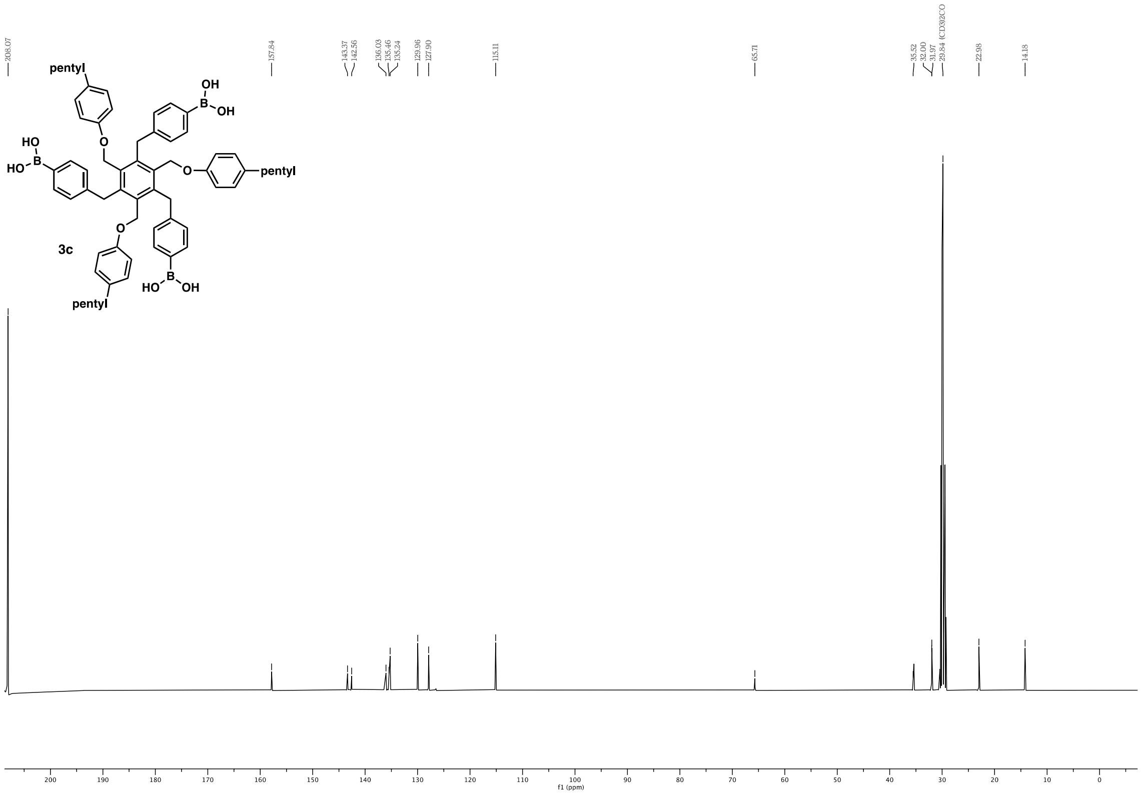

*Figure S37: ^{13}C{^{1}H}-NMR spectrum of (((2,4,6-tris((4-pentylphenoxy)methyl)benzene-1,3,5-triyl)tris(methylene))tris(benzene-4,1-diyl))triboronic acid (**3c**) in acetone-d_6 with few drops of D_2O (101 MHz).*

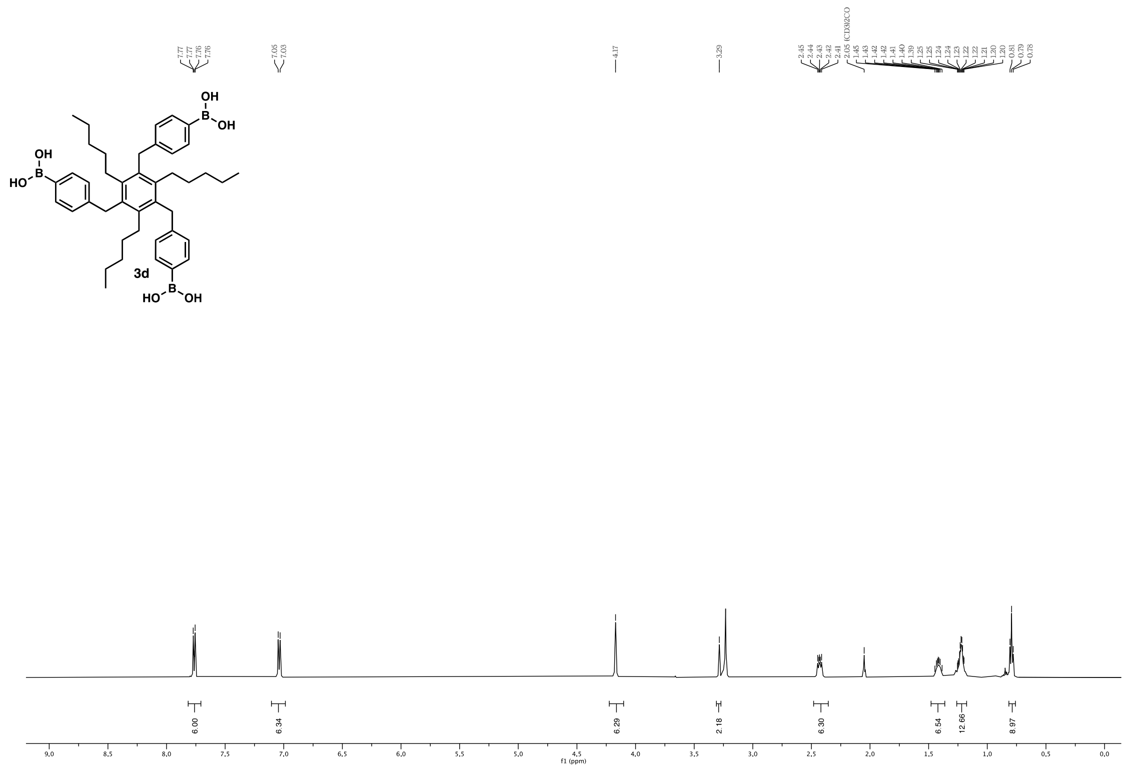

*Figure S38: ^{1}H-NMR spectrum of (((2,4,6-tripentylbenzene-1,3,5-triyl)tris(methylene))tris(benzene-4,1-diyl))triboronic acid (**3d**) in acetone-d$_6$ with few drops of D_2O (500 MHz).*

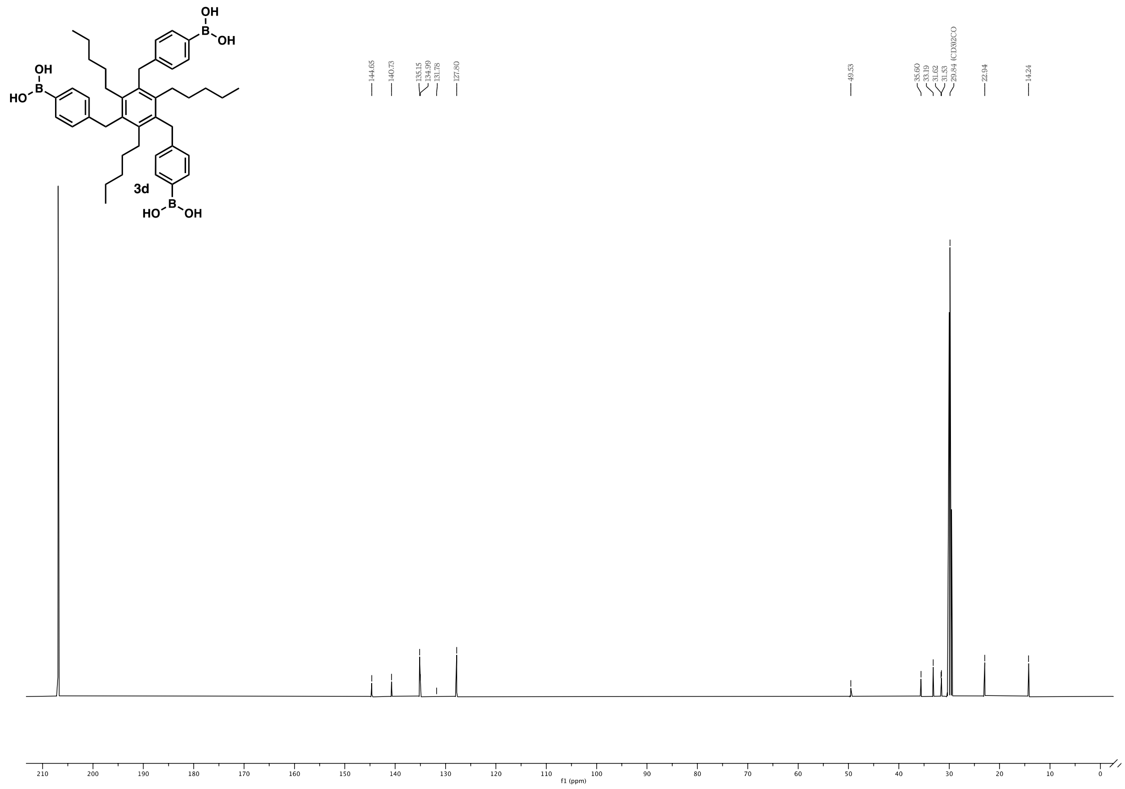

*Figure S39: ^{13}C{^{1}H}-NMR spectrum of (((2,4,6-tripentylbenzene-1,3,5-triyl)tris(methylene))tris(benzene-4,1-diyl))triboronic acid (**3d**) in acetone-d$_6$ with few drops of D_2O (126 MHz).*

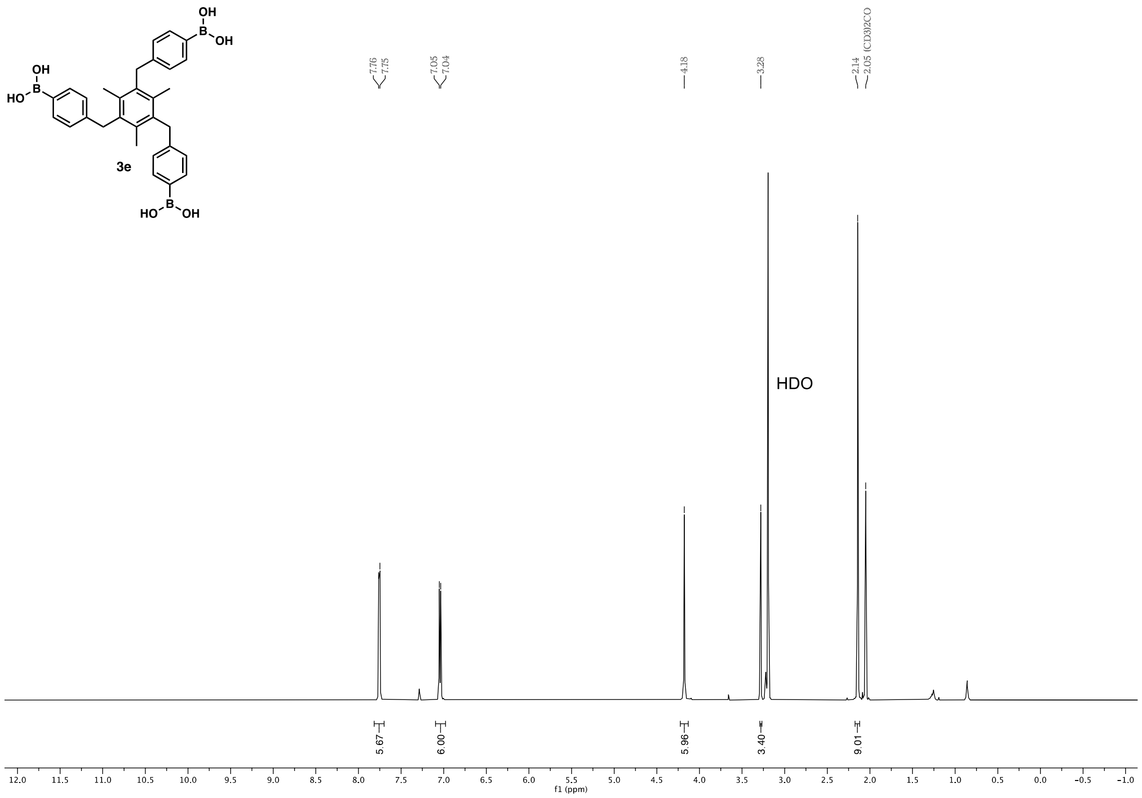

Figure S40: ^{1}H-NMR spectrum of (((2,4,6-trimethylbenzene-1,3,5-triyl)tris(methylene))tris(benzene-4,1diyl))triboronic acid (**3e**) in acetone-d$_6$ with few drops of D_2O (400 MHz).

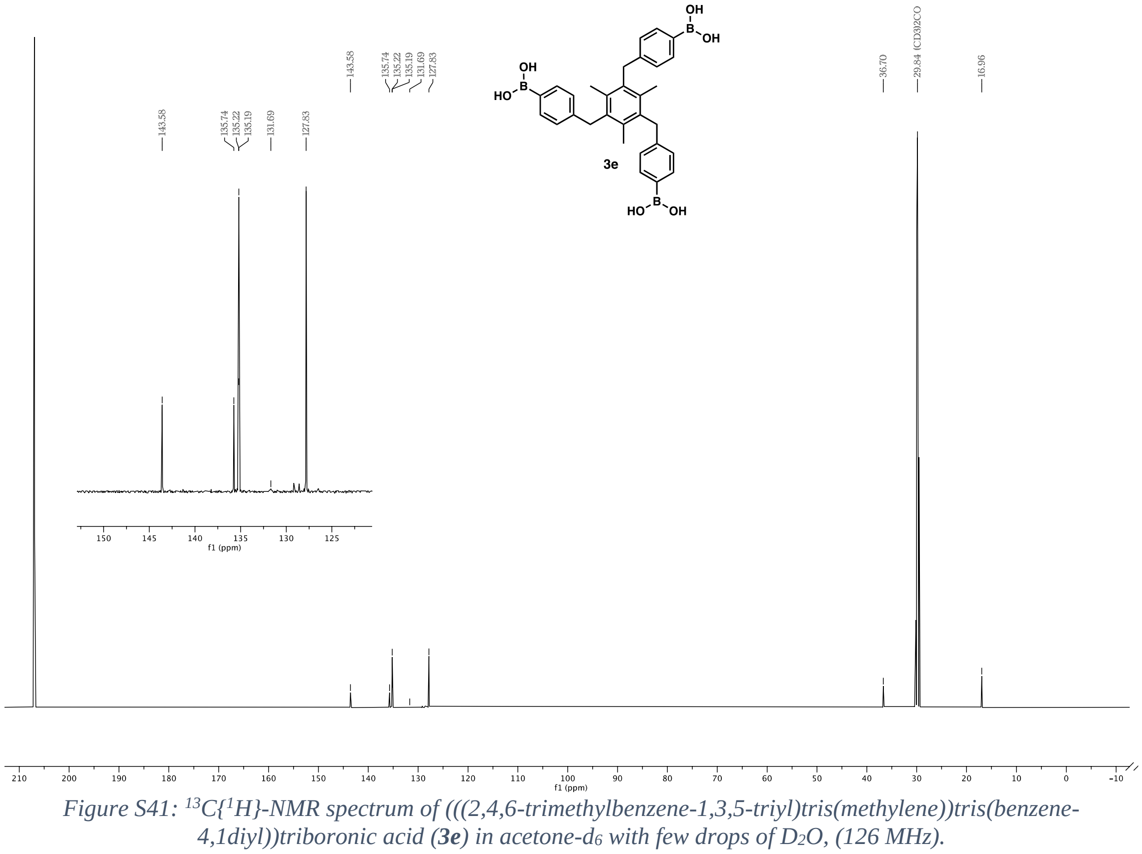

Figure S41: ^{13}C{^{1}H}-NMR spectrum of (((2,4,6-trimethylbenzene-1,3,5-triyl)tris(methylene))tris(benzene-4,1diyl))triboronic acid (**3e**) in acetone-d$_6$ with few drops of D_2O, (126 MHz).

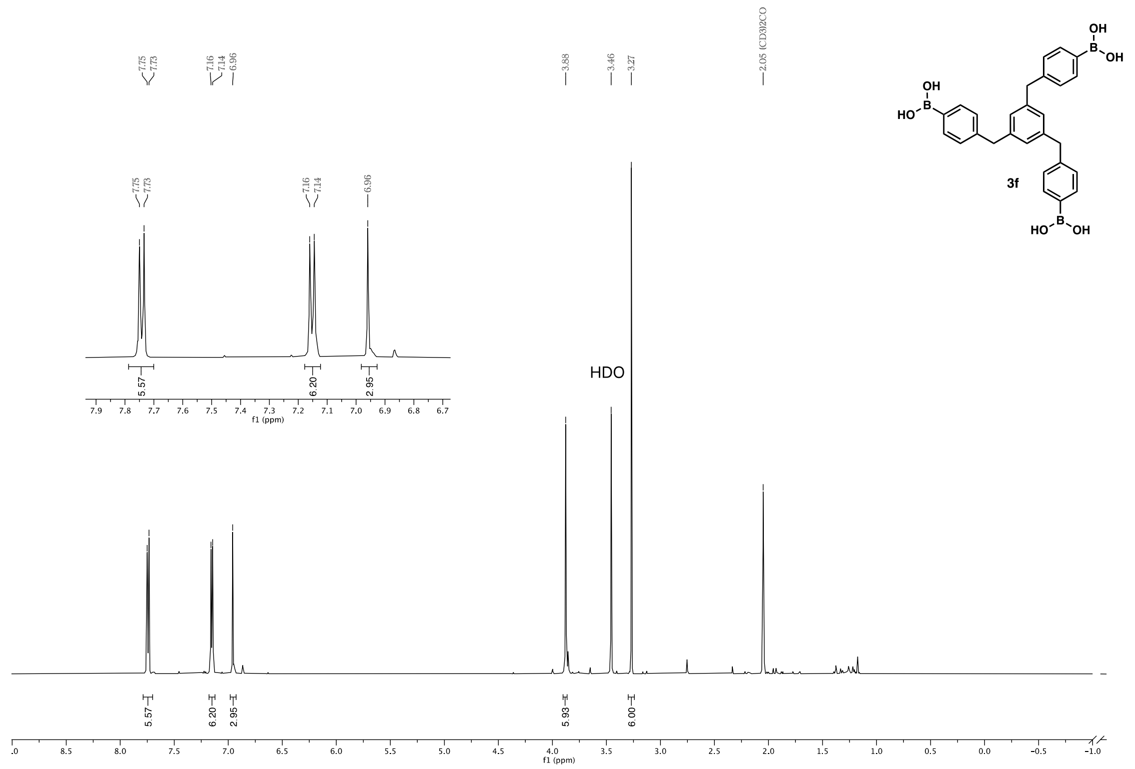

*Figure S42: ^{1}H-NMR spectrum of ((Benzene-1,3,5-triyltris(methylene))tris(benzene-4,1-diyl))triboronic acid (**3f**) in acetone-d_6 with few drops of D_2O (500 MHz).*

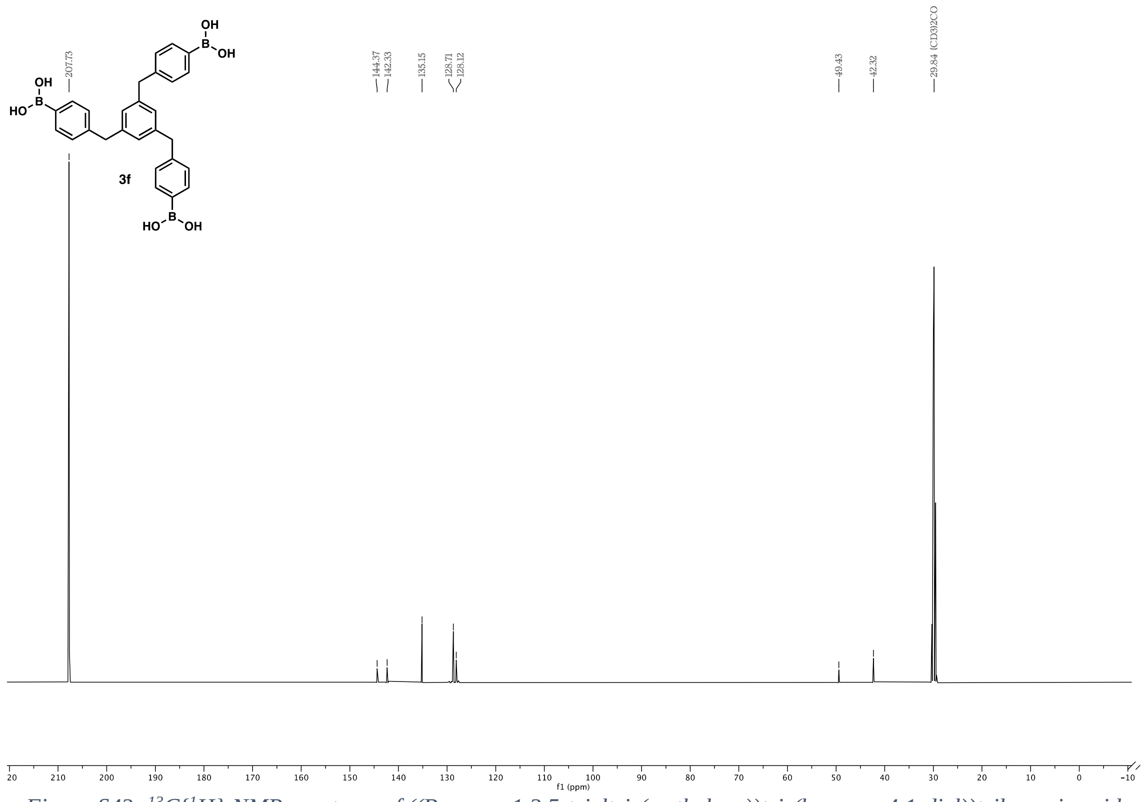

*Figure S43: $^{13}C\{^{1}H\}$-NMR spectrum of ((Benzene-1,3,5-triyltris(methylene))tris(benzene-4,1-diyl))triboronic acid (**3f**) in acetone-d_6 with few drops of D_2O (126 MHz).*

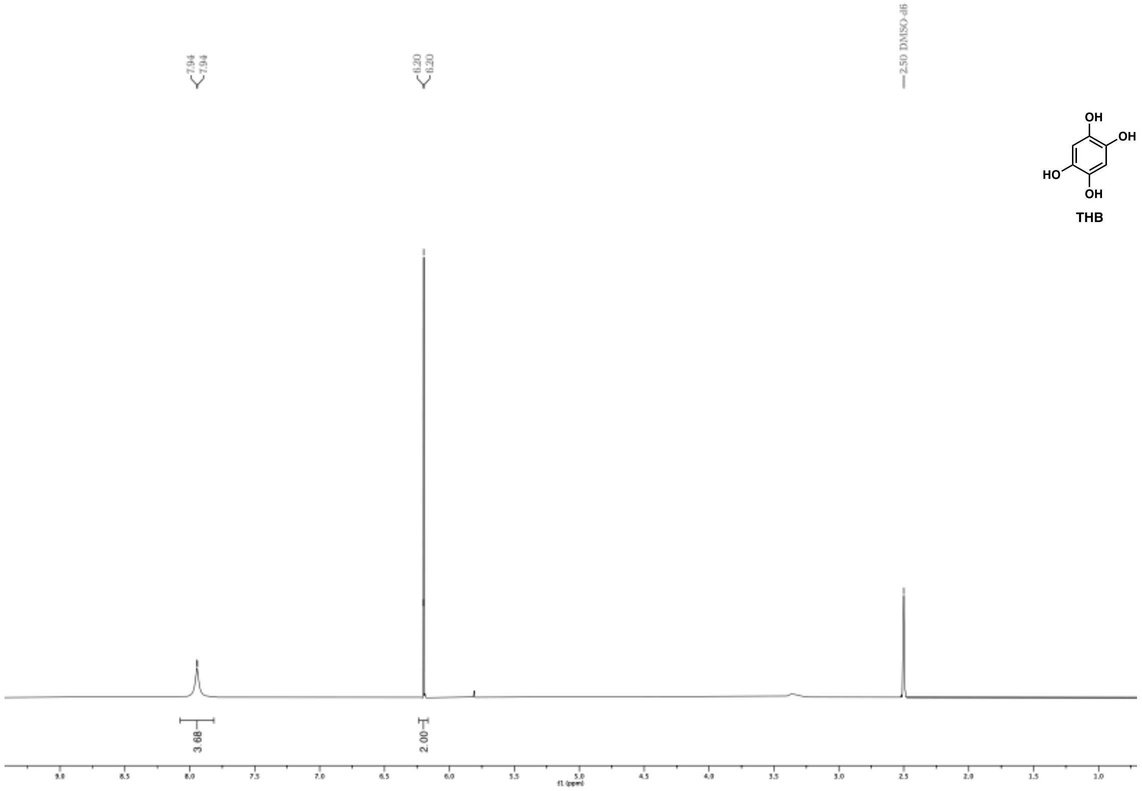

*Figure S44: ^{1}H-NMR spectrum of Benzene-1,2,4,5-tetraol (**THB**), (500 MHz, DMSO-d_6).*

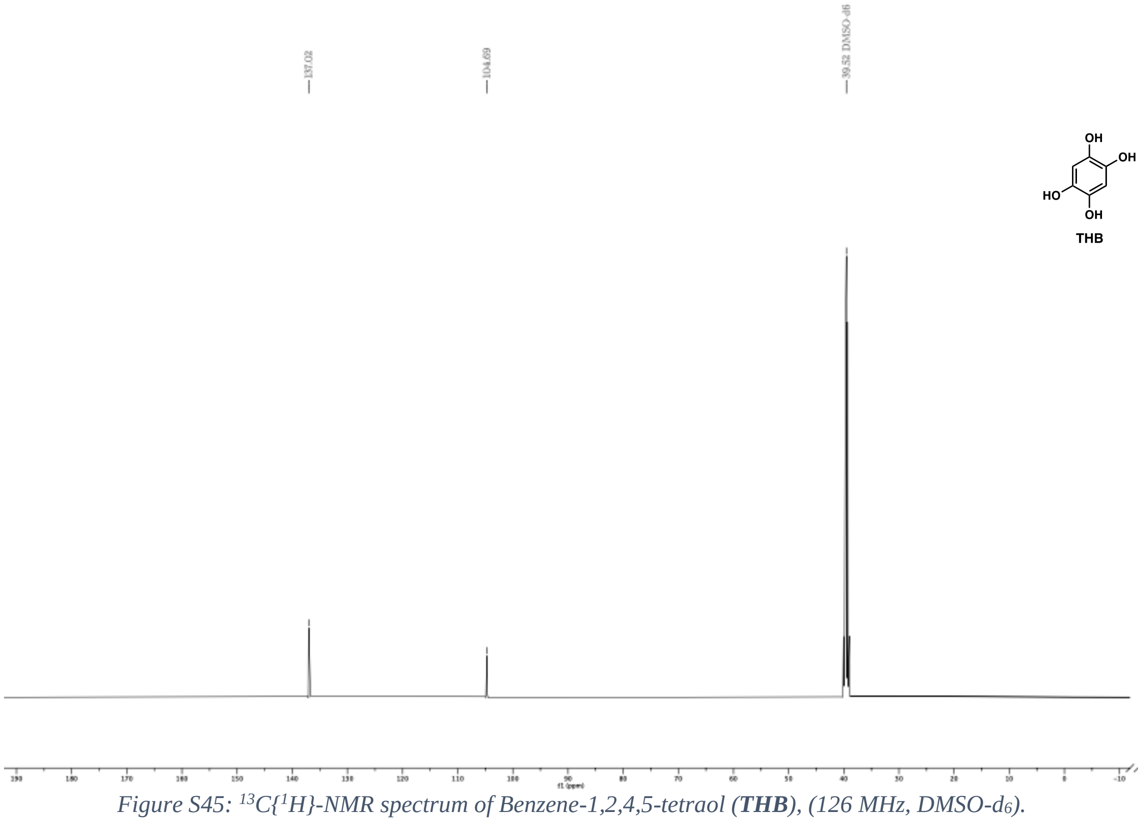

*Figure S45: $^{13}C\{^1H\}$-NMR spectrum of Benzene-1,2,4,5-tetraol (**THB**), (126 MHz, DMSO-d_6).*

5. Synthesis and Characterization of Tetrahedral Cages

General procedure for the formation of boroxine tetrapod cages (TP): A two-necked pressure tube equipped with a magnetic stirring bar was charged with triboronic acid (1.0 equiv) and H_2O (2.0 equiv. per boronic acid moiety) and was evacuated and back-filled with N_2 for a total of five times. Subsequently, anhydrous $CDCl_3$ was added under positive N_2 pressure, the tube sealed tight and heated to 110 °C for 72 hours.
Even after prolonged reaction times of up to 7 days cage formation **could not be observed**.

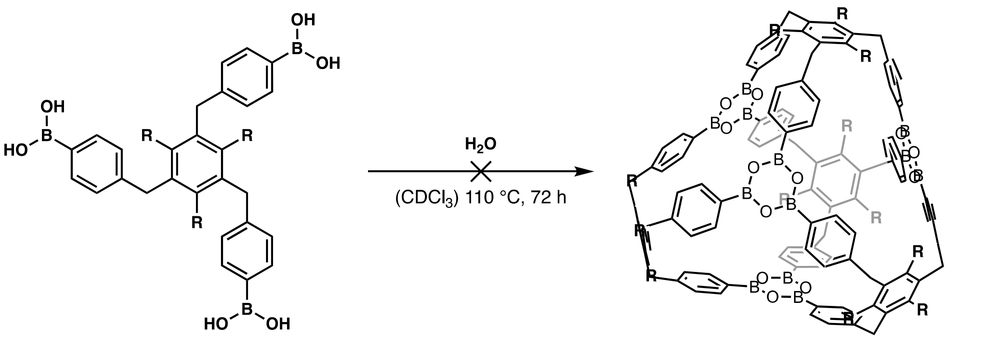

3a: R = CH_2OPh | **TPa**: R = CH_2OPh
3b: R = CH_2CH_3 | **TPb**: R = CH_2CH_3
3c: R = CH_2O-4-C_6H_4-pentyl | **TPc**: R = CH_2O-4-C_6H_4-pentyl
3d: R = $CH_2(CH_2)_3CH_3$ | **TPd**: R = $CH_2(CH_2)_3CH_3$
3e: R = CH_3 | **TPe:** R = CH_3
3f: R = H | **TPf**: R = H

The reaction was carried out using triboronic acid tripods **3a-f**. In none of the cases significant amounts of boroxine cage formation was detected.

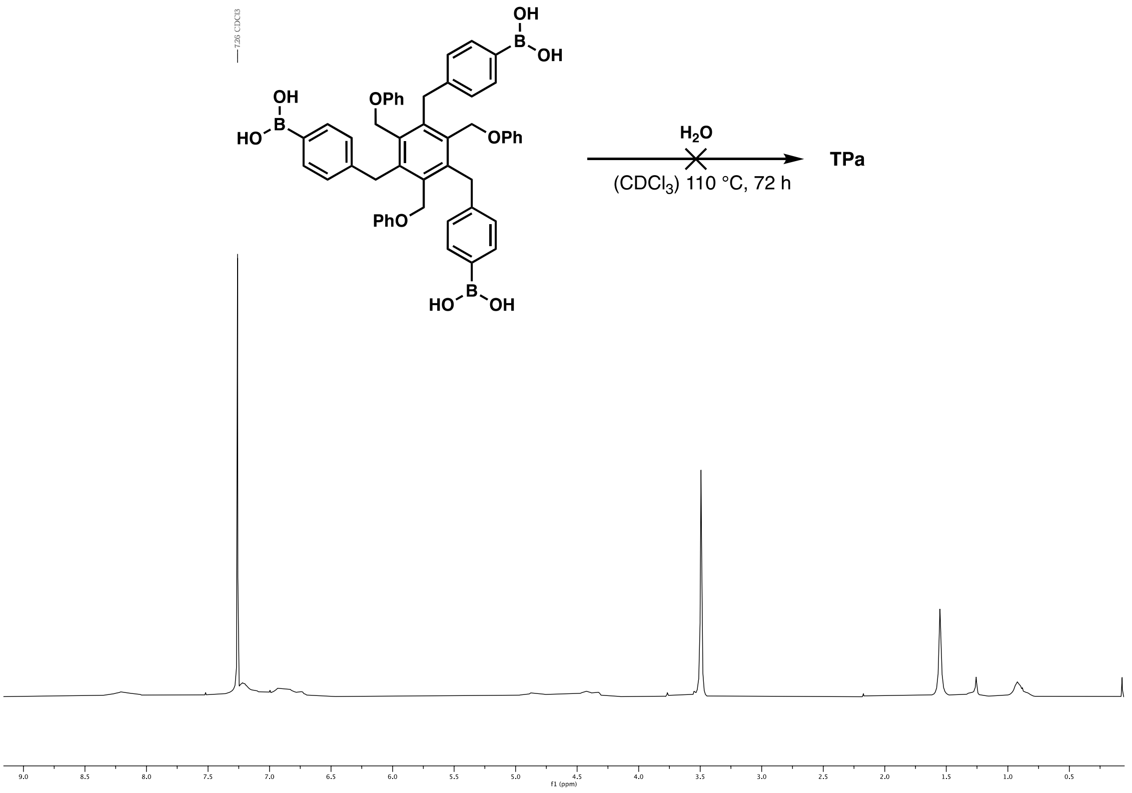

*Figure S46: ^{1}H-NMR spectrum of the trial of boroxine cage formation **TPa** via auto-condensation of **3a** (400 MHz, $CDCl_3$).*

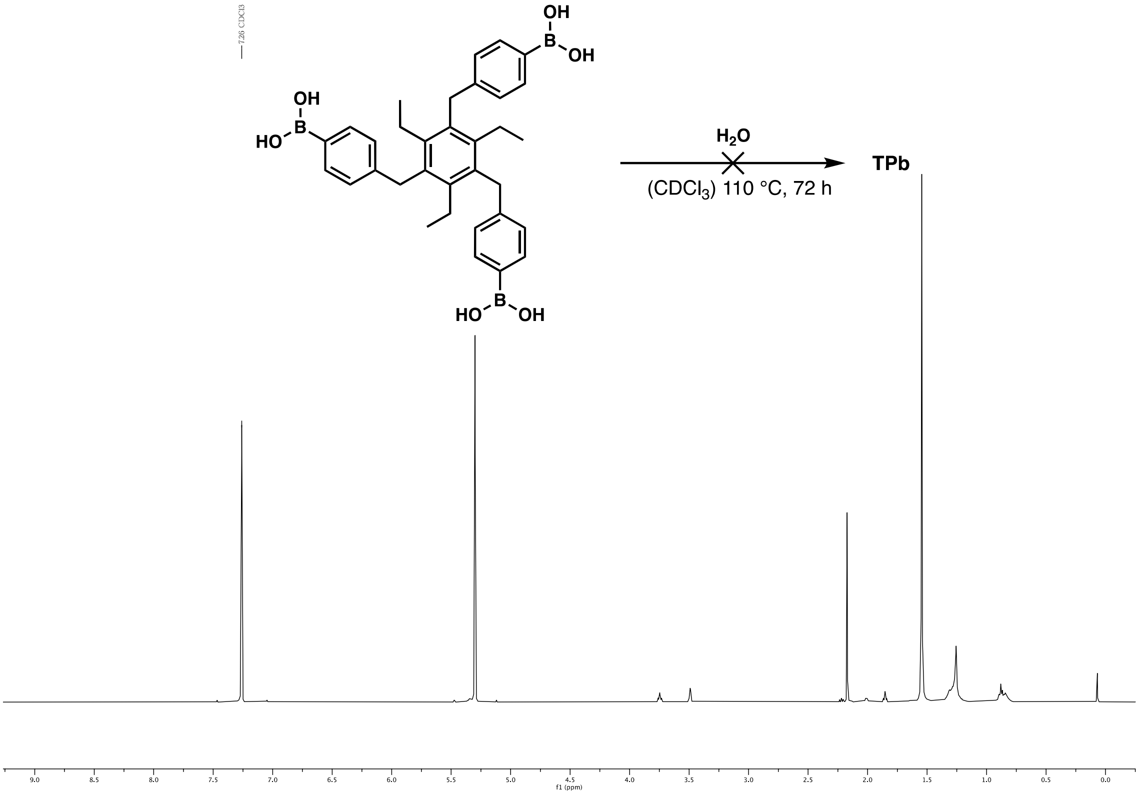

*Figure S47: ^{1}H-NMR spectrum of the trial of boroxine cage formation **TPb** via auto-condensation of **3b**, (400 MHz, CDCl3).*

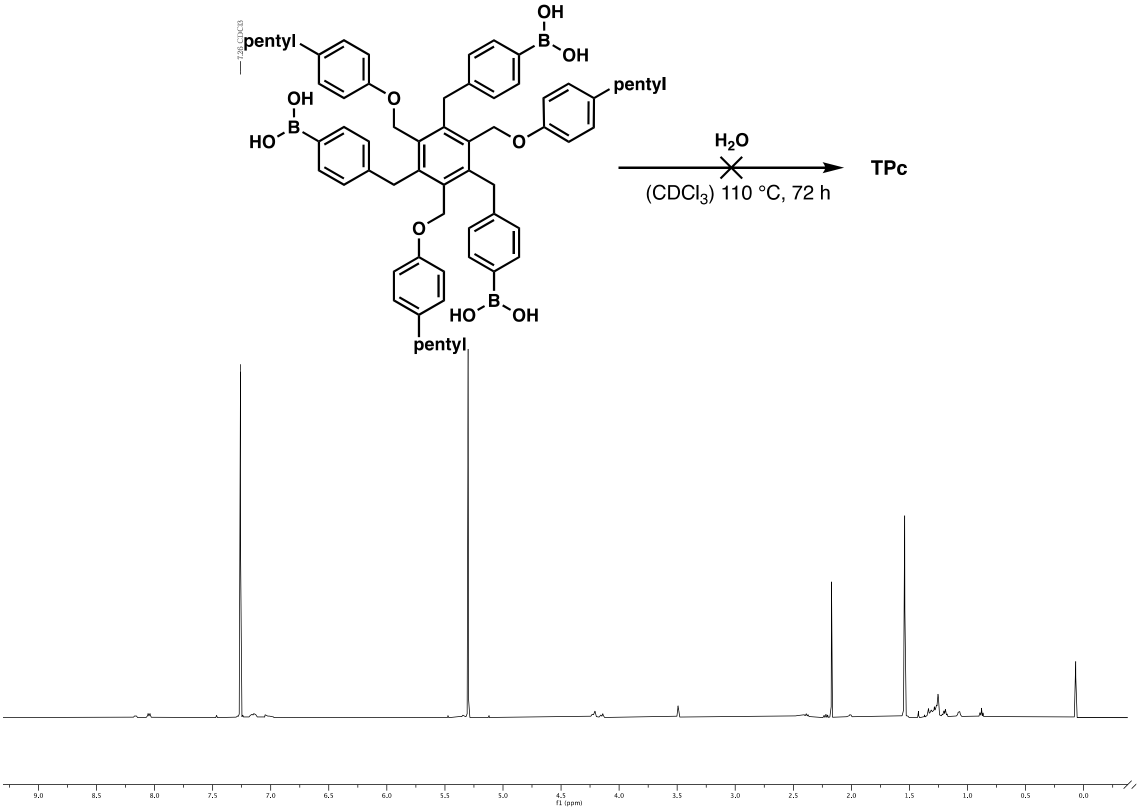

*Figure S48: ^{1}H-NMR spectrum of the trial of boroxine cage formation **TPc** via auto-condensation of **3c**, (400 MHz, CDCl3).*

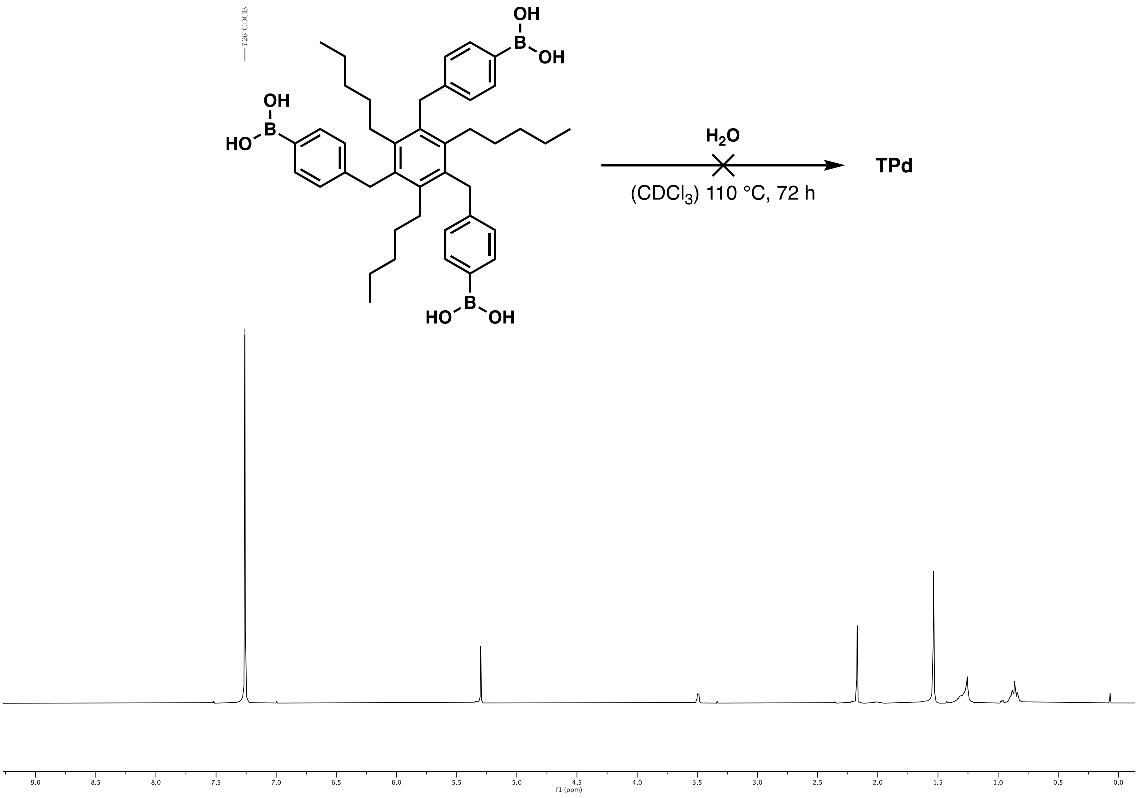

*Figure S49:^{1}H-NMR spectrum of the trial of boroxine cage formation **TPd** via auto-condensation of **3d**, (400 MHz, $CDCl_3$).*

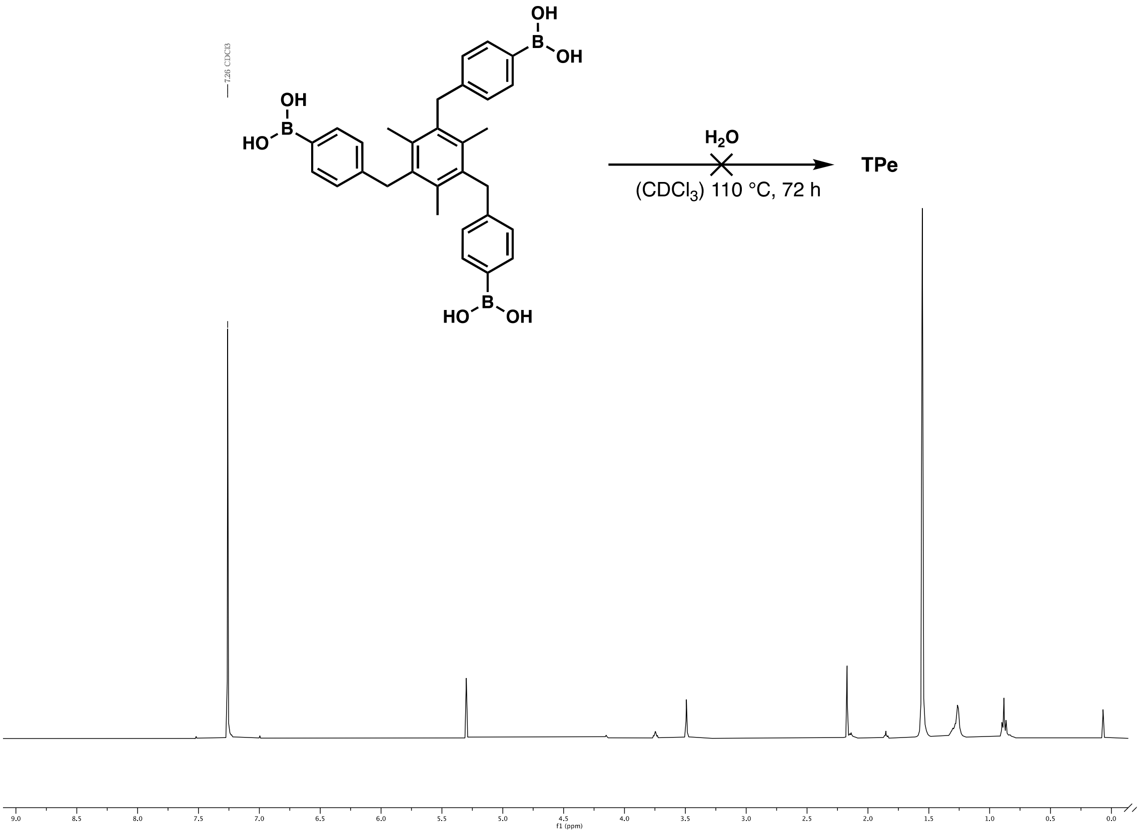

*Figure S50:^{1}H-NMR spectrum of the trial of boroxine cage formation **TPe** via auto-condensation of **3e**, (400 MHz, $CDCl_3$).*

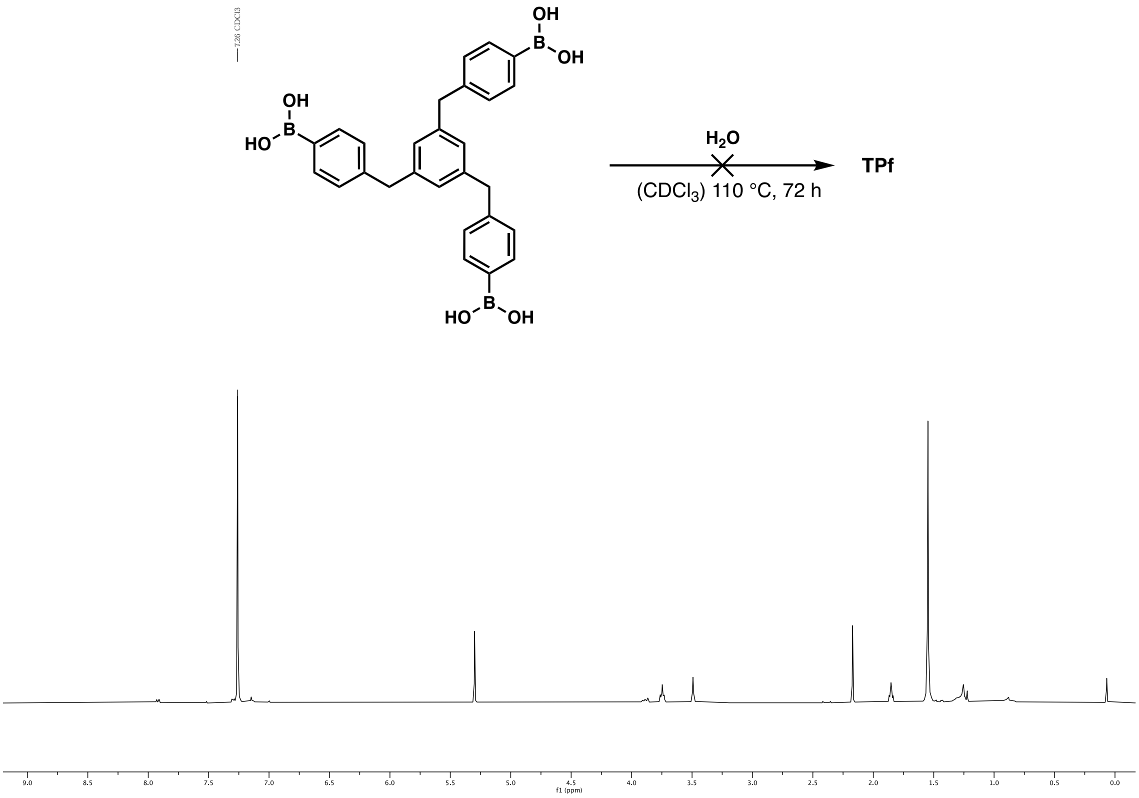

*Figure S51: ^{1}H-NMR spectrum of the trial of boroxine cage formation **TPf** via auto-condensation of **3f**, (400 MHz, $CDCl_3$).*

General procedure A for the formation of boronate tetrahedral cages (T): A two-necked pressure tube equipped with a magnetic stirring bar was charged with triboronic acid (1.0 equiv), benzene-1,2,4,5-tetraol (**THB**) (1.5 equiv.) and H_2O (2.0 equiv. per boronic acid moiety) and was evacuated and back-filled with N_2 for a total of five times. Subsequently, anhydrous $CDCl_3$ was added under positive N_2 pressure, the tube sealed tight and heated to 110 °C. The initially heterogeneous mixture turned homogenous upon completion of the reaction, while on the upper end of the solvent an insoluble solid formed. After allowing to cool to room temperature a 0.5 mL aliquot was extracted under positive N_2 pressure which was analyzed by ^{1}H NMR spectroscopy. The yield was determined adding 1,1,2,2-tetrachloroethane (TCE) as an internal standard and recording a semi-quantitative proton NMR spectrum (for details see Chapter 6).
Removal of the solvent gave a white powder that proved to be difficult to re-dissolve.

Tetrahedral cage Ta

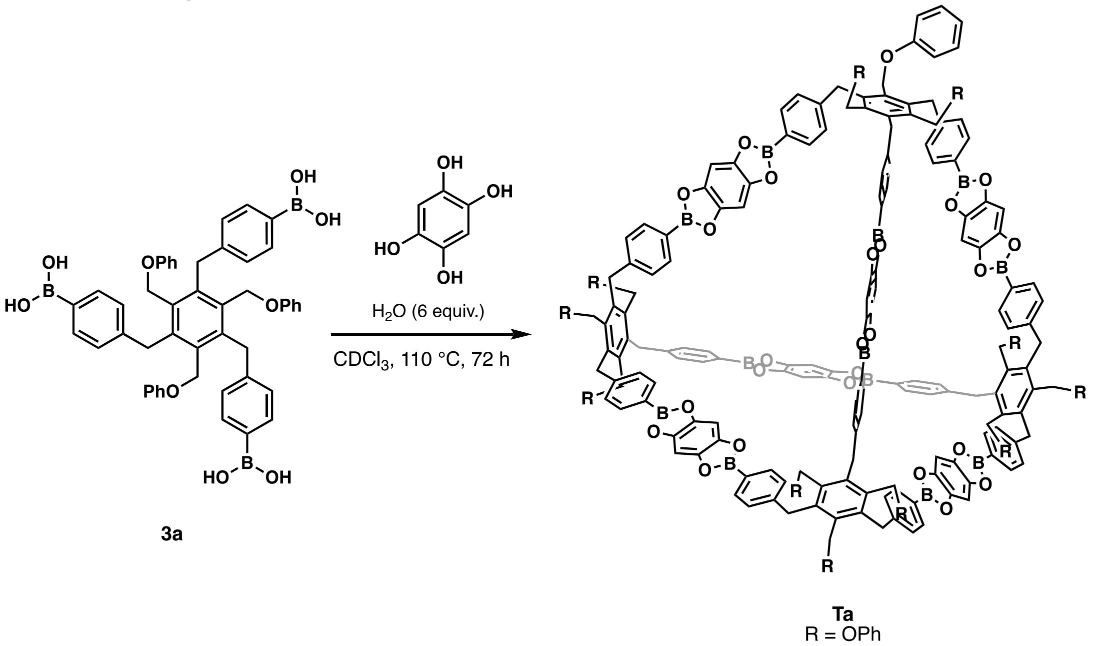

Tetrahedral cage (Ta). General procedure A was applied to triboronic acid precursor **3a** (9.6 mg, 12.0 µmol, 1.0 equiv.), benzene-1,2,4,5-tetraol (**THB**) (2.6 mg, 18.0 µmol, 1.5 equiv.) using 2.4 mL anhydrous $CDCl_3$ (0.005 M) with H_2O (1.3 µL H_2O, 72.2 µmol, 6 equiv.). The product formed after 3 days with a yield of 80 ± 2 %. (See Chapter 6 for details).
Purification of the product was performed as follows. The volume of the reaction mixture was reduced to approx. 1 mL with nitrogen flow and precipitation was induced by addition of 5 mL *n*-pentane. After 1 h, the suspension was centrifugated, decanted and the white precipitate was dissolved in $CDCl_3$. The volume of the solution was reduced to approx. 0.5 mL by rotatory evaporation and 2 mL of $CDCl_3$ were added. This procedure was repeated for four times. During the last cycle, no additional $CDCl_3$ was added and the solution was used directly for NMR spectroscopy.
^{1}H-NMR (500 MHz, $CDCl_3$) δ 8.05 (d, J = 7. Hz, 24H, *H*-5), 7.34 (s, 12H, *H*-10), 7.25 – 7.22 (m, 24H, *H*-16), 7.16 (d, J = 7.9 Hz, 24H, *H*-4), 6.95 (t, J = 7.3 Hz, 12H, *H*-17), 6.85 (d, J = 7.9 Hz, 24H, *H*-15), 4.85 (s, 24H, *H*-12), 4.43 (s, 24H, *H*-2).
^{13}C-NMR{^{1}H} (126 MHz, $CDCl_3$) δ 158.9 (*C*-14), 145.0 (*C*-3), 144.0 (*C*-9), 141.5 (*C*-11), 135.7 (*C*-5), 135.0 (*C*-1), 129.7 (*C*-16), 127.8 (*C*-4), 123.8 (*C*-6), 121.3 (*C*-17), 114.6 (*C*-15), 98.5 (*C*-10), 64.7 (*C*-12), 35.3 (*C*-2).
^{1}H DOSY-NMR (500 MHz, $CDCl_3$): D = 2.83 ± 0.02 x 10^{-10} m^2/s.

HRMS (MALDI+, DCTB, AgTFA) *m/z* calcd. for $C_{228}H_{168}B_{12}O_{36}Ag$ $[M+Ag]^+$ 3720.1637, found 3720.1682.
IR *ν* = 752.9, 861.0, 1017.6, 1136.8, 1222.6, 1364.2, 1394.0, 1490.9 cm^{-1}.

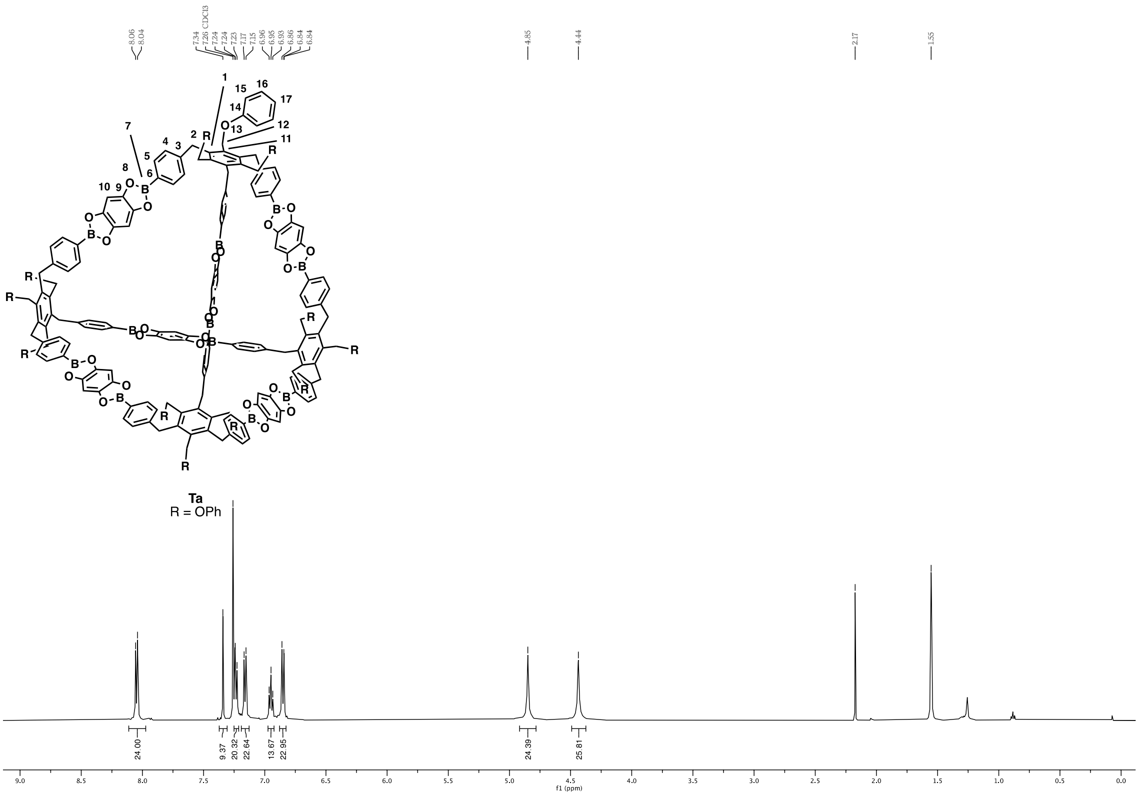

*Figure S52: ^{1}H-NMR spectrum of tetrahedral cage **Ta** (500 MHz, $CDCl_3$).*

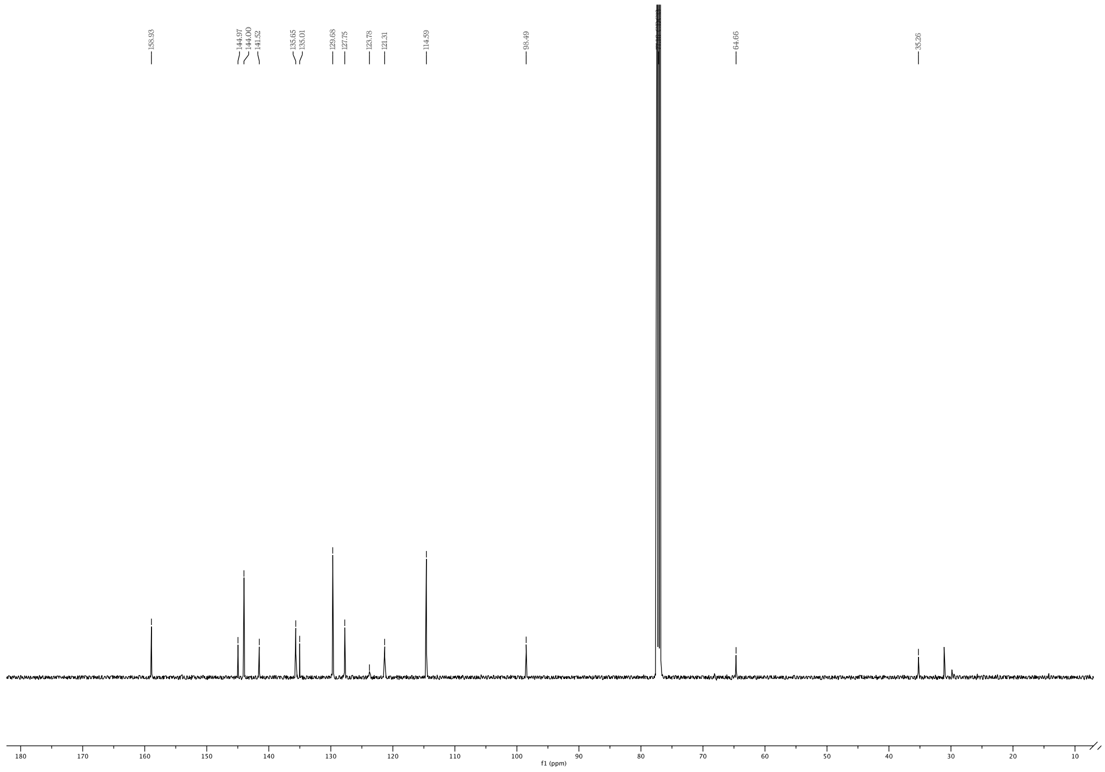

*Figure S53: $^{13}C\{^{1}H\}$-NMR spectrum of tetrahedral cage **Ta** (126 MHz, $CDCl_3$).*

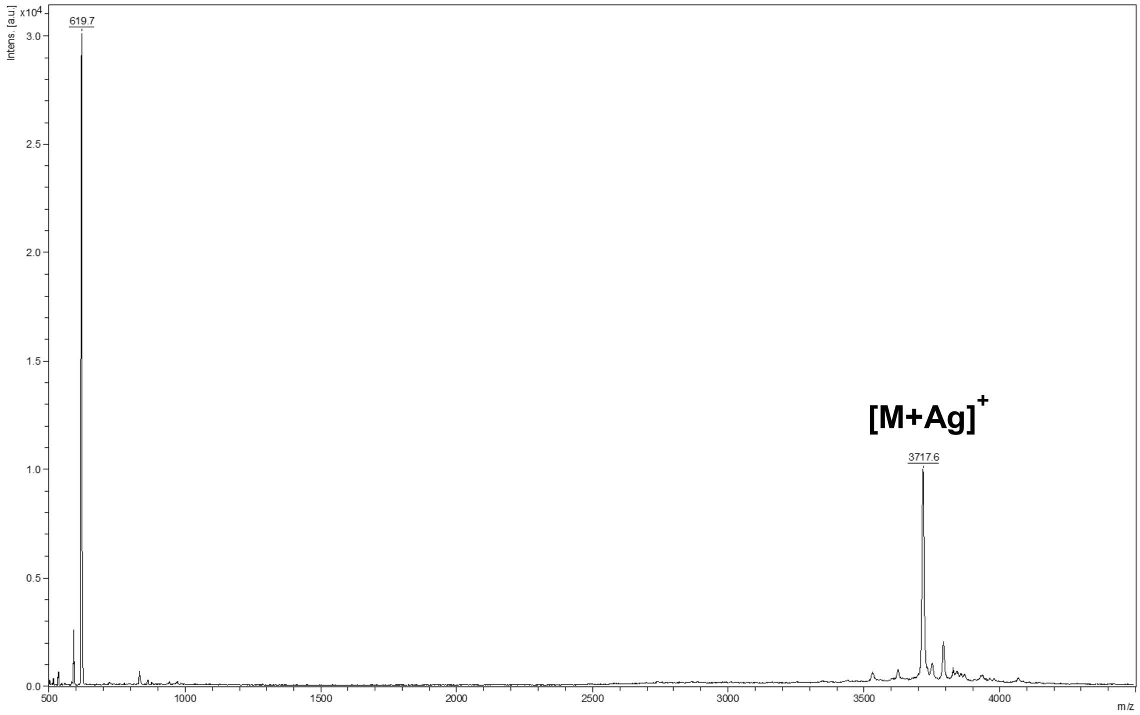

*Figure S54: MALDI-TOF spectrum of tetrahedral cage **Ta**.*

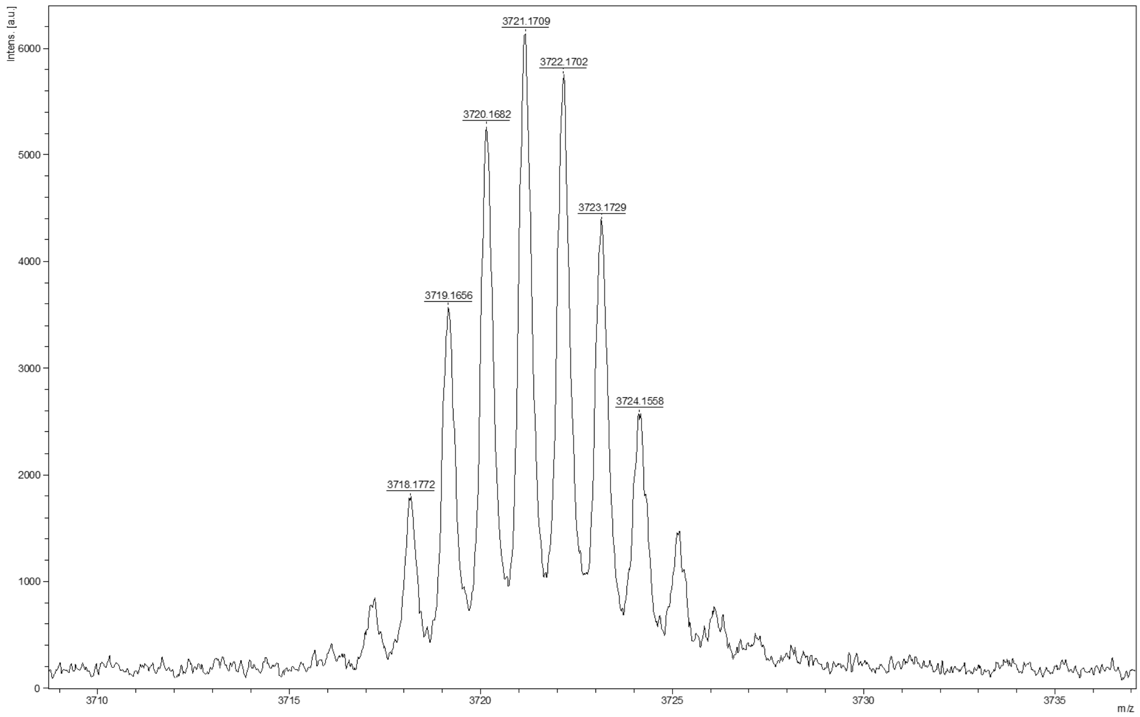

*Figure S55: HR-MALDI isotopic pattern of tetrahedral cage **Ta**.*

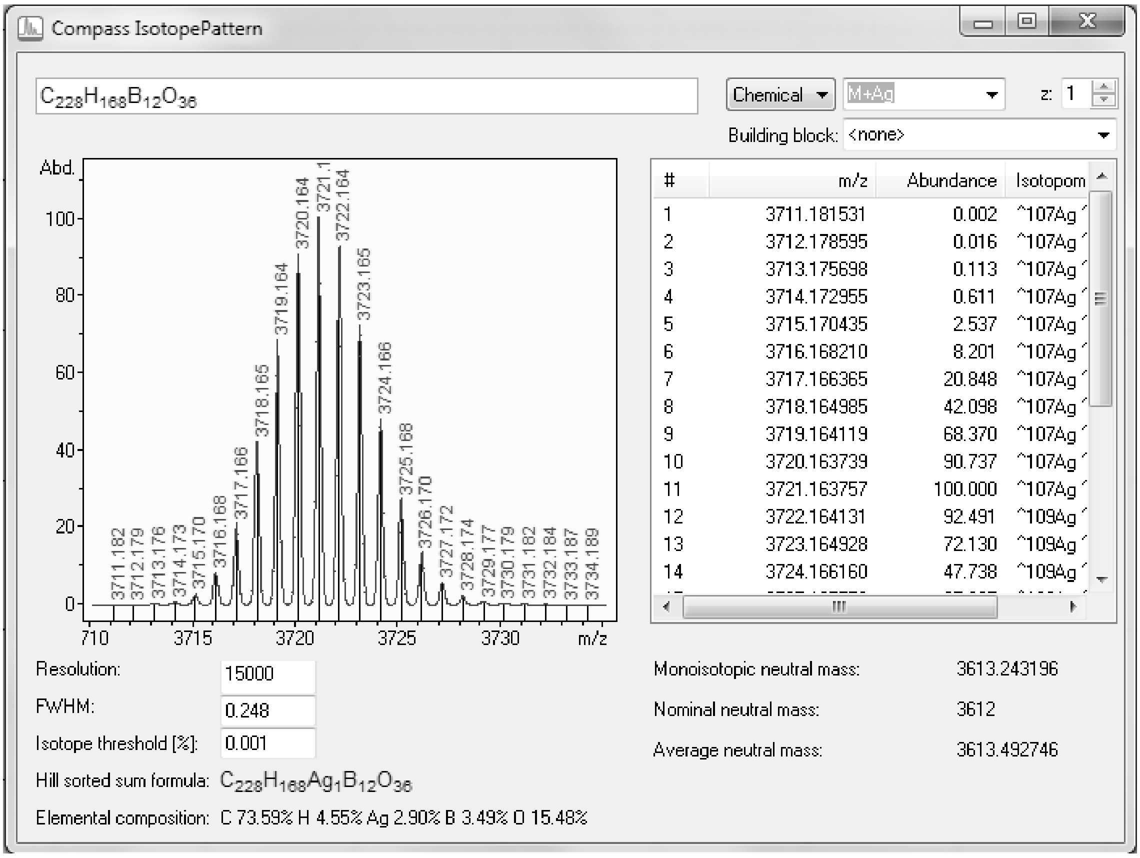

*Figure S56: Simulated HD-MALDI isotopic pattern of tetrahedral cage **Ta**.*

Tetrahedral cage Tb

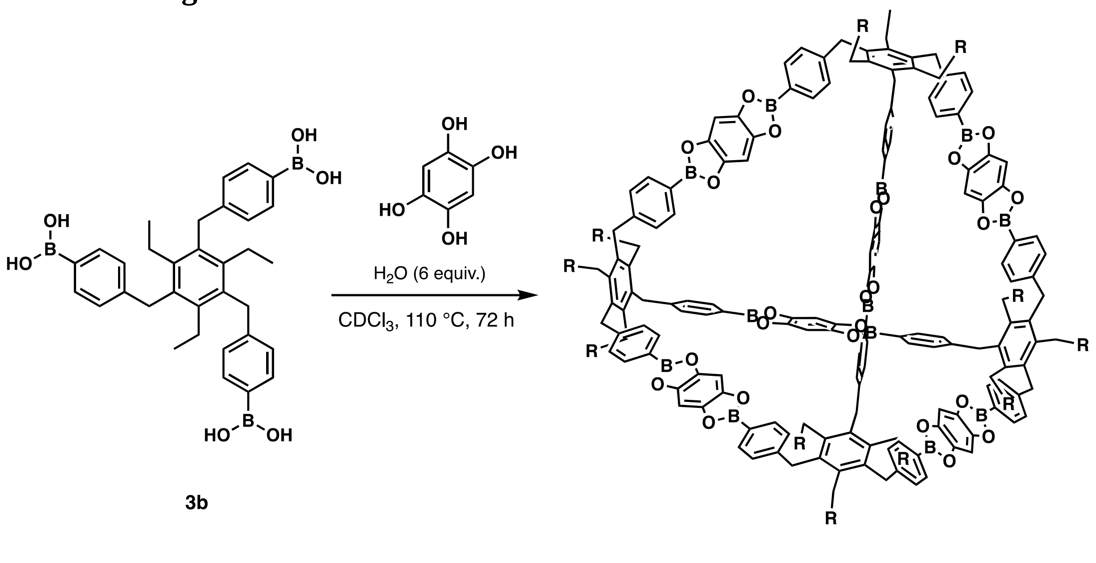

3b

Tb
R = CH_3

Tetrahedral cage (Tb). General procedure A was applied to triboronic acid precursor **3b** (9.5 mg, 16.8 µmol, 1.0 equiv.), benzene-1,2,4,5-tetraol (**THB**) (3.6 mg, 25.3 µmol, 1.5 equiv.) using 3.4 mL anhydrous $CDCl_3$ (0.005 M) with H_2O (1.8 µL, 101.1 µmol, 6.0 equiv.). The product formed after 3 days with an average yield of 38 ± 2 %. (See Chapter 6 for details).

Purification of **Tb** was carried out by size-exclusion chromatography using a Ultrastyragel® 500Å (19x300mm) column on a Waters 600 E with UV detection at 254 nm and CH_2Cl_2 as solvent. The fractions containing the product were joined and the volume was reduced to approx. 1 mL by rotary evaporation. 2 mL of CCl_4 were added and the volume was reduced to approx. 0.5 mL. This procedure was repeated for a total of five times. In the last cycle, $CDCl_3$ was employed instead of CCl_4 and the solution was directly used for NMR spectroscopy.

^{1}H-NMR (500 MHz, $CDCl_3$) δ 8.04 (d, *J* = 8.1 Hz, 24H, *H*-5), 7.31 (s, 12H, *H*-10), 7.20 (d, *J* = 7.8 Hz, 24H, *H*-4), 4.24 (s, 24H, *H*-2), 2.46 (d, *J* = 7.6 Hz, 24H, *H*-12), 1.21 (t, *J* = 7.5 Hz, 36H, *H*-13).

^{13}C-NMR{^{1}H} (126 MHz, $CDCl_3$) δ 145.8 (*C*-3), 144.1 (*C*-9), 141.8 (*C*-11), 135.7 (*C*-5), 133.4 (*C*-1) 127.7 (*C*-4), 123.4 (*C*-6), 98.5 (*C*-10), 35.4 (*C*-2), 23.9 (*C*-12), 15.3 (*C*-13).

^{1}H DOSY-NMR (500 MHz, $CDCl_3$): *D* = 3.10 ± 0.07 x 10^{-10} m^2/s.

HRMS (MALDI+, DCTB, AgTFA) *m/z* calcd. for $C_{168}H_{144}B_{12}O_{24}$ $[M]^+$ 2676.1296, found 2676.1291.

IR ν = 734.3, 909.5, 1136.8, 1364.2, 1610.2, 2255.0, 2929.7, 2959.5 cm^{-1}.

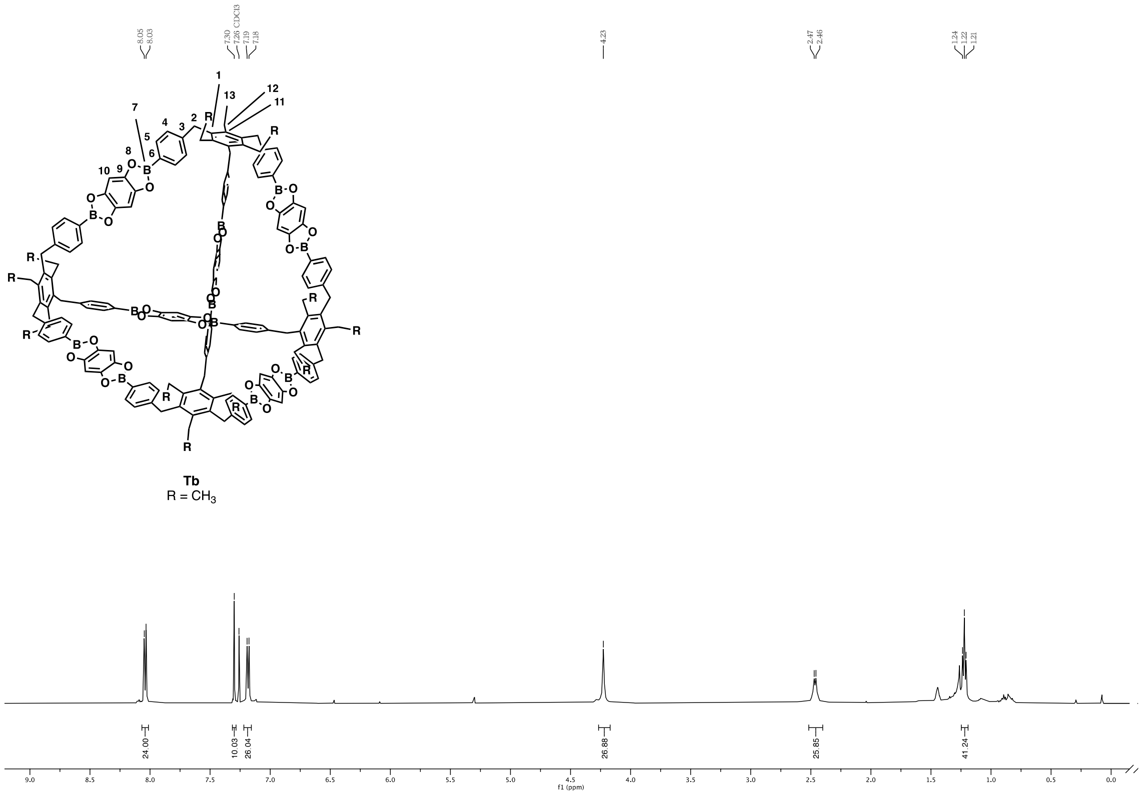

*Figure S57: ^{1}H-NMR spectrum of tetrahedral cage **Tb** (500 MHz, $CDCl_3$).*

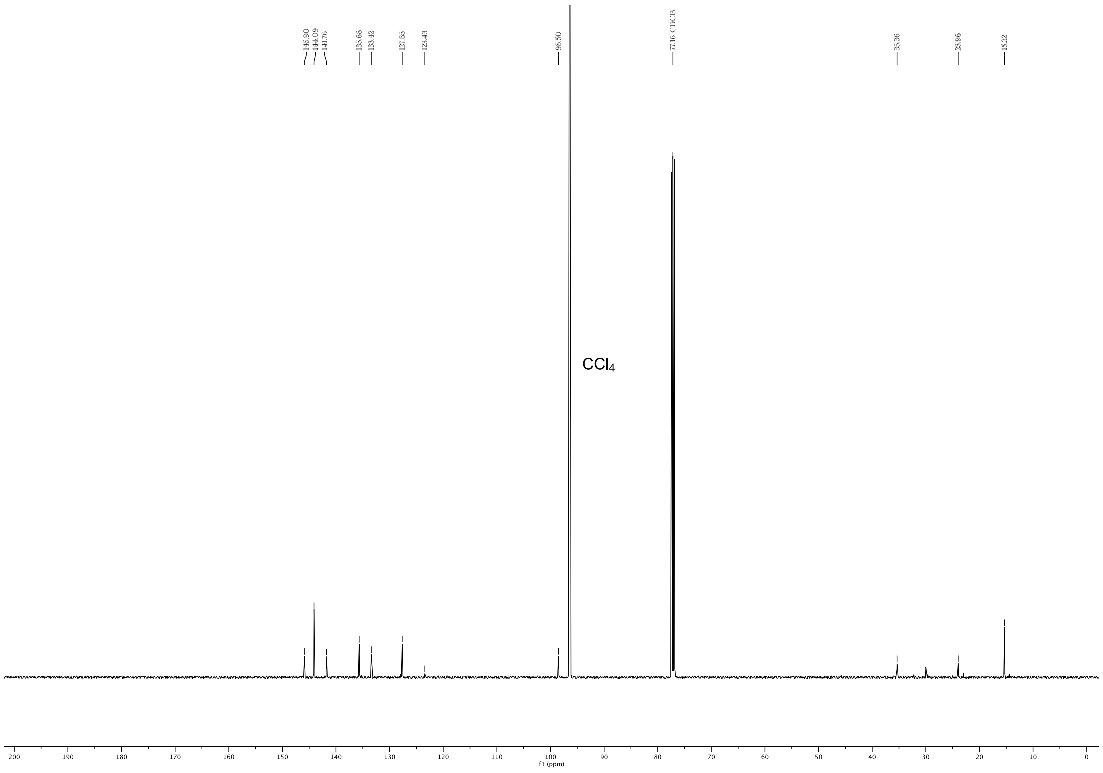

*Figure S58: $^{13}C\{^{1}H\}$-NMR spectrum of tetrahedral cage **Tb** (126 MHz, $CDCl_3$).*

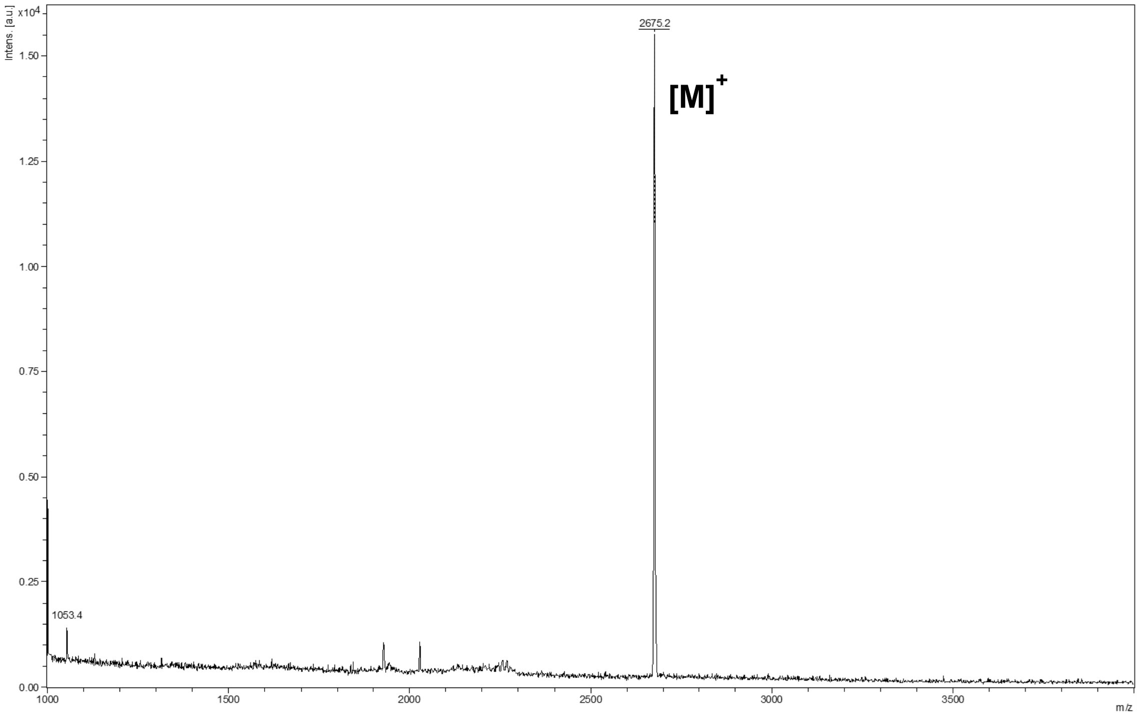

*Figure S59: MALDI-TOF spectrum of tetrahedral cage **Tb**.*

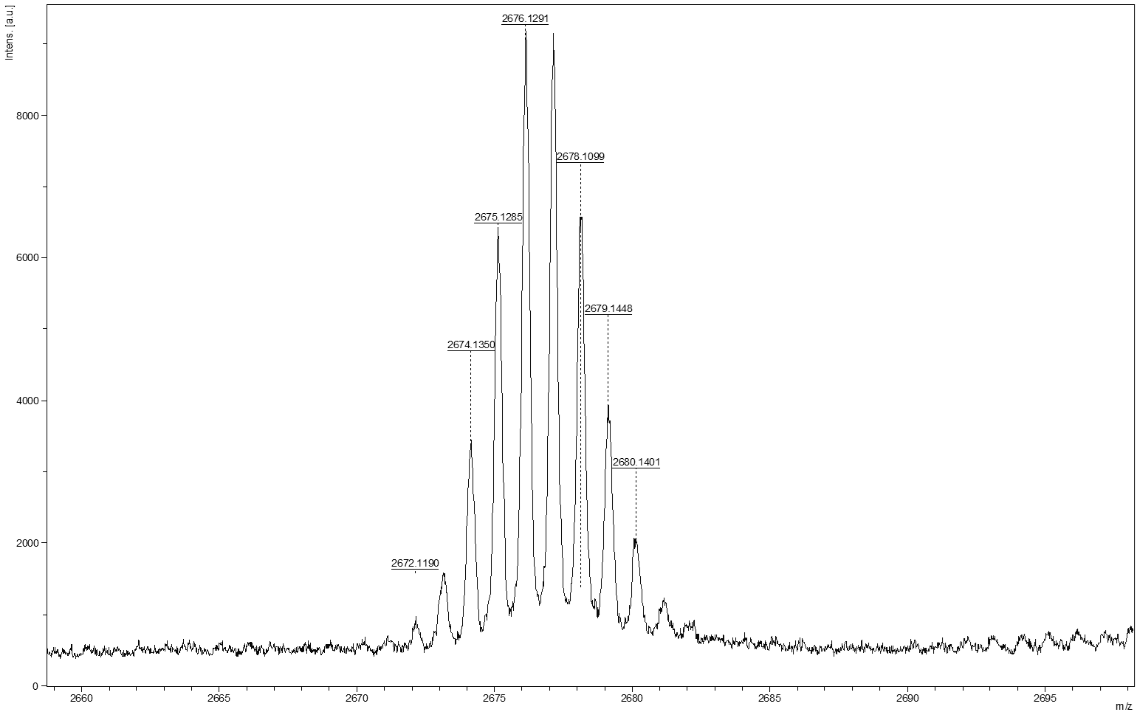

*Figure S60: HR-MALDI isotopic pattern of tetrahedral cage **Tb**.*

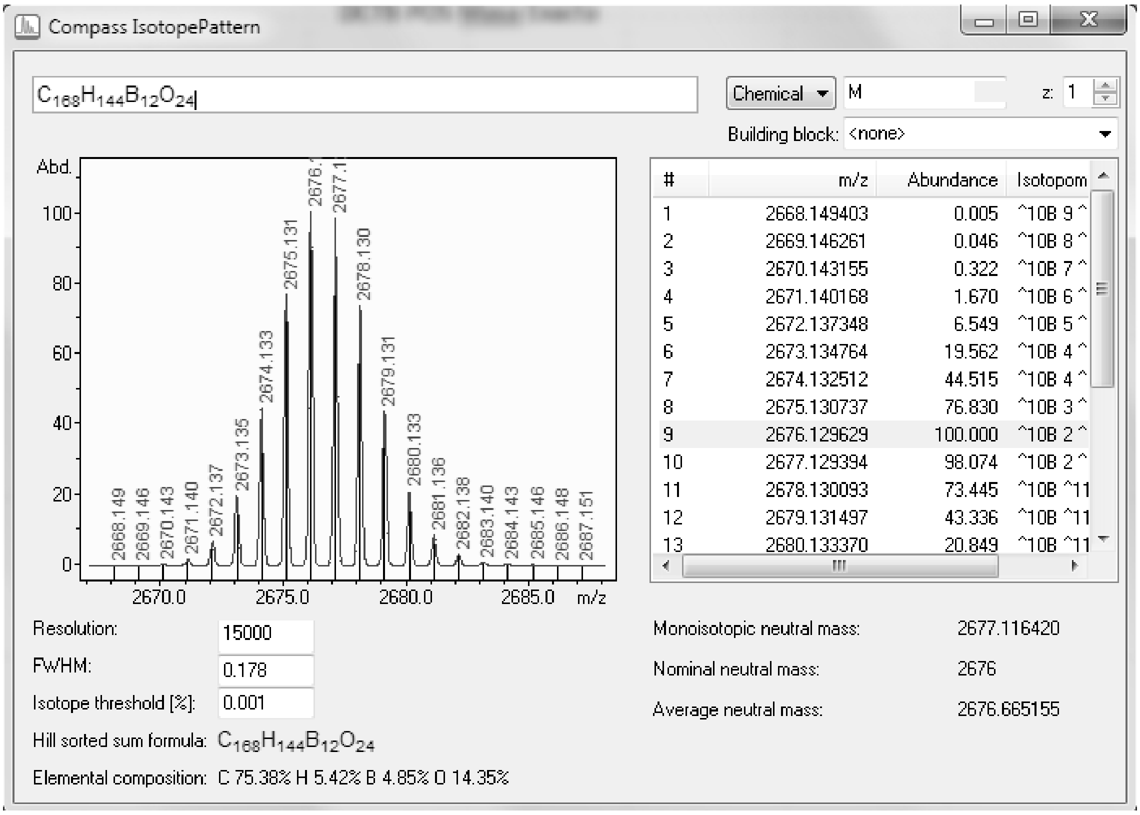

*Figure S61: Simulated HR-MALDI isotopic pattern of tetrahedral cage **Tb**.*

During the reaction a small amount of solid forms on the upper rim of the reaction mixture (Figure S61).

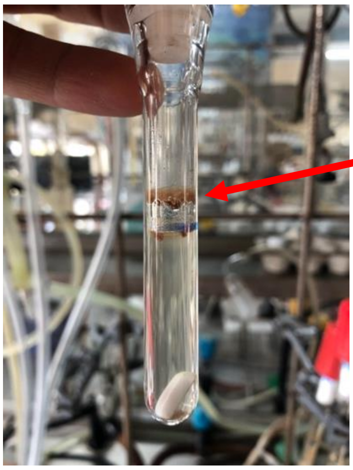

*Figure S62: Picture of the finished reaction of the formation of tetrahedral cage **Tb**. During the reaction, a solid forms on the upper rim of the solvent inside the pressure tube.*

The precipitate was suspended in $CDCl_3$ and sonicated but proved to be completely insoluble (this was judged by ^{1}H-NMR). To further analyze the solid, it was taken up in acetone-d_6, sonicated for five minutes and the supernatant liquid was analyzed by ^{1}H-NMR and MALDI-TOF-MS. The MALDI-TOF-MS showed no traces of cage **Tb** nor starting materials and the ^{1}H-NMR spectrum is depicted in Figure S62 and shows only the presence of traces of 1,2,4,5-tetrahydroxybenzene (**THB**). Thus, it can be ruled out that it consists of boronic acid starting material (which is perfectly soluble in acetone). We concluded that it consists of insoluble oligo/polymeric species that fall outside the

reaction equilibrium. The non-quantitative yield of cage formation (especially for **Tb**) is consistent with the formation of this insoluble side-products.

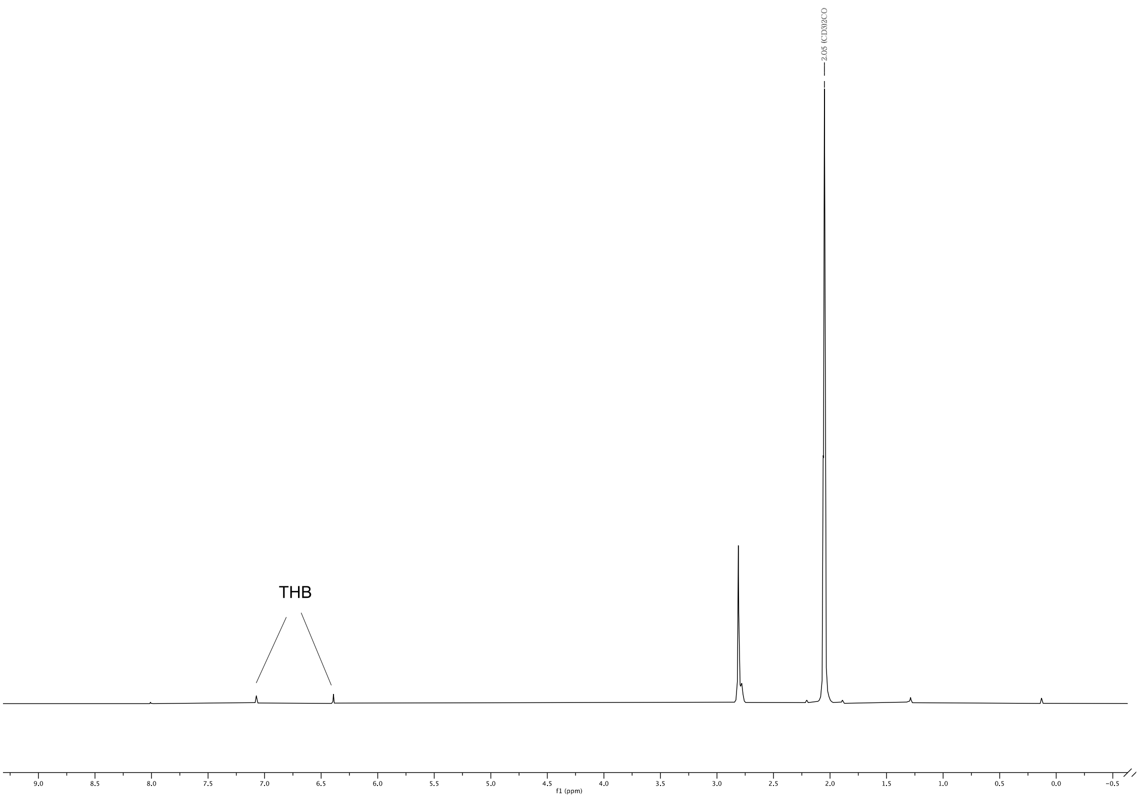

Figure S63: ^{1}H-NMR spectrum in acetone-d$_6$ of the solid that formed on the upper end of the reaction mixture of ***Tb*** *(500 MHz, acetone-d$_6$). Only H_2O and traces of* ***THB*** *are detectable.*

Tetrahedral cage Tc

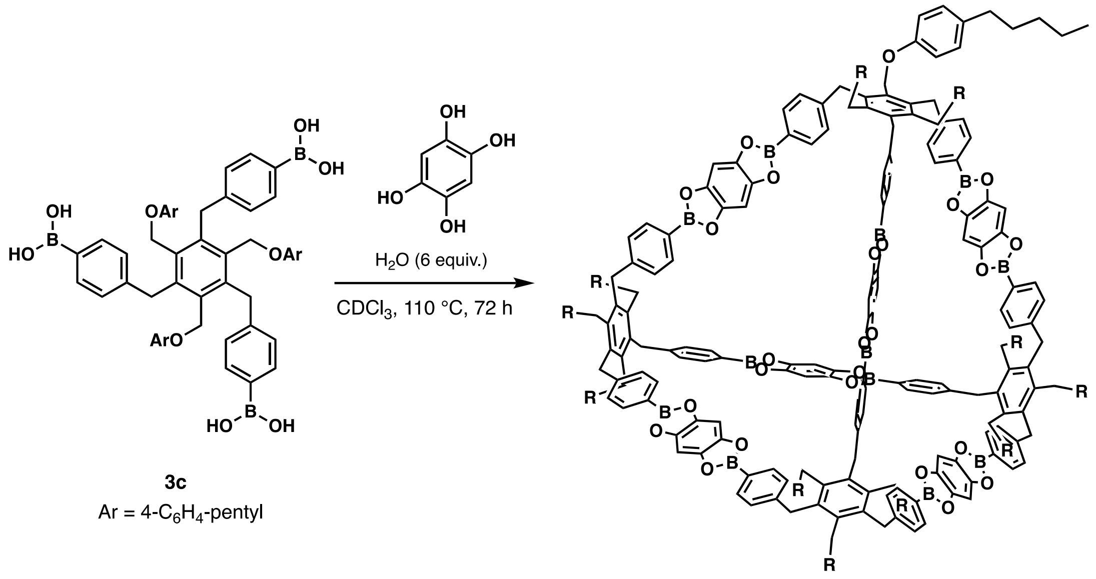

3c
Ar = 4-C_6H_4-pentyl

Tc
R = O-4-C_6H_4-pentyl

Tetrahedral cage (Tc). General procedure A was applied to triboronic acid precursor **3c** (10.1 mg, 10.0 µmol, 1.0 equiv.), benzene-1,2,4,5-tetraol (**THB**) (2.1 mg, 15.0 µmol, 1.5 equiv.) using 2.0 mL anhydrous $CDCl_3$ (0.005 M) with H_2O (1.1 µL, 60.0 µmol, 6.0 equiv.). The product formed after 2 days with an average yield of 82 ± 2% (See Chapter 6 for details).

Purification of **Tc** was carried out by size-exclusion chromatography using a Ultrastyragel® 500Å (19x300mm) column on a Waters 600 E with UV detection at 254 nm and CH_2Cl_2 as solvent. The fractions containing the product were joined and the volume was reduced to approx. 1 mL by rotary evaporation. 2 mL of CCl_4 were added and the volume was reduced to approx. 0.5 mL. This procedure was repeated for a total of five times. In the last cycle, $CDCl_3$ was employed instead of CCl_4 and the solution was directly used for NMR spectroscopy.

^{1}H-NMR (400 MHz, $CDCl_3$) δ 8.04 (d, *J* = 7.8 Hz, 24H, *H*-5), 7.34 (s, 12H, *H*-10), 7.16 (d, *J* = 7.8 Hz, 24H, *H*-4), 7.05 (d 24H, *H*-16), 6.76 (d, *J* = 8.7 Hz, 24H, *H*-15), 4.81 (s, 24H, *H*-12), 4.43 (s, 24H, *H*-2), 2.51 (t, *J* = 7.8 Hz, 24H, *H*-18), 1.62 – 1.48 (m, 24H, *H*-19), 1.34 – 1.22 (m, 48H, *H*-20/21), 0.87 (t, *J* = 6.8 Hz, 36H, *H*-22).

^{13}C-NMR{^{1}H} (126 MHz, $CDCl_3$) δ 157.0 (*C*-14), 145.1 (*C*-3), 144.0 (*C*-9), 141.4 (*C*-11), 135.8 (*C*-17), 135.6 (*C*-5), 135.1 (*C*-1), 129.4 (*C*-16), 127.8 (*C*-4), 123.7 (*C*-6), 114.4 (*C*-15), 98.5 (*C*-10), 64.8 (*C*-12), 35.2 (*C*-2), 35.2 (*C*-18), 31.6 (*C*-19), 31.6 (*C*-20), 22.7 (*C*-21), 14.2 (*C*-22).

^{1}H-DOSY-NMR (500 MHz, $CDCl_3$): $D = 2.36 \pm 0.02 \times 10^{-10}$ m^2/s.

HRMS (MALDI+, DCTB, AgTFA) *m/z* calcd. for $C_{288}H_{288}B_{12}O_{36}Ag$ $[M+Ag]^+$ 4563.1051, found 4563.1024.

IR *ν* = 752.9, 861.1, 1071.7, 1136.8, 1226.3, 1364.2, 1490.9, 2855.1, 2926.0, 2955.8 cm^{-1}.

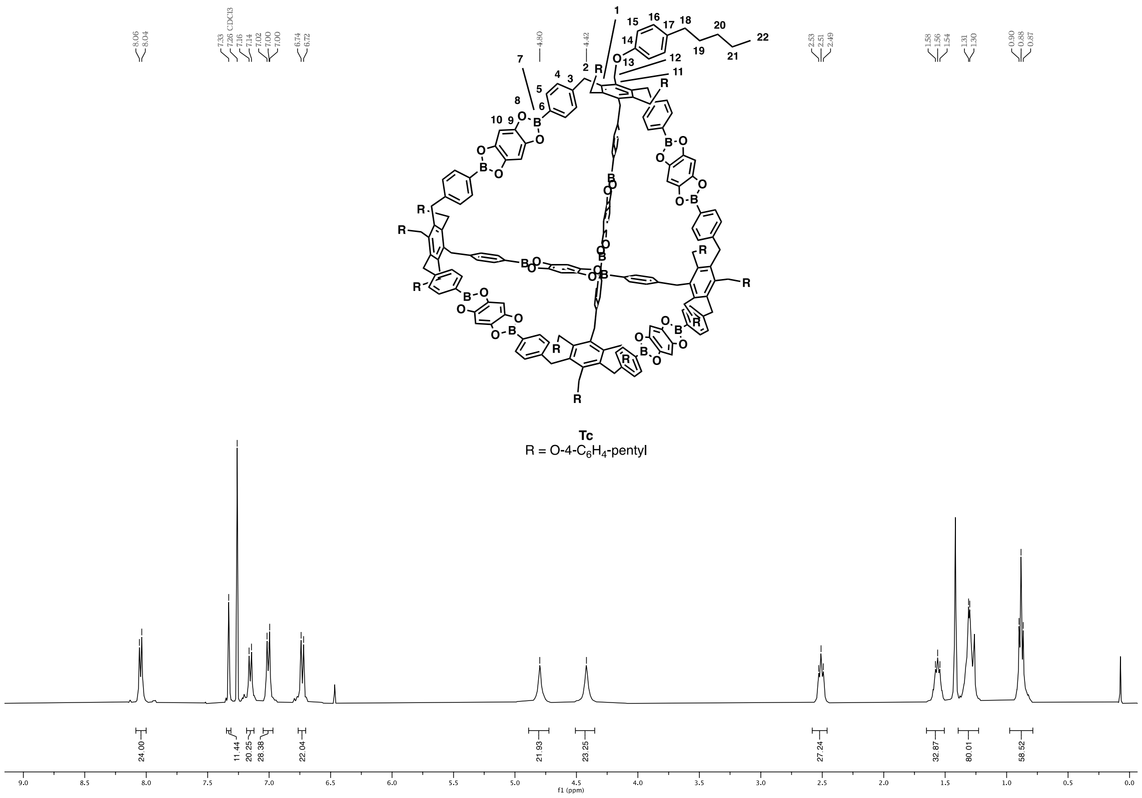

*Figure S64: ¹H-NMR spectrum of tetrahedral cage **Tc** (500 MHz, CDCl₃).*

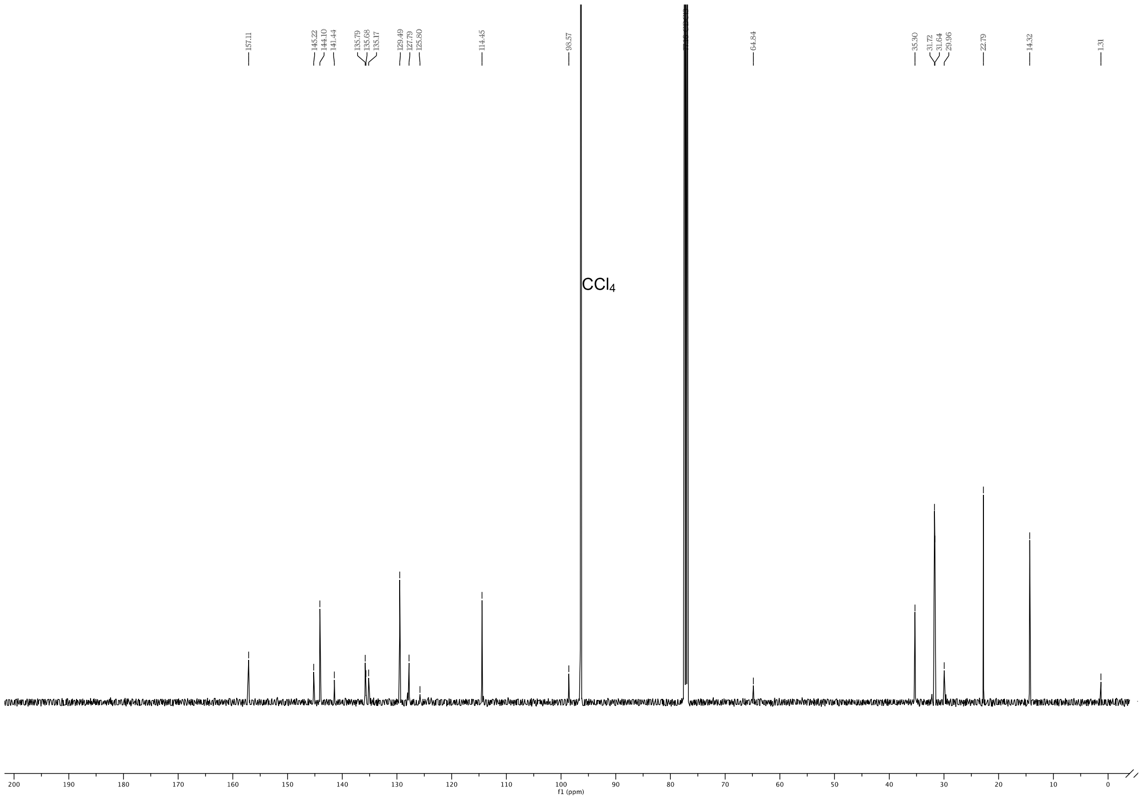

*Figure S65: ¹³C{¹H}-NMR spectrum of tetrahedral cage **Tc** (126 MHz, CDCl₃).*

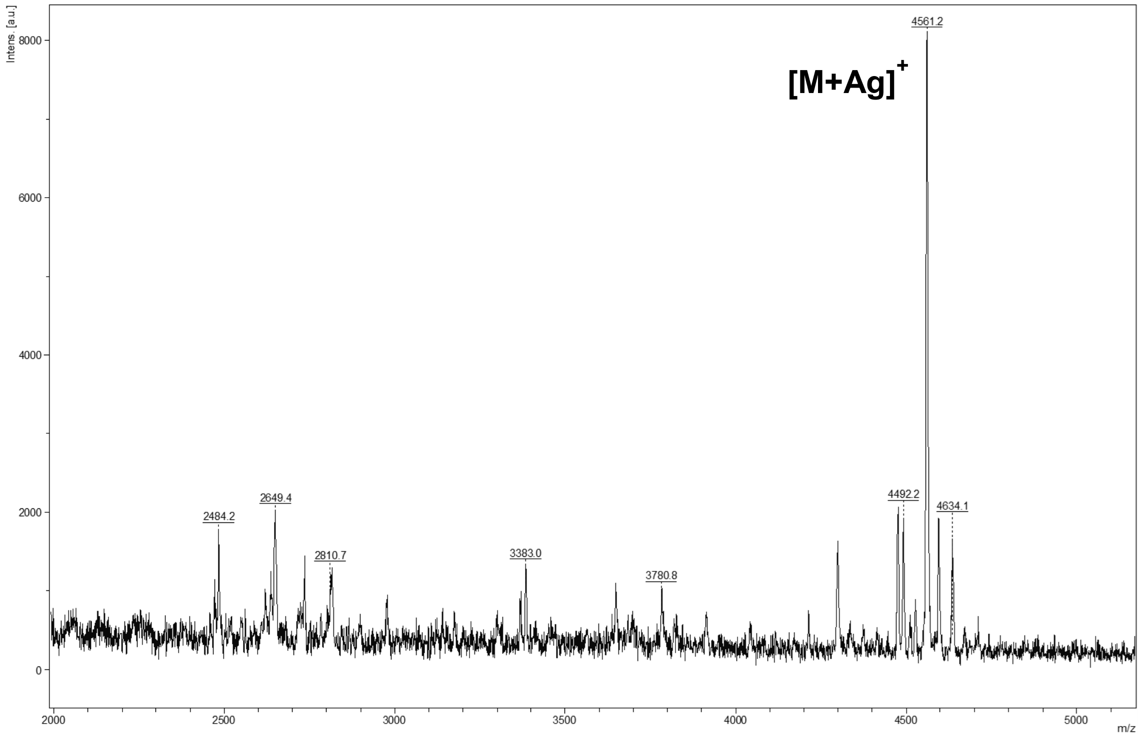

*Figure S66: MALDI-TOF spectrum of tetrahedral cage **Tc**.*

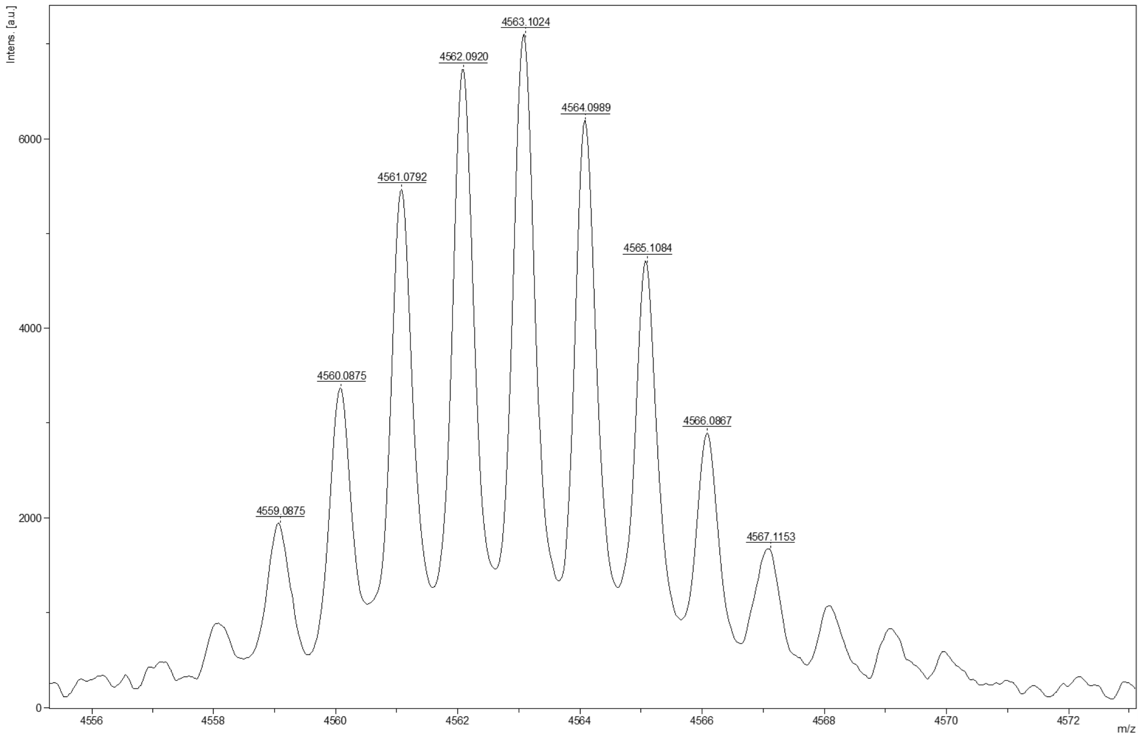

*Figure S67: HR-MALDI isotopic pattern of tetrahedral cage **Tc**.*

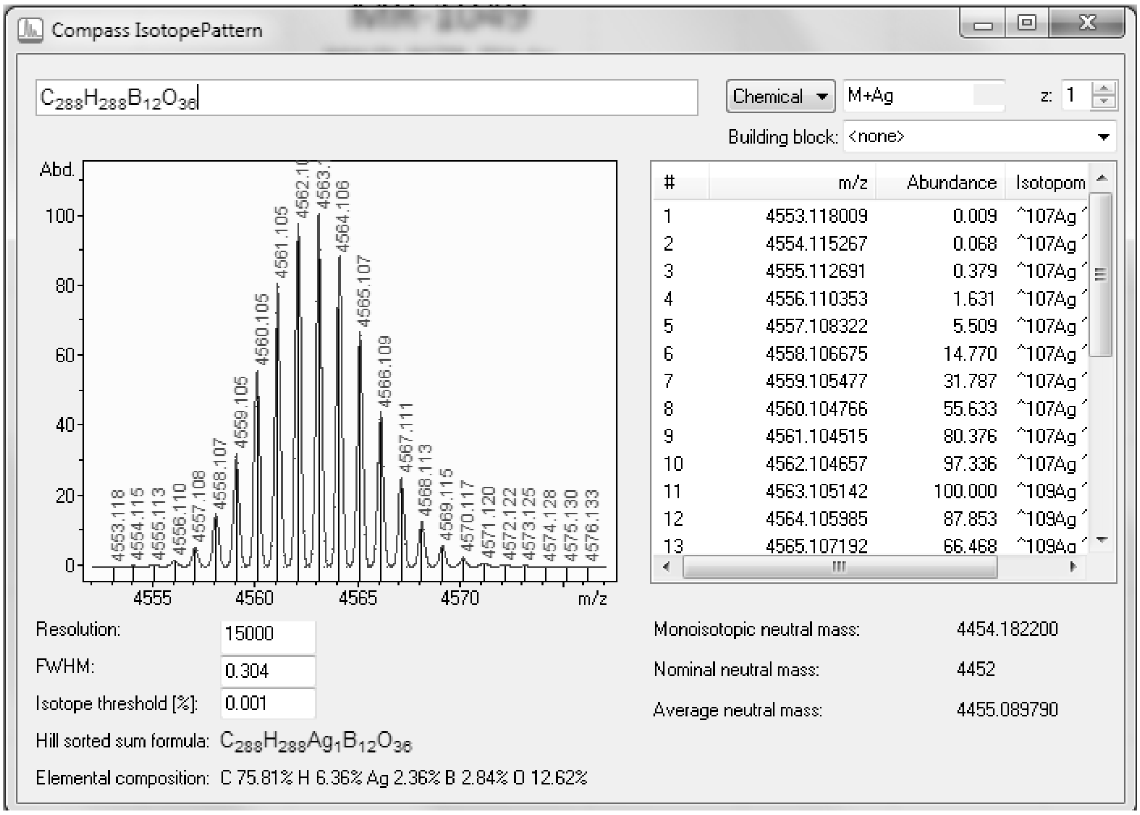

*Figure S68: Simulated HR-MALDI isotopic pattern of tetrahedral cage **Tc**.*

Tetrahedral Cage Td

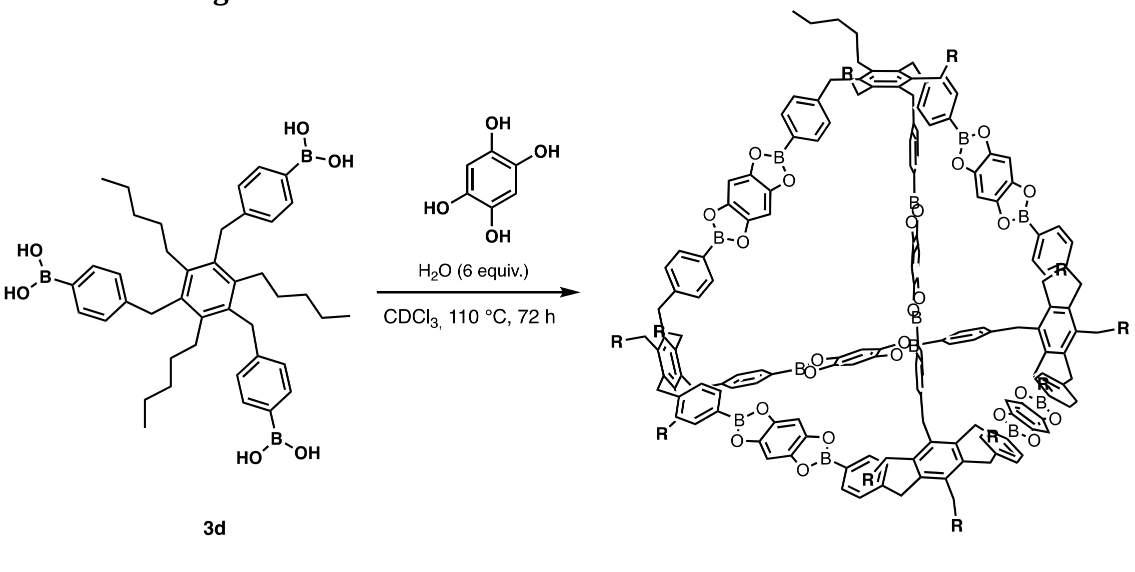

Tetrahedral cage (Td). General procedure A was applied to triboronic acid precursor **3d** (10.2 mg, 10.1 µmol, 1.0 equiv.), benzene-1,2,4,5-tetraol (**THB**) (2.16 mg, 15.2 µmol, 1.5 equiv.) using 2.0 mL anhydrous $CDCl_3$ (0.005 M) with H_2O (1.1 µL, 60.7 µmol, 6.0 equiv.). Clean cage formation could not be observed even after 7 days. The ^{1}H-NMR spectrum showed a complex spectrum that was attributed to the formation of unsymmetrical products and the coexistence of different species. Nonetheless, MALDI-MS showed the presence of **Td** although as a minor byproduct. Integration of the signals that we attributed to **Td** showed that yields never exceeded 24 %. (See Chapter 6 for details).
HRMS (MALDI+, DCTB, AgTFA) *m/z* calcd. for $C_{204}H_{216}B_{12}O_{24}Ag$ $[M+Ag]^+$ 3298.5882, found 3289.5908.

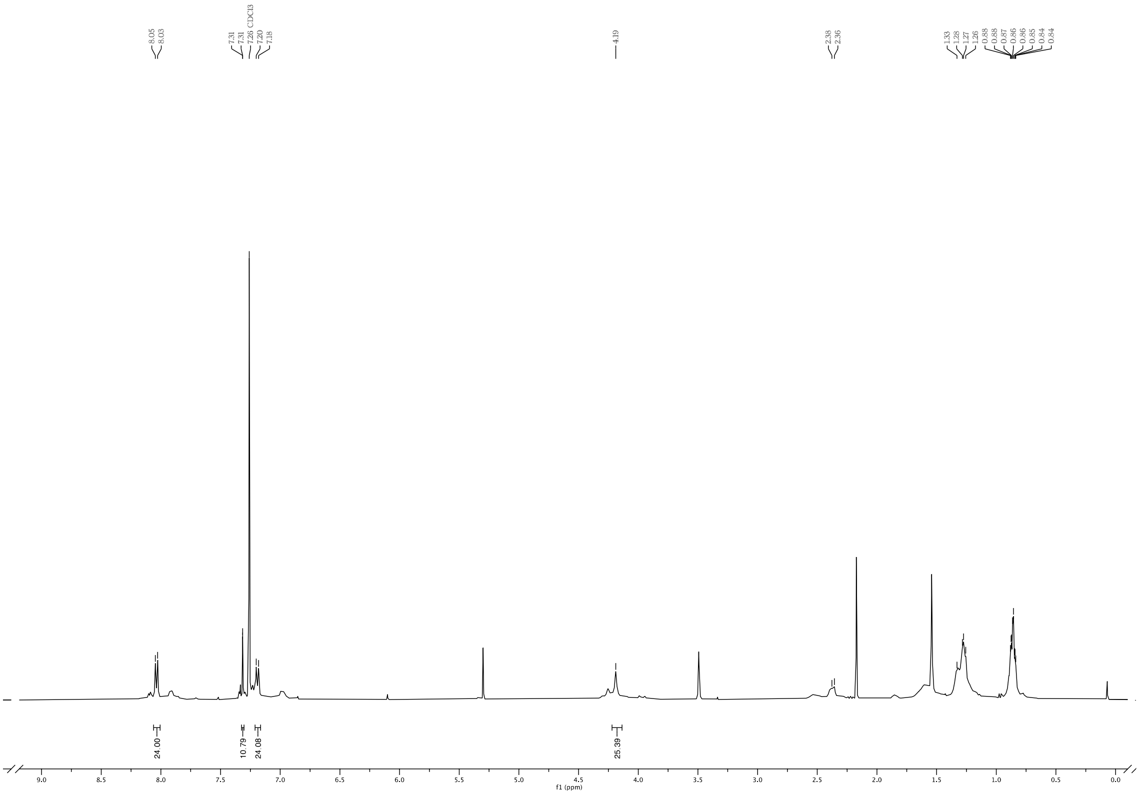

*Figure S69: ^{1}H-NMR spectrum of the partial formation of tetrahedral cage **Td** (500 MHz, $CDCl_3$).*

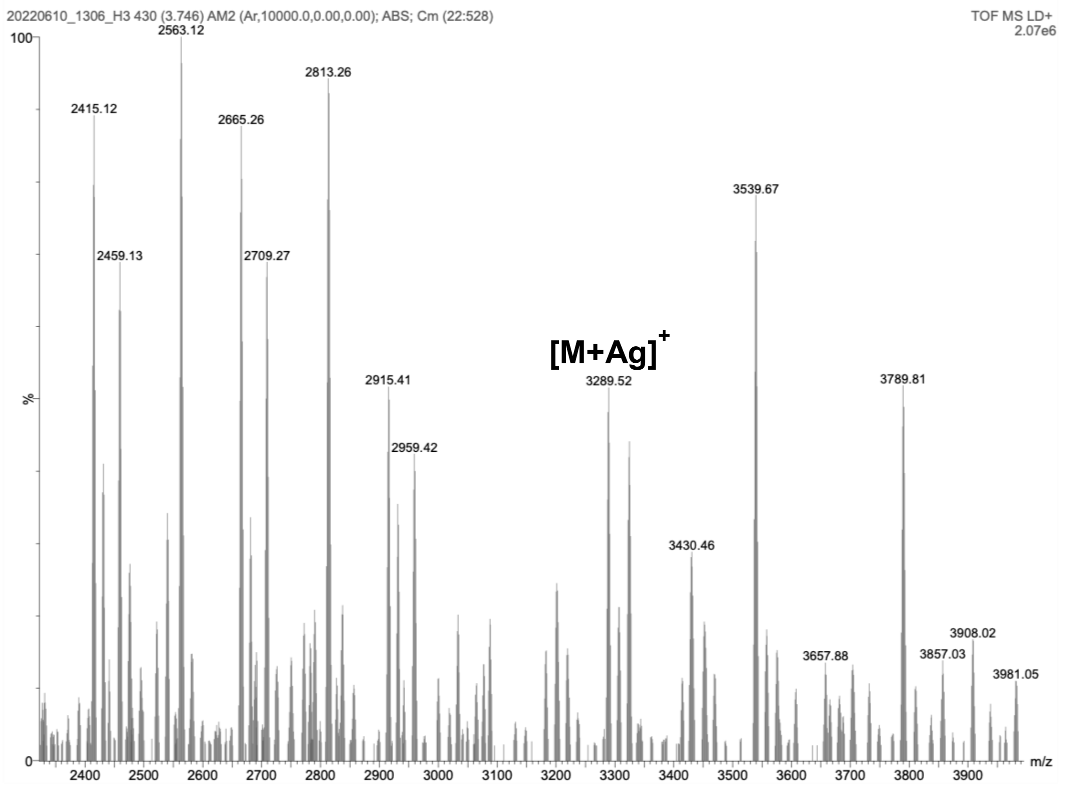

*Figure S70: MALDI-TOF spectrum of tetrahedral cage **Td**.*

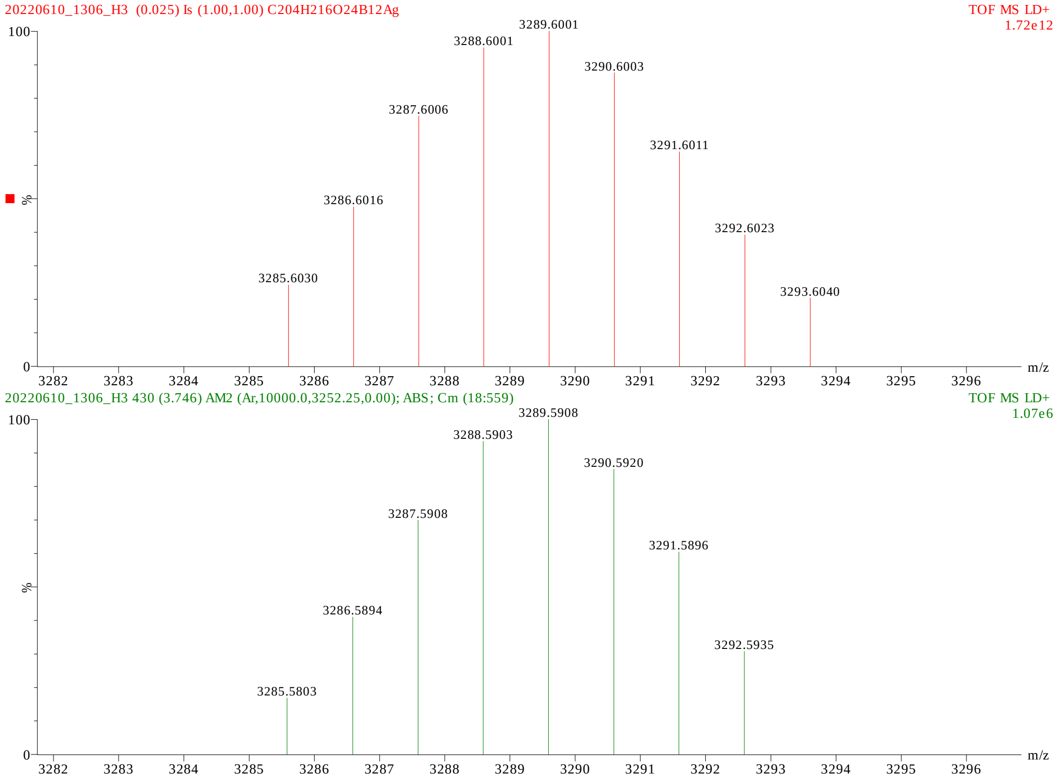

*Figure S71: Simulated (red) and measured (green) HR-MALDI-MS spectrum of tetrahedral cage **Td**.*

Tetrahedral Cage Te

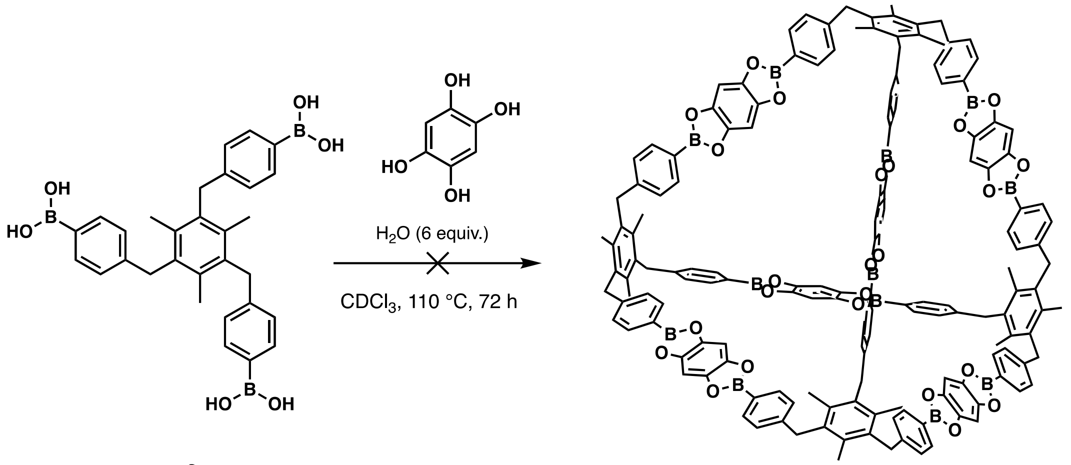

3e

Te

Tetrahedral cage (Te). General procedure A was applied to triboronic acid precursor **3e** (9.70 mg, 18.6 µmol, 1.0 equiv.), benzene-1,2,4,5-tetraol (**THB**) (3.96 mg, 27.9 µmol, 1.5 equiv.) using 3.7 mL anhydrous $CDCl_3$ (0.005 M) with H_2O (2.0 µL, 111.5 µmol, 6.0 equiv.). Cage formation **could not be observed** even after 7 days.

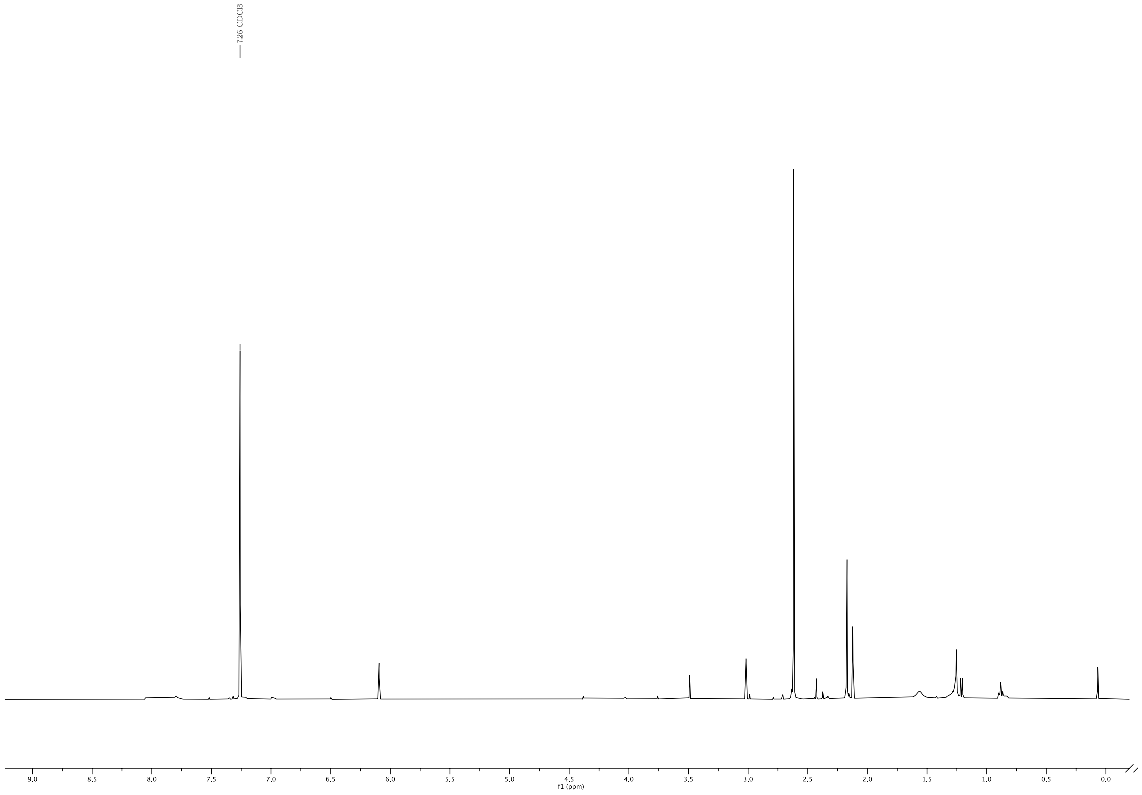

*Figure S72: ^{1}H-NMR spectrum of the trial for the formation of cage **Te** (500 MHz, $CDCl_3$).*

Tetrahedral Cage Tf

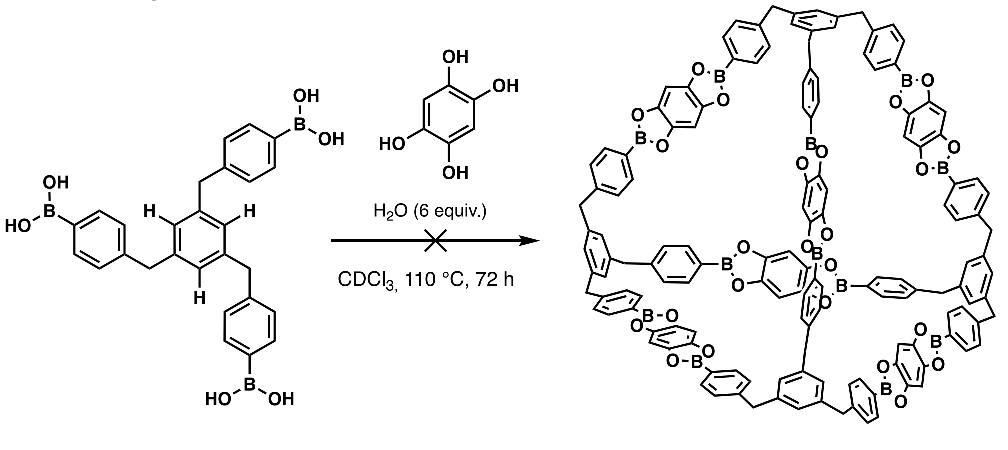

3f

Tf

Tetrahedral cage (Tf). General procedure A was applied to triboronic acid precursor **3f** (9.60 mg, 20.0 µmol, 1.0 equiv.), benzene-1,2,4,5-tetraol (**THB**) (4.26 mg, 30.0 µmol, 1.5 equiv.) using 4.0 mL anhydrous $CDCl_3$ (0.005 M) with H_2O (2.2 µL, 120 µmol, 6.0 equiv.). Cage formation **could not be observed** even after 7 days.

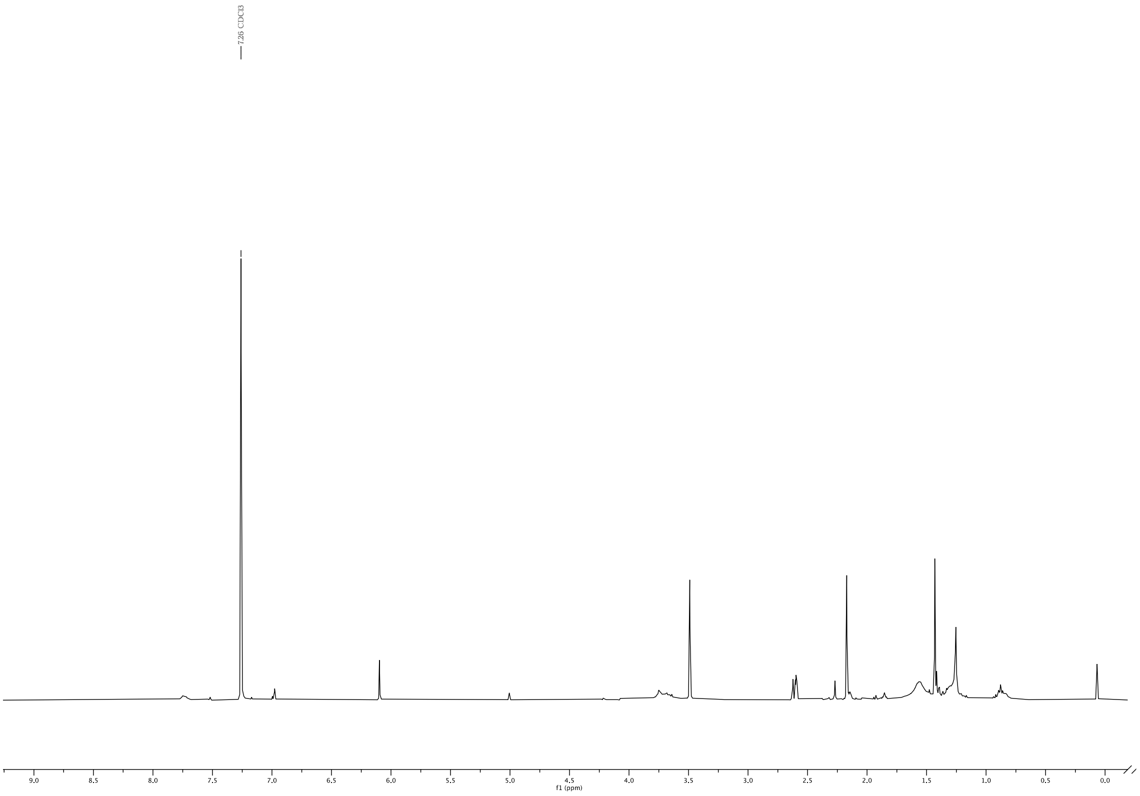

*Figure S73: ^{1}H-NMR spectrum of the trial for the formation of cage **Tf** (500 MHz, $CDCl_3$).*

6. Yield quantification

Yields were determined[6] by using a 0.5 mL aliquot directly extracted from the reaction mixture and addition of 9.47 µmol of 1,1,2,2-tetrachloroethane (TCE) as a stock solution in $CDCl_3$ (100 µL, 94.7 mM). A semi-quantitative ^{1}H-NMR spectrum was recorded using calibrated 90 degrees pulses and relaxation delays of 30 s with a total of 32 scans.
Four product signals were chosen were no evident overlapping with other signals was observed and integrated in relation to the TCE signal (the integral of the $C_2Cl_4\mathit{H}_2$ signal was set to a value of two). For each of the four product signals the yield (y) was determined and the average of all signals gave the yield (Y) of the reaction with its respective standard deviation (ΔY). The final yield $\bar{Y}$ for the formation of each cages was determined by performing three independent reactions and averaging their yields Y. The final error ($\Delta\bar{Y}$) was calculated by performing an error propagation of the standard deviation of Y (ΔY) into the three average reactions as follows:

$$\Delta\bar{Y} = \sqrt[2]{\frac{1}{9}(\Delta Y_1)^2 + \frac{1}{9}(\Delta Y_2)^2 + \frac{1}{9}(\Delta Y_3)^2}$$

where ΔY_x is the standard deviation of the yields y obtained from the individual ^{1}H-NMR signals of the product and the numbers 1,2,3 indicate the three independent reactions.

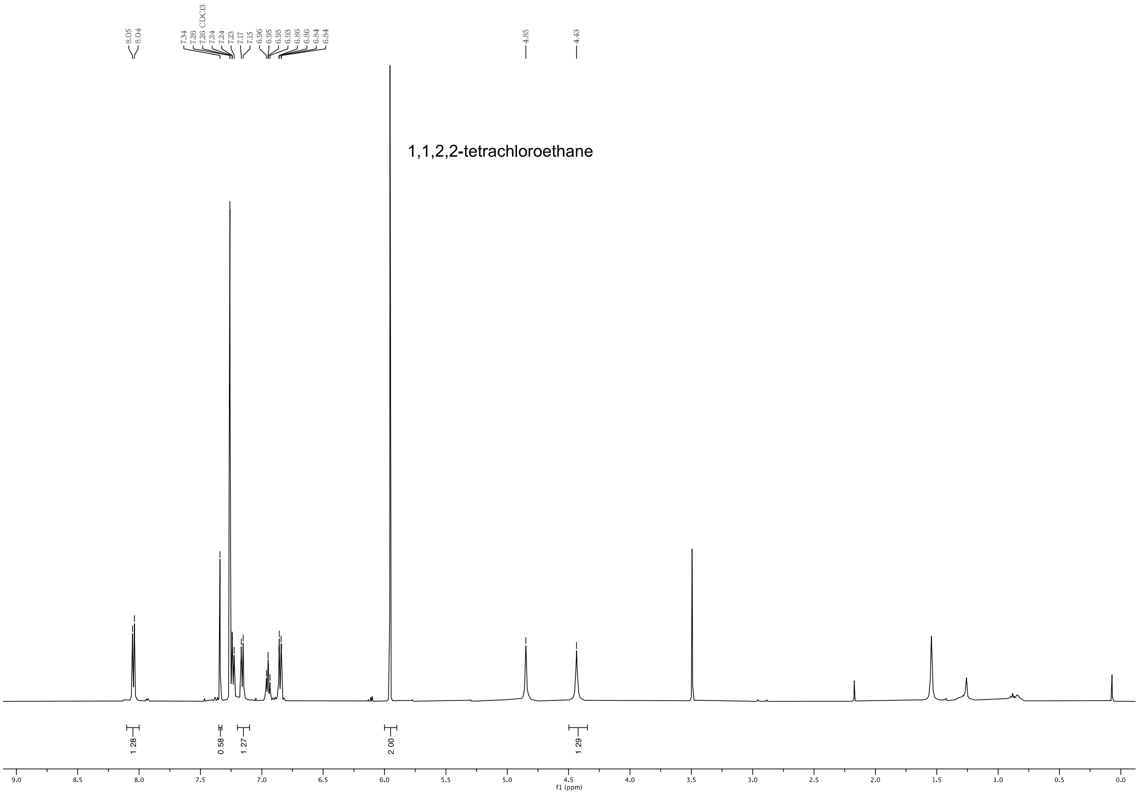

*Figure 74: Representative ¹H-NMR spectrum for the yield quantification of **Ta** using 1,1,2,2-tetrachloroethane as the internal standard (500 MHz, $CDCl_3$).*

*Table S1: Overview over yield determination for tetrahedral cage **Ta** based on semi-quantitative ¹H-NMR measurements using TCE as the internal standard.*

Reaction	Signal [ppm]	Integral (I)	Nuclei (N)	I/N	$n_{aliquot}$[1] [mmol]	n_{tot}[2] [mmol]	$n_{100\%}$[3] [mmol]	y [%]	ΔY [%]
**1**	8.0	1.28	24	0.05333	0.00051	0.00192	0.00238	81	
	7.3	0.58	12	0.04833	0.00046	0.00174	0.00238	73	
	7.2	1.27	24	0.05292	0.00050	0.00190	0.00238	80	
	4.4	1.29	24	0.05375	0.00051	0.00193	0.00238	81	
							Y_1:	79	3.8
**2**	8.0	1.27	24	0.05292	0.00050	0.00221	0.00276	80	
	7.3	0.61	12	0.05083	0.00048	0.00212	0.00276	77	
	7.2	1.33	24	0.05542	0.00052	0.00231	0.00276	84	
	4.4	1.23	24	0.05125	0.00049	0.00214	0.00276	77	
							Y_2:	79	3.1
**3**	8.0	1.38	24	0.05750	0.00054	0.00196	0.00225	87	
	7.3	0.63	12	0.05250	0.00050	0.00179	0.00225	80	
	7.2	1.27	24	0.05292	0.00050	0.00180	0.00225	80	
	4.4	1.34	24	0.05583	0.00053	0.00190	0.00225	85	
							Y_3:	83	3.6
							**overall**	**Ȳ [%]**	**ΔȲ [%]**
								**80**	**2.0**
**At 0.01 M concentration**									
	8.05	2.27	24	0.09458	0.00090	0.00358	0.005	72	
	7.34	1.06	12	0.08833	0.00084	0.00335	0.005	67	
	7.16	2.23	24	0.09292	0.00088	0.00352	0.005	70	
	4.43	2.61	24	0.10875	0.00103	0.00412	0.005	82	
							**$Y_{0.01M}$:**	**73**	**6.7**

[1] $n_{aliquot}$ is calculated by multiplying the moles of added TCE by the I/N ration and defines the moles of product in the 0.5 mL aliquot used for the quantification. [2] n_{tot} defines the moles of the product in the whole reaction mixture. [3] $n_{100\%}$ is the moles of product for a theoretical yield of 100%.

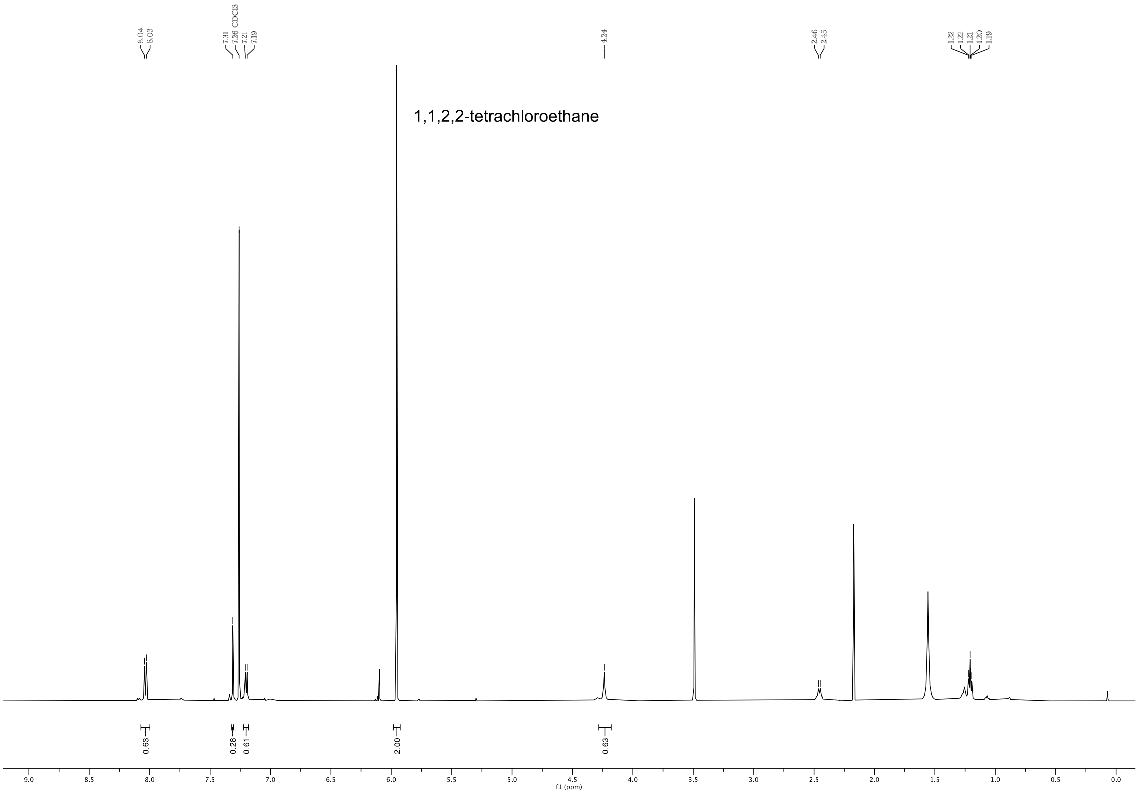

*Figure 75: Representative ^{1}H-NMR spectrum for the yield quantification of **Tb** using 1,1,2,2-tetrachloroethane as the internal standard (500 MHz, $CDCl_3$).*

*Table S2: Overview over yield determination for tetrahedral cage **Tb** based on semi-quantitative ^{1}H-NMR measurements using TCE as the internal standard.*

Reaction	Signal [ppm]	Integral (I)	Nuclei (N)	I/N	$n_{aliquot}$[1] [mmol]	n_{tot}[2] [mmol]	$n_{100\%}$[3] [mmol]	y [%]	ΔY [%]
**1**	8.0	0.63	24	0.02625	0.00025	0.00134	0.00337	40	
	7.3	0.28	12	0.02333	0.00022	0.00119	0.00337	35	
	7.2	0.61	24	0.02542	0.00024	0.00130	0.00337	39	
	4.4	0.63	24	0.02625	0.00025	0.00134	0.00337	40	
							Y_1:	38	2.1
**2**	8.0	0.57	24	0.02375	0.00022	0.00112	0.00306	37	
	7.3	0.28	12	0.02333	0.00022	0.00111	0.00306	36	
	7.2	0.5	24	0.02083	0.00020	0.00099	0.00306	32	
	4.4	0.48	24	0.02000	0.00019	0.00095	0.00306	31	
							Y_2:	34	2.9
**3**	8.0	0.55	24	0.02292	0.00022	0.00143	0.00412	35	
	7.3	0.25	12	0.02083	0.00020	0.00130	0.00412	32	
	7.2	0.56	24	0.02333	0.00022	0.00146	0.00412	35	
	4.4	0.55	24	0.02292	0.00022	0.00143	0.00412	35	
							Y_3:	34	1.7
							**overall**	**Ȳ [%]**	**ΔȲ [%]**
								**36**	**1.3**
**At 0.01 M concentration**									
	8.03	0.78	24	0.03250	0.00031	0.00123	0.005	25	
	7.31	0.46	12	0.03833	0.00036	0.00145	0.005	29	
	7.2	0.8	24	0.03333	0.00032	0.00126	0.005	25	
	4.42	0.88	24	0.03667	0.00035	0.00139	0.005	28	
							**$Y_{0.01M}$:**	**27**	**2.1**

[1] $n_{aliquot}$ is calculated by multiplying the moles of added TCE by the I/N ration and defines the moles of product in the 0.5 ml aliquot used for the quantification. [2] n_{tot} defines the moles of the product in the whole reaction mixture. [3] $n_{100\%}$ is the moles of product for a theoretical yield of 100%.

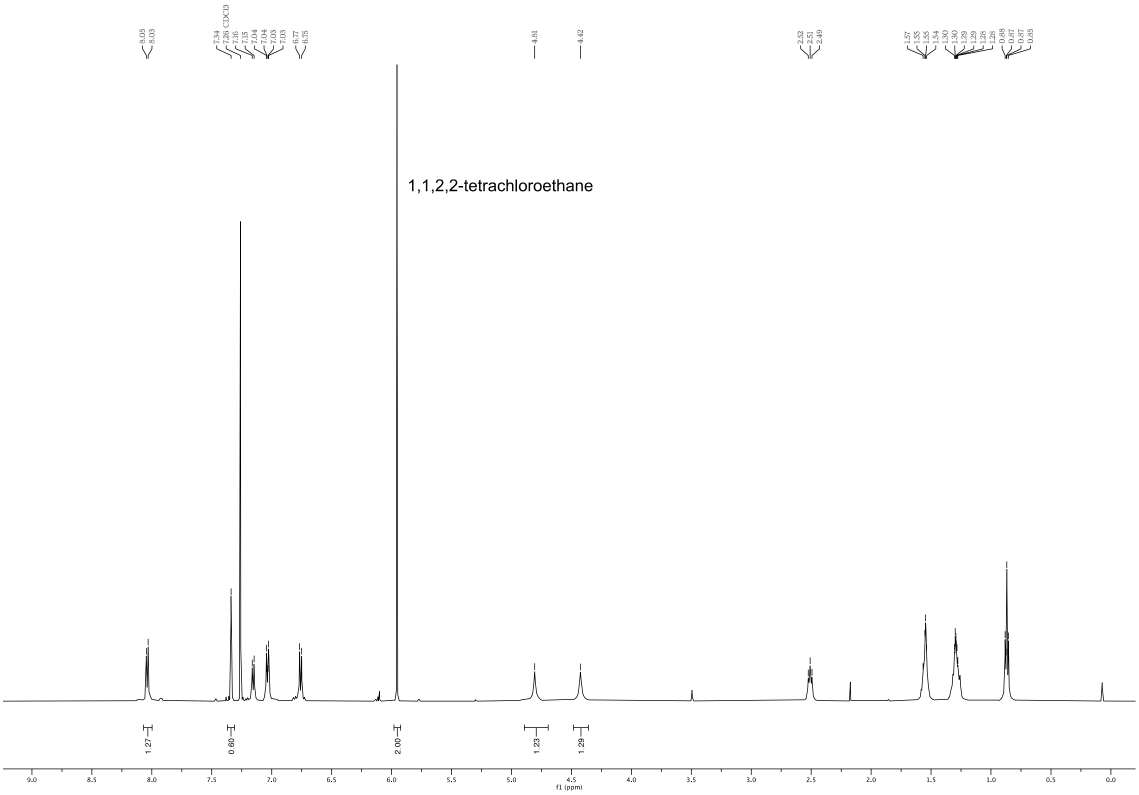

*Figure 76: Representative ^{1}H-NMR spectrum for the yield quantification of **Tc** using 1,1,2,2-tetrachloroethane as the internal standard (500 MHz, $CDCl_3$).*

*Table S3: Overview over yield determination for tetrahedral cage **Tc** based on semi- quantitative ^{1}H-NMR measurements using TCE as the internal standard.*

Reaction	Signal [ppm]	Integral (I)	Nuclei (N)	I/N	$n_{aliquot}$[1] [mmol]	n_{tot}[2] [mmol]	$n_{100\%}$[3] [mmol]	y [%]	ΔY [%]
**1**	8.0	1.27	24	0.05292	0.00050	0.00185	0.00230	80	
	7.3	0.60	12	0.05000	0.00047	0.00175	0.00230	76	
	4.8	1.23	24	0.05125	0.00049	0.00179	0.00230	78	
	4.4	1.29	24	0.05375	0.00051	0.00188	0.00230	82	
							Y_1:	79	2.6
**2**	8.0	1.35	24	0.05625	0.00053	0.00234	0.00268	87	
	7.3	0.63	12	0.05250	0.00050	0.00219	0.00268	82	
	4.8	1.21	24	0.05042	0.00048	0.00210	0.00268	78	
	4.4	1.35	24	0.05625	0.00053	0.00234	0.00268	87	
							Y_2:	84	4.5
**3**	8.0	1.35	24	0.05625	0.00053	0.00234	0.00270	87	
	7.3	0.62	12	0.05167	0.00049	0.00215	0.00270	80	
	4.8	1.17	24	0.04875	0.00046	0.00203	0.00270	76	
	4.4	1.3	24	0.05417	0.00051	0.00226	0.00270	84	
							Y_3:	82	5.0
							**overall**	**$\bar{Y}$ [%]**	**$\Delta\bar{Y}$ [%]**
								**82**	**2.4**

[1] $n_{aliquot}$ is calculated by multiplying the moles of added TCE by the I/N ration and defines the moles of product in the 0.5 ml aliquot used for the quantification. [2] n_{tot} defines the moles of the product in the whole reaction mixture. [3] $n_{100\%}$ is the moles of product for a theoretical yield of 100%.

7. DOSY Experiments and Volume Approximation

Due to low the solubility and the possibility to form boroxine aggregates of the boronic acid tripods in $CDCl_3$, their respective B-protected precursors were used. To determine the final diffusion coefficient D, only signals that correspond to the species in question were considered. Additionally, in several cases some product signals were not considered when there was ^{1}H-NMR chemical shift overlapping with others signals that do not belong to the studied product (such as water or grease). D values obtained from averaging all molecule signals were used for further calculation of radius and volumes. The volume was determined using the Stokes-Einstein equation and assuming a spherical particle shape:

$$D = \frac{k_B T}{6\pi\eta r}$$

The molecular radius was calculated by using:

$$r = \frac{k_B T}{6\pi\eta}\frac{1}{D}$$

And the molecular volume by using:

$$V = \frac{4}{3}\pi r^3$$

With
r: radius of the sphere particle
V: volume of the sphere particle
D: diffusion coefficient; [D] = m^2/s
k_B: Boltzmann constant, $k_B = 1.38 \times 10^{-23}$ J/K
T: temperature; T = 298 K
η: dynamic viscosity; η ($CDCl_3$) = 5.28×10^{-4} Ns/m^2

There are two main different error sources in the calculation of the D values. One derives from the linear regression used to determine D from the area below the signal (area integration) decay of the DOSY measurement, which lies in the order of magnitude 10^{-13} m^2/sec. The other, derives from averaging the different values for D (one for each signal of the molecule) using the standard deviation function "STDEV.S" of Microsoft Excel and lies in the order of magnitude 10^{-12} m^2/sec. As the second error is remarkably bigger than the error from the linear regression, only the latter is considered and defined as ΔD. As the radius r and volume V are functions of D, there errors can be calculated using error propagation as follows:

$$\Delta r(D) = \left|\frac{\partial r}{\partial D}\right| \Delta D = \frac{k_B T}{6\pi\eta D^2}\Delta D$$

and

$$\Delta V(r) = \left|\frac{\partial V}{\partial r}\right| \Delta r = 4r^2\pi\,\Delta r$$

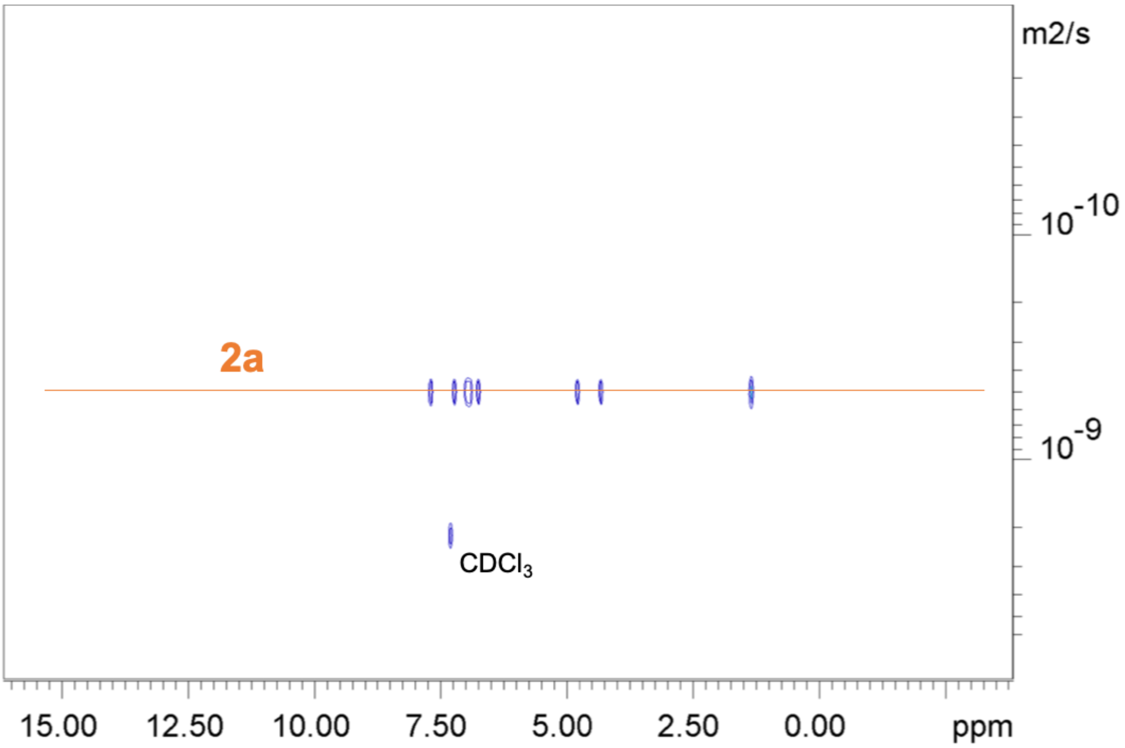

*Figure S77: ^{1}H-DOSY NMR spectrum of precursor **2a** (500 MHz, $CDCl_3$).*

*Table S4: Diffusion constants and chemical shifts for each ^{1}H-NMR DOSY signal corresponding to precursor **2a**.*

Precursor 2a	Chemical shift [ppm]	D [m^2/s]
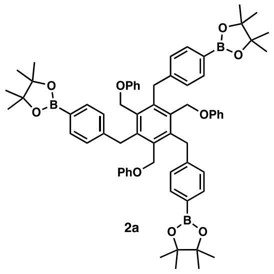	7.68	5.11 x 10^{-10}
	7.20	5.14 x 10^{-10}
	6.99	5.12 x 10^{-10}
	6.91	5.15 x 10^{-10}
	6.75	5.12 x 10^{-10}
	4.81	5.12 x 10^{-10}
	4.32	5.15 x 10^{-10}
	1.34	5.14 x 10^{-10}
	Average:	**5.13 x 10^{-10}**
	Error ΔD:	**1.55 x 10^{-12}**

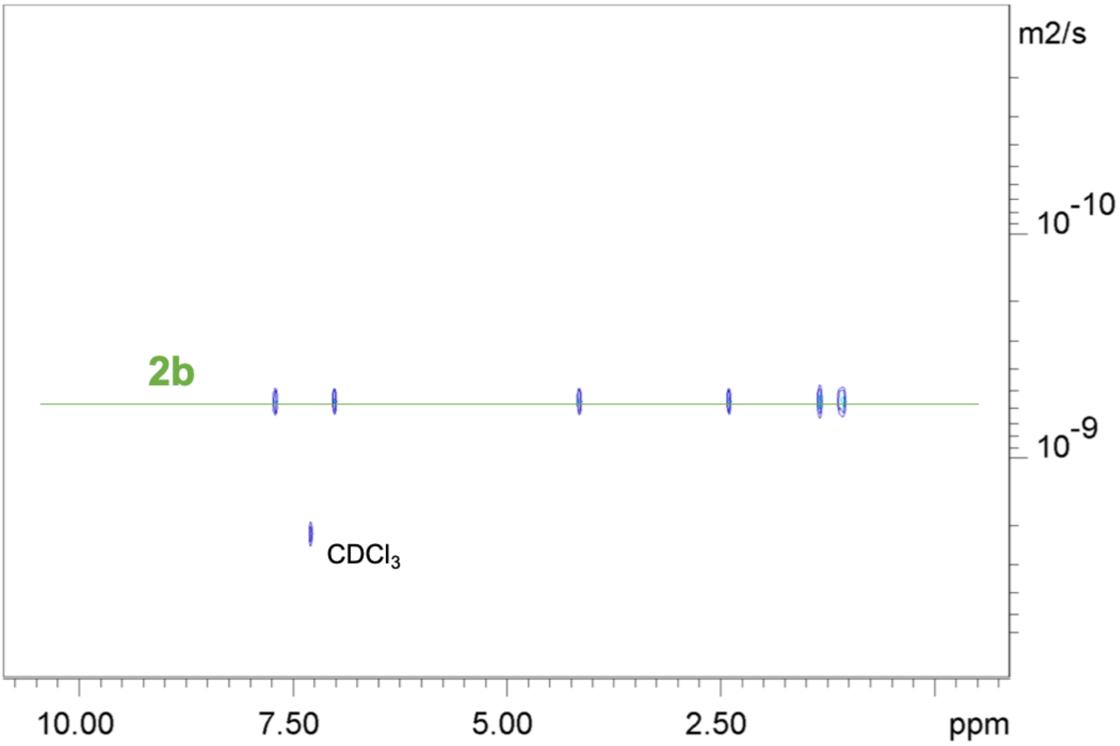

*Figure S78: ^{1}H-DOSY NMR spectrum of precursor **2b** (500 MHz, $CDCl_3$).*

*Table S5: Diffusion constants and chemical shifts for each ^{1}H-NMR DOSY signal corresponding to precursor **2b**.*

**Precursor 2b**	**Chemical shift [ppm]**	**D [m^2/s]**
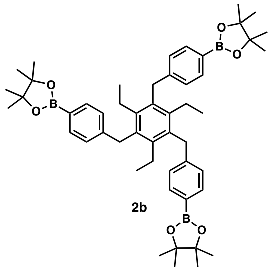	7.71	5.74×10^{-10}
	7.02	5.71×10^{-10}
	4.14	5.68×10^{-10}
	2.43	5.72×10^{-10}
	1.35	5.67×10^{-10}
	1.10	5.71×10^{-10}
	Average:	$\mathbf{5.71 \times 10^{-10}}$
	Error ΔD:	$\mathbf{2.59 \times 10^{-12}}$

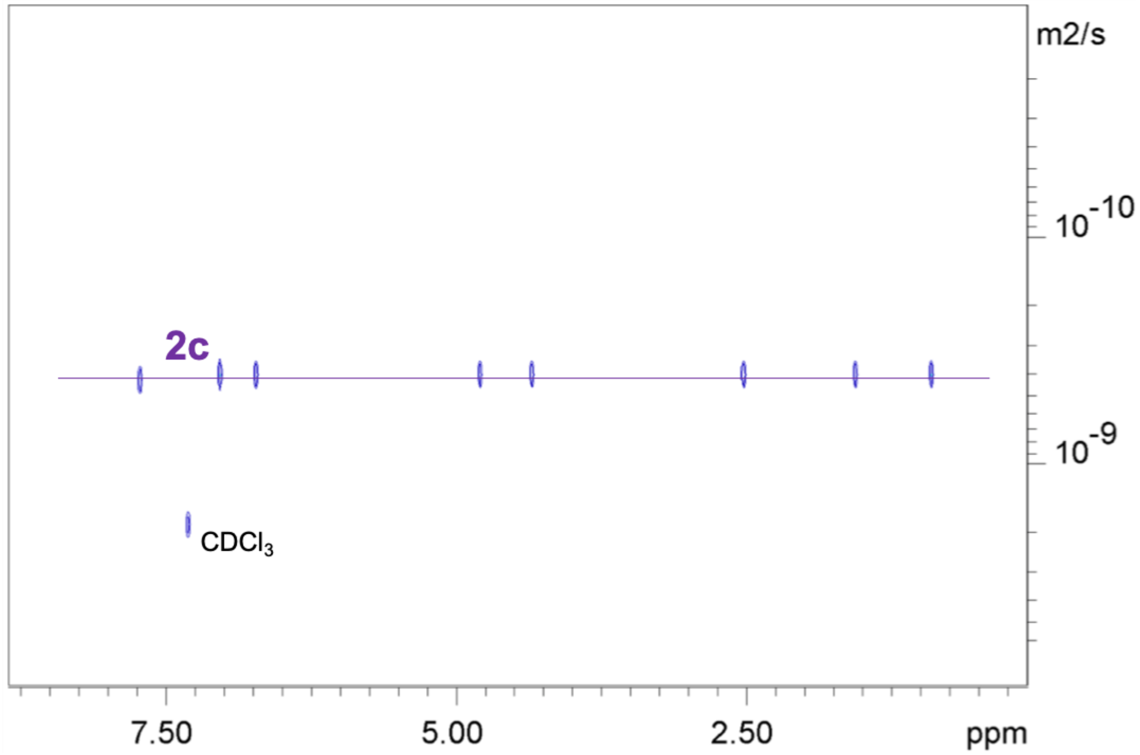

*Figure S79: ^{1}H-DOSY NMR spectrum of precursor **2c** (500 MHz, $CDCl_3$).*

*Table S6: Diffusion constants and chemical shifts for each ^{1}H-NMR DOSY signal corresponding to precursor **2c**.*

Precursor 2c	Chemical shift [ppm]	D [m^2/s]
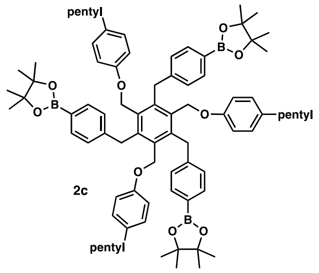	7.71	4.19 x 10^{-10}
	7.02	4.06 x 10^{-10}
	6.70	4.10 x 10^{-10}
	4.80	4.06 x 10^{-10}
	4.34	4.08 x 10^{-10}
	2.54	4.05 x 10^{-10}
	1.58	4.12 x 10^{-10}
	0.91	4.09 x 10^{-10}
	Average:	**4.09 x 10^{-10}**
	Error ΔD:	**4.53 x 10^{-12}**

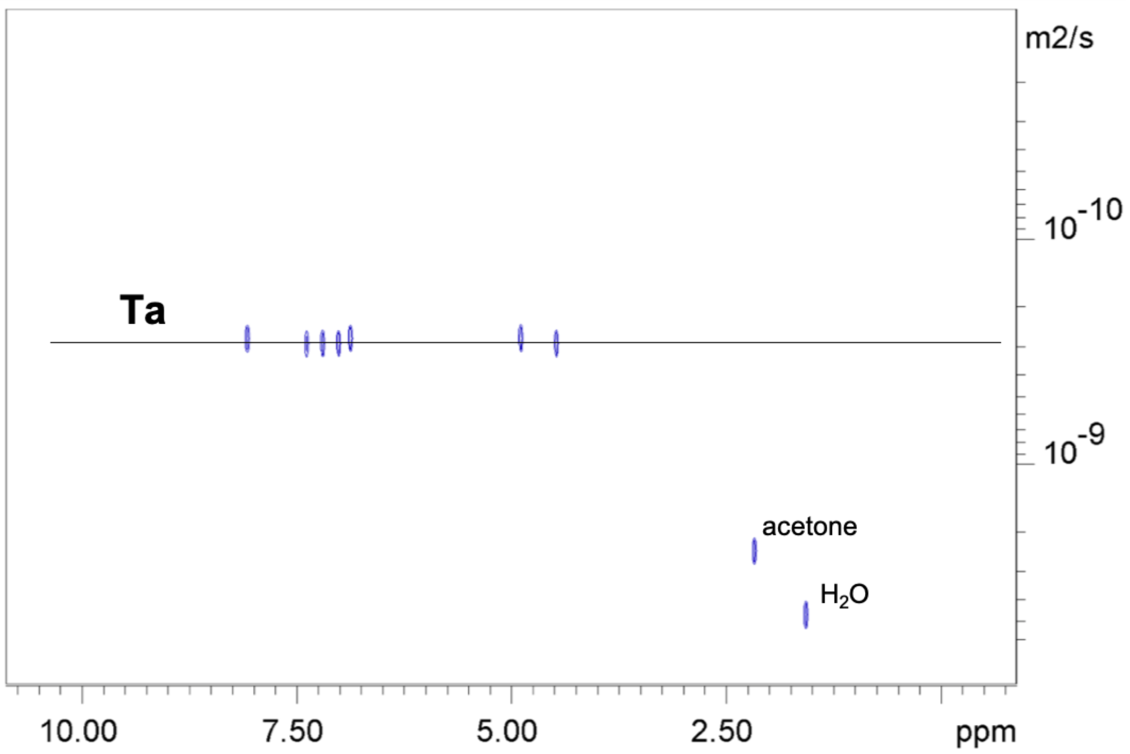

*Figure S80: ^{1}H-DOSY NMR spectrum of cage **Ta** (500 MHz, $CDCl_3$).*

*Table S7: Diffusion constants and chemical shifts for each ^{1}H-NMR DOSY signal corresponding to cage **Ta**.*

Tetrahedron Ta	Chemical shift [ppm]	D [m^2/s]
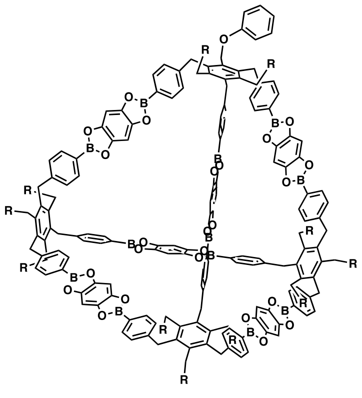	8.07	2.81 x 10^{-10}
	7.37	2.84 x 10^{-10}
	7.18	2.84 x 10^{-10}
	6.98	2.86 x 10^{-10}
	6.87	2.81 x 10^{-10}
	4.87	2.81 x 10^{-10}
	4.46	2.85 x 10^{-10}
**Ta**	Average:	**2.83 x 10^{-10}**
R = OPh	Error ΔD:	**2.12 x 10^{-12}**

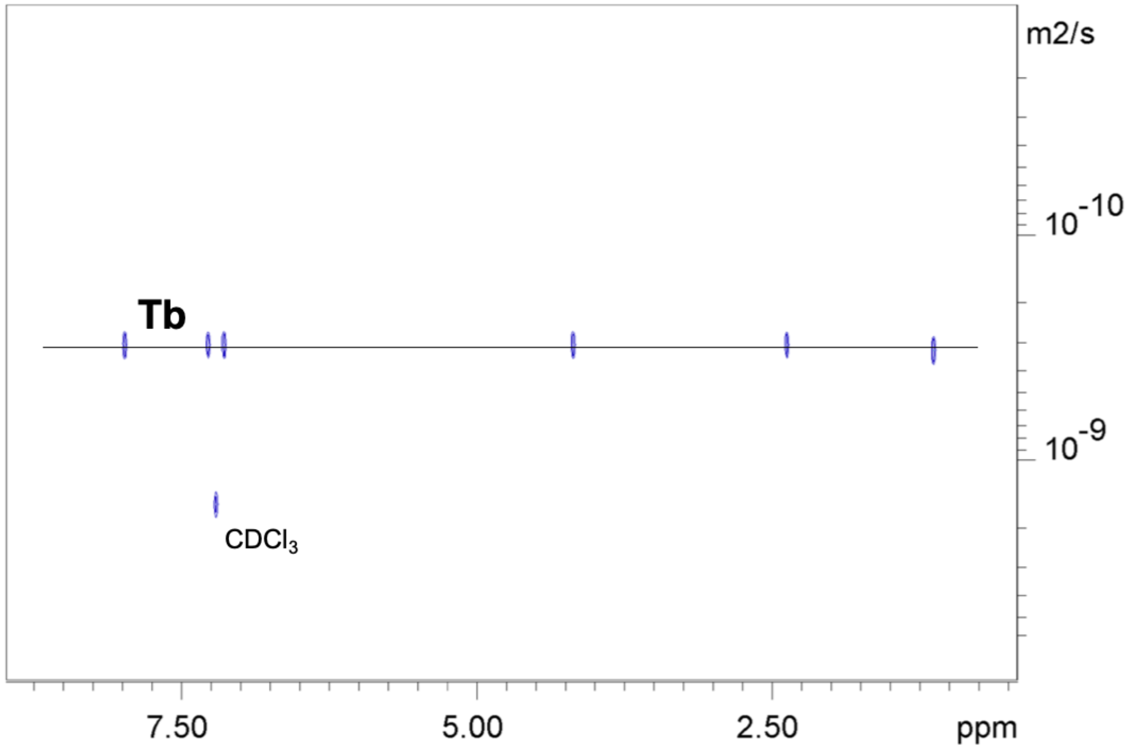

*Figure S81: ^{1}H-DOSY NMR spectrum of cage **Tb** (500 MHz, $CDCl_3$).*

*Table S8: Diffusion constants and chemical shifts for each ^{1}H-NMR DOSY signal corresponding to cage **Tb**.*

Tetrahedron Tb	Chemical shift [ppm]	D [m^2/s]
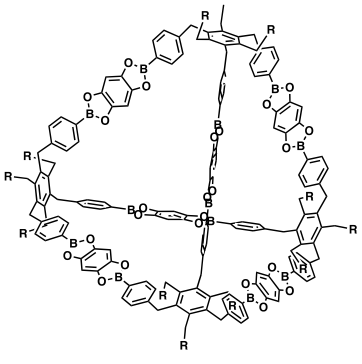 Tb R = CH_3	7.97	3.06×10^{-10}
	7.25	3.09×10^{-10}
	7.13	3.08×10^{-10}
	4.17	3.06×10^{-10}
	2.39	3.09×10^{-10}
	1.13	3.24×10^{-10}
	Average:	**3.10×10^{-10}**
	Error ΔD:	**6.83×10^{-12}**

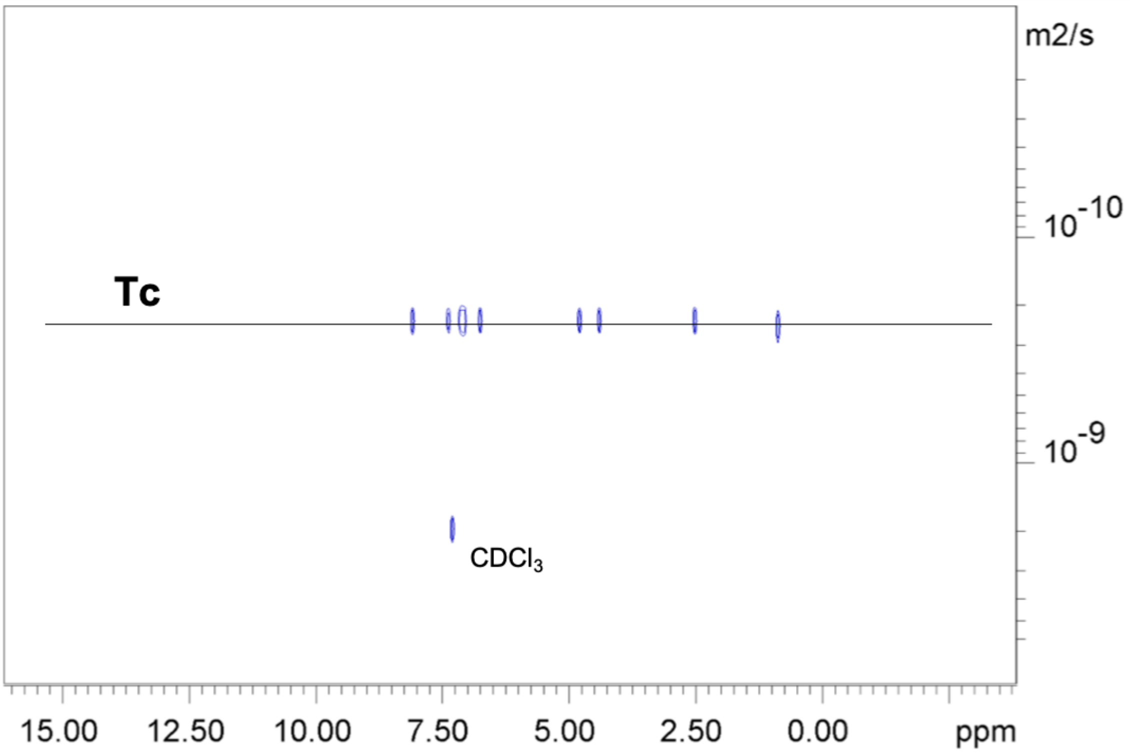

*Figure S82: ^{1}H-DOSY NMR spectrum of cage **Tc** (500 MHz, $CDCl_3$).*

*Table S9: Diffusion constants and chemical shifts for each ^{1}H-NMR DOSY signal corresponding to cage **Tc**.*

Tetrahedron Tc	Chemical shift [ppm]	D [m^2/s]
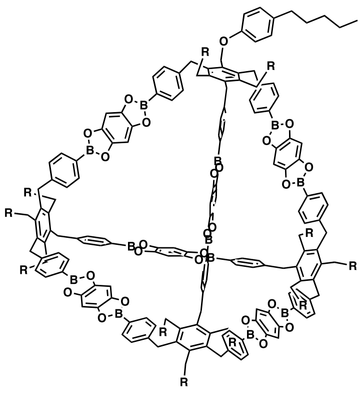	8.04	2.37 x 10^{-10}
	7.33	2.40 x 10^{-10}
	7.16	2.36 x 10^{-10}
	7.03	2.35 x 10^{-10}
	6.76	2.37 x 10^{-10}
	4.79	2.38 x 10^{-10}
	4.43	2.36 x 10^{-10}
	2.51	2.35 x 10^{-10}
**Tc** R = O-4-C_6H_6-pentyl	Average:	**2.36 x 10^{-10}**
	Error ΔD:	**2.33 x 10^{-12}**

After recording a DOSY ^{1}H-NMR for each species individually, a mixture of Bpin-precursor **2a** and the corresponding tetrahedral cage **Ta** was measured as a proof of concept. For this purpose, 0.25 mL of a Bpin-precursor solution and 0.25 mL of the corresponding tetrahedron solution (the concentrations were beforehand adjusted to roughly give an equimolar mixture) were added to an NMR-tube, agitated for several seconds and the ^{1}H-NMR DOSY was recorded. The so-obtained diffusion coefficients are in good agreement with the ones from the individual species.

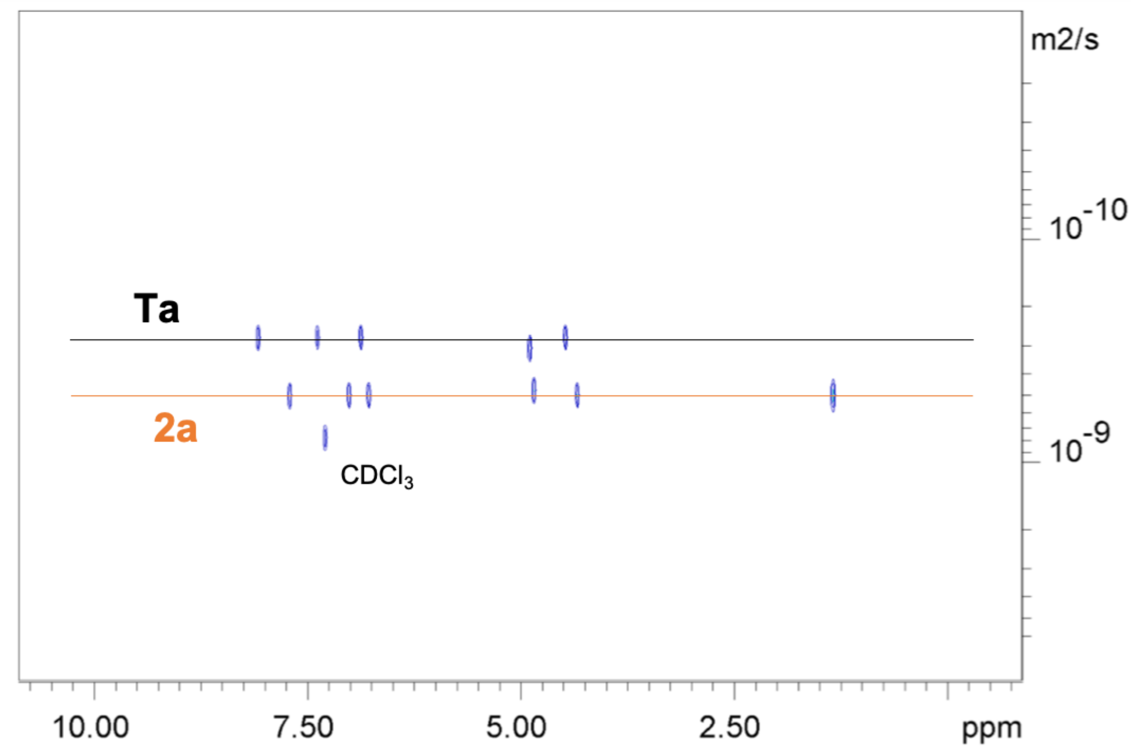

*Figure S83: ^{1}H-DOSY NMR spectrum of a mixture of cage **Ta** and precursor **2a**. Some signals corresponding to **Ta** and **2a** are omitted due to overlapping (500 MHz, $CDCl_3$).*

*Table S10: Diffusion constants and chemical shifts for each ^{1}H-NMR DOSY signal corresponding to a mixture of precursor **2a** and cage **Ta**.*

	**Chemical shift [ppm]**	**D [m^2/s]**
Cage **Ta**	8.06	2.73×10^{-10}
Precursor **2a**	7.70	5.03×10^{-10}
Cage **Ta**	7.36	2.74×10^{-10}
Precursor **2a**	7.02	4.98×10^{-10}
Cage **Ta**	6.86	2.81×10^{-10}
Precursor **2a**	6.78	4.93×10^{-10}
Cage **Ta**	4.89	3.12×10^{-10}
Precursor **2a**	4.82	4.79×10^{-10}
Cage **Ta**	4.46	2.80×10^{-10}
Precursor **2a**	4.45	4.93×10^{-10}

Precursor **2a**	1.36	5.13 x 10^{-10}
	Average **2a**	**4.97 x 10^{-10}**
	Error ΔD(**2a**):	**1.14 x 10^{-11}**
	Average **Ta**	**2.84 x 10^{-10}**
	Error ΔD(**Ta**):	**1.60 x 10^{-11}**

Table S11: Diffusion coefficients, radii, volumes and corresponding errors for each species determined separately.

	**D [10^{-10} m^2/s]**	**ΔD [10^{-10} m^2/s]**	**r [Å]**	**Δr [Å]**	**V [Å^3]**	**ΔV [Å^3]**
**2a**	5.13	0.02	8.06	0.02	2,193	19.9
**2b**	5.71	0.03	7.25	0.03	1,595	21.7
**2c**	4.09	0.05	10.1	0.11	4,318	143
**Ta**	2.83	0.02	14.6	0.11	13,050	293
**Tb**	3.10	0.07	13.3	0.29	9,911	655
**Tc**	2.36	0.02	17.5	0.17	22,429	662

Table S12: Diffusion coefficients, radii, volumes and corresponding errors from precursor-tetrahedron mixture.

	**D [10^{-10} m^2/s]**	**ΔD [10^{-10} m^2/s]**	**r [Å]**	**Δr [Å]**	**V [Å^3]**	**ΔV [Å^3]**
**2a**	4.97	0.11	8.33	0.19	2,420	166
**Ta**	2.84	0.16	14.6	0.82	12,932	2192

Table S13: Comparison radii and volumes derived from DOSY measurements and computational calculations of precursors and tetrahedral cages.

	**Based on ^{1}H-NMR DOSY**		**Based on computational calculations***	
	**r [Å]**	**V [Å^3]**	**r [Å]**	**V [Å^3]**
**2a**	8.06 ± 0.02	2,193 ± 19.9	7.95	2,105
**2b**	7.25 ± 0.03	1,595 ± 21.7	6.18	989
**2c**	10.1 ± 0.11	4,318 ± 143	10.72	5,160
**Ta**	14.6 ± 0.11	13,050 ± 293	13.98	11,445
**Tb**	13.3 ± 0.29	9,911 ± 664	12.35	7,890
**Tc**	17.5 ± 0.17	22,429 ± 662	14.55	12,903

*For radius/volume determination based on molecular modeling see Chapter 8.1.

8. Computational Calculations

8.1 Volume calculation of precursors and cages

Molecular models for the volume determination were obtained by molecular mechanics calculations using Schrodinger/Macromodel. To obtain the global minimum energy structure conformational searches was performed using a hybrid method that included MonteCarlo (MCMM) and Low Mode structure sampling and the OPLS4 force field. Extended nonbonded cutoff distances (a van der Waals cutoff of 8.0 Å and an electrostatic cutoff of 20.0 Å) were used. The lowest-energy structure found was used for further calculations. Approximate volumes were calculated based on the molecule diameters obtained from the molecular models. Due to the irregular shape of the structures, the diameter was calculated by mean averaging the shortest, the medium and the largest possible diameter for each molecule. For the tripods this means that the Bpin-Bpin, foot-foot and Bpin-foot distances were taken into account. For the tetrahedral cages, the foot-foot, foot-tetraol and tetraol-tetraol distance where considered. For all diameters, the outermost carbon-atom of the chain/ring was used as the start or end point of the measurement. The volume was then calculated analogously to DOSY measurements (see chapter 7). This certainly constitutes a rough approximation and only serves for qualitative comparison. However, the radius/volume obtained by this procedure are in good agreement with the results from the DOSY experiments. For the tripodal precursors, the corresponding Bpin-tripods (instead of the boronic acids) were used to ensure comparability with the values obtained from the DOSY-NMR experiments.

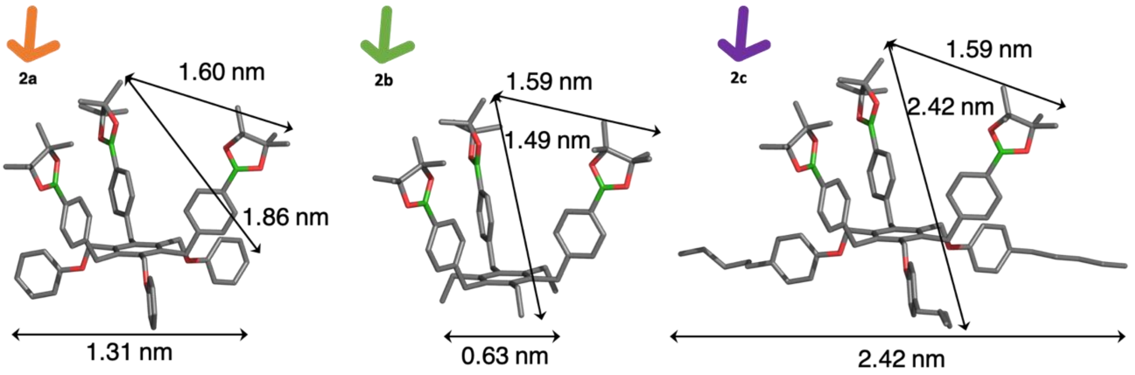

*Figure S84: Molecular models (OPLS4) of tripodal Bpin-precursors **2a**, **2b** and **2c**.*

*Table S14: Overview over volume calculation of Bpin-tripod precursors **2a**, **2b** and **2c** based on molecular modeling.*

precursor		d [nm]	r [nm]	V [nm^3]
2a	1	1.60	0.800	
	2	1.31	0.655	
	3	1.86	0.930	
	**average**		**0.795**	**2.105**
2b	1	1.59	0.795	
	2	0.63	0.315	
	3	1.49	0.745	
	**average**		**0.618**	**0.989**
2c	1	1.59	0.795	

2	2.42	1.210	
3	2.42	1.210	
**average**		**1.072**	**5.160**

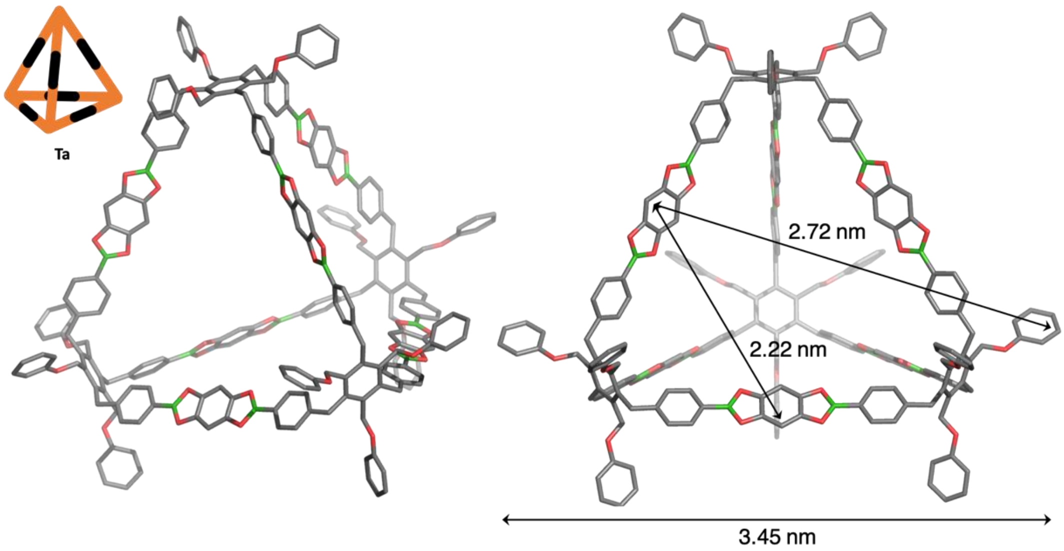

*Figure S85: Molecular model (OPLS4) of tetrahedral cage **Ta**.*

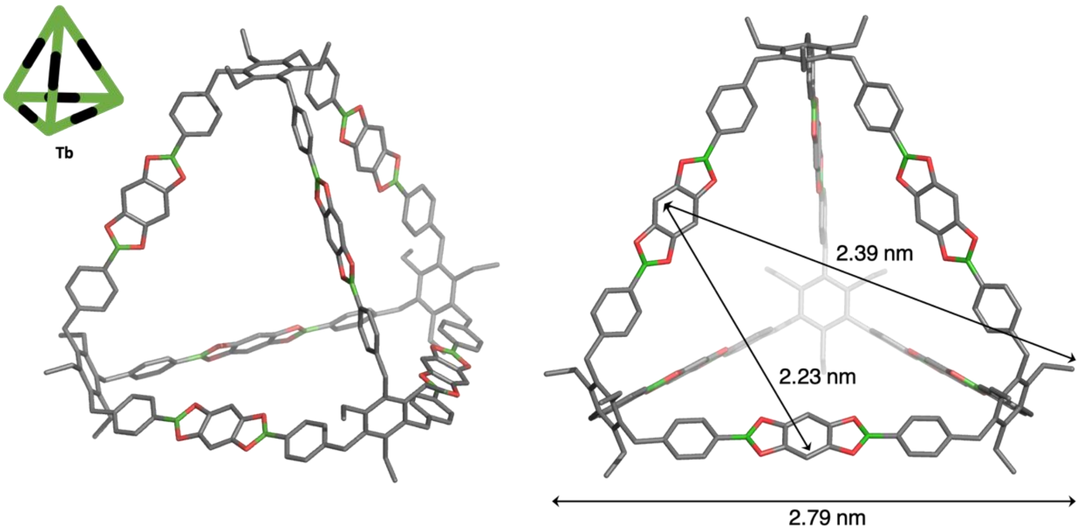

*Figure S86: Molecular model (OPLS4) of tetrahedral cage **Tb**.*

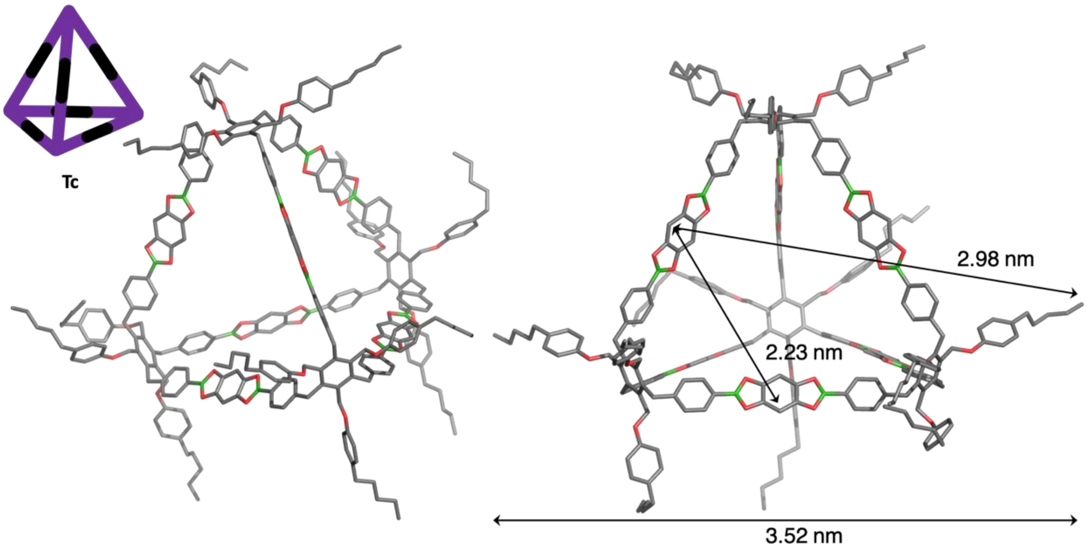

*Figure S87: Molecular model (OPLS4) of tetrahedral cage **Tc**.*

Table S15: Overview over volume calculation of tetrahedral cages based on molecular modeling.

cage		d [nm]	r [nm]	V [nm^3]
**Ta**	1	3.45	1.725	
	2	2.72	1.360	
	3	2.22	1.110	
	**average**		**1.398**	**11.445**
**Tb**	1	2.79	1.395	
	2	2.39	1.195	
	3	2.23	1.115	
	**average**		**1.235**	**7.890**
**Tc**	1	3.52	1.760	
	2	2.98	1.490	
	3	2.23	1.115	
	**average**		**1.455**	**12.903**

8.2 DFT-optimized structures of tripodal building blocks

Quantum mechanical calculations were done using the Gaussian 09 software. A molecular mechanics' conformational search using a hybrid Monte Carlo Multiple Minimun and Low Mode sampling mode and the OPLS4 force field were used for each tripod (Bpin-tripods **2a**, **2b** and **2d**). The Bpin-substituted tripods were used to allow for comparability with the X-ray crystal structures, avoid possible spurious structures from the boronic-acid functionality and to ensure compatibility with NMR-studies in $CDCl_3$, if necessary. Conformers found within 21 kJ/mol of the corresponding lowest energy structure found were further optimized at the M062X/6-31+G** level of theory. Frequency calculations for all optimized geometries were done at the previous level of theory to define the nature of the stationary point found.

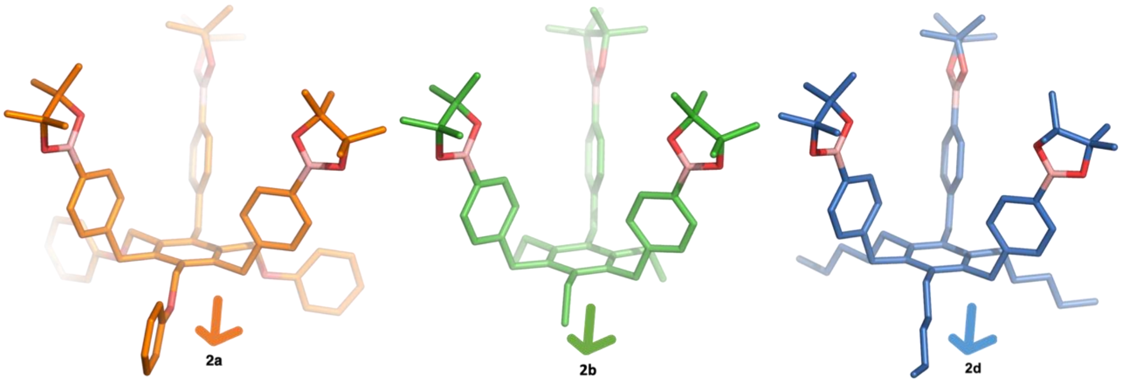

The angles crucial for the assembly of tetrapods/tetrahedral cages were defined as follows (Figure S41):
α: dihedral angle between atoms 1-2-3-4
β: angle between atoms 1-2-3
γ: 90°-β
∂: dihedral angle between atoms 1'-1-2-3

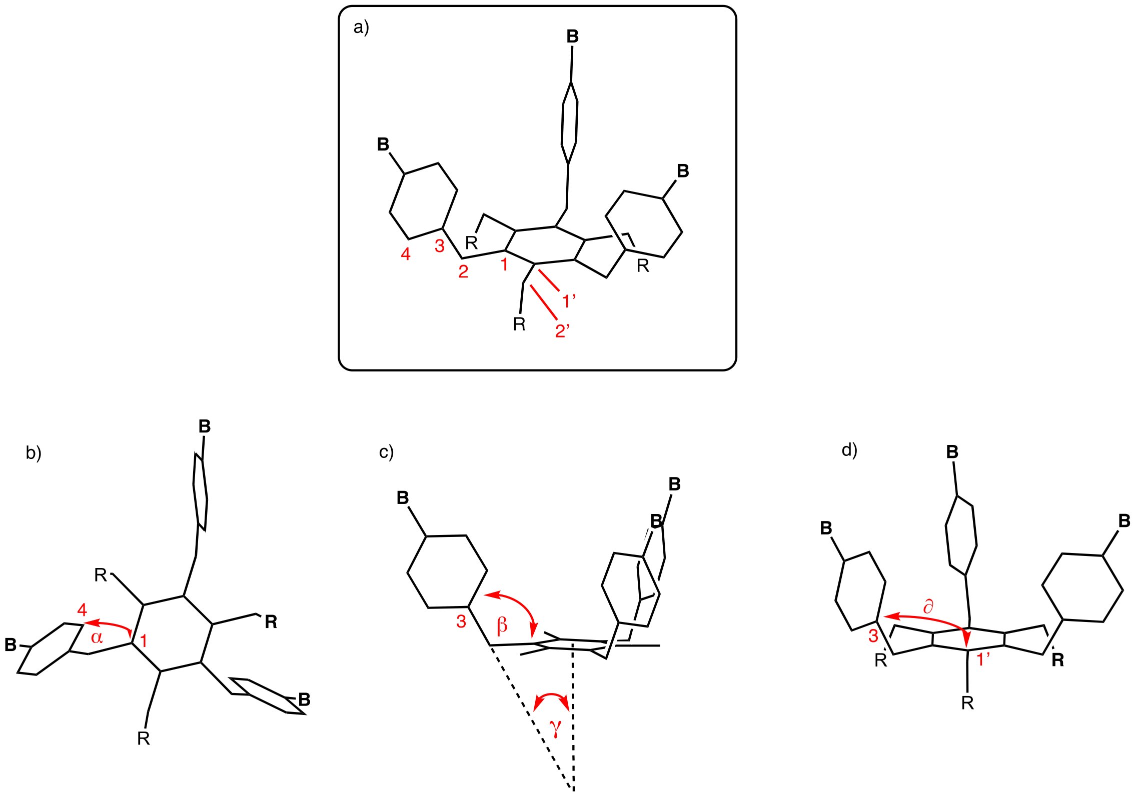

Figure S88: a) Numbering of atoms inside tripodal building-blocks. Definition of angles α (b), β and γ (c) and ∂ (d). The angle γ is obtained by γ = β-90°. The valency and the pinacol-ester attached to the boron-atoms have been omitted for clarity. In c) also the tripodal feet R are omitted.

*Table S16: Angle analysis for DFT-minimized tripods **2a**, **2c** and **2d**.*

	**2a**	**2c**	**2d**
α_1	22.8	22.7	24.6
α_2	23.1	18.8	24.0
α_3	23.1	23.1	24.4
$\bar{\alpha}$	23.0	21.5	24.3
β_1	114.4	114.0	113.9
β_2	114.4	114.0	114.0
β_3	114.4	114.0	114.0
$\bar{\beta}$	114.4	114.0	114.0
γ_1	24.4	24.0	23.9
γ_2	24.4	24.0	24.0
γ_3	24.4	24.0	24.0
$\bar{\gamma}$	24.4	24.0	24.0
∂_1	85.1	83.6	83.1
∂_2	85.1	83.6	83.1
∂_3	85.1	83.5	83.2
$\bar{\partial}$	85.1	83.6	83.1

8.3 DFT-optimized structures of tetrahedral cages

Quantum mechanical calculations were done using the Gaussian 09 software. A molecular mechanics' conformational search using a hybrid Monte Carlo Multiple Minimun and Low Mode sampling mode and the OPLS4 force field were used for each cage (**Ta**, **Tb** and **Td**). Due to the large size of the structures, each cage was optimized at the B3LYP/3-21G level of theory. Frequency calculations were done, and no negative values were found indicating energy minima structures. **Tc** was not optimized due to redundancy in the yield and structure compared to **Ta**.

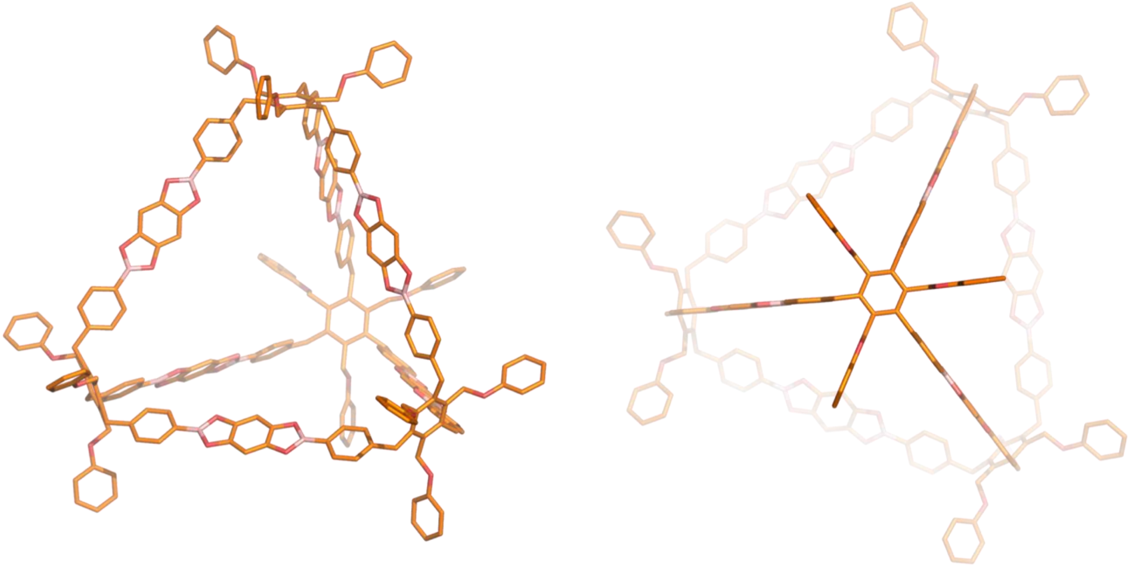

*Figure S89: DFT-optimized structure of tetrahedral cage **Ta**, side view (left) and top-view onto vertex (right). Hydrogen atoms and valence were omitted for clarity.*

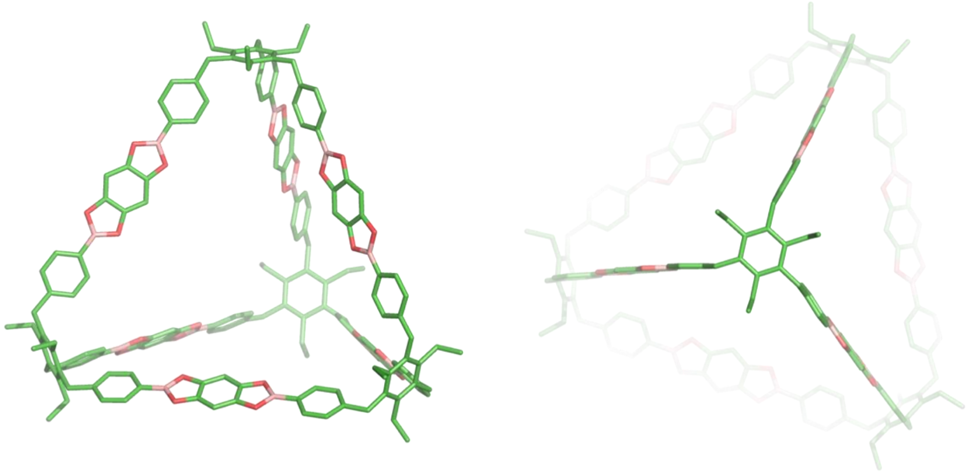

*Figure S90: DFT-optimized structure of tetrahedral cage **Tb**, side view (left) and top-view onto vertex (right). Hydrogen atoms and valence were omitted for clarity.*

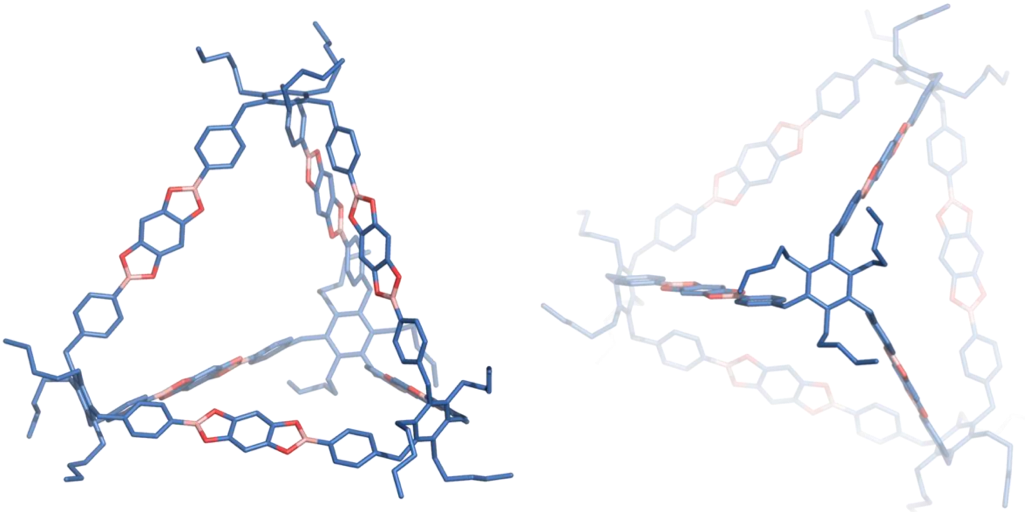

*Figure S91: DFT-optimized structure of tetrahedral cage **Tc**, side view (left) and top-view onto vertex (right). Hydrogen atoms and valence were omitted for clarity.*

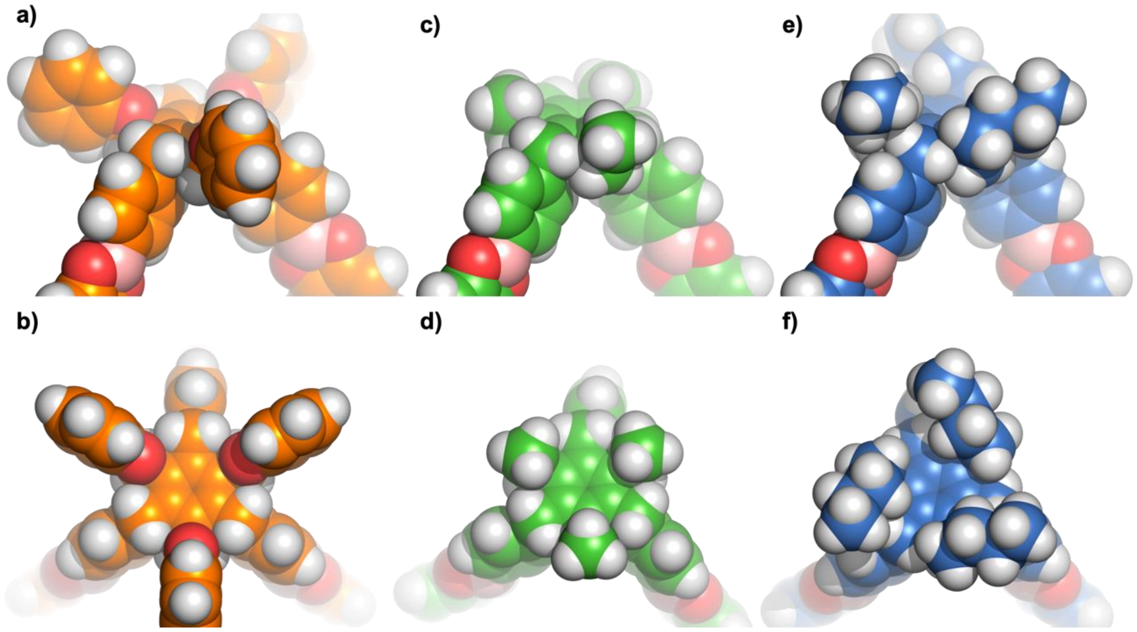

*Figure S92: Detailed view of the tetrahedral vertices of DFT-optimized cages. Side-on (a) and top-view (b) of **Ta**, side-on (c) and top-view (d) of **Tb** and side-on (d) and top-view (f) of **Tc**.*

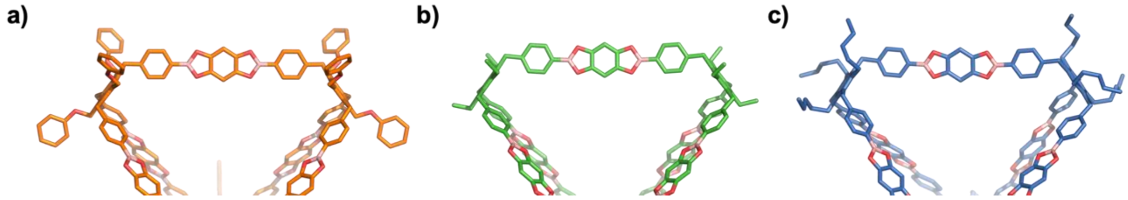

*Figure S93: Detailed view of the tetrahedral edges of DFT-optimized cages **Ta** (a), **Tb** (b) and **Td** (c).*

To confirm that the geometrical differences obtained for the optimized structures indeed arose from the differences in the tripodal feet/substituents we performed structural “permutations” with cages **Ta** and **Tb** (see Figure S94). This means that the DFT-optimized structure of **Ta** was transformed into **Tb*** by changing the tripodal feet without performing any minimization/relaxation step. Then, the permutated structure **Tb*** was submitted to DFT-minimization to generate structure **Tb’**. The same protocol was applied to **Tb** (to **Ta*** and **Ta’**). Analysis of the geometrical features showed nearly perfect agreement between **Tb**/**Tb’** and **Ta**/**Ta’,** respectively.

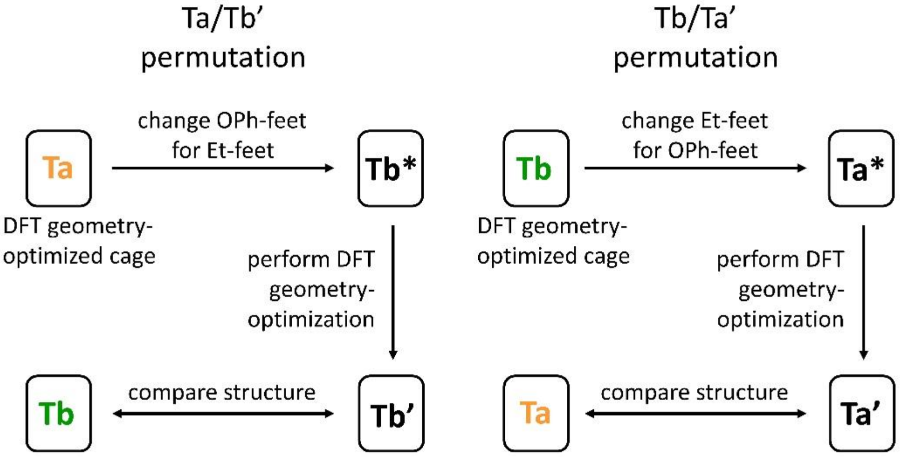

*Figure S94: Schematic explanation of the “structural permutations” performed for cages **Ta** and **Tb**.*

For the DFT-minimized structures of cages **Ta**, **Ta’**, **Tb**, **Tb’** and **Td** all angles of relevance were measured and analyzed. The results are reported in Table S17 - Table S21. The angles α, β, γ, ∂ are equivalent to the angles defined for the tripodal precursors (Section 8.2), the sub-indices 1-3 define the three arms of the tripod, while the top-bar refers to the average of these three. Vertex 1-4 refers to the four vertices (tripodal building blocks) inside one tetrahedron. The angle ε refers to the strain-angle of the tetrahedron edges, enclosed by (atom 2 of a vertex) – (centroid of **THB**) – (atom 2 of neighbouring vertex) (see Figure S95). St. Dev. is the standard deviation function, Min. and Max. give the respective highest and lowest value and the range is obtained by subtraction of those two. The term |Tx-2x| (where x refers to the letter that defines the respective tripods/cages a,c,d) is the difference between the averaged precursor angle (from DFT-minimization) and the averaged angle of the same building-block inside the tetrahedron (DFT-minimized). The term |ε-180°| is the absolute value of the difference of averaged ε from the ideal edge-angle (180°) of a tetrahedron.

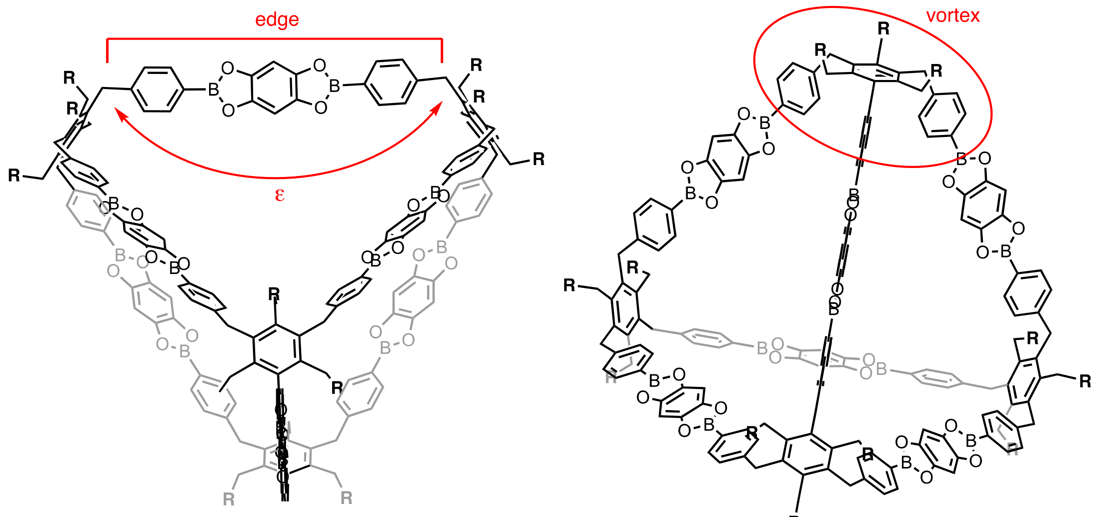

Figure S95: Definition of "vertex", "edge" and angle ε of tetrahedral cages.

Table S17: Angle analysis of tetrahedral cage ***Ta****.*

Ta	α_1	α_2	α_3	$\bar{\alpha}$	β_1	β_2	β_3	$\bar{\beta}$	γ_1	γ_2	γ_3	$\bar{\gamma}$	∂_1	∂_2	∂_3	$\bar{\partial}$	$\varepsilon_{1\text{-}6}$
**Vertex 1**	1.3	1.8	1.7		115.7	115.7	115.7		25.7	25.7	25.7		92.6	92.5	92.5		179.7
**Vertex 2**	0.9	0.1	1.0		115.7	115.7	115.7		25.7	25.7	25.7		92.8	92.9	92.7		179.6
**Vertex 3**	1.4	0.5	1.6		115.7	115.7	115.7		25.7	25.7	25.7		93.4	93.3	93.4		179.7
**Vertex 4**	1.1	1.1	0.6		115.7	115.7	115.7		25.7	25.7	25.7		93.3	93.2	93.2		179.7
**Average**				1.1				115.7				25.7				93.0	179.7
**st. Dev.**				0.5				0.0				0.0				0.4	179.7
**Min.**				0.1				115.7				25.7				92.5	**$\bar{\varepsilon}$:**
**Max.**				1.8				115.7				25.7				93.4	179.7
**Range**				1.7				0.0				0.0				0.9	**|ε-180|:**
**|Ta-2a|**				21.9				1.3				1.3				7.9	0.3

Table S18: Angle analysis of permutated tetrahedral cage ***Ta'****.*

Ta'	α_1	α_2	α_3	$\bar{\alpha}$	β_1	β_2	β_3	$\bar{\beta}$	γ_1	γ_2	γ_3	$\bar{\gamma}$	∂_1	∂_2	∂_3	$\bar{\partial}$	$\varepsilon_{1\text{-}6}$
**Vertex 1**	0.2	0.9	0.9		115.7	115.7	115.7		25.7	25.7	25.7		93.1	93.1	93.1		179.7
**Vertex 2**	0.5	1.7	1.2		115.7	115.7	115.7		25.7	25.7	25.7		93.1	93.4	93.2		179.6
**Vertex 3**	1.1	1.3	0.6		115.7	115.7	115.7		25.7	25.7	25.7		92.7	92.6	92.8		179.7
**Vertex 4**	1.8	0.9	0.6		115.7	115.7	115.7		25.7	25.7	25.7		92.7	92.5	92.7		179.7
**Average**				1.0				115.7				25.7				92.9	179.7
**st. Dev.**				0.5				0.0				0.0				0.3	179.7
**Min.**				0.2				115.7				25.7				92.5	**$\bar{\varepsilon}$:**
**Max.**				1.8				115.7				25.7				93.4	179.7
**Range**				1.6				0.0				0.0				0.9	**|ε-180|:**
**|Ta'-2a|**				22.0				1.3				1.3				7.8	0.3

*Table S19: Angle analysis of tetrahedral cage **Tb**.*

**Tb**	α_1	α_2	α_3	$\bar{\alpha}$	β_1	β_2	β_3	$\bar{\beta}$	γ_1	γ_2	γ_3	$\bar{\gamma}$	∂_1	∂_2	∂_3	$\bar{\partial}$	$\varepsilon_{1\text{-}6}$
**Vertex 1**	25.2	25.0	19.9		115.4	115.8	115.7		25.4	25.8	25.7		100.2	100.9	100.6		174.5
**Vertex 2**	25.1	24.9	18.9		115.8	115.4	115.7		25.8	25.4	25.7		81.0	81.0	80.4		175.1
**Vertex 3**	25.3	19.2	25.4		115.7	115.4	115.7		25.7	25.4	25.7		80.3	80.8	80.8		174.6
**Vertex 4**	25.2	25.2	18.9		115.4	115.8	115.7		25.4	25.8	25.7		100.7	100.2	100.9		175.1
**Average**				23.2				115.6				25.6				90.7	174.5
**st. Dev.**				2.9				0.2				0.2				10.4	174.5
**Min.**				18.9				115.4				25.4				80.3	**$\bar{\varepsilon}$:**
**Max.**				25.4				115.8				25.8				100.9	174.5
**Range**				6.5				0.4				0.4				20.6	**$\|\|\varepsilon$-180$\|$:**
**|Tb-2b|**				1.7				1.6				1.6				7.1	5.3

*Table S20: Angle analysis of permutated tetrahedral cage **Tb'**.*

**Tb'**	α_1	α_2	α_3	$\bar{\alpha}$	β_1	β_2	β_3	$\bar{\beta}$	γ_1	γ_2	γ_3	$\bar{\gamma}$	∂_1	∂_2	∂_3	$\bar{\partial}$	$\varepsilon_{1\text{-}6}$
**Vertex 1**	19.2	25.0	25.2		115.7	115.8	115.4		25.7	25.8	25.4		80.3	80.9	80.9		174.6
**Vertex 2**	19.2	25.1	25.2		115.8	115.4	115.7		25.8	25.4	25.7		80.3	80.8	80.9		174.6
**Vertex 3**	25.1	19.1	25.1		115.8	115.7	115.4		25.8	25.7	25.4		100.7	101.0	100.1		175.1
**Vertex 4**	19.1	25.0	25.0		115.7	115.4	115.8		25.7	25.4	25.8		101.0	100.1	100.7		175.1
**Average**				23.1				115.6				25.6				90.6	174.6
**st. Dev.**				2.9				0.2				0.2				10.4	174.5
**Min.**				19.1				115.4				25.4				80.3	**$\bar{\varepsilon}$:**
**Max.**				25.2				115.8				25.8				101.0	174.8
**Range**				6.1				0.4				0.4				20.7	**$\|\varepsilon$-180$\|$:**
**|Tb'-2b|**				1.6				1.6				1.6				7.0	5.3

*Table S21: Angle analysis of tetrahedral cage **Td**.*

**Td**	α_1	α_2	α_3	$\bar{\alpha}$	β_1	β_2	β_3	$\bar{\beta}$	γ_1	γ_2	γ_3	$\bar{\gamma}$	∂_1	∂_2	∂_3	$\bar{\partial}$	ε_{1-6}
**Vertex 1**	30.0	29.7	29.6		115.1	115.1	115.1		25.1	25.1	25.1		69.9	69.9	69.8		174.1
**Vertex 2**	29.2	29.1	29.2		115.1	115.1	115.1		25.1	25.1	25.1		70.3	70.0	70.1		174.1
**Vertex 3**	29.2	29.1	29.0		115.1	115.1	115.0		25.1	25.1	25.0		70.1	70.1	70.0		174.1
**Vertex 4**	29.6	29.3	29.1		115.1	115.1	115.1		25.1	25.1	25.1		70.2	70.1	70.1		174.1
**Average**				29.3				115.1				25.1				70.1	174.0
**st. Dev.**				0.3				0.0				0.0				0.1	174.0
**Min.**				29.0				115.0				25.0				69.8	**$\bar{\varepsilon}$:**
**Max.**				30.0				115.1				25.1				70.3	174.1
**Range**				1.0				0.1				0.1				0.5	**$\lvert\varepsilon$-180$\rvert$:**
**|Td-2d|**				5.0				1.1				1.1				13.1	5.9

In an attempt to rationalize the yield difference between **Ta** and **Tb** based on the conformational attributes of the tripodal building blocks, a series of computational calculations were carried out. The results did not provide enough evidence to draw clear conclusions, however for the sake of completeness they are below-mentioned.

8.4 Boltzmann distribution of tripods 2a and 2b

To corroborate the differences in the conformational behavior of tripods **2a** and **2b**, conformers found within 21 kJ/mol of the corresponding lowest energy structure were further optimized at the M062X/6-31+G** level of theory. Boltzmann distributions were calculated using the DFT energy of conformer i (relative to the lowest energy conformer), obtained at the M062X/6-31+G** level of theory at 298. The obtained values are shown in Table S22.

*Table S22: Relative Gibbs Energies and Boltzmann distributions (293K) of conformers of tripods **2a** and **2b**.*

Structure	Tripod 2a Gibbs Energy [kcal/mol]	Boltzmann [%]	Structure	Tripod 2b Gibbs Energy [kcal/mol]	Boltzmann [%]
1	-1.3095305E+06	69.1	1	-805262.5407	82.9
5	-1.3095305E+06	4.3	4	-805260.5310	2.8
4	-1.3095300E+06	3.0	5	-805260.5159	2.8
3	-1.3095303E+06	2.4	2	-805260.3936	2.2
34	-1.3095308E+06	2.4	3	-805260.3936	2.2
14	-1.3095282E+06	2.2	7	-805260.1933	1.6
10	-1.3095293E+06	1.3	6	-805260.1895	1.6
11	-1.3095293E+06	1.3	8	-805260.1675	1.5
13	-1.3095293E+06	0.9	9	-805260.1662	1.5
9	-1.3095298E+06	0.7	11	-805259.0996	0.3
8	-1.3095329E+06	0.7	10	-805259.0990	0.3
45	-1.3095298E+06	0.6	14	-805258.5471	0.1
31	-1.3095288E+06	0.6	12	-805258.4526	0.1
12	-1.3095289E+06	0.5			
33	-1.3095288E+06	0.5			
35	-1.3095295E+06	0.4			
25	-1.3095298E+06	0.4			
26	-1.3095298E+06	0.4			
20	-1.3095288E+06	0.4			
19	-1.3095288E+06	0.4			
55	-1.3095296E+06	0.4			
52	-1.3095309E+06	0.3			
56	-1.3095296E+06	0.3			
68	-1.3095300E+06	0.3			
29	-1.3095294E+06	0.3			
63	-1.3095299E+06	0.3			
30	-1.3095309E+06	0.3			
64	-1.3095298E+06	0.3			
40	-1.3095293E+06	0.2			
41	-1.3095292E+06	0.2			
**7**	-1.3095287E+06	0.2			
24	-1.3095295E+06	0.2			
6	-1.3095295E+06	0.2			
51	-1.3095270E+06	0.2			
32	-1.3095293E+06	0.2			
50	-1.3095300E+06	0.2			
49	-1.3095293E+06	0.2			
37	-1.3095272E+06	0.2			
16	-1.3095290E+06	0.2			
44	-1.3095293E+06	0.2			
46	-1.3095310E+06	0.2			
17	-1.3095294E+06	0.2			
18	-1.3095295E+06	0.2			
67	-1.3095297E+06	0.2			
66	-1.3095268E+06	0.2			
57	-1.3095286E+06	0.2			
38	-1.3095297E+06	0.1			
62	-1.3095297E+06	0.1			

61	-1.3095292E+06	0.1
65	-1.3095279E+06	0.1
48	-1.3095279E+06	0.1
60	-1.3095312E+06	0.1
22	-1.3095290E+06	0.1
23	-1.3095292E+06	0.1
21	-1.3095292E+06	0.1
27	-1.3095296E+06	0.1
28	-1.3095295E+06	0.1
39	-1.3095292E+06	0.1
54	-1.3095292E+06	0.1

8.5 Molecular dynamics

Molecular dynamics simulations using the OPLS3 force field were performed at 400K (this being the approximate reaction temperature for the assembly of the cages) over a time-range of 1000µs using the OPLS3-minimized structures as the starting points. The calculations were carried out with the corresponding Bpin-tripod precursors to enable easy experimental support via NMR in $CDCl_3$ and to avoid artifacts resulting from intramolecular hydrogen-bonding.

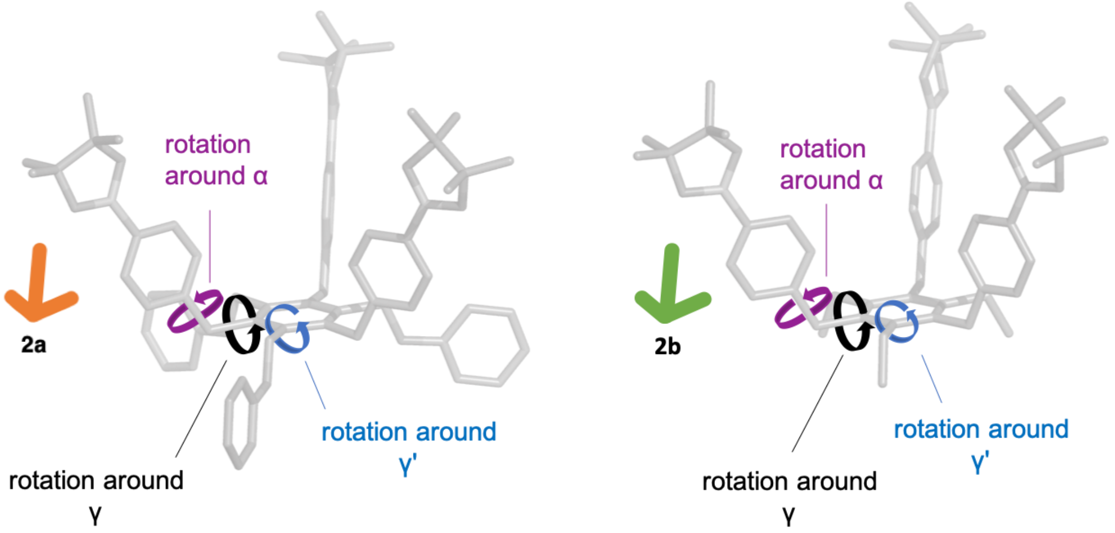

*Figure S96: Definition of rotations of Bpin-tripods **2a** and **2b**.*

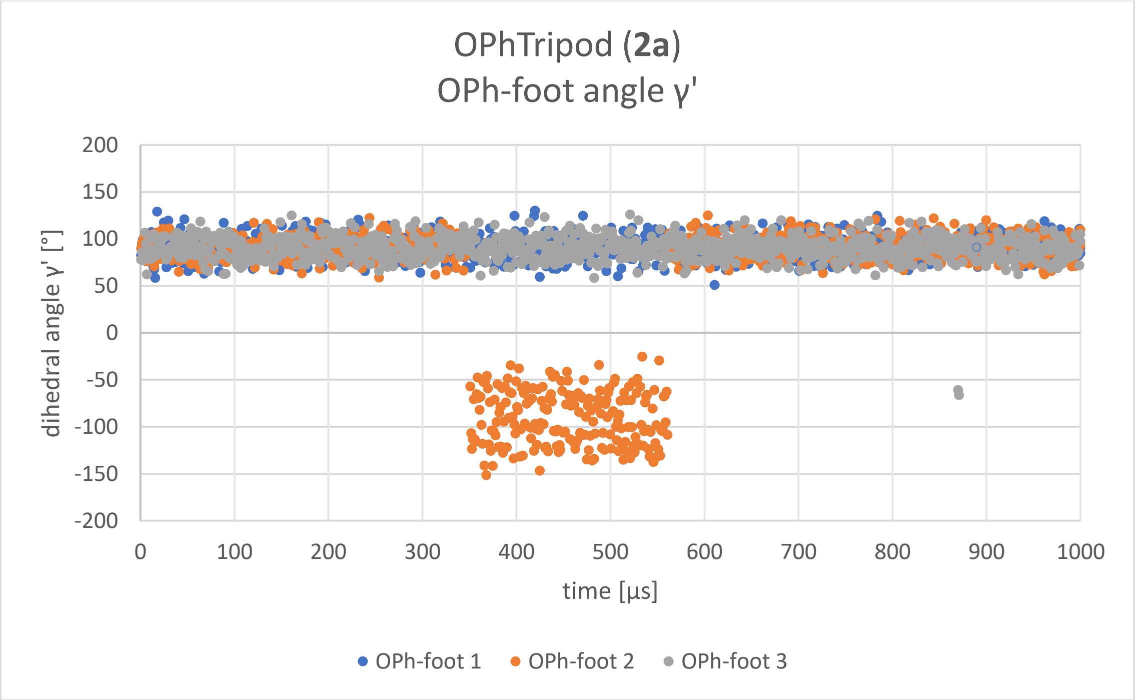

*Figure S97: Movement of the OPh-foot angle γ' of **2a** based on MD simulation at 400 K for 1000 µs. The numbers1-3 define the results obtained from the three different feet of the tripod.*

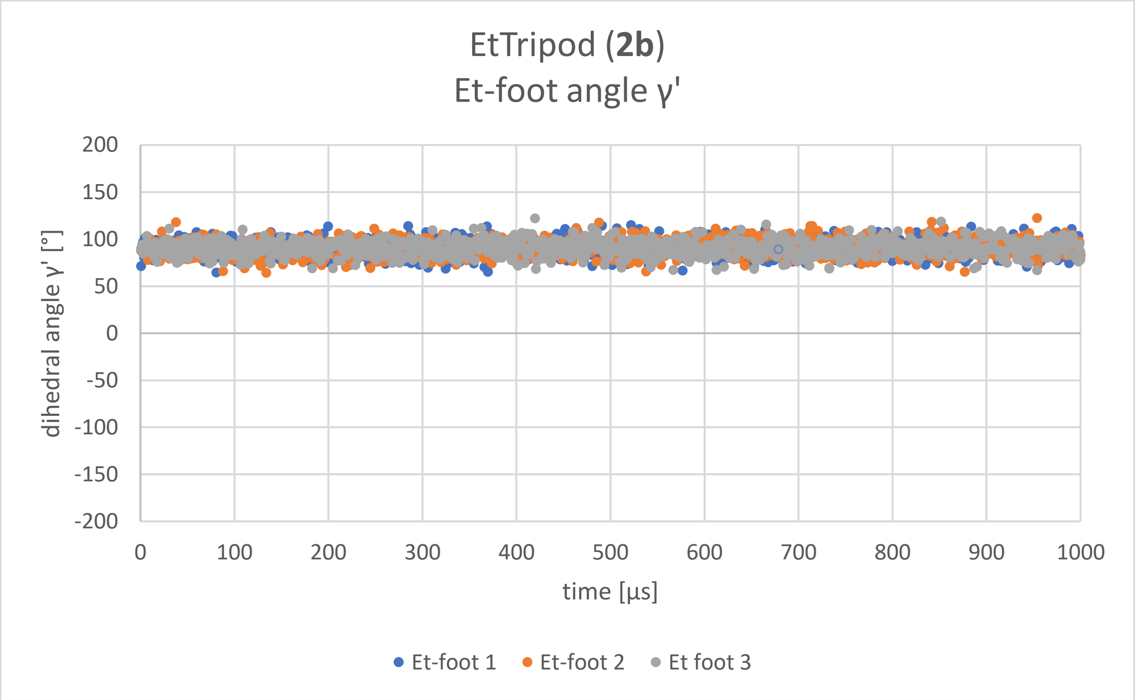

*Figure S98: : Movement of the Et-foot angle γ' of **2b** based on MD simulation at 400 K for 1000 µs. The numbers1-3 define the results obtained from the three different feet of the tripod.*

*Table S23: Analysis of the dihedral angles γ' of the OPh- and Et-feet movement for the MD simulation of **2a** and **2b** at 400 K. Time-averaged dihedral angle, standard deviation, maximal values, minimal values and movement range. The letters 1-3 define the results obtained from the three different feet of the tripod.*

**2a**	γ'_1	γ'_2	γ'_3	$\overline{\gamma}'$
Average 1000µs	90.0	52.5	89.8	**77.4**
St. Dev. 1000µs	11.4	76.0	13.2	**33.5**
Max. Val. 1000µs	129.7	124.8	125.6	**126.7**
Min. Val. 1000µs	50.7	-151.9	-66.5	**-55.9**
Range (Max-Min)	79.0	276.8	192.1	**182.6**
**2b**				
Average 1000µs	90.6	90.5	90.5	**90.5**
St. Dev. 1000µs	8.1	8.3	8.1	**8.2**
Max. Val. 1000µs	114.8	122.3	121.9	**65.1**
Min. Val. 1000µs	64.5	64.0	66.8	**-119.7**
Range (Max-Min)	50.3	58.2	55.1	**54.6**

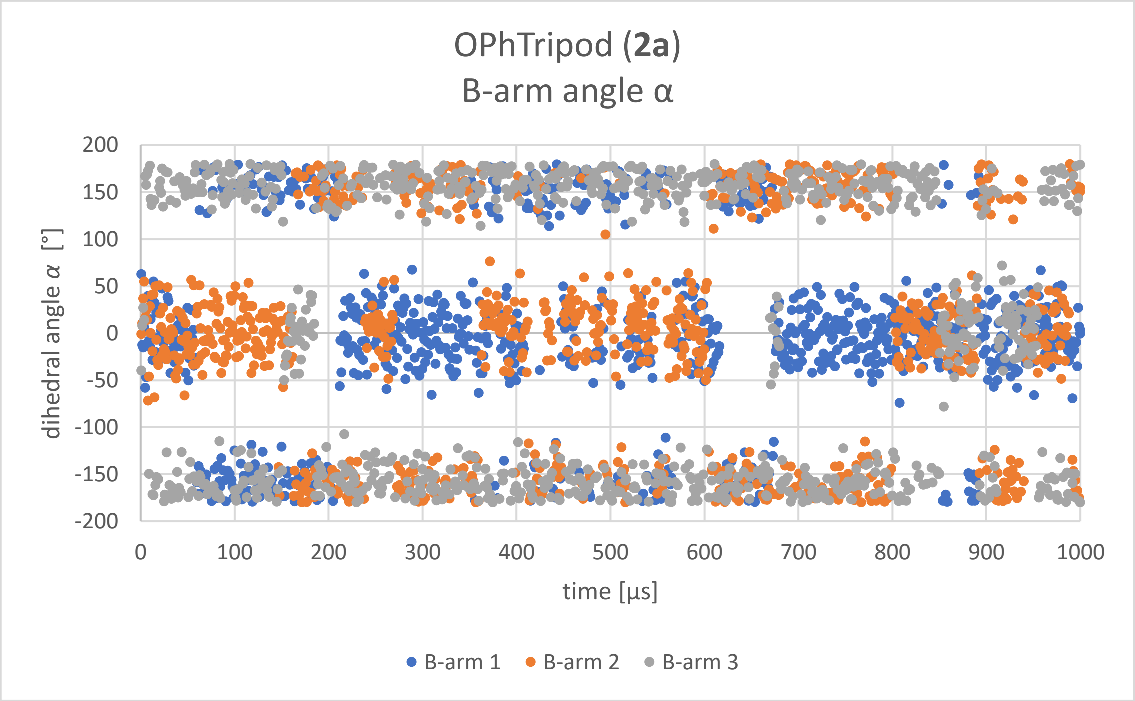

*Figure S99: Movement of the B-arm angle α of **2a** based on MD simulation at 400 K for 1000 µs. The numbers1-3 define the results obtained from the three different arms of the tripod.*

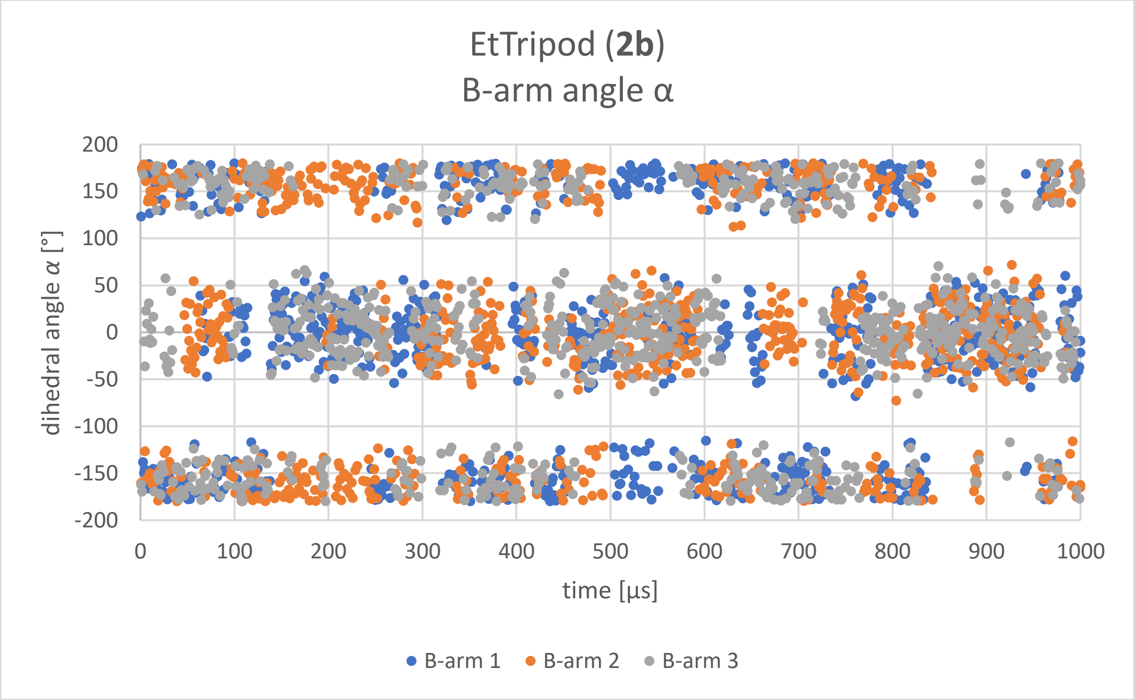

*Figure S100: Movement of the B-arm angle α of **2b** based on MD simulation at 400 K for 1000 µs. The numbers1-3 define the results obtained from the three different arms of the tripod.*

*Table S24: Analysis of the dihedral angles α for the MD simulation of **2a** and **2b** at 400 K. Time-averaged dihedral angle, standard deviation, maximal values, minimal values and movement range. The letters 1-3 define the results obtained from the three different B-arms of the tripod.*

**2a**	α_1	α_2	α_3	$\bar{\alpha}$
Average 1000µs	-0.9	-4.1	1.1	**-1.3**
St. Dev. 1000µs	95.6	113.3	148.5	**119.1**
Max. Val. 1000µs	179.6	179.8	180.0	**179.8**
Min. Val. 1000µs	-179.6	-180.0	-180.0	**-179.9**
Range (Max-Min)	359.2	359.8	359.9	**359.6**
**2b**				
Average 1000µs	-2.1	2.3	1.6	**0.6**
St. Dev. 1000µs	114.5	116.5	108.4	**113.1**
Max. Val. 1000µs	180.0	179.8	179.9	**179.9**
Min. Val. 1000µs	-179.9	-179.9	-180.0	**-179.9**
Range (Max-Min)	359.2	359.7	359.9	**359.8**

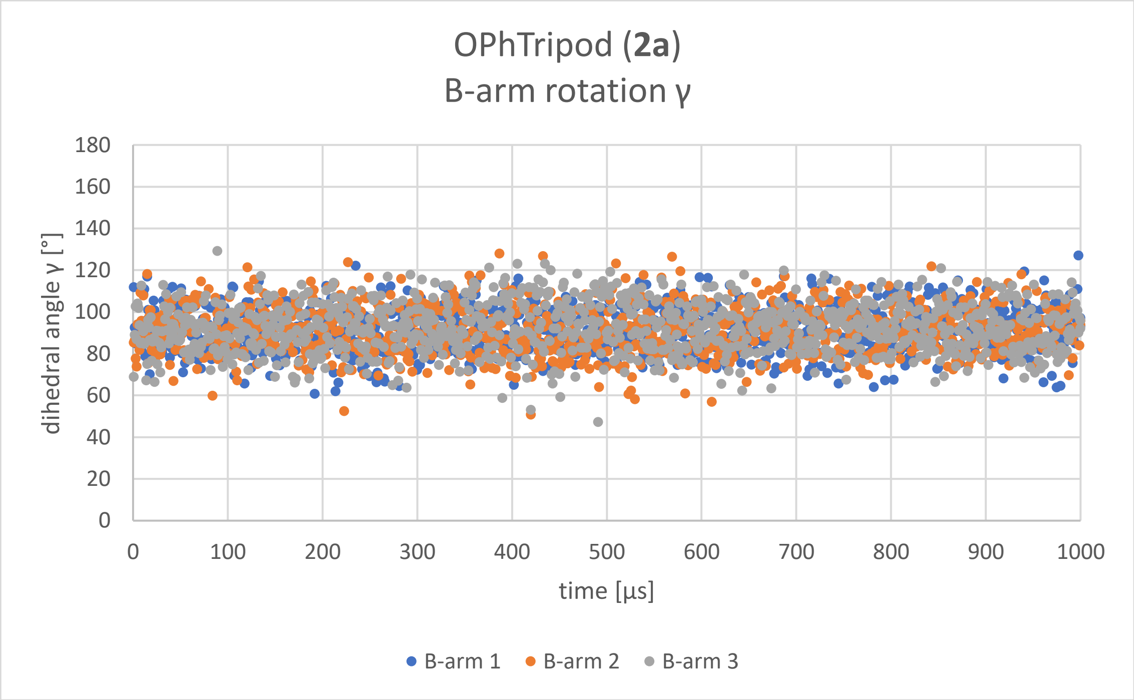

*Figure S101: Movement of the B-arm angle γ of **2a** based on MD simulation at 400 K for 1000 µs. The numbers1-3 define the results obtained from the three different arms of the tripod.*

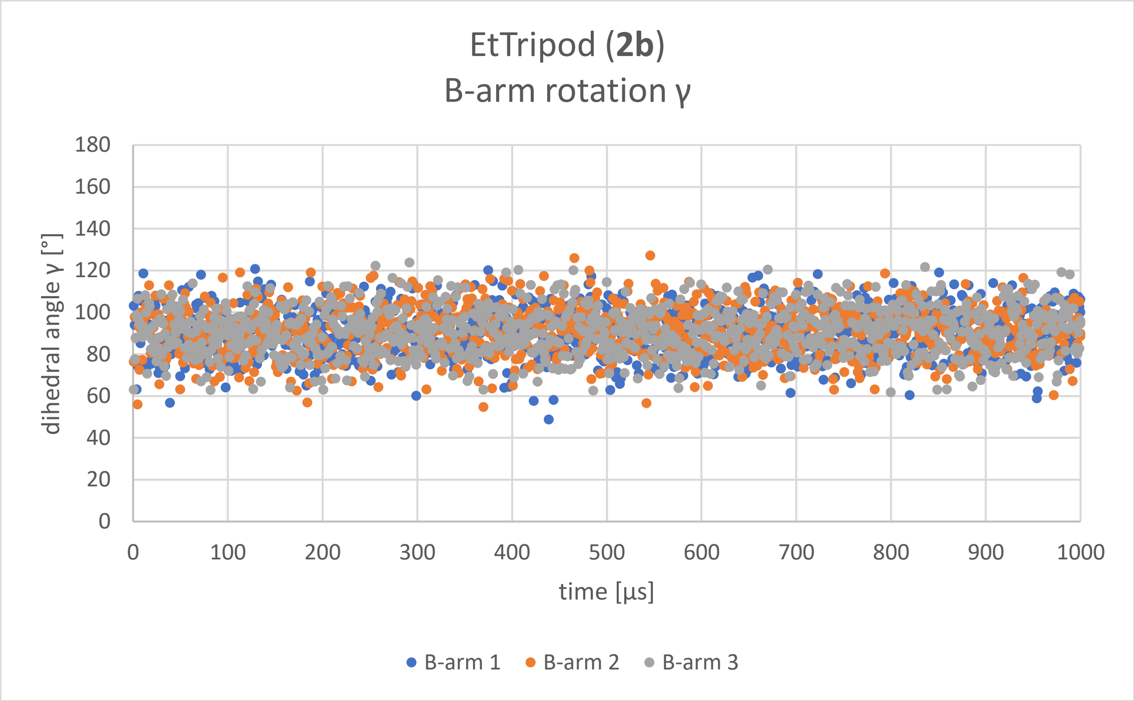

Figure S102: Movement of the B-arm angle γ of ***2b*** *based on MD simulation at 400 K for 1000 µs. The numbers1-3 define the results obtained from the three different arms of the tripod.*

Table S25: Analysis of the dihedral γ for the MD simulation of ***2a*** *and* ***2b*** *at 400 K. Time-averaged dihedral angle, standard deviation, maximal values, minimal values and movement range. The letters 1-3 define the results obtained from the three different B-arms of the tripod.*

**2a**	γ_1	γ_2	γ_3	$\bar{\gamma}$
Average 1000µs	91.2	91.2	92.4	**91.6**
St. Dev. 1000µs	10.7	11.4	11.8	**11.3**
Max. Val. 1000µs	126.9	127.9	129.1	**128.0**
Min. Val. 1000µs	60.6	50.6	47.2	**52.8**
Range (Max-Min)	66.6	77.3	81.9	**75.1**
**2b**				
Average 1000µs	90.1	90.8	90.5	**90.5**
St. Dev. 1000µs	11.5	11.6	11.3	**11.4**
Max. Val. 1000µs	120.8	127.1	123.8	**123.9**
Min. Val. 1000µs	48.8	54.8	61.8	**55.1**
Range (Max-Min)	71.9	72.4	62.0	**68.8**

8.6 Coordinate Scans

Coordinate scans were performed using the OPLS3 force field, using OPLS3-minimized structures as the starting points. The calculations were carried out with the corresponding Bpin-tripod precursors to enable easy experimental support via NMR in $CDCl_3$ and to avoid artefacts resulting from intramolecular hydrogen-bonding.

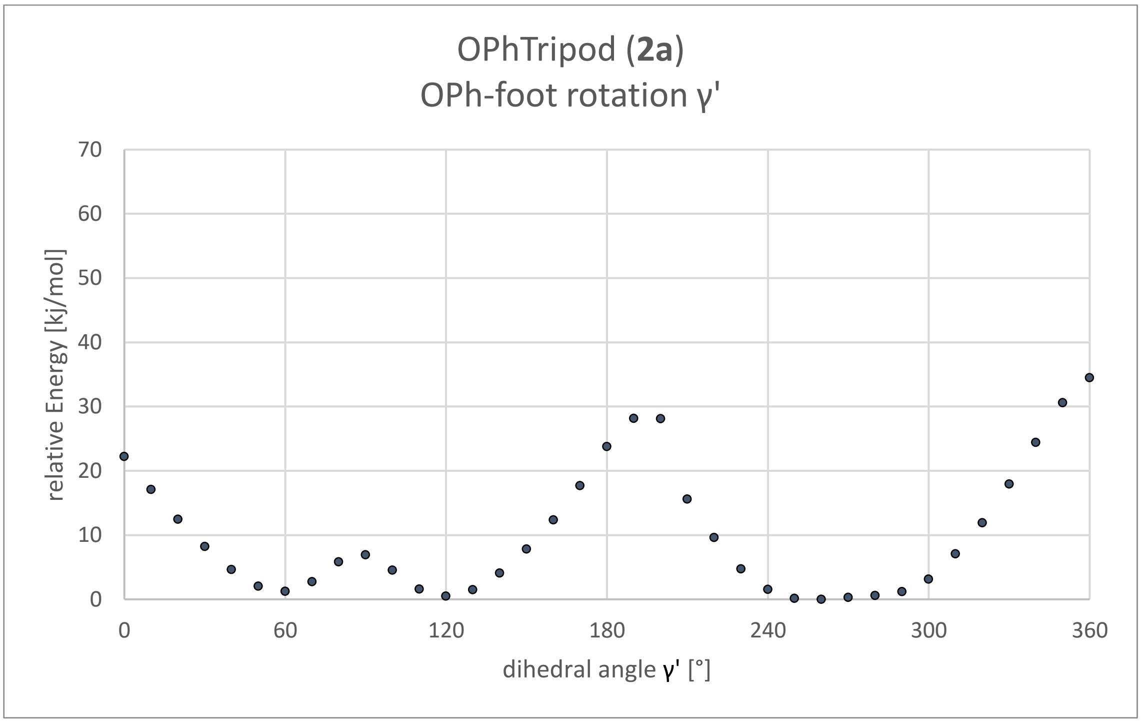

*Figure S103: Coordinate scan of one OPh-foot rotation of **2a**.*

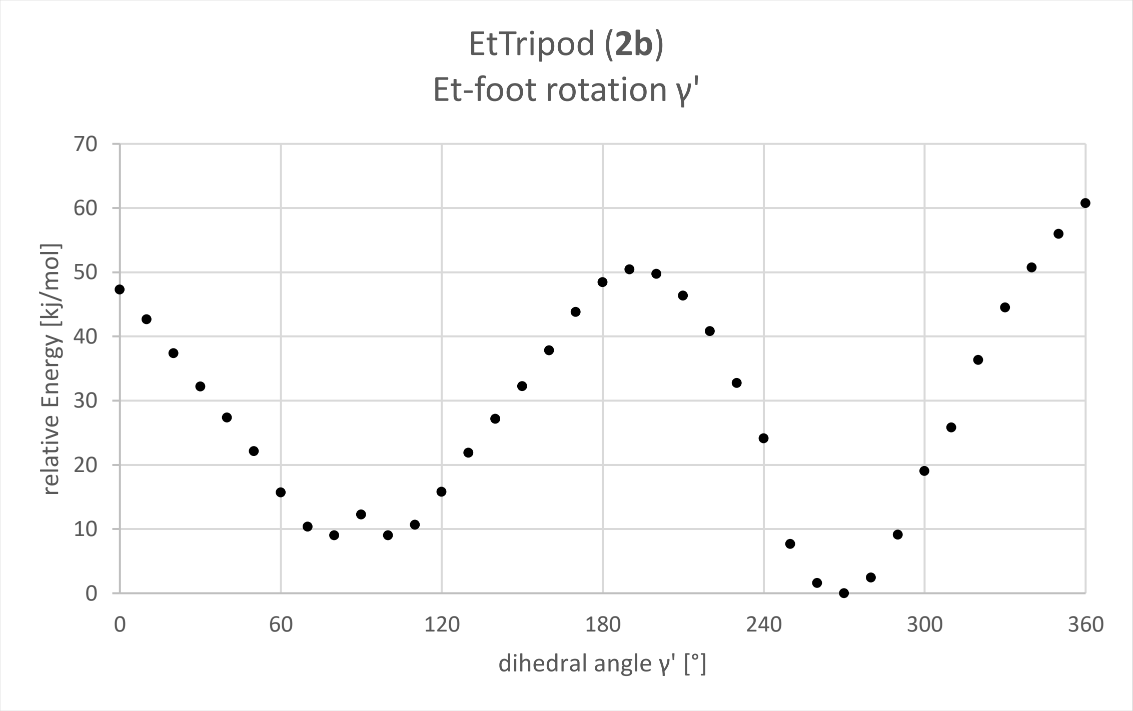

*Figure S104: Coordinate scan of one Et-foot rotation of **2b**.*

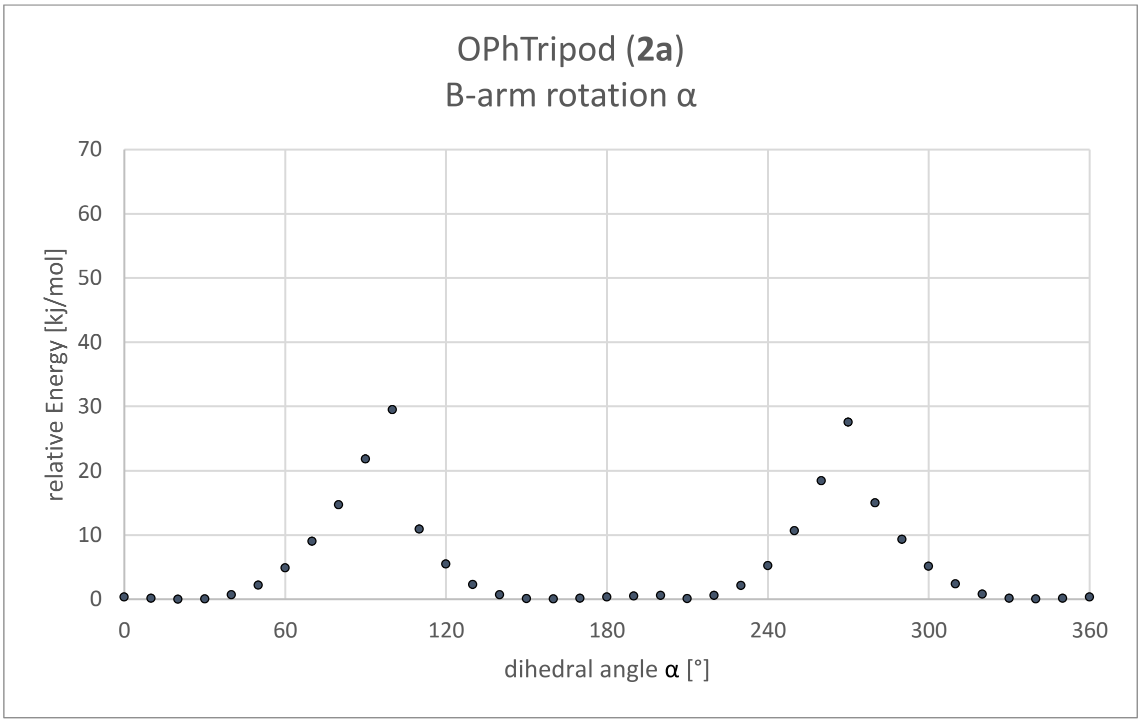

*Figure S105: Coordinate scan of the rotation around dihedral angle α of **2a**.*

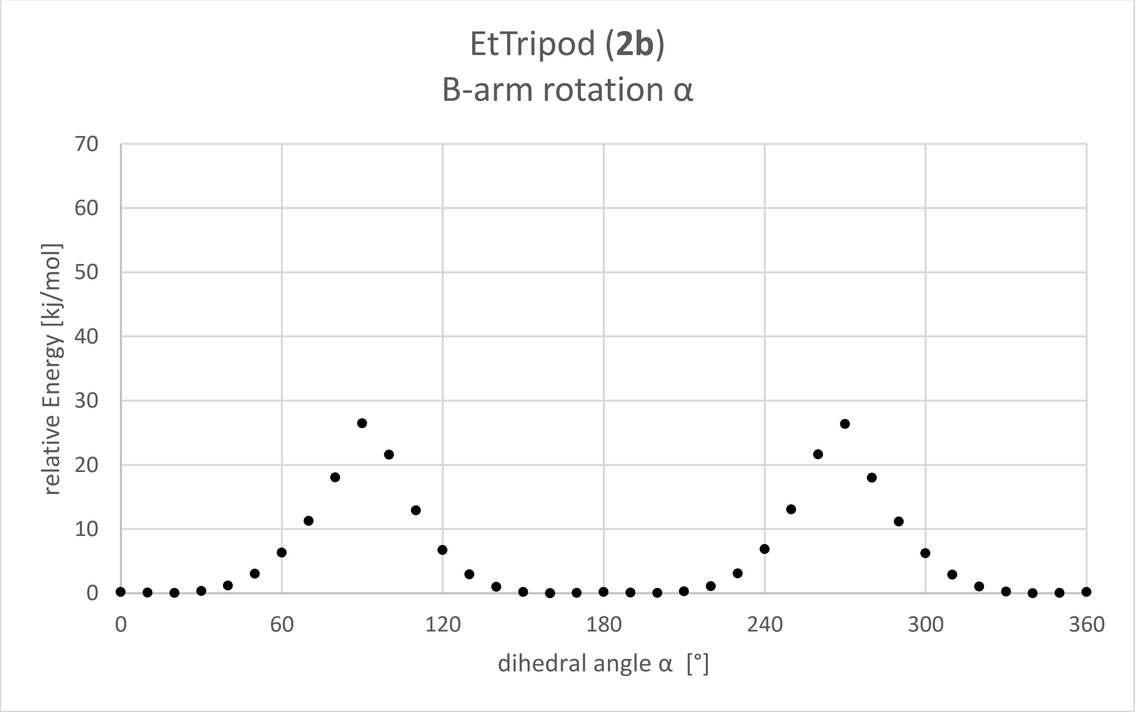

*Figure S106: Coordinate scan of the rotation around dihedral angle α of **2b**.*

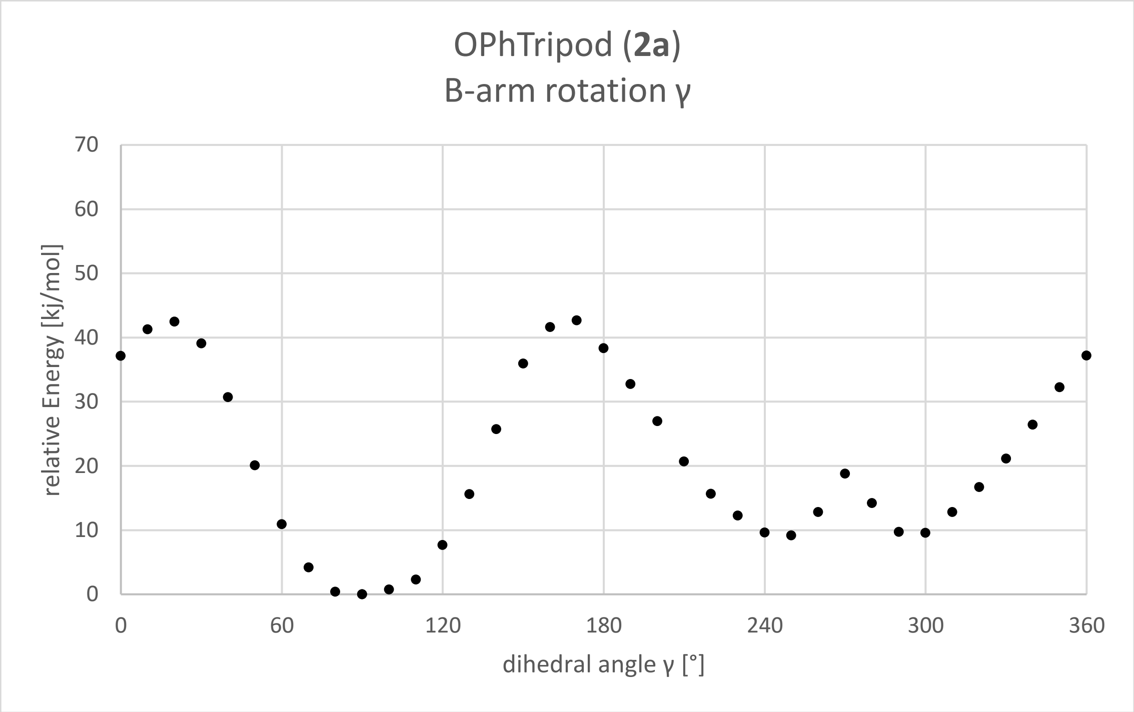

*Figure 107: Coordinate scan of the rotation around dihedral angle γ of **2a**.*

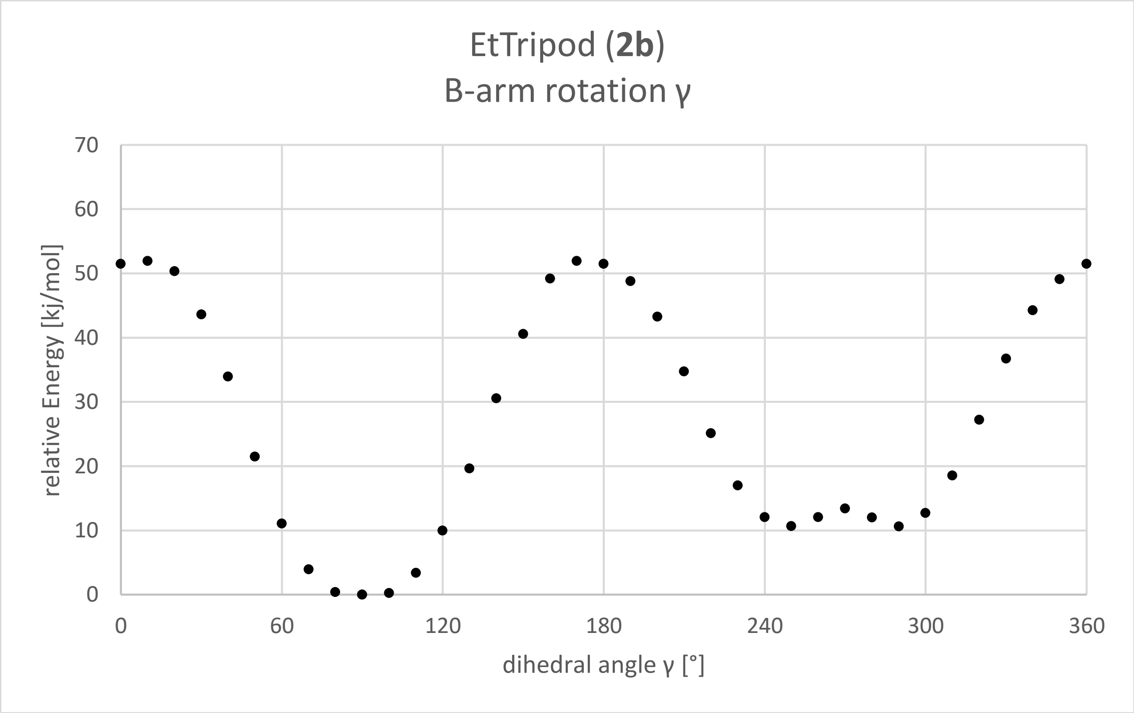

*Figure 108: Coordinate scan of the rotation around dihedral angle γ of **2b**.*

9. Crystallographic Data

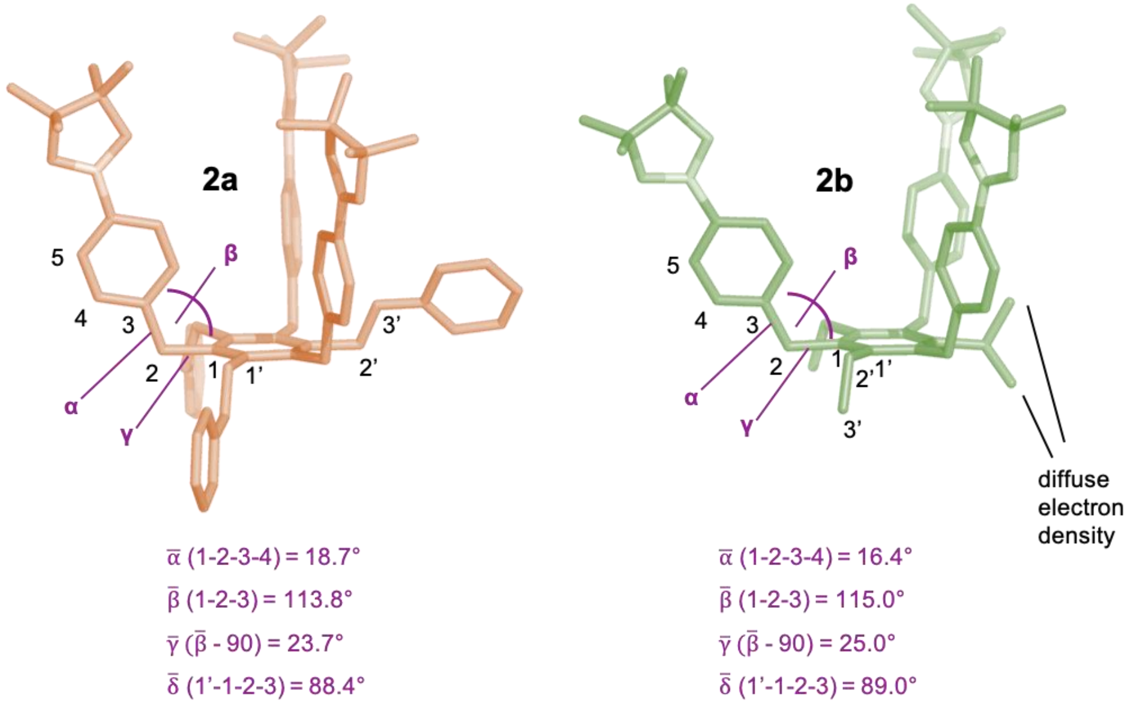

*Figure S109: X-ray crystal structures of Bpin-tripods **2a** and **2b**.*

Single crystals suitable for X-ray analysis of **2a** and **2b** were obtained by slow-cooling of a *n*-pentane/Et_2O mixture.

The data collection for the **2a** and **2b** single crystals were carried out in the SEGAI services of the University of La Laguna on an Agilent Supernova X-ray µ-focus diffractometer at 150 and 100 K, respectively, using CuKα radiation (λ = 1.54184 Å) equipped with an Atlas detector (see Table S26 for details). Data were indexed, integrated and scaled with the Rigaku CrysAlisPro software.[7] The crystal structures were solved by intrinsic phasing with SHELXT software[8] and refined with the full-matrix least squares techniques of *F*[8] by using SHELXL program[9] within the Olex2 software.[10] All non-hydrogen atoms were refined anisotropically. The hydrogen atoms of the organic ligands were set on geometrical positions and refined with a riding model. The high values obtained for wR2 for **2a** are due to the degradation of the crystal during the measurement, leading to poor quality high angle data. A solvent mask implemented within the Olex2 software was used in **2a** to remove the contribution from disordered solvent molecules, the electron density removed accounts for 79 e^- per formula unit, corresponding roughly to a pentane molecule (72 e^-). Some restrictions were also applied in the **2a** structure involving the bond distances and angles of one of the tetramethyl-dioxoborolane atoms (C33 to C38) which were treated as a rigid unit. Most likely, some disorder of this area is contributing to the large anisotropic displacement factors, but a first attempt to model the disorder led to very small occupation factors for the alternative location and therefore, we kept the original refinement.

Deposition numbers 2124239 (compound **2a**) and 2124240 (compound **2b**) contain the supplementary crystallographic data for this paper. These data can be obtained free of charge via http://www.ccdc.cam.ac.uk/data_request/cif, or by emailing data_request@ccdc.cam.ac.uk, or by contacting The Cambridge Crystallographic Data Centre, 12 Union Road, Cambridge CB2 1EZ, UK; fax: +44 1223 336033.

Table S26: Crystallographic and structure refinement details

	**2a**	**2b**
Formula	$C_{147}H_{186}B_6O_{18}$	$C_{102}H_{136}B_6O_{12}$
M (g mol^{-1})	2305.81	1618.96
Crystal system	Monoclinic	Monoclinic
Space group	$I2/a$	$P2_1/c$
a (Å)	25.7430(5)	29.2963(11)
b (Å)	27.0506(4)	14.5054(3)
c (Å)	40.6986(6)	26.0794(7)
β (°)	106.186(2)	107.948(4)
V (Å^3)	27217.6(8)	10543.2(6)
Z	8	4
ρ_{calc} (g cm^{-3})	1.125	1.020
μ (mm^{-1})	0.560	0.499
T (K)	100	150
Reflect. collcd.	58593	42933
Reflect. obs. [$I > 2\sigma(I)$]	26291 (21749)	20339 (10817)
Data Restraints/ Parameters	0/1577	40/1122
R_1 [$I > 2\sigma(I)$] (all data)	0.0821 (0.0956)	0.1309 (0.1785)
wR_2 [$I > 2\sigma(I)$] (all data)	0.2193 (0.2360)	0.3744 (0.4429)
S	1.039	1.400

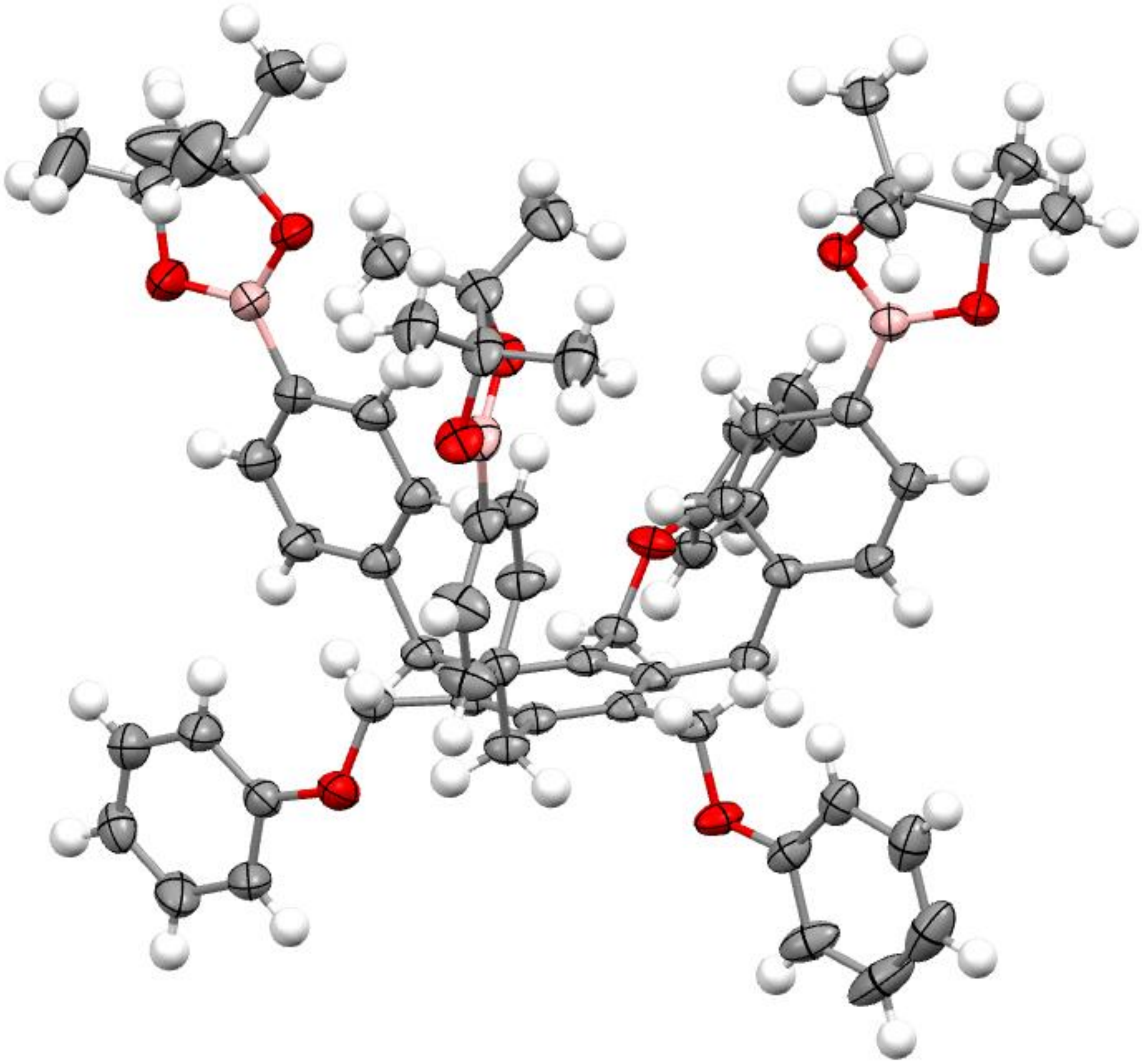

*Figure S110: Thermal ellipsoid plot of the molecular structure of crystalline **2a**. Thermal ellipsoids are shown at the 50% probability level.*

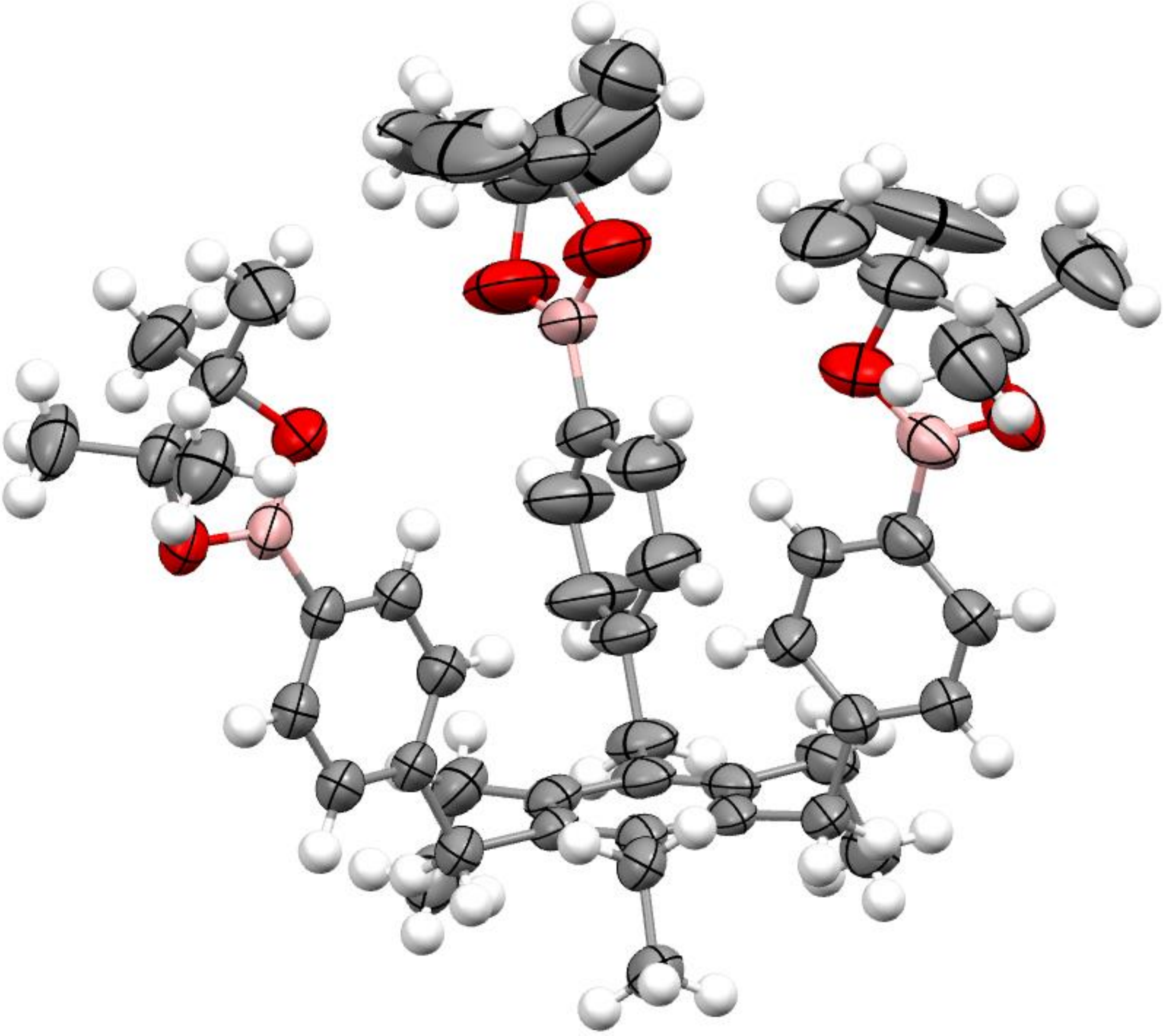

*Figure S111: Thermal ellipsoid plot of the molecular structure of crystalline **2b**. Thermal ellipsoids are shown at the 50% probability level.*

10. Cartesian coordinates and Energies of molecular models based on force field calculations

3a

Potential Energy (OPLS4): -24.869 kJ/mol

C	1.52030	0.81270	-0.25980
C	0.84540	2.03240	-0.05260
C	-0.56250	2.05760	0.00720
C	-1.29670	0.87040	-0.18740
C	-0.62200	-0.34960	-0.39420
C	0.78570	-0.37370	-0.45690
C	3.05930	0.75960	-0.19370
C	1.64150	3.33940	0.07860
C	-1.29030	3.36880	0.36340
C	-2.83200	0.90850	-0.20480
C	-1.42290	-1.66440	-0.47060
C	1.51990	-1.68570	-0.77030
H	3.35800	-0.16490	0.30260
H	3.44010	1.52230	0.48550
H	0.92510	-2.31280	-1.43500
H	2.43140	-1.49160	-1.33580
O	1.80710	-2.35980	0.45090
H	-0.83690	-2.49820	-0.08430
H	-2.24810	-1.60960	0.24100
H	-3.22820	0.15920	-0.88980
O	-3.31650	0.70130	1.11830
H	-3.19230	1.85390	-0.61140
H	-2.21860	3.16530	0.89680
H	-0.70180	3.89930	1.11350
H	1.08060	4.17590	-0.33760
O	1.94800	3.55920	1.45200
H	2.54860	3.30350	-0.52540
C	5.24600	1.03890	-3.92030
C	5.86420	0.56890	-2.75970
C	5.15010	0.48660	-1.56720
C	3.80980	0.87530	-1.52170
C	3.18850	1.34430	-2.68480
C	3.90490	1.42560	-3.87590
B	6.05900	1.13280	-5.27540
H	6.90190	0.26730	-2.78590
H	5.64290	0.11940	-0.67830
H	2.15260	1.64950	-2.67730
H	3.41980	1.79030	-4.77030
C	-3.04920	-2.79200	-4.32140
C	-1.98380	-1.89150	-4.25780
C	-1.44780	-1.52190	-3.02690
C	-1.97270	-2.05400	-1.84390
C	-3.03960	-2.95250	-1.90780
C	-3.57410	-3.31980	-3.13990
B	-3.65730	-3.21120	-5.72170
H	-1.57290	-1.47870	-5.16830
H	-0.62530	-0.82240	-3.00390
H	-3.45880	-3.36860	-1.00280
H	-4.39940	-4.01670	-3.18070
C	-2.13030	6.11910	-2.87310
C	-2.08750	6.56260	-1.54970
C	-1.82080	5.66760	-0.51690
C	-1.59550	4.31830	-0.79650
C	-1.63590	3.87300	-2.12250
C	-1.90330	4.77000	-3.15330
B	-2.43340	7.13750	-4.04660
H	-2.26140	7.60550	-1.32470
H	-1.78890	6.02760	0.50160
H	-1.46160	2.83520	-2.36520
H	-1.93470	4.41970	-4.17530
O	5.53100	1.57790	-6.46650
O	7.38340	0.78160	-5.41050
O	-4.70480	-4.09060	-5.87970
O	-3.20300	-2.74470	-6.93490
O	-2.49750	6.78980	-5.37750
O	-2.66920	8.48160	-3.86460
C	6.61220	1.76970	-7.36030
C	7.67240	0.75450	-6.79620
C	-4.77760	-4.41520	-7.25590
C	-4.14660	-3.13490	-7.91580
C	-2.52770	7.99180	-6.12530
C	-3.16080	8.99200	-5.09020
C	9.14510	1.18210	-6.95860
C	6.08840	1.45590	-8.77640
C	-3.36440	-3.38440	-9.22110
C	-6.26430	-4.66060	-7.58380
C	-3.96390	-5.72350	-7.41920
C	-5.10150	-1.92610	-8.07960
H	-4.01820	-3.77560	-10.00020
H	-2.91370	-2.46460	-9.59520
H	-2.54980	-4.09530	-9.08160
H	-6.40700	-4.82870	-8.65090
H	-6.64790	-5.53360	-7.05470
H	-6.89290	-3.81930	-7.29170
H	-3.99250	-6.08170	-8.44790
H	-2.91710	-5.58670	-7.14490
H	-4.35750	-6.51900	-6.78530
H	-5.84840	-2.11340	-8.85060
H	-5.63110	-1.69970	-7.15330
H	-4.55770	-1.02440	-8.36380
C	6.98970	3.26760	-7.24210
C	7.48570	-0.72050	-7.23170
H	9.41280	1.27150	-8.01110
H	9.34900	2.14020	-6.48020
H	9.82080	0.45730	-6.50330
H	6.89430	1.49430	-9.50890
H	5.63150	0.46790	-8.83480
H	5.32440	2.17070	-9.08370
H	7.79420	3.52840	-7.92910
H	6.14060	3.91180	-7.47370
H	7.31820	3.52440	-6.23420
H	7.72610	-0.85550	-8.28590
H	8.13150	-1.38900	-6.66110
H	6.46080	-1.06130	-7.07920
C	-3.37770	7.72030	-7.38300
C	-1.05620	8.27550	-6.51760
C	-2.67250	10.45060	-5.19960
C	-4.70490	8.94510	-4.97180
H	-3.51960	8.63160	-7.96330
H	-2.90250	6.98320	-8.03110
H	-4.36330	7.32640	-7.13460
H	-0.97690	9.16840	-7.13710
H	-0.42430	8.42310	-5.64090
H	-0.62780	7.44680	-7.08280
H	-2.94000	10.88160	-6.16400
H	-3.11310	11.07510	-4.42170
H	-1.59110	10.53050	-5.08690
H	-5.18270	9.35980	-5.85900
H	-5.07090	7.92550	-4.84440
H	-5.05610	9.51860	-4.11320
C	3.84630	-5.99120	0.56350
C	3.41230	-5.37010	1.73230
C	2.73370	-4.15660	1.65350
C	2.48590	-3.56000	0.41890
C	2.92740	-4.19410	-0.75250
C	3.60660	-5.40840	-0.67890
H	4.37280	-6.93310	0.62130
H	3.59940	-5.82490	2.69420
H	2.39220	-3.66580	2.55260
H	2.75240	-3.76100	-1.72420
H	3.94520	-5.89530	-1.58190
C	-7.38540	0.54260	2.00000
C	-6.42410	0.38380	2.99540
C	-5.07340	0.44360	2.66110
C	-4.67670	0.66010	1.34310
C	-5.65330	0.81900	0.34800
C	-7.00580	0.76010	0.67740

H	-8.43430	0.49700	2.25630
H	-6.72170	0.21560	4.02020
H	-4.31920	0.32200	3.42410
H	-5.38200	0.98870	-0.68160
H	-7.75620	0.88300	-0.09000
C	4.01380	6.93170	2.76220
C	3.57930	5.95770	3.65810
C	2.89200	4.84420	3.18140
C	2.63600	4.69660	1.81970
C	3.07860	5.68340	0.92560
C	3.76660	6.79920	1.39770
H	4.54760	7.79680	3.12900
H	3.77320	6.06290	4.71560
H	2.55040	4.08250	3.86620
H	2.89870	5.60060	-0.13430
H	4.10690	7.55780	0.70780

3b

Potential Energy (OPLS4): -46.550 kJ/mol

C	1.67150	0.80000	0.78890
C	0.90550	1.98540	0.76060
C	-0.49390	1.92510	0.93450
C	-1.12720	0.68030	1.14050
C	-0.36070	-0.50460	1.16840
C	1.03900	-0.44480	0.99570
C	1.60150	3.34870	0.58580
C	-2.64780	0.61940	1.38050
C	1.88170	-1.73180	1.07920
C	-1.79340	5.46340	4.56060
C	-2.12630	6.03080	3.32880
C	-1.97550	5.29850	2.15400
C	-1.49060	3.98950	2.19800
C	-1.15630	3.42060	3.43200
C	-1.30800	4.15470	4.60520
B	-1.96410	6.29740	5.89530
H	-2.50210	7.04320	3.28680
H	-2.23680	5.75290	1.20950
H	-0.77870	2.41010	3.49200
H	-1.04760	3.70760	5.55410
C	5.71120	1.12650	4.09770
C	4.33570	1.33900	4.21250
C	3.51570	1.25900	3.09010
C	4.06500	0.96520	1.83700
C	5.44050	0.75180	1.72310
C	6.25860	0.83250	2.84700
B	6.64210	1.21760	5.37510
H	3.90290	1.56670	5.17630
H	2.45460	1.42780	3.20400
H	5.87990	0.52240	0.76340
H	7.32210	0.66550	2.75060
C	-1.74110	-3.18770	5.44090
C	-0.86980	-2.12420	5.19650
C	-0.63850	-1.68970	3.89420
C	-1.27800	-2.31730	2.81920
C	-2.14990	-3.38040	3.06440
C	-2.37950	-3.81320	4.36790
B	-2.00330	-3.68030	6.92240
H	-0.37120	-1.63420	6.02080
H	0.04020	-0.86520	3.73020
H	-2.65240	-3.87470	2.24600
H	-3.05550	-4.63660	4.54980
H	-2.89240	-0.18190	2.07700
H	-2.97860	1.49660	1.93600
C	-3.46550	0.46560	0.08420
H	1.08160	4.11840	1.15520
H	2.57630	3.34130	1.07300
C	1.74150	3.77900	-0.88650
H	1.45160	-2.41920	1.80740
C	2.06160	-2.43400	-0.27980
H	2.85290	-1.52330	1.52690
C	3.20040	0.86090	0.57860
C	-1.33200	3.22150	0.88380
C	-1.05830	-1.86740	1.37310
H	-2.32710	3.03020	0.49010
H	-0.91650	3.89600	0.13810
H	3.47180	1.68310	-0.07890
H	3.52660	-0.00590	0.00780
H	-0.52520	-2.66180	0.85670
H	-2.01880	-1.86650	0.86230
H	2.24170	4.74480	-0.96180
H	2.65980	-3.33900	-0.17170
H	-4.53320	0.43110	0.30190
H	-3.20990	-0.44900	-0.45000
H	-3.29720	1.29470	-0.60260
H	0.77220	3.87280	-1.37530
H	2.32330	3.06220	-1.46530
H	2.56440	-1.79330	-1.00360
H	1.10630	-2.72240	-0.71730
O	-1.65800	5.82260	7.15090
O	-2.43280	7.59140	5.94610
O	8.00070	1.01210	5.36050
O	6.18800	1.47650	6.65050
O	-2.84190	-4.72030	7.25680
O	-1.42230	-3.12240	8.03920
C	-2.18160	6.74460	8.08890
C	-2.18650	8.07660	7.25310
C	8.47690	1.43570	6.62020
C	7.25040	1.20650	7.54590
C	-2.61090	-5.02480	8.62000
C	-2.09660	-3.64400	9.16930
C	-1.23610	6.73450	9.30700
C	-3.57570	6.19420	8.48070
C	-3.32390	9.06180	7.59050
C	-0.83070	8.82180	7.17230
C	-1.57240	-6.17440	8.62020
C	-3.95180	-5.50930	9.20770
C	-3.19610	-2.60830	9.51350
C	-1.06290	-3.73220	10.31020
C	8.79670	2.93880	6.51980
C	9.76300	0.66090	6.95640
C	7.04880	-0.24080	8.05020
C	7.15000	2.23010	8.68910
H	-3.75080	-2.90090	10.40450
H	-2.76930	-1.62260	9.70280
H	-3.91300	-2.49300	8.69950
H	-1.49120	-4.21090	11.19040
H	-0.17850	-4.29920	10.01900
H	-0.71390	-2.74220	10.60560
H	-1.35600	-6.51220	9.63330
H	-1.93260	-7.03670	8.05800
H	-0.62980	-5.86860	8.16440
H	-3.87050	-5.67730	10.28120
H	-4.75500	-4.79100	9.04240
H	-4.27130	-6.44460	8.74710
H	-1.52850	7.48990	10.03580
H	-1.24620	5.76600	9.80820
H	-0.20110	6.92680	9.02400
H	-4.04920	6.81820	9.23810
H	-4.24780	6.14610	7.62290
H	-3.50530	5.18420	8.88630
H	-3.24830	9.40860	8.62080
H	-3.29540	9.93730	6.94100
H	-4.30810	8.61100	7.46180
H	-0.56870	9.27220	8.12930
H	-0.01740	8.15310	6.88710
H	-0.85930	9.62160	6.43140
H	7.81280	-0.51730	8.77620
H	6.07780	-0.36080	8.53180
H	7.09270	-0.96160	7.23270
H	7.96500	2.10460	9.40170
H	7.18810	3.25440	8.31650
H	6.21140	2.12300	9.23420
H	10.13750	0.93450	7.94250
H	9.60210	-0.41750	6.94520
H	10.54830	0.87900	6.23210
H	9.16780	3.29680	7.48020
H	9.55640	3.09880	5.75480
H	7.89260	3.48620	6.25340

3c

Potential Energy (OPLS4): -39.341 kJ/mol

C	1.49870	0.80860	-0.10160
C	0.82230	2.03080	0.08570
C	-0.58600	2.05720	0.13090
C	-1.31890	0.86890	-0.06230
C	-0.64280	-0.35320	-0.25190
C	0.76580	-0.37940	-0.29520
C	3.03680	0.75520	-0.01740
C	1.61730	3.33910	0.20930
C	-1.31640	3.37140	0.47010
C	-2.85400	0.90760	-0.09610
C	-1.44490	-1.66710	-0.33190
C	1.50280	-1.69590	-0.58250
H	3.32660	-0.16010	0.50080
H	3.41360	1.53010	0.64990
H	0.91840	-2.32840	-1.25130
H	2.42480	-1.51090	-1.13360
O	1.76680	-2.35790	0.65050
H	-0.86570	-2.50210	0.06150
H	-2.27640	-1.60800	0.37200
H	-3.24190	0.15720	-0.78460
O	-3.35250	0.70210	1.22200
H	-3.21130	1.85190	-0.50760
H	-2.24940	3.17250	0.99690
H	-0.73420	3.90750	1.22120
H	1.06030	4.17110	-0.22140
O	1.91260	3.57610	1.58220
H	2.52930	3.29580	-0.38680
C	5.25370	0.95140	-3.73140
C	5.84790	0.45910	-2.56750
C	5.12500	0.40420	-1.37870
C	3.79990	0.84260	-1.34040
C	3.20250	1.33340	-2.50700
C	3.92760	1.38760	-3.69430
B	6.07680	1.01500	-5.08210
H	6.87370	0.11900	-2.58820
H	5.59910	0.01930	-0.48710
H	2.17830	1.67610	-2.50470
H	3.46120	1.76960	-4.59140
C	-3.03750	-2.78670	-4.19910
C	-1.94660	-1.91820	-4.12530
C	-1.42100	-1.55260	-2.88890
C	-1.98220	-2.05660	-1.71030
C	-3.07430	-2.92350	-1.78430
C	-3.59830	-3.28700	-3.02210
B	-3.63400	-3.20020	-5.60600
H	-1.50770	-1.52670	-5.03220
H	-0.57860	-0.87750	-2.85790
H	-3.52180	-3.31740	-0.88280
H	-4.44350	-3.95910	-3.07070
C	-2.12780	6.09610	-2.79540
C	-2.08700	6.55200	-1.47600
C	-1.82930	5.66530	-0.43390
C	-1.61090	4.31200	-0.69990
C	-1.64860	3.85450	-2.02170
C	-1.90730	4.74320	-3.06190
B	-2.42130	7.10480	-3.97960
H	-2.25580	7.59790	-1.26150
H	-1.79910	6.03470	0.58130
H	-1.47930	2.81340	-2.25380
H	-1.93680	4.38330	-4.08070
O	5.57400	1.48540	-6.27450
O	7.38680	0.61150	-5.21130
O	-4.70610	-4.04750	-5.77470
O	-3.14610	-2.75740	-6.81510
O	-2.48370	6.74430	-5.30710
O	-2.64580	8.45280	-3.81200
C	6.66740	1.63790	-7.16090
C	7.68240	0.57820	-6.59550
C	-4.76710	-4.38000	-7.14960
C	-4.08580	-3.12500	-7.80820
C	-2.50000	7.93870	-6.06710

C	-3.12750	8.95550	-5.04480
C	9.17220	0.94630	-6.74760
C	6.13990	1.35240	-8.58150
C	-3.29090	-3.40840	-9.09880
C	-6.25530	-4.58140	-7.49970
C	-3.99230	-5.71410	-7.29060
C	-4.99950	-1.88800	-7.99560
H	-3.94390	-3.78480	-9.88580
H	-2.80570	-2.50580	-9.47160
H	-2.50130	-4.14320	-8.94100
H	-6.38630	-4.75310	-8.56780
H	-6.67440	-5.43800	-6.97070
H	-6.86190	-3.71870	-7.22380
H	-4.01660	-6.07950	-8.31680
H	-2.94590	-5.60760	-7.00150
H	-4.41960	-6.49180	-6.65650
H	-5.73910	-2.05710	-8.77770
H	-5.53670	-1.63860	-7.07970
H	-4.42290	-1.00580	-8.27650
C	7.10430	3.11880	-7.03290
C	7.43840	-0.88610	-7.03850
H	9.44980	1.02810	-7.79810
H	9.41180	1.89380	-6.26460
H	9.81510	0.19320	-6.29070
H	6.95050	1.36450	-9.30960
H	5.64600	0.38290	-8.64840
H	5.40510	2.09750	-8.88850
H	7.92320	3.35010	-7.71350
H	6.28350	3.79790	-7.26640
H	7.43650	3.35720	-6.02160
H	7.68040	-1.02660	-8.09160
H	8.05240	-1.58280	-6.46640
H	6.39950	-1.18530	-6.89420
C	-3.34790	7.66150	-7.32500
C	-1.02440	8.20490	-6.45650
C	-2.62450	10.40810	-5.16740
C	-4.67240	8.92510	-4.93210
H	-3.47860	8.56740	-7.91630
H	-2.87750	6.91230	-7.96270
H	-4.33830	7.28010	-7.07610
H	-0.93450	9.09040	-7.08500
H	-0.39480	8.35620	-5.57870
H	-0.60120	7.36630	-7.01080
H	-2.88340	10.83130	-6.13760
H	-3.06250	11.04520	-4.39830
H	-1.54300	10.47870	-5.05060
H	-5.14260	9.33550	-5.82530
H	-5.04890	7.91060	-4.79570
H	-5.02120	9.51090	-4.08080
C	3.77070	-6.01390	0.83740
C	3.31720	-5.37080	1.99010
C	2.65270	-4.15160	1.88680
C	2.43520	-3.56430	0.64310
C	2.89490	-4.21260	-0.51240
C	3.56030	-5.43250	-0.41410
C	4.49700	-7.34590	0.94570
H	3.47840	-5.81050	2.96340
H	2.29900	-3.64970	2.77470
H	2.74570	-3.78660	-1.49130
H	3.91210	-5.92270	-1.31010
C	-7.43690	0.50490	2.05610
C	-6.47930	0.35680	3.06060
C	-5.12560	0.42990	2.74290
C	-4.71490	0.64850	1.43040
C	-5.68050	0.79610	0.42410
C	-7.03590	0.72360	0.73710
C	-8.91690	0.42310	2.39730
H	-6.77910	0.18370	4.08370
H	-4.38010	0.31600	3.51530
H	-5.39930	0.96660	-0.60250
H	-7.77170	0.83570	-0.04570
C	4.00070	6.95580	2.86120
C	3.55170	5.99100	3.76410
C	2.85760	4.87710	3.29890
C	2.60640	4.71360	1.93900
C	3.06030	5.68530	1.03520

C	3.75480	6.80130	1.49590
C	4.76380	8.17220	3.36300
H	3.74050	6.09900	4.82210
H	2.50830	4.12700	3.99230
H	2.88540	5.59100	-0.02430
H	4.10340	7.54210	0.79120
H	4.26980	-7.94950	0.06520
C	6.01890	-7.17670	1.10150
H	4.08910	-7.90670	1.78870
H	-9.46290	1.11100	1.75050
C	-9.47200	-1.00870	2.26610
H	-9.07240	0.79880	3.41010
H	4.50320	9.02890	2.74040
H	4.41220	8.42850	4.36390
C	6.28830	7.94610	3.37260
C	6.76000	-8.52070	1.21490
H	6.40920	-6.61520	0.25060
H	6.22650	-6.57010	1.98500
C	-10.95210	-1.15360	2.67190
H	-9.33660	-1.36360	1.24280
H	-8.87440	-1.67590	2.89000
C	7.10060	9.12840	3.93710
H	6.50820	7.05820	3.96830
H	6.63250	7.71480	2.36300
C	8.28070	-8.35040	1.37140
H	6.36840	-9.08060	2.06630
H	6.55180	-9.12630	0.33090
H	-11.20390	-2.21570	2.68260
H	-11.08440	-0.80860	3.69900
C	-11.93620	-0.41690	1.74380
H	8.14160	8.81510	4.03560
C	7.04280	10.40300	3.07420
H	6.76290	9.35280	4.95050
C	9.01810	-9.69190	1.48610
H	8.67810	-7.79360	0.52070
H	8.49420	-7.74660	2.25520
C	-13.40280	-0.65980	2.12670
H	-11.73990	0.65580	1.76490
H	-11.77700	-0.73540	0.71210
C	7.93820	11.52510	3.61800
H	7.34290	10.16830	2.05140
H	6.01700	10.76880	3.01410
H	10.09220	-9.53910	1.59580
H	8.67640	-10.26020	2.35210
H	8.86210	-10.30810	0.59970
H	-14.07560	-0.12610	1.45460
H	-13.65730	-1.71910	2.07400
H	-13.61030	-0.31670	3.14100
H	7.87600	12.41400	2.98930
H	7.64310	11.81440	4.62740
H	8.98430	11.21810	3.65110

Ta

Potential Energy (OPLS4): 2008.357 kJ/mol

C	-0.50610	0.03700	0.20530
C	0.81050	-0.46560	0.18990
C	1.90480	0.42230	0.21610
C	1.68200	1.81380	0.20040
C	0.36590	2.31720	0.21860
C	-0.72760	1.42870	0.19110
C	-1.69880	-0.92960	0.26610
C	1.05650	-1.98020	0.04020
C	3.33750	-0.12770	0.28990
C	2.87110	2.78430	0.05680
C	0.12370	3.83240	0.29640
C	-2.16120	1.97590	0.04260
O	-2.10140	-1.25960	-1.06060
O	3.83460	-0.31890	-1.03190
O	0.03510	4.36120	-1.02400
C	1.67040	-4.32140	3.63480
C	1.95670	-4.84880	2.37150
C	1.75260	-4.08520	1.22670
C	1.25840	-2.78350	1.32760
C	0.97350	-2.25010	2.58990
C	1.17790	-3.01570	3.73460
C	4.56810	4.49950	3.65620
C	4.88690	5.00890	2.39330
C	4.33540	4.44580	1.24700
C	3.45780	3.36460	1.34600
C	3.13330	2.85310	2.60770
C	3.68650	3.41760	3.75410
C	-4.49600	2.64110	3.63220
C	-3.14240	2.30440	3.74000
C	-2.37610	2.09010	2.59740
C	-2.95650	2.21040	1.32940
C	-4.30710	2.54650	1.22040
C	-5.07110	2.76100	2.36300
C	-5.32150	-3.78760	-1.84510
C	-4.58350	-3.21690	-2.87920
C	-3.51390	-2.37710	-2.57850
C	-3.17640	-2.10360	-1.25330
C	-3.92680	-2.68460	-0.21870
C	-4.99760	-3.52500	-0.51600
C	7.61960	-1.89760	-1.77820
C	6.76880	-1.53910	-2.82110
C	5.51130	-1.01420	-2.53300
C	5.09780	-0.84500	-1.21200
C	5.96390	-1.20880	-0.16850
C	7.22240	-1.73450	-0.45310
C	-0.60050	8.41500	-1.75780
C	-0.46450	7.50500	-2.80340
C	-0.25290	6.15800	-2.51950
C	-0.17650	5.71370	-1.19990
C	-0.31490	6.63970	-0.15360
C	-0.52660	7.98790	-0.43400
B	1.90240	-5.19970	4.93850
O	2.38590	-6.50650	4.89270
B	5.20380	5.14570	4.96140
O	4.93720	4.68450	6.25060
B	-5.37490	2.88430	4.93340
O	-6.72530	3.22590	4.87950
O	1.63260	-4.74050	6.22770
O	-4.87150	2.76690	6.22900
O	6.08950	6.22130	4.91670
C	1.96180	-5.81210	6.99480
C	2.40720	-6.85700	6.20370
C	2.80850	-8.08140	6.74160
C	1.88420	-5.91250	8.38620
C	6.37640	6.41900	6.22840
C	5.69450	5.51000	7.01890
C	5.81500	5.49660	8.41080
C	7.23060	7.38300	6.76740
C	-5.96010	3.04600	6.99210
C	-7.05650	3.31870	6.19230
C	-8.30700	3.63860	6.72440
C	-6.03190	3.07120	8.38720
C	2.73000	-8.18170	8.13200
C	2.28530	-7.13770	8.92490
O	2.30450	-7.49330	10.23580
B	2.78040	-8.80350	10.18460
O	3.05510	-9.25600	8.89490
C	6.67030	6.46060	8.95050
C	7.35170	7.36850	8.15830
O	8.11420	8.19120	8.92220
B	7.85770	7.72800	10.21170
O	6.96350	6.65850	10.26200
C	-8.37820	3.66420	8.11850
C	-7.28340	3.39040	8.92020
O	-7.61990	3.48110	10.23320
B	-8.97050	3.82510	10.17410
O	-9.46760	3.94530	8.87730
H	1.92010	-2.13670	-0.60750
H	0.24940	-2.44280	-0.52780
H	2.57750	3.60860	-0.59450
H	3.67910	2.31600	-0.50540
H	-2.15300	2.90450	-0.52850
H	-2.73450	1.30810	-0.60160
H	-2.52160	-0.48740	0.82810
H	-1.43940	-1.82480	0.83220
H	3.98030	0.54600	0.85740
H	3.35940	-1.05850	0.85700
H	-0.78060	4.04590	0.86740
H	0.91820	4.31990	0.86170
H	2.33900	-5.85600	2.27370
H	1.98050	-4.51040	0.25910
H	0.59240	-1.24420	2.69420
H	0.95140	-2.59070	4.70310
H	5.56590	5.84550	2.29700
H	4.59350	4.85420	0.27980
H	2.45450	2.01840	2.71020
H	3.42770	3.01060	4.72220
H	-2.68020	2.20740	4.71310
H	-1.33290	1.83060	2.70860
H	-4.77010	2.64180	0.24800
H	-6.11610	3.02030	2.25890
H	-6.15200	-4.43950	-2.07590
H	-4.83780	-3.42340	-3.90860
H	-2.93690	-1.93050	-3.37460
H	-3.69380	-2.49560	0.81730
H	-5.57390	-3.97090	0.28150
H	8.59580	-2.30510	-1.99920
H	7.08070	-1.66670	-3.84740
H	4.84570	-0.73410	-3.33600
H	5.67780	-1.09070	0.86470
H	7.88720	-2.01410	0.35130
H	-0.76500	9.46070	-1.97560
H	-0.52280	7.84020	-3.82870
H	-0.14660	5.44620	-3.32460
H	-0.26130	6.33150	0.87860
H	-0.63310	8.69880	0.37250
H	3.15470	-8.89600	6.12400
H	1.53640	-5.09850	9.00360
H	5.28430	4.78770	9.02770
H	7.76270	8.09060	6.15030
H	-9.16130	3.85150	6.10010
H	-5.17770	2.85820	9.01150
C	3.35350	-10.64050	18.99990
C	2.18980	-11.15780	18.39620
C	2.22830	-11.60820	17.06130
C	3.44400	-11.59250	16.34860
C	4.60750	-11.07300	16.95060
C	4.56860	-10.62540	18.28630
C	3.29160	-10.06640	20.42370
C	0.90060	-11.32510	19.22490
C	0.93990	-12.08820	16.37550
C	3.52690	-12.23810	14.95110
C	5.90980	-10.96960	16.14220
C	5.87280	-10.21650	18.99930
C	-2.04420	-8.14780	19.10660
C	-2.33190	-9.37780	19.70710
C	-1.37510	-10.38730	19.73810

C	-0.11640	-10.18200	19.17010
C	0.17530	-8.95360	18.56600
C	-0.78320	-7.94430	18.53600
C	2.97780	-9.70940	11.47510
C	3.44060	-11.02300	11.34750
C	3.61100	-11.82210	12.47340
C	3.32100	-11.32020	13.74350
C	2.86170	-10.00480	13.87670
C	2.69070	-9.20660	12.74870
C	7.02850	-6.03990	18.96740
C	7.77250	-6.99100	19.67290
C	7.38870	-8.32820	19.67110
C	6.25620	-8.73480	18.96330
C	5.50940	-7.78690	18.25400
C	5.89440	-6.44880	18.25750
O	3.56310	-11.10620	21.35980
O	0.79050	-13.48950	16.58750
O	6.65850	-12.17130	16.30500
C	3.62480	-10.43600	25.47340
C	3.87480	-11.69990	24.94450
C	3.84550	-11.88570	23.56440
C	3.56870	-10.81910	22.70980
C	3.31820	-9.54930	23.25390
C	3.34670	-9.35980	24.63390
C	-2.49120	-15.61900	15.14840
C	-1.48180	-16.25050	15.87100
C	-0.40010	-15.50620	16.33560
C	-0.31950	-14.13700	16.08380
C	-1.34230	-13.51090	15.35370
C	-2.42590	-14.25230	14.88770
C	10.32490	-12.78080	14.41800
C	9.76580	-13.75580	15.24050
C	8.53880	-13.51640	15.85450
C	7.86680	-12.31120	15.65280
C	8.43970	-11.33520	14.82190
C	9.66700	-11.57140	14.20610
C	-11.83900	3.34310	17.00250
C	-11.41190	3.22560	18.34020
C	-10.82920	4.32980	18.99380
C	-10.72810	5.56800	18.32840
C	-11.16000	5.68790	16.99210
C	-11.74130	4.58250	16.33930
C	-12.38400	2.11430	16.25880
C	-11.67680	1.92170	19.11890
C	-10.27850	4.17690	20.42000
C	-10.23750	6.81340	19.09340
C	-10.97020	7.01310	16.23810
C	-12.36210	4.75610	14.93890
C	-8.65830	-1.18680	19.04000
C	-9.90160	-1.40120	19.64360
C	-10.85980	-0.39270	19.65980
C	-10.58970	0.84490	19.07290
C	-9.34820	1.06290	18.46430
C	-8.38990	0.05290	18.44980
C	-5.99660	7.70690	19.07430
C	-6.88960	8.45440	19.84880
C	-8.24810	8.15430	19.84420
C	-8.73420	7.10240	19.06550
C	-7.84370	6.34920	18.29150
C	-6.48460	6.65150	18.29650
C	-9.86550	4.06690	11.46440
C	-11.20980	4.42850	11.32930
C	-11.99760	4.64470	12.45530
C	-11.45380	4.50210	13.73310
C	-10.10950	4.13770	13.87350
C	-9.32210	3.92280	12.74550
O	-13.79640	2.04690	16.43750
O	-11.30780	4.49000	21.35460
O	-12.12500	7.82700	16.42470
C	-16.11220	-0.97220	14.73530
C	-16.69040	0.03140	15.50880
C	-15.88450	1.02370	16.06180
C	-14.50650	1.02080	15.84770
C	-13.93420	0.00410	15.06660
C	-14.73740	-0.99010	14.51200
C	-10.68610	4.36500	25.47440

C	-11.92250	4.72220	24.94170
C	-12.09280	4.75270	23.55970
C	-11.03800	4.42930	22.70680
C	-9.79600	4.07050	23.25450
C	-9.62220	4.03890	24.63650
C	-12.47850	11.61250	14.71590
C	-13.49670	11.07740	15.50130
C	-13.34280	9.80910	16.05590
C	-12.18040	9.07200	15.83160
C	-11.16050	9.62110	15.03820
C	-11.31110	10.88960	14.48200
C	9.16050	9.09330	19.02180
C	10.19530	8.35820	18.40970
C	10.56630	8.63630	17.07890
C	9.93800	9.68570	16.37840
C	8.90350	10.42180	16.99010
C	8.53650	10.14550	18.32230
C	8.69400	8.73070	20.43990
C	10.98860	7.31920	19.22710
C	11.63460	7.77800	16.38400
C	10.44870	10.09510	14.98260
C	8.15860	11.50550	16.19560
C	7.52960	11.05890	19.04940
C	9.69960	3.18290	19.11590
C	10.85130	3.56320	19.81240
C	11.25030	4.89570	19.83960
C	10.50350	5.86810	19.17200
C	9.34930	5.49250	18.47480
C	8.95190	4.15840	18.44780
C	8.54050	8.35370	11.50300
C	9.44530	9.41270	11.37690
C	10.04980	9.96060	12.50390
C	9.75900	9.45800	13.77340
C	8.85080	8.40100	13.90490
C	8.24750	7.85310	12.77610
C	3.34220	9.94300	19.04020
C	3.80620	11.00450	19.82380
C	5.15360	11.35040	19.81460
C	6.05720	10.64090	19.02150
C	5.59830	9.57630	18.23730
C	4.24930	9.23150	18.24750
O	9.45450	9.47590	21.38730
O	12.91650	8.36080	16.60310
O	8.82210	12.75430	16.37210
C	8.83170	9.14330	25.49540
C	9.80150	9.99960	24.97960
C	9.98100	10.08420	23.60100
C	9.19950	9.32020	22.73480
C	8.22500	8.46020	23.26560
C	8.04270	8.37320	24.64420
C	16.41940	6.65980	15.11620
C	16.44840	7.83540	15.86260
C	15.25730	8.37440	16.34280
C	14.03860	7.74820	16.08320
C	14.02100	6.56380	15.32950
C	15.21070	6.02180	14.84770
C	7.51610	16.25020	14.51520
C	8.63680	16.24770	15.34200
C	9.04320	15.06020	15.94580
C	8.33880	13.87620	15.72970
C	7.21020	13.89030	14.89480
C	6.80100	15.07640	14.28910
B	-7.56600	-2.34080	19.02910
O	-6.30100	-2.19040	18.46120
O	-7.78480	-3.59360	19.60020
C	-5.72720	-3.39800	18.70080
C	-6.60640	-4.22820	19.37460
C	-6.26670	-5.53070	19.74550
C	-4.43940	-3.80800	18.34680
C	-4.97950	-5.94000	19.39190
C	-4.09880	-5.11100	18.71850
O	-2.91910	-5.74720	18.49860
B	-3.13980	-6.99750	19.07630
O	-4.40820	-7.14690	19.63530
B	9.25050	1.65900	19.08640
O	8.12400	1.20690	18.39900

O	9.95380	0.65140	19.74480
C	8.14430	-0.12800	18.64970
C	9.22720	-0.45560	19.44740
C	9.48280	-1.76570	19.85680
C	7.23450	-1.08780	18.19890
C	8.57280	-2.72430	19.40700
C	7.49020	-2.39910	18.60790
O	6.76620	-3.50960	18.31150
B	7.47010	-4.51340	18.97690
O	8.59450	-4.05690	19.66290
B	1.80130	9.55510	19.05340
O	1.26860	8.51190	18.29590
O	0.85760	10.23700	19.82010
C	-0.05130	8.56470	18.61280
C	-0.29360	9.58660	19.51460
C	-1.57180	9.86140	20.00480
C	-1.07060	7.73840	18.13280
C	-2.59020	9.03610	19.52420
C	-2.34980	8.01320	18.62300
O	-3.50440	7.36630	18.31750
B	-4.44670	8.05740	19.07940
O	-3.90910	9.09520	19.83910
H	0.40160	-12.24840	18.92780
H	1.14530	-11.51540	20.27000
H	4.48950	-12.74010	14.84610
H	2.82050	-13.06500	14.87550
H	6.70890	-10.79680	18.60830
H	5.83090	-10.53590	20.04120
H	-3.30140	-9.55450	20.15310
H	-1.61640	-11.33150	20.20630
H	1.14150	-8.77040	18.11790
H	-0.54270	-6.99990	18.06680
H	3.66870	-11.42960	10.37140
H	3.96900	-12.83530	12.35430
H	2.63250	-9.59250	14.84900
H	2.33260	-8.19290	12.86700
H	8.65240	-6.69450	20.22780
H	7.97550	-9.04830	20.22430
H	4.62890	-8.07600	17.69790
H	5.30670	-5.72750	17.70570
H	3.99720	-9.24290	20.53600
H	2.31830	-9.61210	20.61150
H	0.95930	-11.85250	15.31110
H	0.07840	-11.53850	16.75540
H	6.48820	-10.10010	16.45640
H	5.69280	-10.78120	15.09050
H	3.64690	-10.28930	26.54400
H	4.08980	-12.53190	25.59900
H	4.03810	-12.86270	23.14650
H	3.10060	-8.70200	22.62300
H	3.15360	-8.38200	25.05060
H	-3.33170	-16.19440	14.78690
H	-1.53590	-17.31080	16.07060
H	0.38640	-15.98840	16.89690
H	-1.31220	-12.45420	15.13960
H	-3.21200	-13.76840	14.32640
H	11.27700	-12.96410	13.94050
H	10.27940	-14.69220	15.40250
H	8.09860	-14.26730	16.49360
H	7.94830	-10.39170	14.64390
H	10.10620	-10.81910	13.56720
H	-11.88830	2.14920	20.16400
H	-12.61470	1.48100	18.77890
H	-10.76310	7.69970	18.73720
H	-10.55880	6.75140	20.13370
H	-12.78220	5.75820	14.84650
H	-13.24370	4.12130	14.84520
H	-10.12830	-2.35300	20.10480
H	-11.81450	-0.57700	20.13240
H	-9.11500	2.01140	18.00190
H	-7.43420	0.23690	17.97790
H	-6.53150	9.27410	20.45720
H	-8.92300	8.74450	20.44840
H	-8.19410	5.52920	17.68110
H	-5.80920	6.06190	17.69150
H	-11.64840	4.54460	10.34730
H	-13.03420	4.92530	12.33050
H	-9.66480	4.01990	14.85140
H	-8.28460	3.64320	12.86960
H	-12.12300	2.16190	15.20100
H	-11.89930	1.20450	16.61420
H	-9.40790	4.81740	20.56410
H	-9.89750	3.16770	20.57850
H	-10.78660	6.82880	15.17920
H	-10.06960	7.52360	16.58060
H	-16.73550	-1.74320	14.30510
H	-17.75700	0.04180	15.67960
H	-16.32510	1.80540	16.66250
H	-12.87230	-0.02960	14.88070
H	-14.29510	-1.77150	13.91120
H	-10.55150	4.34060	26.54640
H	-12.74500	4.97450	25.59490
H	-13.04820	5.02870	23.13890
H	-8.95870	3.81410	22.62490
H	-8.66580	3.76250	25.05600
H	-12.59550	12.59650	14.28460
H	-14.40060	11.64130	15.68020
H	-14.12760	9.38710	16.66600
H	-10.24830	9.07950	14.84380
H	-10.52510	11.31040	13.87190
H	12.03620	7.34390	18.92520
H	11.03760	7.62530	20.27240
H	10.39210	11.17980	14.88370
H	11.51960	9.90730	14.90340
H	7.60280	12.07730	18.66730
H	7.83690	11.17450	20.08950
H	11.44280	2.82370	20.33530
H	12.14460	5.16990	20.38180
H	8.75360	6.22580	17.95010
H	8.05810	3.88330	17.90420
H	9.68430	9.81390	10.40120
H	10.74730	10.77820	12.38590
H	8.60740	7.99540	14.87650
H	7.54990	7.03490	12.89330
H	3.12060	11.56640	20.44380
H	5.49310	12.17390	20.42730
H	6.27820	9.01090	17.61600
H	3.90900	8.40730	17.63520
H	7.62700	8.92410	20.55320
H	8.79190	7.65850	20.61280
H	11.42150	7.68650	15.31850
H	11.60050	6.75230	16.75210
H	7.11680	11.56610	16.51190
H	8.10280	11.23500	15.14080
H	8.69050	9.07590	26.56480
H	10.41160	10.59500	25.64320
H	10.73080	10.74570	23.19320
H	7.60300	7.85460	22.62550
H	7.29220	7.71080	25.05060
H	17.34230	6.23960	14.74250
H	17.38780	8.32740	16.06840
H	15.27130	9.28560	16.92230
H	13.09690	6.05320	15.10920
H	15.19510	5.11030	14.26800
H	7.19870	17.17040	14.04560
H	9.18840	17.16030	15.51520
H	9.91120	15.05010	16.58820
H	6.64130	12.99360	14.70590
H	5.93230	15.08510	13.64680
H	-6.95250	-6.17730	20.27130
H	-3.75300	-3.16110	17.82220
H	10.32690	-2.02020	20.47940
H	6.39030	-0.83310	17.57660
H	-1.76010	10.65900	20.70710
H	-0.88260	6.94180	17.42940
C	-0.50610	0.03700	0.20530
C	0.81050	-0.46560	0.18990
C	1.90480	0.42230	0.21610
C	1.68200	1.81380	0.20040
C	0.36590	2.31720	0.21860
C	-0.72760	1.42870	0.19110
C	-1.69880	-0.92960	0.26610

C	1.05650	-1.98020	0.04020
C	3.33750	-0.12770	0.28990
C	2.87110	2.78430	0.05680
C	0.12370	3.83240	0.29640
C	-2.16120	1.97590	0.04260
O	-2.10140	-1.25960	-1.06060
O	3.83460	-0.31890	-1.03190
O	0.03510	4.36120	-1.02400
C	1.67040	-4.32140	3.63480
C	1.95670	-4.84880	2.37150
C	1.75260	-4.08520	1.22670
C	1.25840	-2.78350	1.32760
C	0.97350	-2.25010	2.58990
C	1.17790	-3.01570	3.73460
C	4.56810	4.49950	3.65620
C	4.88690	5.00890	2.39330
C	4.33540	4.44580	1.24700
C	3.45780	3.36460	1.34600
C	3.13330	2.85310	2.60770
C	3.68650	3.41760	3.75410
C	-4.49600	2.64110	3.63220
C	-3.14240	2.30440	3.74000
C	-2.37610	2.09010	2.59740
C	-2.95650	2.21040	1.32940
C	-4.30710	2.54650	1.22040
C	-5.07110	2.76100	2.36300
C	-5.32150	-3.78760	-1.84510
C	-4.58350	-3.21690	-2.87920
C	-3.51390	-2.37710	-2.57850
C	-3.17640	-2.10360	-1.25330
C	-3.92680	-2.68460	-0.21870
C	-4.99760	-3.52500	-0.51600
C	7.61960	-1.89760	-1.77820
C	6.76880	-1.53910	-2.82110
C	5.51130	-1.01420	-2.53300
C	5.09780	-0.84500	-1.21200
C	5.96390	-1.20880	-0.16850
C	7.22240	-1.73450	-0.45310
C	-0.60050	8.41500	-1.75780
C	-0.46450	7.50500	-2.80340
C	-0.25290	6.15800	-2.51950
C	-0.17650	5.71370	-1.19990
C	-0.31490	6.63970	-0.15360
C	-0.52660	7.98790	-0.43400
B	1.90240	-5.19970	4.93850
O	2.38590	-6.50650	4.89270
B	5.20380	5.14570	4.96140
O	4.93720	4.68450	6.25060
B	-5.37490	2.88430	4.93340
O	-6.72530	3.22590	4.87950
O	1.63260	-4.74050	6.22770
O	-4.87150	2.76690	6.22900
O	6.08950	6.22130	4.91670
C	1.96180	-5.81210	6.99480
C	2.40720	-6.85700	6.20370
C	2.80850	-8.08140	6.74160
C	1.88420	-5.91250	8.38620
C	6.37640	6.41900	6.22840
C	5.69450	5.51000	7.01890
C	5.81500	5.49660	8.41080
C	7.23060	7.38300	6.76740
C	-5.96010	3.04600	6.99210
C	-7.05650	3.31870	6.19230
C	-8.30700	3.63860	6.72440
C	-6.03190	3.07120	8.38720
C	2.73000	-8.18170	8.13200
C	2.28530	-7.13770	8.92490
O	2.30450	-7.49330	10.23580
B	2.78040	-8.80350	10.18460
O	3.05510	-9.25600	8.89490
C	6.67030	6.46060	8.95050
C	7.35170	7.36850	8.15830
O	8.11420	8.19120	8.92220
B	7.85770	7.72800	10.21170
O	6.96350	6.65850	10.26200
C	-8.37820	3.66420	8.11850
C	-7.28340	3.39040	8.92020
O	-7.61990	3.48110	10.23320
B	-8.97050	3.82510	10.17410
O	-9.46760	3.94530	8.87730
H	1.92010	-2.13670	-0.60750
H	0.24940	-2.44280	-0.52780
H	2.57750	3.60860	-0.59450
H	3.67910	2.31600	-0.50540
H	-2.15300	2.90450	-0.52850
H	-2.73450	1.30810	-0.60160
H	-2.52160	-0.48740	0.82810
H	-1.43940	-1.82480	0.83220
H	3.98030	0.54600	0.85740
H	3.35940	-1.05850	0.85700
H	-0.78060	4.04590	0.86740
H	0.91820	4.31990	0.86170
H	2.33900	-5.85600	2.27370
H	1.98050	-4.51040	0.25910
H	0.59240	-1.24420	2.69420
H	0.95140	-2.59070	4.70310
H	5.56590	5.84550	2.29700
H	4.59350	4.85420	0.27980
H	2.45450	2.01840	2.71020
H	3.42770	3.01060	4.72220
H	-2.68020	2.20740	4.71310
H	-1.33290	1.83060	2.70860
H	-4.77010	2.64180	0.24800
H	-6.11610	3.02030	2.25890
H	-6.15200	-4.43950	-2.07590
H	-4.83780	-3.42340	-3.90860
H	-2.93690	-1.93050	-3.37460
H	-3.69380	-2.49560	0.81730
H	-5.57390	-3.97090	0.28150
H	8.59580	-2.30510	-1.99920
H	7.08070	-1.66670	-3.84740
H	4.84570	-0.73410	-3.33600
H	5.67780	-1.09070	0.86470
H	7.88720	-2.01410	0.35130
H	-0.76500	9.46070	-1.97560
H	-0.52280	7.84020	-3.82870
H	-0.14660	5.44620	-3.32460
H	-0.26130	6.33150	0.87860
H	-0.63310	8.69880	0.37250
H	3.15470	-8.89600	6.12400
H	1.53640	-5.09850	9.00360
H	5.28430	4.78770	9.02770
H	7.76270	8.09060	6.15030
H	-9.16130	3.85150	6.10010
H	-5.17770	2.85820	9.01150
C	3.35350	-10.64050	18.99990
C	2.18980	-11.15780	18.39620
C	2.22830	-11.60820	17.06130
C	3.44400	-11.59250	16.34860
C	4.60750	-11.07300	16.95060
C	4.56860	-10.62540	18.28630
C	3.29160	-10.06640	20.42370
C	0.90060	-11.32510	19.22490
C	0.93990	-12.08820	16.37550
C	3.52690	-12.23810	14.95110
C	5.90980	-10.96960	16.14220
C	5.87280	-10.21650	18.99930
C	-2.04420	-8.14780	19.10660
C	-2.33190	-9.37780	19.70710
C	-1.37510	-10.38730	19.73810
C	-0.11640	-10.18200	19.17010
C	0.17530	-8.95360	18.56600
C	-0.78320	-7.94430	18.53600
C	2.97780	-9.70940	11.47510
C	3.44060	-11.02300	11.34750
C	3.61100	-11.82210	12.47340
C	3.32100	-11.32020	13.74350
C	2.86170	-10.00480	13.87670
C	2.69070	-9.20660	12.74870
C	7.02850	-6.03990	18.96740
C	7.77250	-6.99100	19.67290
C	7.38870	-8.32820	19.67110

C	6.25620	-8.73480	18.96330
C	5.50940	-7.78690	18.25400
C	5.89440	-6.44880	18.25750
O	3.56310	-11.10620	21.35980
O	0.79050	-13.48950	16.58750
O	6.65850	-12.17130	16.30500
C	3.62480	-10.43600	25.47340
C	3.87480	-11.69990	24.94450
C	3.84550	-11.88570	23.56440
C	3.56870	-10.81910	22.70980
C	3.31820	-9.54930	23.25390
C	3.34670	-9.35980	24.63390
C	-2.49120	-15.61900	15.14840
C	-1.48180	-16.25050	15.87100
C	-0.40010	-15.50620	16.33560
C	-0.31950	-14.13700	16.08380
C	-1.34230	-13.51090	15.35370
C	-2.42590	-14.25230	14.88770
C	10.32490	-12.78080	14.41800
C	9.76580	-13.75580	15.24050
C	8.53880	-13.51640	15.85450
C	7.86680	-12.31120	15.65280
C	8.43970	-11.33520	14.82190
C	9.66700	-11.57140	14.20610
C	-11.83900	3.34310	17.00250
C	-11.41190	3.22560	18.34020
C	-10.82920	4.32980	18.99380
C	-10.72810	5.56800	18.32840
C	-11.16000	5.68790	16.99210
C	-11.74130	4.58250	16.33930
C	-12.38400	2.11430	16.25880
C	-11.67680	1.92170	19.11890
C	-10.27850	4.17690	20.42000
C	-10.23750	6.81340	19.09340
C	-10.97020	7.01310	16.23810
C	-12.36210	4.75610	14.93890
C	-8.65830	-1.18680	19.04000
C	-9.90160	-1.40120	19.64360
C	-10.85980	-0.39270	19.65980
C	-10.58970	0.84490	19.07290
C	-9.34820	1.06290	18.46430
C	-8.38990	0.05290	18.44980
C	-5.99660	7.70690	19.07430
C	-6.88960	8.45440	19.84880
C	-8.24810	8.15430	19.84420
C	-8.73420	7.10240	19.06550
C	-7.84370	6.34920	18.29150
C	-6.48460	6.65150	18.29650
C	-9.86550	4.06690	11.46440
C	-11.20980	4.42850	11.32930
C	-11.99760	4.64470	12.45530
C	-11.45380	4.50210	13.73310
C	-10.10950	4.13770	13.87350
C	-9.32210	3.92280	12.74550
O	-13.79640	2.04690	16.43750
O	-11.30780	4.49000	21.35460
O	-12.12500	7.82700	16.42470
C	-16.11220	-0.97220	14.73530
C	-16.69040	0.03140	15.50880
C	-15.88450	1.02370	16.06180
C	-14.50650	1.02080	15.84770
C	-13.93420	0.00410	15.06660
C	-14.73740	-0.99010	14.51200
C	-10.68610	4.36500	25.47440
C	-11.92250	4.72220	24.94170
C	-12.09280	4.75270	23.55970
C	-11.03800	4.42930	22.70680
C	-9.79600	4.07050	23.25450
C	-9.62220	4.03890	24.63650
C	-12.47850	11.61250	14.71590
C	-13.49670	11.07740	15.50130
C	-13.34280	9.80910	16.05590
C	-12.18040	9.07200	15.83160
C	-11.16050	9.62110	15.03820
C	-11.31110	10.88960	14.48200
C	9.16050	9.09330	19.02180
C	10.19530	8.35820	18.40970
C	10.56630	8.63630	17.07890
C	9.93800	9.68570	16.37840
C	8.90350	10.42180	16.99010
C	8.53650	10.14550	18.32230
C	8.69400	8.73070	20.43990
C	10.98860	7.31920	19.22710
C	11.63460	7.77800	16.38400
C	10.44870	10.09510	14.98260
C	8.15860	11.50550	16.19560
C	7.52960	11.05890	19.04940
C	9.69960	3.18290	19.11590
C	10.85130	3.56320	19.81240
C	11.25030	4.89570	19.83960
C	10.50350	5.86810	19.17200
C	9.34930	5.49250	18.47480
C	8.95190	4.15840	18.44780
C	8.54050	8.35370	11.50300
C	9.44530	9.41270	11.37690
C	10.04980	9.96060	12.50390
C	9.75900	9.45800	13.77340
C	8.85080	8.40100	13.90490
C	8.24750	7.85310	12.77610
C	3.34220	9.94300	19.04020
C	3.80620	11.00450	19.82380
C	5.15360	11.35040	19.81460
C	6.05720	10.64090	19.02150
C	5.59830	9.57630	18.23730
C	4.24930	9.23150	18.24750
O	9.45450	9.47590	21.38730
O	12.91650	8.36080	16.60310
O	8.82210	12.75430	16.37210
C	8.83170	9.14330	25.49540
C	9.80150	9.99960	24.97960
C	9.98100	10.08420	23.60100
C	9.19950	9.32020	22.73480
C	8.22500	8.46020	23.26560
C	8.04270	8.37320	24.64420
C	16.41940	6.65980	15.11620
C	16.44840	7.83540	15.86260
C	15.25730	8.37440	16.34280
C	14.03860	7.74820	16.08320
C	14.02100	6.56380	15.32950
C	15.21070	6.02180	14.84770
C	7.51610	16.25020	14.51520
C	8.63680	16.24770	15.34200
C	9.04320	15.06020	15.94580
C	8.33880	13.87620	15.72970
C	7.21020	13.89030	14.89480
C	6.80100	15.07640	14.28910
B	-7.56600	-2.34080	19.02910
O	-6.30100	-2.19040	18.46120
O	-7.78480	-3.59360	19.60020
C	-5.72720	-3.39800	18.70080
C	-6.60640	-4.22820	19.37460
C	-6.26670	-5.53070	19.74550
C	-4.43940	-3.80800	18.34680
C	-4.97950	-5.94000	19.39190
C	-4.09880	-5.11100	18.71850
O	-2.91910	-5.74720	18.49860
B	-3.13980	-6.99750	19.07630
O	-4.40820	-7.14690	19.63530
B	9.25050	1.65900	19.08640
O	8.12400	1.20690	18.39900
O	9.95380	0.65140	19.74480
C	8.14430	-0.12800	18.64970
C	9.22720	-0.45560	19.44740
C	9.48280	-1.76570	19.85680
C	7.23450	-1.08780	18.19890
C	8.57280	-2.72430	19.40700
C	7.49020	-2.39910	18.60790
O	6.76620	-3.50960	18.31150
B	7.47010	-4.51340	18.97690
O	8.59450	-4.05690	19.66290
B	1.80130	9.55510	19.05340
O	1.26860	8.51190	18.29590

O	0.85760	10.23700	19.82010
C	-0.05130	8.56470	18.61280
C	-0.29360	9.58660	19.51460
C	-1.57180	9.86140	20.00480
C	-1.07060	7.73840	18.13280
C	-2.59020	9.03610	19.52420
C	-2.34980	8.01320	18.62300
O	-3.50440	7.36630	18.31750
B	-4.44670	8.05740	19.07940
O	-3.90910	9.09520	19.83910
H	0.40160	-12.24840	18.92780
H	1.14530	-11.51540	20.27000
H	4.48950	-12.74010	14.84610
H	2.82050	-13.06500	14.87550
H	6.70890	-10.79680	18.60830
H	5.83090	-10.53590	20.04120
H	-3.30140	-9.55450	20.15310
H	-1.61640	-11.33150	20.20630
H	1.14150	-8.77040	18.11790
H	-0.54270	-6.99990	18.06680
H	3.66870	-11.42960	10.37140
H	3.96900	-12.83530	12.35430
H	2.63250	-9.59250	14.84900
H	2.33260	-8.19290	12.86700
H	8.65240	-6.69450	20.22780
H	7.97550	-9.04830	20.22430
H	4.62890	-8.07600	17.69790
H	5.30670	-5.72750	17.70570
H	3.99720	-9.24290	20.53600
H	2.31830	-9.61210	20.61150
H	0.95930	-11.85250	15.31110
H	0.07840	-11.53850	16.75540
H	6.48820	-10.10010	16.45640
H	5.69280	-10.78120	15.09050
H	3.64690	-10.28930	26.54400
H	4.08980	-12.53190	25.59900
H	4.03810	-12.86270	23.14650
H	3.10060	-8.70200	22.62300
H	3.15360	-8.38200	25.05060
H	-3.33170	-16.19440	14.78690
H	-1.53590	-17.31080	16.07060
H	0.38640	-15.98840	16.89690
H	-1.31220	-12.45420	15.13960
H	-3.21200	-13.76840	14.32640
H	11.27700	-12.96410	13.94050
H	10.27940	-14.69220	15.40250
H	8.09860	-14.26730	16.49360
H	7.94830	-10.39170	14.64390
H	10.10620	-10.81910	13.56720
H	-11.88830	2.14920	20.16400
H	-12.61470	1.48100	18.77890
H	-10.76310	7.69970	18.73720
H	-10.55880	6.75140	20.13370
H	-12.78220	5.75820	14.84650
H	-13.24370	4.12130	14.84520
H	-10.12830	-2.35300	20.10480
H	-11.81450	-0.57700	20.13240
H	-9.11500	2.01140	18.00190
H	-7.43420	0.23690	17.97790
H	-6.53150	9.27410	20.45720
H	-8.92300	8.74450	20.44840
H	-8.19410	5.52920	17.68110
H	-5.80920	6.06190	17.69150
H	-11.64840	4.54460	10.34730
H	-13.03420	4.92530	12.33050
H	-9.66480	4.01990	14.85140
H	-8.28460	3.64320	12.86960
H	-12.12300	2.16190	15.20100
H	-11.89930	1.20450	16.61420
H	-9.40790	4.81740	20.56410
H	-9.89750	3.16770	20.57850
H	-10.78660	6.82880	15.17920
H	-10.06960	7.52360	16.58060
H	-16.73550	-1.74320	14.30510
H	-17.75700	0.04180	15.67960
H	-16.32510	1.80540	16.66250

H	-12.87230	-0.02960	14.88070
H	-14.29510	-1.77150	13.91120
H	-10.55150	4.34060	26.54640
H	-12.74500	4.97450	25.59490
H	-13.04820	5.02870	23.13890
H	-8.95870	3.81410	22.62490
H	-8.66580	3.76250	25.05600
H	-12.59550	12.59650	14.28460
H	-14.40060	11.64130	15.68020
H	-14.12760	9.38710	16.66600
H	-10.24830	9.07950	14.84380
H	-10.52510	11.31040	13.87190
H	12.03620	7.34390	18.92520
H	11.03760	7.62530	20.27240
H	10.39210	11.17980	14.88370
H	11.51960	9.90730	14.90340
H	7.60280	12.07730	18.66730
H	7.83690	11.17450	20.08950
H	11.44280	2.82370	20.33530
H	12.14460	5.16990	20.38180
H	8.75360	6.22580	17.95010
H	8.05810	3.88330	17.90420
H	9.68430	9.81390	10.40120
H	10.74730	10.77820	12.38590
H	8.60740	7.99540	14.87650
H	7.54990	7.03490	12.89330
H	3.12060	11.56640	20.44380
H	5.49310	12.17390	20.42730
H	6.27820	9.01090	17.61600
H	3.90900	8.40730	17.63520
H	7.62700	8.92410	20.55320
H	8.79190	7.65850	20.61280
H	11.42150	7.68650	15.31850
H	11.60050	6.75230	16.75210
H	7.11680	11.56610	16.51190
H	8.10280	11.23500	15.14080
H	8.69050	9.07590	26.56480
H	10.41160	10.59500	25.64320
H	10.73080	10.74570	23.19320
H	7.60300	7.85460	22.62550
H	7.29220	7.71080	25.05060
H	17.34230	6.23960	14.74250
H	17.38780	8.32740	16.06840
H	15.27130	9.28560	16.92230
H	13.09690	6.05320	15.10920
H	15.19510	5.11030	14.26800
H	7.19870	17.17040	14.04560
H	9.18840	17.16030	15.51520
H	9.91120	15.05010	16.58820
H	6.64130	12.99360	14.70590
H	5.93230	15.08510	13.64680
H	-6.95250	-6.17730	20.27130
H	-3.75300	-3.16110	17.82220
H	10.32690	-2.02020	20.47940
H	6.39030	-0.83310	17.57660
H	-1.76010	10.65900	20.70710
H	-0.88260	6.94180	17.42940
C	-0.50610	0.03700	0.20530
C	0.81050	-0.46560	0.18990
C	1.90480	0.42230	0.21610
C	1.68200	1.81380	0.20040
C	0.36590	2.31720	0.21860
C	-0.72760	1.42870	0.19110
C	-1.69880	-0.92960	0.26610
C	1.05650	-1.98020	0.04020
C	3.33750	-0.12770	0.28990
C	2.87110	2.78430	0.05680
C	0.12370	3.83240	0.29640
C	-2.16120	1.97590	0.04260
O	-2.10140	-1.25960	-1.06060
O	3.83460	-0.31890	-1.03190
O	0.03510	4.36120	-1.02400
C	1.67040	-4.32140	3.63480
C	1.95670	-4.84880	2.37150
C	1.75260	-4.08520	1.22670
C	1.25840	-2.78350	1.32760

C	0.97350	-2.25010	2.58990
C	1.17790	-3.01570	3.73460
C	4.56810	4.49950	3.65620
C	4.88690	5.00890	2.39330
C	4.33540	4.44580	1.24700
C	3.45780	3.36460	1.34600
C	3.13330	2.85310	2.60770
C	3.68650	3.41760	3.75410
C	-4.49600	2.64110	3.63220
C	-3.14240	2.30440	3.74000
C	-2.37610	2.09010	2.59740
C	-2.95650	2.21040	1.32940
C	-4.30710	2.54650	1.22040
C	-5.07110	2.76100	2.36300
C	-5.32150	-3.78760	-1.84510
C	-4.58350	-3.21690	-2.87920
C	-3.51390	-2.37710	-2.57850
C	-3.17640	-2.10360	-1.25330
C	-3.92680	-2.68460	-0.21870
C	-4.99760	-3.52500	-0.51600
C	7.61960	-1.89760	-1.77820
C	6.76880	-1.53910	-2.82110
C	5.51130	-1.01420	-2.53300
C	5.09780	-0.84500	-1.21200
C	5.96390	-1.20880	-0.16850
C	7.22240	-1.73450	-0.45310
C	-0.60050	8.41500	-1.75780
C	-0.46450	7.50500	-2.80340
C	-0.25290	6.15800	-2.51950
C	-0.17650	5.71370	-1.19990
C	-0.31490	6.63970	-0.15360
C	-0.52660	7.98790	-0.43400
B	1.90240	-5.19970	4.93850
O	2.38590	-6.50650	4.89270
B	5.20380	5.14570	4.96140
O	4.93720	4.68450	6.25060
B	-5.37490	2.88430	4.93340
O	-6.72530	3.22590	4.87950
O	1.63260	-4.74050	6.22770
O	-4.87150	2.76690	6.22900
O	6.08950	6.22130	4.91670
C	1.96180	-5.81210	6.99480
C	2.40720	-6.85700	6.20370
C	2.80850	-8.08140	6.74160
C	1.88420	-5.91250	8.38620
C	6.37640	6.41900	6.22840
C	5.69450	5.51000	7.01890
C	5.81500	5.49660	8.41080
C	7.23060	7.38300	6.76740
C	-5.96010	3.04600	6.99210
C	-7.05650	3.31870	6.19230
C	-8.30700	3.63860	6.72440
C	-6.03190	3.07120	8.38720
C	2.73000	-8.18170	8.13200
C	2.28530	-7.13770	8.92490
O	2.30450	-7.49330	10.23580
B	2.78040	-8.80350	10.18460
O	3.05510	-9.25600	8.89490
C	6.67030	6.46060	8.95050
C	7.35170	7.36850	8.15830
O	8.11420	8.19120	8.92220
B	7.85770	7.72800	10.21170
O	6.96350	6.65850	10.26200
C	-8.37820	3.66420	8.11850
C	-7.28340	3.39040	8.92020
O	-7.61990	3.48110	10.23320
B	-8.97050	3.82510	10.17410
O	-9.46760	3.94530	8.87730
H	1.92010	-2.13670	-0.60750
H	0.24940	-2.44280	-0.52780
H	2.57750	3.60860	-0.59450
H	3.67910	2.31600	-0.50540
H	-2.15300	2.90450	-0.52850
H	-2.73450	1.30810	-0.60160
H	-2.52160	-0.48740	0.82810
H	-1.43940	-1.82480	0.83220
H	3.98030	0.54600	0.85740
H	3.35940	-1.05850	0.85700
H	-0.78060	4.04590	0.86740
H	0.91820	4.31990	0.86170
H	2.33900	-5.85600	2.27370
H	1.98050	-4.51040	0.25910
H	0.59240	-1.24420	2.69420
H	0.95140	-2.59070	4.70310
H	5.56590	5.84550	2.29700
H	4.59350	4.85420	0.27980
H	2.45450	2.01840	2.71020
H	3.42770	3.01060	4.72220
H	-2.68020	2.20740	4.71310
H	-1.33290	1.83060	2.70860
H	-4.77010	2.64180	0.24800
H	-6.11610	3.02030	2.25890
H	-6.15200	-4.43950	-2.07590
H	-4.83780	-3.42340	-3.90860
H	-2.93690	-1.93050	-3.37460
H	-3.69380	-2.49560	0.81730
H	-5.57390	-3.97090	0.28150
H	8.59580	-2.30510	-1.99920
H	7.08070	-1.66670	-3.84740
H	4.84570	-0.73410	-3.33600
H	5.67780	-1.09070	0.86470
H	7.88720	-2.01410	0.35130
H	-0.76500	9.46070	-1.97560
H	-0.52280	7.84020	-3.82870
H	-0.14660	5.44620	-3.32460
H	-0.26130	6.33150	0.87860
H	-0.63310	8.69880	0.37250
H	3.15470	-8.89600	6.12400
H	1.53640	-5.09850	9.00360
H	5.28430	4.78770	9.02770
H	7.76270	8.09060	6.15030
H	-9.16130	3.85150	6.10010
H	-5.17770	2.85820	9.01150
C	3.35350	-10.64050	18.99990
C	2.18980	-11.15780	18.39620
C	2.22830	-11.60820	17.06130
C	3.44400	-11.59250	16.34860
C	4.60750	-11.07300	16.95060
C	4.56860	-10.62540	18.28630
C	3.29160	-10.06640	20.42370
C	0.90060	-11.32510	19.22490
C	0.93990	-12.08820	16.37550
C	3.52690	-12.23810	14.95110
C	5.90980	-10.96960	16.14220
C	5.87280	-10.21650	18.99930
C	-2.04420	-8.14780	19.10660
C	-2.33190	-9.37780	19.70710
C	-1.37510	-10.38730	19.73810
C	-0.11640	-10.18200	19.17010
C	0.17530	-8.95360	18.56600
C	-0.78320	-7.94430	18.53600
C	2.97780	-9.70940	11.47510
C	3.44060	-11.02300	11.34750
C	3.61100	-11.82210	12.47340
C	3.32100	-11.32020	13.74350
C	2.86170	-10.00480	13.87670
C	2.69070	-9.20660	12.74870
C	7.02850	-6.03990	18.96740
C	7.77250	-6.99100	19.67290
C	7.38870	-8.32820	19.67110
C	6.25620	-8.73480	18.96330
C	5.50940	-7.78690	18.25400
C	5.89440	-6.44880	18.25750
O	3.56310	-11.10620	21.35980
O	0.79050	-13.48950	16.58750
O	6.65850	-12.17130	16.30500
C	3.62480	-10.43600	25.47340
C	3.87480	-11.69990	24.94450
C	3.84550	-11.88570	23.56440
C	3.56870	-10.81910	22.70980
C	3.31820	-9.54930	23.25390
C	3.34670	-9.35980	24.63390

C	-2.49120	-15.61900	15.14840
C	-1.48180	-16.25050	15.87100
C	-0.40010	-15.50620	16.33560
C	-0.31950	-14.13700	16.08380
C	-1.34230	-13.51090	15.35370
C	-2.42590	-14.25230	14.88770
C	10.32490	-12.78080	14.41800
C	9.76580	-13.75580	15.24050
C	8.53880	-13.51640	15.85450
C	7.86680	-12.31120	15.65280
C	8.43970	-11.33520	14.82190
C	9.66700	-11.57140	14.20610
C	-11.83900	3.34310	17.00250
C	-11.41190	3.22560	18.34020
C	-10.82920	4.32980	18.99380
C	-10.72810	5.56800	18.32840
C	-11.16000	5.68790	16.99210
C	-11.74130	4.58250	16.33930
C	-12.38400	2.11430	16.25880
C	-11.67680	1.92170	19.11890
C	-10.27850	4.17690	20.42000
C	-10.23750	6.81340	19.09340
C	-10.97020	7.01310	16.23810
C	-12.36210	4.75610	14.93890
C	-8.65830	-1.18680	19.04000
C	-9.90160	-1.40120	19.64360
C	-10.85980	-0.39270	19.65980
C	-10.58970	0.84490	19.07290
C	-9.34820	1.06290	18.46430
C	-8.38990	0.05290	18.44980
C	-5.99660	7.70690	19.07430
C	-6.88960	8.45440	19.84880
C	-8.24810	8.15430	19.84420
C	-8.73420	7.10240	19.06550
C	-7.84370	6.34920	18.29150
C	-6.48460	6.65150	18.29650
C	-9.86550	4.06690	11.46440
C	-11.20980	4.42850	11.32930
C	-11.99760	4.64470	12.45530
C	-11.45380	4.50210	13.73310
C	-10.10950	4.13770	13.87350
C	-9.32210	3.92280	12.74550
O	-13.79640	2.04690	16.43750
O	-11.30780	4.49000	21.35460
O	-12.12500	7.82700	16.42470
C	-16.11220	-0.97220	14.73530
C	-16.69040	0.03140	15.50880
C	-15.88450	1.02370	16.06180
C	-14.50650	1.02080	15.84770
C	-13.93420	0.00410	15.06660
C	-14.73740	-0.99010	14.51200
C	-10.68610	4.36500	25.47440
C	-11.92250	4.72220	24.94170
C	-12.09280	4.75270	23.55970
C	-11.03800	4.42930	22.70680
C	-9.79600	4.07050	23.25450
C	-9.62220	4.03890	24.63650
C	-12.47850	11.61250	14.71590
C	-13.49670	11.07740	15.50130
C	-13.34280	9.80910	16.05590
C	-12.18040	9.07200	15.83160
C	-11.16050	9.62110	15.03820
C	-11.31110	10.88960	14.48200
C	9.16050	9.09330	19.02180
C	10.19530	8.35820	18.40970
C	10.56630	8.63630	17.07890
C	9.93800	9.68570	16.37840
C	8.90350	10.42180	16.99010
C	8.53650	10.14550	18.32230
C	8.69400	8.73070	20.43990
C	10.98860	7.31920	19.22710
C	11.63460	7.77800	16.38400
C	10.44870	10.09510	14.98260
C	8.15860	11.50550	16.19560
C	7.52960	11.05890	19.04940
C	9.69960	3.18290	19.11590

C	10.85130	3.56320	19.81240
C	11.25030	4.89570	19.83960
C	10.50350	5.86810	19.17200
C	9.34930	5.49250	18.47480
C	8.95190	4.15840	18.44780
C	8.54050	8.35370	11.50300
C	9.44530	9.41270	11.37690
C	10.04980	9.96060	12.50390
C	9.75900	9.45800	13.77340
C	8.85080	8.40100	13.90490
C	8.24750	7.85310	12.77610
C	3.34220	9.94300	19.04020
C	3.80620	11.00450	19.82380
C	5.15360	11.35040	19.81460
C	6.05720	10.64090	19.02150
C	5.59830	9.57630	18.23730
C	4.24930	9.23150	18.24750
O	9.45450	9.47590	21.38730
O	12.91650	8.36080	16.60310
O	8.82210	12.75430	16.37210
C	8.83170	9.14330	25.49540
C	9.80150	9.99960	24.97960
C	9.98100	10.08420	23.60100
C	9.19950	9.32020	22.73480
C	8.22500	8.46020	23.26560
C	8.04270	8.37320	24.64420
C	16.41940	6.65980	15.11620
C	16.44840	7.83540	15.86260
C	15.25730	8.37440	16.34280
C	14.03860	7.74820	16.08320
C	14.02100	6.56380	15.32950
C	15.21070	6.02180	14.84770
C	7.51610	16.25020	14.51520
C	8.63680	16.24770	15.34200
C	9.04320	15.06020	15.94580
C	8.33880	13.87620	15.72970
C	7.21020	13.89030	14.89480
C	6.80100	15.07640	14.28910
B	-7.56600	-2.34080	19.02910
O	-6.30100	-2.19040	18.46120
O	-7.78480	-3.59360	19.60020
C	-5.72720	-3.39800	18.70080
C	-6.60640	-4.22820	19.37460
C	-6.26670	-5.53070	19.74550
C	-4.43940	-3.80800	18.34680
C	-4.97950	-5.94000	19.39190
C	-4.09880	-5.11100	18.71850
O	-2.91910	-5.74720	18.49860
B	-3.13980	-6.99750	19.07630
O	-4.40820	-7.14690	19.63530
B	9.25050	1.65900	19.08640
O	8.12400	1.20690	18.39900
O	9.95380	0.65140	19.74480
C	8.14430	-0.12800	18.64970
C	9.22720	-0.45560	19.44740
C	9.48280	-1.76570	19.85680
C	7.23450	-1.08780	18.19890
C	8.57280	-2.72430	19.40700
C	7.49020	-2.39910	18.60790
O	6.76620	-3.50960	18.31150
B	7.47010	-4.51340	18.97690
O	8.59450	-4.05690	19.66290
B	1.80130	9.55510	19.05340
O	1.26860	8.51190	18.29590
O	0.85760	10.23700	19.82010
C	-0.05130	8.56470	18.61280
C	-0.29360	9.58660	19.51460
C	-1.57180	9.86140	20.00480
C	-1.07060	7.73840	18.13280
C	-2.59020	9.03610	19.52420
C	-2.34980	8.01320	18.62300
O	-3.50440	7.36630	18.31750
B	-4.44670	8.05740	19.07940
O	-3.90910	9.09520	19.83910
H	0.40160	-12.24840	18.92780
H	1.14530	-11.51540	20.27000

H	4.48950	-12.74010	14.84610
H	2.82050	-13.06500	14.87550
H	6.70890	-10.79680	18.60830
H	5.83090	-10.53590	20.04120
H	-3.30140	-9.55450	20.15310
H	-1.61640	-11.33150	20.20630
H	1.14150	-8.77040	18.11790
H	-0.54270	-6.99990	18.06680
H	3.66870	-11.42960	10.37140
H	3.96900	-12.83530	12.35430
H	2.63250	-9.59250	14.84900
H	2.33260	-8.19290	12.86700
H	8.65240	-6.69450	20.22780
H	7.97550	-9.04830	20.22430
H	4.62890	-8.07600	17.69790
H	5.30670	-5.72750	17.70570
H	3.99720	-9.24290	20.53600
H	2.31830	-9.61210	20.61150
H	0.95930	-11.85250	15.31110
H	0.07840	-11.53850	16.75540
H	6.48820	-10.10010	16.45640
H	5.69280	-10.78120	15.09050
H	3.64690	-10.28930	26.54400
H	4.08980	-12.53190	25.59900
H	4.03810	-12.86270	23.14650
H	3.10060	-8.70200	22.62300
H	3.15360	-8.38200	25.05060
H	-3.33170	-16.19440	14.78690
H	-1.53590	-17.31080	16.07060
H	0.38640	-15.98840	16.89690
H	-1.31220	-12.45420	15.13960
H	-3.21200	-13.76840	14.32640
H	11.27700	-12.96410	13.94050
H	10.27940	-14.69220	15.40250
H	8.09860	-14.26730	16.49360
H	7.94830	-10.39170	14.64390
H	10.10620	-10.81910	13.56720
H	-11.88830	2.14920	20.16400
H	-12.61470	1.48100	18.77890
H	-10.76310	7.69970	18.73720
H	-10.55880	6.75140	20.13370
H	-12.78220	5.75820	14.84650
H	-13.24370	4.12130	14.84520
H	-10.12830	-2.35300	20.10480
H	-11.81450	-0.57700	20.13240
H	-9.11500	2.01140	18.00190
H	-7.43420	0.23690	17.97790
H	-6.53150	9.27410	20.45720
H	-8.92300	8.74450	20.44840
H	-8.19410	5.52920	17.68110
H	-5.80920	6.06190	17.69150
H	-11.64840	4.54460	10.34730
H	-13.03420	4.92530	12.33050
H	-9.66480	4.01990	14.85140
H	-8.28460	3.64320	12.86960
H	-12.12300	2.16190	15.20100
H	-11.89930	1.20450	16.61420
H	-9.40790	4.81740	20.56410
H	-9.89750	3.16770	20.57850
H	-10.78660	6.82880	15.17920
H	-10.06960	7.52360	16.58060
H	-16.73550	-1.74320	14.30510
H	-17.75700	0.04180	15.67960
H	-16.32510	1.80540	16.66250
H	-12.87230	-0.02960	14.88070
H	-14.29510	-1.77150	13.91120
H	-10.55150	4.34060	26.54640
H	-12.74500	4.97450	25.59490
H	-13.04820	5.02870	23.13890
H	-8.95870	3.81410	22.62490
H	-8.66580	3.76250	25.05600
H	-12.59550	12.59650	14.28460
H	-14.40060	11.64130	15.68020
H	-14.12760	9.38710	16.66600
H	-10.24830	9.07950	14.84380
H	-10.52510	11.31040	13.87190
H	12.03620	7.34390	18.92520
H	11.03760	7.62530	20.27240
H	10.39210	11.17980	14.88370
H	11.51960	9.90730	14.90340
H	7.60280	12.07730	18.66730
H	7.83690	11.17450	20.08950
H	11.44280	2.82370	20.33530
H	12.14460	5.16990	20.38180
H	8.75360	6.22580	17.95010
H	8.05810	3.88330	17.90420
H	9.68430	9.81390	10.40120
H	10.74730	10.77820	12.38590
H	8.60740	7.99540	14.87650
H	7.54990	7.03490	12.89330
H	3.12060	11.56640	20.44380
H	5.49310	12.17390	20.42730
H	6.27820	9.01090	17.61600
H	3.90900	8.40730	17.63520
H	7.62700	8.92410	20.55320
H	8.79190	7.65850	20.61280
H	11.42150	7.68650	15.31850
H	11.60050	6.75230	16.75210
H	7.11680	11.56610	16.51190
H	8.10280	11.23500	15.14080
H	8.69050	9.07590	26.56480
H	10.41160	10.59500	25.64320
H	10.73080	10.74570	23.19320
H	7.60300	7.85460	22.62550
H	7.29220	7.71080	25.05060
H	17.34230	6.23960	14.74250
H	17.38780	8.32740	16.06840
H	15.27130	9.28560	16.92230
H	13.09690	6.05320	15.10920
H	15.19510	5.11030	14.26800
H	7.19870	17.17040	14.04560
H	9.18840	17.16030	15.51520
H	9.91120	15.05010	16.58820
H	6.64130	12.99360	14.70590
H	5.93230	15.08510	13.64680
H	-6.95250	-6.17730	20.27130
H	-3.75300	-3.16110	17.82220
H	10.32690	-2.02020	20.47940
H	6.39030	-0.83310	17.57660
H	-1.76010	10.65900	20.70710
H	-0.88260	6.94180	17.42940

Tb

Potential Energy (OPLS4): 1440.033 kJ/mol

C	-3.57780	3.05090	3.17190
C	-2.69850	3.86580	2.40830
C	-2.24920	5.11250	2.92320
C	-2.70300	5.55720	4.19530
C	-3.58460	4.74480	4.96050
C	-4.03010	3.49630	4.44450
C	-4.02220	1.68530	2.62950
C	-2.22040	3.39090	1.03050
C	-1.26040	5.96350	2.11470
C	-2.25030	6.91850	4.74160
C	-4.04670	5.20980	6.34830
C	-5.00780	2.63740	5.25900
C	-5.31710	1.76750	1.80520
C	-1.96400	6.95250	1.17200
C	-5.34160	6.03850	6.30270
C	1.37500	0.92350	0.93500
C	0.58690	1.12300	-0.21790
C	-0.56880	1.92110	-0.16980
C	-0.95960	2.53240	1.03660
C	-0.17440	2.33350	2.19810
C	0.98740	1.53360	2.14590
C	1.15880	6.88620	7.45710
C	0.11080	7.81550	7.61730
C	-0.98820	7.81300	6.73950
C	-1.06030	6.87090	5.69350
C	-0.02090	5.92410	5.54340
C	1.08570	5.93650	6.41760
C	-3.27270	-0.00630	8.25850
C	-2.54830	0.28760	7.08500
C	-3.08850	1.15790	6.11540
C	-4.36530	1.73470	6.30630
C	-5.08040	1.44960	7.48700
C	-4.53450	0.59020	8.45710
B	2.66050	0.02640	0.82300
O	2.93730	-0.55000	-0.40630
B	2.39150	6.94160	8.42750
O	3.56150	6.23740	8.16150
B	-2.71220	-0.99640	9.34000
O	-1.58470	-1.77730	9.10540
O	3.54420	-0.18560	1.88060
O	-3.44190	-1.20870	10.50200
O	2.38430	7.86060	9.46780
C	4.45800	-0.96170	1.23340
C	4.09500	-1.16830	-0.09250
C	4.87560	-1.93200	-0.97570
C	5.64850	-1.51580	1.76030
C	3.67040	7.73520	9.86680
C	4.35960	6.79740	9.10760
C	5.72350	6.51970	9.34170
C	4.28890	8.45430	10.90590
C	-2.72020	-2.22890	11.02080
C	-1.64180	-2.56510	10.21160
C	-0.76510	-3.61820	10.55370
C	-2.99700	-2.91500	12.21770
C	6.05730	-2.47860	-0.44860
C	6.44090	-2.28150	0.87280
O	7.62750	-2.89530	1.13880
B	7.87590	-3.43620	-0.12160
O	6.94950	-3.22600	-1.13250
C	6.34380	7.23240	10.38910
C	5.64730	8.17200	11.13980
O	6.43270	8.75470	12.07420
B	7.61630	8.07990	11.80770
O	7.63540	7.12650	10.79500
C	-1.03770	-4.30460	11.75610
C	-2.12220	-3.96450	12.55560
O	-2.22000	-4.76600	13.64120
B	-1.12710	-5.58810	13.40000
O	-0.34210	-5.34910	12.27660
H	-2.05930	4.23090	0.35960
H	-3.01040	2.84040	0.52510
H	-3.08520	7.40190	5.24320
H	-2.02070	7.62080	3.94480
H	-5.73550	3.28140	5.74690
H	-5.63530	2.02090	4.62090
H	-4.14520	0.96850	3.43950
H	-3.23070	1.23070	2.03560
H	-0.58140	6.50130	2.77360
H	-0.57890	5.32890	1.55000
H	-4.17620	4.35890	7.01460
H	-3.26210	5.77880	6.84330
H	0.87490	0.66110	-1.15180
H	-1.15510	2.06370	-1.06660
H	-0.45340	2.79110	3.13720
H	1.58430	1.38600	3.03510
H	0.16070	8.54770	8.41100
H	-1.77250	8.54470	6.86970
H	-0.06210	5.18670	4.75470
H	1.88450	5.21880	6.29130
H	-1.57730	-0.16160	6.92870
H	-2.51890	1.37240	5.22210
H	-6.05730	1.88180	7.65080
H	-5.09510	0.37090	9.35510
H	4.58750	-2.08470	-2.00540
H	5.94080	-1.35540	2.78750
H	6.26540	5.79700	8.75110
H	3.75090	9.18710	11.48850
H	0.07030	-3.88950	9.92610
H	-3.84220	-2.65670	12.83840
H	-5.60250	0.78180	1.43810
H	-5.63370	6.34850	7.30590
H	-1.23370	7.53750	0.61300
H	-5.20490	2.42270	0.94240
H	-6.14630	2.15330	2.39690
H	-2.59660	7.64910	1.72090
H	-2.59970	6.43970	0.45110
H	-6.16980	5.47350	5.87760
H	-5.22620	6.93800	5.70000
C	15.43370	-7.89070	1.41360
C	14.32870	-8.67270	0.97910
C	13.45250	-8.17320	-0.02310
C	13.70020	-6.90370	-0.61330
C	14.80830	-6.12320	-0.18440
C	15.68450	-6.62530	0.81620
C	16.33620	-8.39820	2.54550
C	14.07970	-10.05760	1.59170
C	12.22750	-8.99280	-0.45040
C	12.77230	-6.37910	-1.71770
C	15.04290	-4.72960	-0.78270
C	16.90180	-5.79850	1.25010
C	11.32030	-10.26440	4.96010
C	11.67290	-11.41760	4.22930
C	12.56250	-11.33450	3.14380
C	13.11170	-10.09300	2.76870
C	12.75660	-8.93180	3.49460
C	11.86640	-9.01930	4.58560
C	9.19090	-4.21670	-0.48690
C	9.35830	-4.67410	-1.81050
C	10.51800	-5.37100	-2.18860
C	11.53280	-5.62640	-1.24680
C	11.37080	-5.17290	0.08380
C	10.20480	-4.47130	0.46050
C	16.23930	-2.75930	4.30350
C	17.39300	-2.74610	3.49280
C	17.59230	-3.73410	2.51300
C	16.63680	-4.75130	2.32520
C	15.47690	-4.76940	3.13510
C	15.28110	-3.77740	4.11930
C	12.52000	-9.94240	-1.62420
C	15.92600	-4.75850	-2.04150
C	1.94130	-11.51260	16.69680
C	3.28000	-11.75840	17.10960
C	3.84960	-11.00020	18.16960
C	3.06970	-10.01710	18.83810
C	1.73120	-9.76920	18.42600
C	1.16380	-10.52340	17.36130

C	1.34230	-12.30760	15.52820
C	4.10580	-12.84910	16.41240
C	5.30890	-11.23320	18.58320
C	3.67260	-9.23490	20.01430
C	0.90570	-8.67920	19.12320
C	-0.29020	-10.26770	16.93740
C	6.08550	-11.72730	12.69320
C	5.75340	-13.06960	12.96970
C	5.11190	-13.41730	14.17150
C	4.78740	-12.42460	15.11690
C	5.11270	-11.07570	14.84260
C	5.75930	-10.73110	13.63700
C	6.11820	-5.75560	19.04270
C	6.26620	-6.43900	20.26740
C	5.47270	-7.56050	20.56540
C	4.51680	-8.02160	19.63930
C	4.36500	-7.34300	18.40740
C	5.16250	-6.21590	18.11230
C	-0.88070	-6.83020	14.32710
C	-1.81550	-7.14210	15.33510
C	-1.62100	-8.25730	16.17000
C	-0.47910	-9.06960	16.01510
C	0.46310	-8.75570	15.00970
C	0.25890	-7.64460	14.16700
C	0.60700	-13.57580	15.99010
C	0.13440	-9.20510	20.34480
C	14.84200	6.26710	16.44870
C	15.23880	6.92420	15.25150
C	14.45670	7.99200	14.73090
C	13.28380	8.41280	15.41760
C	12.88440	7.75350	16.61350
C	13.67380	6.69320	17.13880
C	15.66660	5.09090	16.98850
C	16.51610	6.48690	14.51940
C	14.87510	8.68130	13.42460
C	12.45080	9.58520	14.87900
C	11.59670	8.17720	17.33300
C	13.28320	6.02230	18.46400
C	16.01290	3.70220	11.20140
C	17.14160	4.54350	11.28730
C	17.28970	5.43920	12.36090
C	16.30630	5.51120	13.36700
C	15.16960	4.67350	13.28360
C	15.02550	3.77400	12.20590
C	8.91070	8.45950	12.60760
C	8.88210	9.55350	13.49610
C	10.03160	9.91490	14.22110
C	11.22300	9.17540	14.07640
C	11.25300	8.07440	13.19110
C	10.10330	7.72380	12.45540
C	11.10340	2.25080	18.23650
C	11.88520	2.58200	19.36280
C	12.58380	3.80030	19.42050
C	12.51620	4.71000	18.34760
C	11.73730	4.38410	17.21290
C	11.03460	3.15980	17.15940
C	16.80110	5.53720	17.92560
C	15.78230	9.89900	13.66250
C	11.83420	9.26030	18.39820
B	6.81730	-11.38240	11.34550
O	7.26290	-10.09810	11.04380
O	7.11360	-12.41700	10.46960
C	7.91940	-10.37430	9.88500
C	7.82930	-11.72200	9.55830
C	8.44440	-12.25660	8.41290
C	8.63860	-9.46850	9.07330
C	9.16260	-11.35300	7.60980
C	9.26060	-10.00230	7.92220
O	10.01420	-9.33350	7.00800
B	10.33210	-10.40250	6.17340
O	9.83830	-11.66290	6.48130
B	15.89830	2.70290	9.99390
O	14.86700	1.77260	9.88180
O	16.92460	2.67960	9.06030
C	15.31460	1.09810	8.78820
C	16.50870	1.63090	8.31670
C	17.17690	1.09740	7.20020
C	14.70990	-0.01520	8.16280
C	16.57230	-0.01000	6.58060
C	15.37870	-0.55440	7.04030
O	14.99790	-1.62300	6.28980
B	16.06310	-1.64030	5.39180
O	17.05410	-0.67610	5.50800
B	10.33250	0.87990	18.23030
O	9.48190	0.48640	17.19920
O	10.45910	0.05690	19.34050
C	9.02790	-0.68020	17.73580
C	9.60170	-0.92230	18.97820
C	9.28680	-2.06130	19.74010
C	8.08820	-1.57700	17.17590
C	8.35300	-2.95070	19.17980
C	7.76810	-2.72350	17.93980
O	6.88940	-3.71220	17.61820
B	7.01810	-4.49630	18.76390
O	7.90490	-4.09100	19.75090
C	5.45710	-12.32600	19.65500
C	17.52720	-9.22430	2.03260
H	13.71670	-10.73860	0.82550
H	15.00850	-10.52880	1.90200
H	13.30790	-5.74250	-2.41720
H	12.45180	-7.20260	-2.35140
H	17.33160	-5.30230	0.38300
H	17.71790	-6.43200	1.58680
H	11.26090	-12.37670	4.51010
H	12.82460	-12.23180	2.60160
H	13.16330	-7.96780	3.22310
H	11.60070	-8.13040	5.14000
H	8.58770	-4.48580	-2.54500
H	10.62580	-5.70840	-3.20940
H	12.13690	-5.35880	0.82350
H	10.08450	-4.12650	1.47790
H	18.13480	-1.97140	3.62800
H	18.48530	-3.70750	1.90510
H	14.72950	-5.54000	3.00810
H	14.39420	-3.79480	4.73690
H	16.69200	-7.56700	3.15280
H	15.76050	-8.98110	3.26300
H	11.39760	-8.33520	-0.70440
H	11.83210	-9.55770	0.39220
H	15.47950	-4.06080	-0.04310
H	14.09210	-4.24630	-1.00420
H	4.86330	-13.27020	17.06730
H	3.46840	-13.70720	16.20950
H	2.91330	-8.91150	20.72090
H	4.27900	-9.90750	20.61700
H	-0.90010	-10.13400	17.82850
H	-0.74720	-11.13360	16.46700
H	6.00000	-13.84090	12.25360
H	4.87270	-14.45350	14.36610
H	4.87020	-10.29790	15.55420
H	6.00890	-9.69960	13.43370
H	6.99320	-6.09380	20.98920
H	5.59960	-8.06410	21.51330
H	3.63640	-7.68090	17.68340
H	5.04000	-5.69840	17.17140
H	-2.69210	-6.52250	15.46250
H	-2.35190	-8.48290	16.93350
H	1.35050	-9.35990	14.88290
H	0.98060	-7.40980	13.39730
H	0.67320	-11.68210	14.93970
H	2.10890	-12.57210	14.80260
H	5.76420	-10.30970	18.93560
H	5.91720	-11.47610	17.71290
H	0.21230	-8.21070	18.42930
H	1.54360	-7.84500	19.41120
H	17.03620	7.36620	14.14510
H	17.24740	6.05460	15.19650
H	12.14080	10.21130	15.71290
H	13.03760	10.27180	14.27520
H	12.71320	6.68590	19.10780
H	14.18420	5.84790	19.04820
H	17.90550	4.49460	10.52390

H	18.16650	6.06900	12.41010
H	14.40260	4.71350	14.04490
H	14.15700	3.13310	12.14940
H	7.97030	10.12160	13.61690
H	9.99110	10.75960	14.89400
H	12.15370	7.48810	13.07660
H	10.13000	6.88170	11.77800
H	11.94570	1.89480	20.19510
H	13.17180	4.03440	20.29670
H	11.67420	5.06940	16.37880
H	10.43890	2.91500	16.29130
H	15.03040	4.37320	17.50190
H	16.06840	4.50020	16.16640
H	14.00150	8.97680	12.84610
H	15.36910	7.97980	12.75490
H	10.84960	8.52330	16.62290
H	11.10910	7.31120	17.77900
H	8.37710	-13.30610	8.16740
H	8.71630	-8.42200	9.32560
H	18.10800	1.51110	6.84240
H	13.78550	-0.43710	8.52790
H	9.73280	-2.24020	20.70720
H	7.63260	-1.39150	16.21470
H	-0.42990	-8.40180	20.81810
H	0.19250	-14.11340	15.13730
H	-0.21490	-13.34170	16.66580
H	1.27390	-14.25630	16.51800
H	0.80240	-9.62620	21.09510
H	-0.57180	-9.98700	20.06890
H	6.50560	-12.46940	19.91610
H	4.92150	-12.06980	20.56800
H	5.06730	-13.28380	19.31320
H	11.62560	-10.50340	-1.89520
H	16.06330	-3.75180	-2.43680
H	13.29930	-10.66240	-1.37850
H	12.85140	-9.40240	-2.51000
H	15.48650	-5.36630	-2.83130
H	16.91310	-5.16870	-1.83280
H	18.14470	-9.56600	2.86320
H	17.19950	-10.10410	1.48020
H	18.16280	-8.64440	1.36450
H	16.05690	10.36570	12.71660
H	10.89970	9.52460	18.89310
H	17.36960	4.67660	18.27790
H	16.41860	6.06060	18.80070
H	17.49690	6.21140	17.42790
H	16.70280	9.62000	14.17370
H	15.29060	10.65420	14.27460
H	12.24640	10.16980	17.96370
H	12.53180	8.92770	19.16560

Tc

Potential Energy (OPLS4): 2002.784 kJ/mol

C	6.63970	21.76580	-11.96440
C	7.09870	21.47860	-10.65320
C	8.48420	21.56640	-10.34580
C	9.41650	21.91310	-11.36480
C	8.95380	22.18200	-12.68280
C	7.57250	22.06520	-12.99080
C	5.13420	21.81600	-12.25540
C	6.09740	21.12440	-9.55310
C	8.94870	21.35860	-8.89550
C	10.91990	22.01330	-11.06180
C	9.93180	22.66210	-13.76570
C	7.08390	22.29650	-14.42150
H	4.91980	22.55120	-13.03060
H	4.60790	22.22120	-11.39100
H	7.83360	21.96130	-15.13640
H	6.21370	21.67370	-14.62830
O	6.77670	23.66760	-14.62940
H	9.44480	23.43870	-14.35650
H	10.78370	23.18560	-13.33770
H	11.47060	21.53090	-11.86900
O	11.38970	23.35820	-10.95810
H	11.20830	21.43490	-10.18510
H	9.75970	22.03770	-8.65930
H	8.18440	21.67810	-8.18840
H	6.55140	20.40780	-8.87100
O	5.67930	22.30770	-8.88650
H	5.23110	20.59910	-9.95310
C	3.17680	18.07500	-13.35990
C	4.57370	18.09870	-13.16610
C	5.22660	19.29840	-12.81300
C	4.48470	20.49130	-12.64510
C	3.08930	20.46510	-12.84170
C	2.44210	19.26710	-13.19690
B	2.42760	16.75020	-13.75500
H	5.14430	17.18930	-13.29180
H	6.29800	19.28850	-12.67320
H	2.50800	21.36840	-12.71950
H	1.37110	19.25910	-13.34510
C	11.48120	19.65650	-16.52220
C	11.50360	21.02750	-16.84940
C	10.99630	21.98560	-15.95150
C	10.45990	21.58710	-14.71000
C	10.43630	20.21330	-14.37780
C	10.94280	19.25610	-15.28120
B	12.05500	18.60040	-17.53570
H	11.91570	21.34390	-17.79760
H	11.02340	23.03270	-16.21920
H	10.03250	19.88210	-13.43170
H	10.92290	18.20660	-15.02310
C	10.22960	17.35320	-7.73260
C	9.42480	17.52290	-8.87900
C	9.00830	18.81100	-9.27760
C	9.39350	19.94720	-8.52940
C	10.20070	19.77630	-7.38660
C	10.61480	18.49030	-6.99270
B	10.69670	15.92610	-7.26240
H	9.12430	16.65640	-9.45120
H	8.38840	18.91480	-10.15670
H	10.49790	20.63520	-6.80070
H	11.22860	18.37170	-6.11080
O	3.09960	15.53900	-13.90580
O	1.05650	16.78070	-13.97940
O	12.61190	19.02080	-18.73770
O	12.02490	17.23720	-17.25730
O	10.29620	14.76480	-7.91940
O	11.48010	15.80650	-6.12120
C	5.43530	25.06740	-18.36750
C	5.59830	25.93450	-17.26960
C	6.04730	25.43180	-16.03390
C	6.34020	24.06350	-15.87360
C	6.17470	23.19470	-16.98110
C	5.72650	23.69650	-18.21910
C	4.95950	25.61060	-19.70070
H	5.38340	26.98830	-17.37030
H	6.17090	26.10360	-15.19710
H	6.38620	22.13900	-16.90990
H	5.60920	23.02300	-19.05550
C	10.19760	25.08970	-7.33650
C	11.41940	24.38870	-7.41800
C	11.83930	23.84110	-8.64520
C	11.03550	23.98410	-9.79400
C	9.84210	24.72990	-9.72590
C	9.42020	25.27520	-8.49860
C	9.70560	25.61820	-6.00100
H	12.02690	24.24530	-6.53640
H	12.76140	23.28130	-8.69910
H	9.22730	24.84210	-10.60730
H	8.48410	25.81200	-8.44910
C	3.77070	22.20000	-5.13920
C	4.06550	20.98260	-5.78510
C	4.68540	20.98120	-7.05000
C	5.02830	22.20340	-7.67920
C	4.70890	23.41430	-7.03480
C	4.08390	23.41770	-5.77410
C	3.14630	22.20380	-3.75830
H	3.82700	20.04250	-5.30920
H	4.89520	20.02750	-7.50840
H	4.96440	24.35080	-7.50830
H	3.86500	24.35790	-5.28950
H	4.42510	24.82590	-20.23760
H	4.23360	26.40230	-19.51380
C	6.12540	26.13590	-20.55330
H	9.39670	26.65640	-6.13070
C	8.53740	24.79770	-5.42170
H	10.53210	25.63750	-5.28960
H	2.45230	23.04240	-3.68570
H	2.55000	21.29980	-3.62770
C	4.21300	22.30090	-2.65790
C	5.70230	26.63630	-21.94460
H	6.64920	26.92910	-20.01810
H	6.85130	25.33160	-20.68100
C	8.85070	23.30210	-5.23390
H	8.24460	25.23040	-4.46440
H	7.67040	24.89810	-6.07630
C	3.61050	22.33290	-1.24620
H	4.89860	21.45660	-2.74440
H	4.81240	23.19870	-2.81590
C	4.88450	27.93770	-21.91020
H	6.60230	26.80320	-22.53830
H	5.14210	25.85580	-22.46150
C	7.67880	22.53380	-4.60700
H	9.09090	22.85720	-6.19970
H	9.73920	23.18690	-4.61180
H	3.01370	21.43410	-1.08400
C	4.68580	22.43820	-0.15510
H	2.92490	23.17760	-1.16310
C	4.55410	28.45750	-23.31370
H	3.95380	27.78260	-21.36410
H	5.43750	28.70410	-21.36530
C	7.92760	21.02400	-4.52920
H	7.47390	22.92170	-3.60850
H	6.77700	22.70690	-5.19250
C	4.08910	22.48220	1.25610
H	5.36920	21.59120	-0.23080
H	5.28760	23.33350	-0.31820
H	3.97510	29.37980	-23.26020
H	5.46110	28.66890	-23.88100
H	3.96790	27.73030	-23.87640
H	7.08120	20.51830	-4.06370
H	8.05740	20.59540	-5.52330
H	8.81770	20.79570	-3.94260
H	4.87520	22.55570	2.00820
H	3.43060	23.34250	1.38070
H	3.51020	21.58290	1.46910
C	13.32420	1.73690	-4.56000
C	14.49280	1.51180	-5.33540
C	15.56990	2.43550	-5.27930

C	15.44780	3.62190	-4.50770
C	14.27860	3.84740	-3.73370
C	13.20110	2.92320	-3.78940
C	12.22560	0.66770	-4.49460
C	14.60220	0.26090	-6.20870
C	16.88980	2.11280	-5.99260
C	16.58830	4.64110	-4.48550
C	14.21400	5.04900	-2.78170
C	11.92020	3.18940	-2.99660
H	11.85180	0.61010	-3.47180
H	12.63220	-0.32930	-4.65870
H	11.72110	4.25960	-2.94850
H	11.05830	2.77690	-3.51860
O	12.01640	2.64050	-1.69020
H	13.58630	4.83500	-1.91760
H	15.19700	5.19540	-2.33300
H	16.19750	5.64770	-4.34490
O	17.52220	4.32380	-3.46320
H	17.08150	4.67460	-5.45680
H	17.73780	2.55550	-5.47160
H	17.08560	1.04360	-5.90500
H	15.21240	0.46290	-7.08760
O	15.14290	-0.82420	-5.46810
H	13.62440	-0.00160	-6.61200
C	8.80990	1.20380	-7.15320
C	10.04070	1.81830	-7.46370
C	11.15470	1.65710	-6.61450
C	11.04730	0.87780	-5.43970
C	9.81570	0.26590	-5.12990
C	8.70610	0.42810	-5.98050
B	7.56210	1.36930	-8.09390
H	10.12660	2.41440	-8.36140
H	12.08760	2.13620	-6.87540
H	9.71810	-0.33450	-4.23610
H	7.76630	-0.04710	-5.73540
C	12.91120	8.84740	-4.49100
C	13.71220	8.80430	-3.33120
C	14.13030	7.57250	-2.79350
C	13.75390	6.36150	-3.40880
C	12.95530	6.40010	-4.57510
C	12.53820	7.63650	-5.11110
B	12.44870	10.23690	-5.06490
H	14.00340	9.72680	-2.84850
H	14.73990	7.56100	-1.90050
H	12.65390	5.48570	-5.06600
H	11.92540	7.66010	-6.00130
C	17.17220	3.20950	-10.20690
C	16.22570	3.84890	-9.37940
C	16.11790	3.50380	-8.01620
C	16.95840	2.51000	-7.46400
C	17.90120	1.86910	-8.29330
C	18.00650	2.21650	-9.65350
B	17.31280	3.59050	-11.72600
H	15.58280	4.61140	-9.79610
H	15.38720	4.01080	-7.40250
H	18.55230	1.10820	-7.88610
H	18.73580	1.72110	-10.27940
O	7.62650	2.12550	-9.26060
O	6.36590	0.73780	-7.77570
O	12.83360	11.40610	-4.42050
O	11.65000	10.33520	-6.20140
O	16.51620	4.57180	-12.31140
O	18.27530	2.96320	-12.50740
C	8.83810	2.95240	1.05550
C	10.03900	2.29520	1.38750
C	11.08120	2.21050	0.44520
C	10.94400	2.77660	-0.83730
C	9.73500	3.43910	-1.16600
C	8.69100	3.52540	-0.22360
C	7.71750	3.04740	2.07280
H	10.16740	1.85570	2.36590
H	11.99930	1.70570	0.70780
H	9.58150	3.89140	-2.13340
H	7.77670	4.03640	-0.48770
C	20.92460	6.71270	-2.78500
C	20.02910	7.07750	-3.81070

C	18.88300	6.29900	-4.06560
C	18.61650	5.14170	-3.29220
C	19.51900	4.78480	-2.27130
C	20.66510	5.56110	-2.01610
C	22.16070	7.54980	-2.50780
H	20.21990	7.95500	-4.41110
H	18.22490	6.61290	-4.86090
H	19.32790	3.90310	-1.67720
H	21.34430	5.26720	-1.22920
C	15.77120	-4.58650	-7.25460
C	15.22810	-3.54520	-8.03350
C	15.00360	-2.27530	-7.46610
C	15.32320	-2.03070	-6.10720
C	15.86610	-3.07870	-5.33820
C	16.09070	-4.34830	-5.90340
C	16.02160	-5.95080	-7.86730
H	14.98270	-3.71530	-9.07160
H	14.58640	-1.50650	-8.09770
H	16.11460	-2.90370	-4.30160
H	16.51200	-5.13560	-5.29530
H	6.76300	3.11830	1.54980
H	7.68620	2.11760	2.64110
C	7.89700	4.25010	3.01310
H	22.24320	7.69270	-1.42950
C	23.45560	6.91420	-3.04240
H	22.03140	8.54630	-2.93230
H	15.84880	-6.71670	-7.10980
H	15.28910	-6.13020	-8.65540
C	17.44490	-6.07370	-8.42940
C	6.74900	4.42900	4.02090
H	8.84620	4.16820	3.54440
H	7.97000	5.15500	2.40810
C	23.48090	6.75220	-4.57150
H	24.29930	7.53320	-2.73510
H	23.60450	5.94210	-2.57040
C	17.72610	-7.45770	-9.03240
H	17.60050	-5.30550	-9.18810
H	18.16480	-5.87060	-7.63520
C	6.69800	3.33620	5.10140
H	6.86730	5.39790	4.50830
H	5.79720	4.47420	3.48980
C	24.81130	6.17730	-5.07840
H	22.66530	6.09850	-4.88450
H	23.29830	7.71870	-5.04300
H	17.00630	-7.66120	-9.82670
C	19.15160	-7.57570	-9.59070
H	17.57050	-8.22360	-8.27110
C	5.59120	3.58500	6.13220
H	6.53760	2.36030	4.64280
H	7.65930	3.28160	5.61410
C	24.83930	6.02020	-6.60290
H	25.63380	6.82360	-4.76890
H	24.99120	5.20670	-4.61410
C	19.43640	-8.95920	-10.18620
H	19.31010	-6.81500	-10.35650
H	19.87390	-7.37030	-8.79930
H	5.57750	2.79740	6.88620
H	5.73700	4.53450	6.64820
H	4.60910	3.60760	5.65880
H	25.79150	5.60460	-6.93350
H	24.04900	5.35150	-6.94560
H	24.70590	6.98070	-7.10170
H	20.45340	-9.01290	-10.57580
H	19.32800	-9.74290	-9.43570
H	18.75500	-9.18430	-11.00730
C	17.03360	4.54490	-13.56790
C	18.05610	3.61020	-13.67760
C	18.74640	3.40290	-14.88780
C	18.34720	4.19090	-15.98510
C	17.32640	5.12810	-15.88280
C	16.63480	5.33490	-14.66890
H	19.54130	2.67680	-14.97020
H	15.83910	6.06040	-14.58670
C	12.23350	12.30710	-5.23450
C	11.54710	11.68940	-6.27280
C	10.85240	12.44090	-7.24650

C	10.88840	13.84680	-7.10980
C	11.57490	14.45620	-6.06660
C	12.26890	13.70820	-5.09580
H	10.32040	11.96380	-8.05630
H	12.80090	14.18440	-4.28590
C	6.35300	1.94850	-9.69970
C	5.62380	1.14000	-8.83610
C	4.27650	0.81250	-9.08640
C	3.70380	1.34720	-10.25730
C	4.42730	2.15720	-11.12410
C	5.77730	2.48530	-10.87210
H	3.71100	0.18290	-8.41590
H	6.34180	3.11590	-11.54290
C	-3.18830	5.39930	-17.08530
C	-2.75910	5.12830	-18.41150
C	-2.19870	3.86320	-18.73090
C	-1.99590	2.89880	-17.70850
C	-2.41940	3.17240	-16.38070
C	-2.98040	4.43720	-16.06150
C	-3.94300	6.69930	-16.77680
C	-2.92520	6.18790	-19.50160
C	-1.87500	3.51240	-20.18950
C	-1.34790	1.55470	-18.04260
C	-2.34270	2.07740	-15.30840
C	-3.39060	4.75110	-14.62220
H	-4.66590	6.54410	-15.97650
H	-4.57660	6.95710	-17.62600
H	-2.73070	4.24250	-13.92050
H	-3.24580	5.81120	-14.41450
O	-4.74240	4.37660	-14.39490
H	-3.21660	2.14720	-14.66010
H	-2.45550	1.09010	-15.75510
H	-0.76280	1.19980	-17.19470
O	-2.33560	0.59940	-18.40350
H	-0.62030	1.67170	-18.84500
H	-2.10770	2.46260	-20.36930
H	-2.55230	4.02820	-20.86940
H	-2.10550	6.12020	-20.21670
O	-4.17550	6.03950	-20.15960
H	-2.83500	7.18680	-19.07690
C	-1.56520	10.22270	-15.78640
C	-0.93700	8.96380	-15.88920
C	-1.68750	7.81220	-16.20530
C	-3.08100	7.90900	-16.42730
C	-3.70670	9.16790	-16.32370
C	-2.95500	10.31460	-16.00530
B	-0.74280	11.51450	-15.42900
H	0.12800	8.88560	-15.72130
H	-1.18090	6.86030	-16.27580
H	-4.77130	9.25760	-16.48900
H	-3.44620	11.27460	-15.92740
C	1.21550	1.98880	-12.78260
C	0.04290	1.29460	-12.42090
C	-1.09830	1.33850	-13.24330
C	-1.08620	2.07880	-14.44260
C	0.08670	2.78050	-14.80710
C	1.22890	2.73410	-13.98030
B	2.48140	1.91430	-11.85270
H	0.02330	0.71900	-11.50590
H	-1.98580	0.79460	-12.95150
H	0.12460	3.35500	-15.72140
H	2.12440	3.26880	-14.26440
C	2.21120	4.21570	-21.55160
C	1.79860	4.71230	-20.29760
C	0.48180	4.49810	-19.84060
C	-0.43990	3.77780	-20.63560
C	-0.02620	3.28490	-21.88980
C	1.28830	3.50310	-22.34410
B	3.67710	4.44460	-22.07160
H	2.50050	5.26110	-19.68560
H	0.19310	4.89110	-18.87630
H	-0.71990	2.73470	-22.51030
H	1.59500	3.12040	-23.30750
O	0.63100	11.46560	-15.20640
O	-1.39400	12.73490	-15.29690
O	2.43280	1.15940	-10.68710

O	3.67350	2.55340	-12.18340
O	4.62470	5.11380	-21.30270
O	4.05640	3.95770	-23.31740
C	-6.58110	4.96840	-10.65180
C	-7.28060	4.40620	-11.73760
C	-6.63420	4.22220	-12.97460
C	-5.28670	4.59390	-13.14890
C	-4.58620	5.15660	-12.05300
C	-5.23130	5.34040	-10.81380
C	-7.27590	5.16210	-9.31750
H	-8.31410	4.11170	-11.62670
H	-7.17770	3.79020	-13.80220
H	-3.55270	5.45530	-12.13450
H	-4.68320	5.76830	-9.98720
C	-1.28220	-3.35040	-19.41710
C	-0.26400	-2.44450	-19.05750
C	-0.58230	-1.11610	-18.71400
C	-1.92960	-0.67680	-18.72310
C	-2.93970	-1.59110	-19.08140
C	-2.62300	-2.91850	-19.42700
C	-0.93970	-4.77860	-19.80170
H	0.76810	-2.76260	-19.05100
H	0.22820	-0.45500	-18.44940
H	-3.97020	-1.26740	-19.09280
H	-3.41340	-3.60110	-19.70330
C	-5.31820	8.61850	-23.28720
C	-4.07020	8.81670	-22.66320
C	-3.65780	7.97250	-21.61330
C	-4.49300	6.91510	-21.17380
C	-5.73840	6.72660	-21.80430
C	-6.15180	7.56880	-22.85370
C	-5.75820	9.52030	-24.42410
H	-3.42110	9.61590	-22.98990
H	-2.69460	8.15800	-21.16410
H	-6.38280	5.92310	-21.47850
H	-7.10930	7.40200	-23.32530
H	-6.82440	6.00820	-8.79800
H	-8.31460	5.43680	-9.50300
C	-7.19860	3.90060	-8.44240
H	-1.65880	-5.44660	-19.32570
C	-0.94870	-5.01010	-21.32260
H	0.03440	-5.04510	-19.38960
H	-6.83970	9.65360	-24.37210
H	-5.32170	10.51050	-24.28630
C	-5.36040	8.95190	-25.79380
C	-7.82340	4.06970	-7.04690
H	-7.66650	3.06130	-8.95860
H	-6.14920	3.62880	-8.31970
C	0.09630	-4.17490	-22.08170
H	-0.76930	-6.06840	-21.51520
H	-1.94310	-4.79370	-21.71550
C	-5.82380	9.83860	-26.95890
H	-4.27690	8.83060	-25.83350
H	-5.78550	7.95350	-25.90670
C	-9.35520	4.20130	-7.06510
H	-7.55400	3.20200	-6.44290
H	-7.38010	4.93230	-6.54750
C	0.10270	-4.46910	-23.58820
H	-0.09970	-3.11290	-21.92690
H	1.08670	-4.36880	-21.66770
H	-5.40020	10.83770	-26.84630
C	-5.42760	9.26640	-28.32740
H	-6.90760	9.95640	-26.91550
C	-9.95120	4.28870	-5.65530
H	-9.64990	5.08780	-7.62700
H	-9.79120	3.34740	-7.58560
C	1.14360	-3.63550	-24.34370
H	0.30010	-5.52870	-23.75590
H	-0.88610	-4.27020	-24.00380
C	-5.90230	10.14570	-29.49000
H	-4.34370	9.15500	-28.37990
H	-5.84510	8.26490	-28.44140
H	-11.03700	4.38080	-5.69650
H	-9.71630	3.39850	-5.07090
H	-9.56520	5.15470	-5.11680
H	1.12060	-3.85920	-25.41060

H	0.95630	-2.56780	-24.22520
H	2.15210	-3.84140	-23.98370
H	-5.60840	9.71600	-30.44820
H	-6.98810	10.24580	-29.49150
H	-5.47320	11.14650	-29.43020
C	5.68330	5.03150	-22.15010
C	5.35030	4.36200	-23.32130
C	6.28900	4.15870	-24.35300
C	7.58610	4.66520	-24.13630
C	7.92420	5.33180	-22.96500
C	6.98290	5.53790	-21.93360
H	6.02860	3.64210	-25.26460
H	7.24390	6.05770	-21.02410
C	0.84390	12.77130	-14.89620
C	-0.33520	13.50480	-14.94580
C	-0.36520	14.88170	-14.65010
C	0.86200	15.48000	-14.30310
C	2.04620	14.75450	-14.25810
C	2.07450	13.37380	-14.55300
H	-1.28270	15.44940	-14.68560
H	2.99200	12.80590	-14.51570
C	17.03660	9.24390	-25.21790
C	17.98240	8.55560	-24.41260
C	17.92510	7.14050	-24.30650
C	16.88210	6.42250	-24.94910
C	15.94340	7.10990	-25.76250
C	15.99340	8.52540	-25.85970
C	17.18260	10.75310	-25.44990
C	19.07530	9.33520	-23.67890
C	19.02750	6.38090	-23.55730
C	16.78740	4.90540	-24.79060
C	14.92660	6.31870	-26.59700
C	14.94200	9.27300	-26.67980
H	16.85880	11.00700	-26.45920
H	18.23670	11.03020	-25.46020
H	13.98760	8.74920	-26.63660
H	14.74100	10.24700	-26.23390
O	15.37040	9.41940	-28.02620
H	14.75830	6.82430	-27.54800
H	15.35520	5.36490	-26.90450
H	15.74500	4.58960	-24.82420
O	17.54110	4.24940	-25.80030
H	17.13270	4.60680	-23.80140
H	19.23700	5.44470	-24.07540
H	19.97730	6.91040	-23.62640
H	19.32220	8.84430	-22.73810
O	20.23230	9.46140	-24.49300
H	18.70730	10.31740	-23.38420
C	15.15080	13.42270	-22.65750
C	15.15720	12.03340	-22.41370
C	15.80130	11.15240	-23.30740
C	16.45190	11.65470	-24.45890
C	16.44030	13.04290	-24.70270
C	15.79430	13.91860	-23.81000
B	14.44900	14.42110	-21.66550
H	14.66610	11.64560	-21.53230
H	15.79240	10.09320	-23.09420
H	16.93410	13.44220	-25.57760
H	15.79660	14.98190	-24.00540
C	11.01550	5.53220	-24.82630
C	11.32810	5.10270	-26.13220
C	12.59600	5.36250	-26.68620
C	13.57330	6.05510	-25.94280
C	13.26570	6.48110	-24.62990
C	11.99380	6.22100	-24.07900
B	9.58930	5.24920	-24.22790
H	10.58490	4.57500	-26.71360
H	12.81650	5.03000	-27.69100
H	13.99440	7.01310	-24.03520
H	11.76100	6.55400	-23.07740
C	18.30210	5.47180	-19.35150
C	17.52100	6.45190	-19.99830
C	17.73730	6.75520	-21.35850
C	18.74170	6.07860	-22.08940
C	19.51960	5.09810	-21.44160
C	19.30070	4.79780	-20.08420
B	18.08000	5.12210	-17.83490
H	16.75370	6.97300	-19.44310
H	17.12670	7.51200	-21.82930
H	20.29300	4.57330	-21.98530
H	19.90550	4.04570	-19.59690
O	13.87370	13.98500	-20.47520
O	14.46800	15.78450	-21.93410
O	8.63560	4.58520	-24.99060
O	9.22220	5.73110	-22.97470
O	17.11660	5.76820	-17.06380
O	18.87070	4.15050	-17.23380
C	12.97090	11.36540	-30.88810
C	14.23220	10.83330	-31.22030
C	15.00790	10.18980	-30.23770
C	14.54100	10.06620	-28.91460
C	13.27110	10.60210	-28.58490
C	12.49300	11.24580	-29.56750
C	12.13210	12.05170	-31.94880
H	14.60900	10.91300	-32.22950
H	15.97360	9.78340	-30.50060
H	12.86840	10.53190	-27.58630
H	11.52540	11.64600	-29.30210
C	17.76710	0.04780	-25.96650
C	16.98060	0.64880	-24.96300
C	16.88340	2.05140	-24.87980
C	17.57290	2.87320	-25.80560
C	18.35210	2.26250	-26.80770
C	18.45210	0.86080	-26.89060
C	17.88790	-1.46340	-26.04630
H	16.45440	0.03620	-24.24570
H	16.27430	2.46980	-24.09350
H	18.88230	2.87910	-27.51870
H	19.06090	0.41400	-27.66320
C	23.66050	11.49350	-23.13010
C	22.54180	11.39920	-22.27800
C	21.37840	10.72710	-22.70270
C	21.32110	10.13810	-23.99060
C	22.44530	10.23770	-24.83330
C	23.60810	10.90870	-24.41060
C	24.91590	12.21070	-22.67240
H	22.57200	11.84000	-21.29220
H	20.54500	10.67710	-22.01930
H	22.41340	9.79120	-25.81640
H	24.45910	10.96890	-25.07330
H	11.50180	12.80550	-31.47530
H	12.79790	12.59040	-32.62350
C	11.26560	11.05050	-32.72950
H	17.80950	-1.76230	-27.09240
C	19.20570	-1.99290	-25.45470
H	17.03980	-1.92460	-25.53850
H	25.36660	12.71440	-23.52880
H	24.64370	12.99570	-21.96560
C	25.92780	11.24900	-22.03260
C	10.34270	11.70420	-33.77200
H	11.90130	10.30720	-33.21270
H	10.64800	10.49940	-32.01890
C	19.38050	-1.68020	-23.95870
H	19.24630	-3.07260	-25.60300
H	20.04550	-1.57730	-26.01320
C	27.21640	11.95780	-21.58950
H	25.46620	10.75710	-21.17520
H	26.17430	10.45930	-22.74380
C	11.09170	12.29730	-34.97660
H	9.64270	10.94890	-34.13240
H	9.73450	12.47350	-33.29410
C	20.67290	-2.27100	-23.37850
H	19.38470	-0.59960	-23.80840
H	18.52310	-2.06440	-23.40480
H	26.97010	12.74830	-20.87910
C	28.23010	10.99610	-20.95290
H	27.67390	12.44830	-22.45000
C	10.13950	12.86580	-36.03500
H	11.76540	13.08890	-34.64790
H	11.71780	11.52960	-35.43340
C	20.84630	-1.94640	-21.89060
H	20.67670	-3.35330	-23.51400

H	21.53180	-1.88800	-23.93150
C	29.51840	11.70550	-20.52030
H	27.78070	10.50790	-20.08700
H	28.47710	10.20220	-21.65920
H	10.69540	13.28100	-36.87620
H	9.47610	12.09360	-36.42570
H	9.51990	13.66220	-35.62140
H	21.77320	-2.37210	-21.50520
H	20.88130	-0.86940	-21.72310
H	20.02440	-2.35110	-21.29920
H	30.21880	11.00060	-20.07130
H	30.01730	12.17140	-21.37070
H	29.31380	12.48350	-19.78410
C	13.51740	15.18990	-19.95760
C	13.86230	16.23320	-20.80820
C	13.59400	17.57750	-20.48250
C	12.95960	17.81340	-19.24650
C	12.61650	16.77470	-18.38970
C	12.88260	15.42760	-18.71920
H	13.86380	18.38560	-21.14570
H	12.61590	14.61840	-18.05610

11. Cartesian Coordinates and Energies of DFT optimized structures

2a

Sum of Electronic and Thermal Free Energies (M06-2X-D3/6-31+G(d.p)): -2078.623572 Hartree

The energy value was obtained from the structure without Bpin-substituents. The Bpin-substituents were added *a posteriori* of the DFT-calculation and was subjected to minimization using the OPLS4 force field.

No imaginary frequencies found.

C	0.60650	-1.26830	-0.26660
C	1.39990	-0.11230	-0.24850
C	0.79930	1.15500	-0.26500
C	-0.59870	1.26370	-0.25000
C	-1.39640	0.11040	-0.26800
C	-0.79130	-1.15420	-0.25050
C	2.91410	-0.24200	-0.15950
C	-1.24320	2.63920	-0.16160
C	-1.66090	-2.39960	-0.16110
C	2.47950	4.22990	3.54370
C	2.14700	2.87530	3.47360
C	1.87700	2.28280	2.24390
C	1.94940	3.03830	1.06710
C	2.26840	4.39360	1.13850
C	2.53620	4.98520	2.37000
C	2.42400	-4.27270	3.53560
C	1.42210	-3.30210	3.46790
C	1.04500	-2.76860	2.23930
C	1.65990	-3.21040	1.06140
C	2.66930	-4.16950	1.13030
C	3.04640	-4.70090	2.36070
C	-4.91610	0.03480	3.52800
C	-3.57530	0.42080	3.46350
C	-2.92280	0.48270	2.23560
C	-3.60890	0.16780	1.05610
C	-4.94330	-0.23090	1.12250
C	-5.59520	-0.29240	2.35170
O	-1.39530	3.13930	-1.47880
O	3.42350	-0.35310	-1.47710
O	-2.01550	-2.78460	-1.47810
C	-2.80160	6.98890	-2.14290
C	-2.71250	6.53670	-0.82840
C	-2.24590	5.25270	-0.57250
C	-1.86670	4.41940	-1.63450
C	-1.95910	4.88020	-2.94340
C	-2.42650	6.16500	-3.20310
C	7.45790	-1.06700	-2.15140
C	6.55450	-0.97460	-3.20910
C	5.20920	-0.73460	-2.94560
C	4.76730	-0.58680	-1.63520
C	5.68230	-0.67950	-0.57630
C	7.02550	-0.92030	-0.83590
C	-4.65920	-5.91740	-2.13750
C	-4.12790	-5.18640	-3.19910
C	-3.24450	-4.14250	-2.94090
C	-2.89270	-3.82950	-1.63240
C	-3.43020	-4.56880	-0.56910
C	-4.31320	-5.61160	-0.82330
C	1.27170	-2.64870	-0.30590
C	1.66140	2.42190	-0.30140
C	-2.92480	0.22410	-0.31000
B	2.78640	4.90470	4.94230
H	2.09780	2.28190	4.37610
H	1.61120	1.23560	2.21620
H	2.30740	4.98930	0.23780
H	2.78910	6.03490	2.41580
B	2.85320	-4.88020	4.93290
H	0.93550	-2.96140	4.37140
H	0.27450	-2.01120	2.21350
H	3.16280	-4.50260	0.22850
H	3.82520	-5.44870	2.40460
B	-5.66010	-0.03370	4.92360
H	-3.04070	0.67770	4.36730
H	-1.88280	0.77570	2.21110
H	-5.47510	-0.49440	0.21930
H	-6.63190	-0.59450	2.39230
H	-2.19920	2.60370	0.35960
H	-0.62810	3.30380	0.44490
H	3.36070	0.60130	0.36590
H	3.18290	-1.11000	0.44220
H	-2.54520	-2.19690	0.44300
H	-1.15400	-3.20890	0.36330
H	-3.16580	7.98690	-2.33990
H	-3.00350	7.17270	-0.00600
H	-2.18640	4.91920	0.45230
H	-1.66270	4.22560	-3.75060
H	-2.49630	6.51540	-4.22190
H	8.50350	-1.25250	-2.35030
H	6.89040	-1.08740	-4.22880
H	4.49250	-0.66030	-3.75100
H	5.37680	-0.56600	0.45130
H	7.72240	-0.98990	-0.01430
H	-5.34480	-6.72930	-2.33320
H	-4.39560	-5.42400	-4.21760
H	-2.82160	-3.56290	-3.74920
H	-3.17200	-4.34860	0.45560
H	-4.72260	-6.17720	0.00010
H	1.17600	3.15950	-0.94240
H	2.60170	2.23280	-0.81750
H	2.15360	-2.59610	-0.94620
H	0.63730	-3.36660	-0.82420
H	-3.31850	-0.56570	-0.95170
H	-3.22840	1.13230	-0.82910
O	3.11830	6.23080	5.10680
O	2.75490	4.23940	6.14740
O	3.83130	-5.83550	5.09510
O	2.29560	-4.51940	6.13900
O	-6.97650	-0.40420	5.08270
O	-5.07200	0.26960	6.13110
C	3.57320	6.38850	6.43820
C	2.82120	5.21690	7.16950
C	3.74100	-6.30850	6.42640
C	3.10820	-5.06960	7.15960
C	-7.19470	-0.59740	6.46820
C	-6.10660	0.34970	7.09420
C	3.17380	7.80900	6.88610
C	5.11520	6.25570	6.37030
C	3.58140	4.58270	8.35180
C	1.35150	5.50750	7.56410
C	5.17040	-6.67830	6.87160
C	2.85020	-7.57420	6.35960
C	2.17910	-5.40810	8.34280
C	4.09960	-3.94620	7.55360
C	-8.65710	-0.20050	6.75430
C	-6.98650	-2.11250	6.71550
C	-6.48820	1.84910	7.17230
C	-5.51250	-0.12590	8.43550
H	5.19080	-6.96710	7.92210
H	5.56340	-7.51230	6.28910
H	5.86790	-5.85110	6.73950
H	2.75220	-8.04150	7.33900
H	1.84630	-7.34470	5.99990
H	3.26790	-8.32000	5.68210
H	2.72480	-5.92050	9.13470
H	1.73830	-4.50720	8.77080
H	1.34900	-6.04760	8.04230
H	4.74940	-4.26020	8.37000
H	4.73600	-3.65550	6.71670
H	3.57440	-3.04810	7.88080

H	3.41430	7.97030	7.93660
H	3.69590	8.56940	6.30430
H	2.10770	7.99570	6.75560
H	5.56890	6.40510	7.34970
H	5.42190	5.27310	6.00930
H	5.54900	6.99270	5.69340
H	3.74930	5.31090	9.14480
H	3.02490	3.74760	8.77850
H	4.55190	4.18800	8.05070
H	1.29590	6.22520	8.38210
H	0.78010	5.91290	6.72800
H	0.83970	4.60100	7.88930
H	-8.87160	-0.23960	7.82200
H	-8.88440	0.80690	6.40520
H	-9.35520	-0.86920	6.24960
H	-7.17450	-2.37320	7.75670
H	-7.65970	-2.71240	6.10180
H	-5.97040	-2.42670	6.47340
H	-7.25710	2.02340	7.92450
H	-5.62800	2.46590	7.43520
H	-6.86690	2.22100	6.21930
H	-6.27990	-0.17580	9.20750
H	-5.05600	-1.11250	8.35490
H	-4.73160	0.55040	8.78490

2b

Sum of Electronic and Thermal Free Energies
(M06-2X-D3/6-31+G(d.p)): -1278.194509 Hartree

The energy value was obtained from the structure without Bpin-substituents. The Bpin-substituents were added *a posteriori* of the DFT-calculation and was subjected to minimization using the OPLS4 force field.

No imaginary frequencies found.

C	-1.04730	0.93730	-1.32750
C	0.28800	1.38040	-1.32580
C	1.33540	0.44230	-1.32740
C	1.05150	-0.93560	-1.32820
C	-0.28450	-1.37370	-1.32960
C	-1.33590	-0.43890	-1.32840
C	0.60310	2.88140	-1.26460
C	2.19390	-1.95910	-1.26980
C	-2.79340	-0.91670	-1.27040
C	4.28090	1.73100	2.72150
C	4.86960	2.32570	1.60300
C	4.38040	2.06300	0.32610
C	3.30100	1.19760	0.15360
C	2.70180	0.61020	1.27490
C	3.19310	0.87210	2.54990
C	-3.65090	2.82830	2.71870
C	-2.35930	2.32470	2.54910
C	-1.88220	2.03530	1.27470
C	-2.68960	2.25680	0.15210
C	-3.98250	2.75000	0.32250
C	-4.45920	3.03720	1.59880
C	-0.63690	-4.57080	2.71760
C	-0.41590	-5.37730	1.59870
C	-0.40010	-4.82180	0.32190
C	-0.61160	-3.45450	0.15010
C	-0.82100	-2.64260	1.27180
C	-0.83840	-3.19940	2.54660
C	2.67390	-2.36610	-2.66940
C	0.71700	3.50290	-2.66300
C	-3.38580	-1.12810	-2.67020
C	-2.19500	1.95410	-1.26250
C	2.79000	0.92740	-1.26170
C	-0.59160	-2.87600	-1.26490
B	4.84060	2.03080	4.17160
H	5.70970	2.99340	1.72800
H	4.84070	2.53230	-0.53080
H	1.85370	-0.05180	1.16710
H	2.72740	0.40600	3.40710
B	-4.19600	3.15630	4.16820
H	-1.72360	2.15740	3.40750
H	-0.88200	1.63840	1.16830
H	-4.61840	2.91060	-0.53540
H	-5.46060	3.42380	1.72230
B	-0.65580	-5.20600	4.16750
H	-0.25630	-6.43860	1.72320
H	-0.22340	-5.45460	-0.53530
H	-0.97160	-1.57720	1.16450
H	-1.00940	-2.56350	3.40410
H	3.02180	-1.57770	-0.67350
H	1.89530	-2.83640	-0.69810
H	-0.14170	3.40660	-0.66810
H	1.51170	3.06050	-0.69180
H	-3.40420	-0.22000	-0.69850
H	-2.87690	-1.82470	-0.67470
H	2.90620	1.82390	-1.86810
H	3.46250	0.22270	-1.74390
H	-3.02710	1.60780	-1.87300
H	-1.91990	2.89050	-1.74090
H	0.12620	-3.42430	-1.87250
H	-1.53850	-3.10580	-1.74640
H	0.94800	4.56580	-2.59830
H	-4.42200	-1.45910	-2.60780
H	3.47870	-3.09780	-2.60680
H	3.04280	-1.50470	-3.22640
H	1.86400	-2.80420	-3.25290
H	-0.21290	3.39250	-3.22120
H	1.50210	3.02180	-3.24660
H	-2.82440	-1.87810	-3.22760
H	-3.36010	-0.20730	-3.25320
O	4.32380	1.49310	5.32900
O	5.90610	2.86310	4.43450
O	-5.45450	3.65110	4.42950
O	-3.47240	2.98230	5.32660
O	-0.86350	-4.49010	5.32530
O	-0.46630	-6.54460	4.42990
C	5.23770	1.78000	6.37140
C	5.93170	3.08410	5.83270
C	-5.47440	4.05470	5.78640
C	-4.37260	3.12210	6.41010
C	-1.07120	-5.42560	6.36720
C	-0.28750	-6.67780	5.82800
C	-2.60750	-5.59280	6.47690
C	-0.50360	-4.79820	7.65650
C	1.24470	-6.65820	6.05610
C	-0.87500	-8.04580	6.22990
C	6.14940	0.53220	6.48110
C	4.41020	1.95830	7.66040
C	5.14980	4.40210	6.06080
C	7.41020	3.25790	6.23510
C	-6.90730	3.81990	6.30590
C	-5.14320	5.56810	5.77560
C	-3.57710	3.73370	7.58100
C	-4.83110	1.68400	6.75930
H	-6.97460	4.02460	7.37400
H	-7.62400	4.46520	5.79690
H	-7.24000	2.79500	6.14110
H	-5.17500	5.98750	6.78080
H	-4.15080	5.76240	5.36660
H	-5.85370	6.12700	5.16540
H	-4.23010	3.95420	8.42500
H	-2.79970	3.05310	7.92960
H	-3.07540	4.65870	7.29660
H	-5.50200	1.67960	7.61770
H	-5.35470	1.21240	5.92660
H	-3.98240	1.04400	7.00400
H	6.86910	0.63770	7.29240
H	5.56780	-0.36990	6.67490
H	6.70940	0.35770	5.56150
H	5.04220	2.27090	8.49110
H	3.62240	2.70230	7.54190
H	3.91840	1.02810	7.94690
H	5.14710	4.68370	7.11340
H	5.59060	5.22860	5.50220
H	4.11130	4.31790	5.73770
H	7.51380	3.34370	7.31650
H	8.02550	2.42020	5.90660
H	7.84020	4.15410	5.78680
H	-2.87530	-6.26930	7.28790
H	-3.09870	-4.63860	6.67100
H	-3.03820	-5.99060	5.55710
H	-0.54810	-5.50220	8.48690
H	0.53440	-4.48690	7.53810
H	-1.06410	-3.90780	7.94340
H	1.49010	-6.79690	7.10860
H	1.74090	-7.45250	5.49720
H	1.69010	-5.71620	5.73330
H	-0.85230	-8.17890	7.31120
H	-1.90810	-8.16060	5.90140
H	-0.31310	-8.86550	5.78120

3d

Sum of Electronic and Thermal Free Energies (M06-2X-D3/6-31+G(d.p)): -1631.622885Hartree

The energy value was obtained from the structure without Bpin-substituents. The Bpin-substituents were added *a posteriori* of the DFT-calculation and was subjected to minimization using the OPLS4 force field.

No imaginary frequencies found.

C	-0.98520	1.00400	-0.48850
C	0.37630	1.35720	-0.48540
C	1.36040	0.35320	-0.48700
C	0.98500	-1.00240	-0.48880
C	-0.37640	-1.35280	-0.49190
C	-1.36260	-0.35010	-0.49030
C	0.78820	2.83310	-0.42300
C	2.05480	-2.09880	-0.42810
C	-2.84700	-0.72920	-0.43020
C	4.37500	1.49600	3.55680
C	4.99360	2.05280	2.43530
C	4.49380	1.80480	1.15900
C	3.37380	0.99000	0.98970
C	2.75140	0.43400	2.11490
C	3.25020	0.68540	3.38950
C	-3.49140	3.03200	3.55300
C	-2.21950	2.47970	3.38670
C	-1.74970	2.17510	2.11270
C	-2.54600	2.42420	0.98740
C	-3.81880	2.96880	1.15550
C	-4.28740	3.27490	2.43120
C	-0.89480	-4.52550	3.55350
C	-0.69970	-5.34030	2.43550
C	-0.66230	-4.78590	1.15820
C	-0.82650	-3.41190	0.98540
C	-1.00900	-2.59280	2.10640
C	-1.04830	-3.14780	3.38190
C	2.50320	-2.54730	-1.82650
C	0.95620	3.44460	-1.82190
C	-3.45940	-0.89370	-1.82850
C	-2.06410	2.09450	-0.42630
C	2.84460	0.74190	-0.42400
C	-0.78140	-2.83250	-0.42890
B	4.94380	1.78340	5.00600
H	5.86340	2.68240	2.55830
H	4.98350	2.24610	0.30330
H	1.87990	-0.19680	2.01580
H	2.76340	0.25050	4.25100
B	-4.02860	3.37700	5.00170
H	-1.59630	2.28620	4.24840
H	-0.76230	1.74810	2.01370
H	-4.44610	3.15350	0.29610
H	-5.27410	3.69850	2.55360
B	-0.93800	-5.15840	5.00380
H	-0.57740	-6.40650	2.55990
H	-0.50480	-5.42410	0.30120
H	-1.12040	-1.52280	1.99750
H	-1.19900	-2.50570	4.23840
H	2.90770	-1.77190	0.16520
H	1.69650	-2.94960	0.14930
H	0.07370	3.40700	0.16480
H	1.69560	2.95820	0.16610
H	-3.40490	0.00620	0.14730
H	-2.99050	-1.63140	0.16300
H	3.01750	1.62490	-1.03700
H	3.46930	-0.00820	-0.90230
H	-2.91360	1.80400	-1.04250
H	-1.72770	3.01180	-0.90230
H	-0.10530	-3.42450	-1.04340
H	-1.74490	-2.99990	-0.90330
C	-4.95440	-1.22530	-1.76050
H	-2.92960	-1.67730	-2.37230
H	-3.31110	0.02000	-2.40620
C	-5.57990	-1.39710	-3.15020
H	-5.09650	-2.13860	-1.17900
H	-5.47600	-0.43280	-1.21970
C	-7.07520	-1.72570	-3.07180
H	-5.43640	-0.48430	-3.73150
H	-5.05920	-2.19060	-3.68970
H	-7.50510	-1.84530	-4.06680
H	-7.24450	-2.65260	-2.52200
H	-7.62500	-0.93200	-2.56420
C	1.41830	4.90420	-1.75320
H	0.01350	3.37900	-2.36740
H	1.67290	2.85840	-2.39920
C	1.58410	5.53260	-3.14220
H	0.69880	5.48450	-1.17180
H	2.36520	4.95770	-1.21180
C	2.05010	6.99060	-3.06160
H	2.30210	4.95120	-3.72390
H	0.63680	5.48110	-3.68240
H	1.33310	7.60160	-2.51140
H	3.01250	7.06750	-2.55370
C	3.54090	-3.67330	-1.75830
H	2.91400	-1.69620	-2.37170
H	1.63810	-2.87860	-2.40300
C	4.00050	-4.13040	-3.14810
H	4.40320	-3.33610	-1.17940
H	3.11890	-4.52140	-1.21500
C	5.03720	-5.25670	-3.06970
H	3.13800	-4.46730	-3.72650
H	4.42270	-3.28250	-3.69080
H	5.35320	-5.57000	-4.06510
H	5.92520	-4.93520	-2.52370
H	4.62980	-6.13020	-2.55870
H	2.16250	7.42300	-4.05620
O	4.39740	1.28390	6.16690
O	-5.26720	3.92090	5.26000
O	-1.12540	-4.43500	6.16040
O	6.04760	2.56540	5.26430
O	-3.31720	3.17020	6.16230
O	-0.79240	-6.50220	5.26790
C	5.32020	1.53770	7.21000
C	-5.27640	4.31990	6.61840
C	6.07920	2.80020	6.66010
C	-4.21590	3.34100	7.24260
C	-0.62060	-6.63930	6.66650
C	6.16910	0.24810	7.33760
C	4.49730	1.77070	8.49320
C	5.36220	4.15740	6.87070
C	7.56310	2.90570	7.06640
C	-6.71970	4.14160	7.13130
C	-4.88400	5.81860	6.61490
C	-3.40200	3.91470	8.42000
C	-4.73330	1.92080	7.58290
C	-2.90610	-5.48020	7.30910
C	-0.78060	-4.75160	8.49250
C	0.91110	-6.66830	6.89760
C	-1.25210	-7.98740	7.06880
H	6.89020	0.32740	8.15070
H	6.72320	0.03580	6.42230
H	5.14050	2.06120	9.32340
H	3.74750	2.55120	8.36300
H	5.36970	4.45050	7.92010
H	5.84500	4.95500	6.30480
H	4.32200	4.12070	6.54420
H	7.66720	2.99770	8.14710
H	8.13770	2.03540	6.74890
H	8.03790	3.77500	6.61040
H	-6.78330	4.34630	8.19970
H	-7.40780	4.81620	6.62090
H	-7.09260	3.13120	6.96260
H	-4.90200	6.23490	7.62170
H	-3.88320	5.97410	6.20980
H	-5.56920	6.40840	6.00480
H	-4.04950	4.15690	9.26230
H	-2.65380	3.20190	8.76850
H	-2.86250	4.82040	8.14250
H	-5.40770	1.93950	8.43850

H	-5.27180	1.47460	6.74580
H	-3.91220	1.24610	7.82810
H	1.15000	-6.81360	7.95070
H	1.38280	-7.47850	6.34030
H	1.38690	-5.74130	6.57470
H	-1.23640	-8.11950	8.15030
H	-2.28750	-8.06990	6.73770
H	-0.71530	-8.82520	6.62260
H	5.54320	-0.62210	7.53920
H	3.95910	0.86910	8.78770
C	-1.36510	-5.36230	7.20270
H	-3.36720	-4.51070	7.50140
H	-3.34730	-5.86490	6.38870
H	-3.19730	-6.14720	8.12000
H	-1.31280	-3.84330	8.77700
H	-0.84960	-5.45260	9.32380
H	0.26710	-4.47400	8.37610

Ta

Sum of Electronic and Thermal Free Energies
(M06-2X-D3/3-21G: -11729.23898 Hartree

No imaginary frequencies found.

C	2.97530	9.14410	-8.97280
C	4.03410	8.32720	-9.40030
C	5.25180	8.34380	-8.69930
C	5.44920	9.25300	-7.64790
C	4.38520	10.06710	-7.22420
C	3.16650	10.05600	-7.92090
C	1.63490	9.07160	-9.66920
C	3.91330	7.52770	-10.69070
C	6.36510	7.40540	-9.10730
C	6.83960	9.45580	-7.06060
C	4.56860	10.98550	-6.03710
C	2.10950	11.11290	-7.62990
O	1.67780	10.05860	-10.76770
O	7.15360	8.11200	-10.13780
O	5.09010	12.26180	-6.56880
C	2.49620	3.42450	-10.32070
C	2.91290	3.94740	-11.56220
C	3.36660	5.25720	-11.66590
C	3.41930	6.09040	-10.53480
C	3.00820	5.57460	-9.29820
C	2.55270	4.26120	-9.19200
C	7.85380	7.07110	-3.55830
C	8.73830	8.04640	-4.06290
C	8.40090	8.80260	-5.17930
C	7.16990	8.61130	-5.83100
C	6.28660	7.64430	-5.33210
C	6.62320	6.88450	-4.21260
C	-0.93370	10.07700	-4.68930
C	0.10330	9.17500	-4.98650
C	1.08230	9.49590	-5.92590
C	1.05340	10.72650	-6.59590
C	0.01860	11.63180	-6.30220
C	-0.95790	11.31420	-5.36550
C	-1.60830	10.61790	-13.31630
C	-0.42370	11.34980	-13.46530
C	0.64590	11.13920	-12.60330
C	0.54730	10.19040	-11.57530
C	-0.63220	9.45750	-11.42200
C	-1.70360	9.67570	-12.29400
C	10.50980	6.32500	-11.91240
C	10.08670	7.61350	-12.26130
C	8.97030	8.17560	-11.65350
C	8.25730	7.45550	-10.68410
C	8.67490	6.16960	-10.33220
C	9.79940	5.61170	-10.94890
C	5.92550	15.49470	-4.02760
C	6.12360	15.57160	-5.41180
C	5.83640	14.48150	-6.22470
C	5.34500	13.29350	-5.66430
C	5.14550	13.21130	-4.28400
C	5.43700	14.31310	-3.47340
B	1.99680	1.98830	-10.20290
O	1.89660	1.05750	-11.27330
B	8.22120	6.23910	-2.33410
O	7.40400	5.23370	-1.74650
B	-2.00510	9.72390	-3.66300
O	-3.09140	10.55850	-3.28170
O	1.55540	1.36650	-9.00200
O	-2.07710	8.50390	-2.93480
O	9.43350	6.34270	-1.59810
C	1.18580	0.05420	-9.34590
C	1.39220	-0.13280	-10.72010
C	1.11970	-1.32990	-11.36650
C	0.69180	-0.94200	-8.51620
C	9.35210	5.39670	-0.56100
C	8.12420	4.72570	-0.65090
C	7.74480	3.72770	0.23520
C	10.29160	5.11950	0.42160
C	-3.21150	8.59940	-2.10960
C	-3.82490	9.84270	-2.31910
C	-4.97280	10.23880	-1.64780
C	-3.70100	7.65960	-1.21390
C	0.62560	-2.32610	-10.53670
C	0.41930	-2.13910	-9.16250
O	-0.08560	-3.32910	-8.60920
B	-0.18720	-4.25960	-9.68040
O	0.25510	-3.63820	-10.88060
C	8.68450	3.45030	1.21770
C	9.91230	4.12130	1.30750
O	10.63290	3.61220	2.40250
B	9.81700	2.60610	2.98900
O	8.60340	2.50330	2.25380
C	-5.46200	9.29910	-0.75190
C	-4.84910	8.05550	-0.54300
O	-5.58310	7.33910	0.41890
B	-6.66890	8.17500	0.80120
O	-6.59590	9.39490	0.07420
H	4.89830	7.52760	-11.17280
H	3.24220	8.07680	-11.36260
H	6.94190	10.51940	-6.81390
H	7.57000	9.25260	-7.85350
H	2.62850	12.02260	-7.30410
H	1.62030	11.36160	-8.57950
H	0.82560	9.31540	-8.97420
H	1.45910	8.06830	-10.06910
H	6.99850	7.15880	-8.24990
H	5.95550	6.47670	-9.51610
H	3.61610	11.15110	-5.52460
H	5.27790	10.55590	-5.32320
H	2.87640	3.31340	-12.44100
H	3.68490	5.64590	-12.62780
H	3.05060	6.21040	-8.42190
H	2.23650	3.87270	-8.23020
H	9.69030	8.20220	-3.56800
H	9.09140	9.55030	-5.55620
H	5.33480	7.49480	-5.82760
H	5.93220	6.13860	-3.83550
H	0.13530	8.21960	-4.47440
H	1.87700	8.79380	-6.14830
H	-0.01350	12.58730	-6.81570
H	-1.75170	12.01960	-5.14690
H	-2.44120	10.78270	-13.98880
H	-0.33570	12.08580	-14.25610
H	1.57120	11.69200	-12.69830
H	-0.72980	8.72110	-10.63590
H	-2.61430	9.10170	-12.16660
H	11.38000	5.88800	-12.38670
H	10.62980	8.17980	-13.00910
H	8.62410	9.16920	-11.90590
H	8.13950	5.59650	-9.58750
H	10.11520	4.61320	-10.66860
H	6.14950	16.34490	-3.39510
H	6.50290	16.48440	-5.85650
H	5.98100	14.51650	-7.29650
H	4.76760	12.30480	-3.83100
H	5.27860	14.23900	-2.40360
H	1.27830	-1.47380	-12.42390
H	0.53280	-0.79800	-7.45890
H	6.80020	3.21120	0.16590
H	11.23650	5.63560	0.49060
H	-5.44470	11.19550	-1.80880
H	-3.22910	6.70290	-1.05300
C	-3.16700	-11.54880	-5.43690
C	-4.14990	-10.88410	-6.18730
C	-3.78790	-10.21050	-7.36620
C	-2.47370	-10.29530	-7.85260
C	-1.49430	-10.96090	-7.09630
C	-1.84980	-11.63670	-5.91830
C	-3.53340	-12.20630	-4.12530
C	-5.61850	-11.00210	-5.80210
C	-4.82890	-9.42430	-8.13100
C	-2.13940	-9.79120	-9.25030
C	-0.06040	-10.98150	-7.57590

C	-0.85290	-12.56770	-5.24100
C	-7.21360	-7.88320	-3.21180
C	-8.04270	-8.89600	-3.73640
C	-7.52180	-9.88340	-4.56460
C	-6.15590	-9.89450	-4.89740
C	-5.32820	-8.88880	-4.38010
C	-5.84860	-7.89780	-3.54890
C	-0.69080	-5.69410	-9.55920
C	-0.75320	-6.53710	-10.68780
C	-1.21360	-7.84370	-10.57470
C	-1.62840	-8.35380	-9.33200
C	-1.56580	-7.52060	-8.20680
C	-1.10460	-6.20960	-8.31810
C	1.61340	-10.83700	-2.09400
C	1.72090	-12.19850	-2.44510
C	0.92760	-12.73810	-3.45090
C	0.00040	-11.93790	-4.14120
C	-0.10960	-10.58400	-3.79640
C	0.68480	-10.04080	-2.78750
O	-3.94190	-13.58890	-4.44820
O	-5.45950	-10.36880	-9.07610
O	0.07330	-12.16520	-8.45030
C	-5.17100	-16.25060	-1.44630
C	-5.12910	-16.62510	-2.79510
C	-4.71680	-15.71800	-3.76400
C	-4.33850	-14.41810	-3.39810
C	-4.37830	-14.03920	-2.05390
C	-4.79460	-14.95840	-1.08550
C	-8.50730	-9.12850	-11.67990
C	-8.03530	-10.44700	-11.66850
C	-7.02420	-10.82410	-10.79270
C	-6.46730	-9.88560	-9.91190
C	-6.93460	-8.56900	-9.91890
C	-7.95240	-8.19810	-10.80350
C	3.72330	-13.10490	-10.29470
C	2.57960	-13.89290	-10.47380
C	1.38640	-13.55270	-9.84760
C	1.31790	-12.41540	-9.03000
C	2.45590	-11.62560	-8.84790
C	3.65250	-11.97540	-9.48170
C	-11.38080	4.36550	4.90970
C	-11.47920	3.17540	5.64970
C	-10.78900	3.05510	6.86640
C	-10.09740	4.15630	7.39860
C	-10.00450	5.34380	6.65510
C	-10.69260	5.47010	5.43650
C	-12.05360	4.46890	3.55940
C	-12.43610	2.07910	5.20060
C	-10.82170	1.74880	7.62770
C	-9.56890	4.10320	8.82580
C	-9.19370	6.50530	7.18460
C	-10.80380	6.83120	4.76270
C	-10.80090	-0.99280	2.57960
C	-12.16880	-0.97230	2.92140
C	-12.67850	0.01580	3.75560
C	-11.84090	1.01680	4.27800
C	-10.48100	1.00200	3.93940
C	-9.96770	0.01160	3.10300
C	-5.47980	2.68980	9.33950
C	-6.29300	3.00090	10.44870
C	-7.59730	3.44760	10.27130
C	-8.13480	3.59980	8.98120
C	-7.33120	3.28990	7.87560
C	-6.02260	2.84200	8.05130
C	-7.73830	7.81800	1.82820
C	-8.77150	8.72450	2.14310
C	-9.74320	8.39720	3.08150
C	-9.71800	7.15400	3.73750
C	-8.69380	6.24910	3.42710
C	-7.71770	6.57570	2.48660
O	-13.42470	4.95920	3.81010
O	-11.99160	1.81600	8.52750
O	-10.11330	7.32050	8.00460
C	-16.12140	5.58260	0.65640
C	-16.47280	5.82830	1.98950
C	-15.55470	5.61120	3.01000
C	-14.26660	5.14340	2.71240
C	-13.91050	4.89660	1.38420
C	-14.84070	5.11790	0.36350
C	-12.95870	-1.37300	11.07760
C	-13.72550	-0.20190	11.03410
C	-13.37670	0.83540	10.17740
C	-12.25200	0.71750	9.34800
C	-11.48340	-0.44850	9.38740
C	-11.84170	-1.48740	10.25250
C	-8.79660	10.80650	9.93340
C	-10.12000	10.36000	10.03480
C	-10.52270	9.20060	9.38270
C	-9.60520	8.46720	8.61650
C	-8.28360	8.90800	8.51200
C	-7.88700	10.07580	9.17150
C	8.50170	-3.47720	9.39960
C	9.39590	-3.88450	8.39680
C	10.20770	-2.93060	7.76010
C	10.20790	-1.59720	8.19980
C	9.31120	-1.19610	9.20440
C	8.49870	-2.14480	9.84560
C	7.56570	-4.48330	10.03060
C	9.58310	-5.36550	8.09620
C	11.11200	-3.35130	6.62350
C	11.26080	-0.62300	7.68850
C	9.25130	0.25620	9.62160
C	7.72360	-1.76610	11.10060
C	7.10480	-7.04640	4.93170
C	8.04540	-7.83610	5.62450
C	8.83050	-7.28720	6.63160
C	8.70380	-5.93170	6.98260
C	7.77020	-5.14270	6.29710
C	6.98200	-5.69120	5.28620
C	10.18240	1.76810	4.20970
C	11.41630	1.94660	4.86840
C	11.74680	1.17950	5.97950
C	10.85780	0.20770	6.47120
C	9.62910	0.02830	5.82140
C	9.29580	0.79630	4.70660
C	3.62550	-0.40040	10.53440
C	4.17680	-0.55220	11.82340
C	5.48470	-0.99200	11.99000
C	6.28780	-1.29500	10.87650
C	5.74380	-1.14770	9.59340
C	4.43210	-0.70640	9.42400
O	8.28690	-5.06420	11.18200
O	12.39330	-3.76000	7.23510
O	10.24900	0.42580	10.69810
C	6.51290	-7.99630	13.60340
C	7.80980	-7.52510	13.84240
C	8.36710	-6.55280	13.02040
C	7.63400	-6.03490	11.94290
C	6.33970	-6.50140	11.69990
C	5.78660	-7.47990	12.53210
C	15.59630	-5.10210	4.88000
C	15.68870	-5.01670	6.27470
C	14.60810	-4.56770	7.02460
C	13.41430	-4.19590	6.38940
C	13.31670	-4.27930	4.99820
C	14.40900	-4.73210	4.25140
C	10.83830	4.14530	12.54880
C	11.56820	3.00970	12.92170
C	11.34760	1.79280	12.28760
C	10.39060	1.69210	11.26740
C	9.65960	2.82180	10.89150
C	9.88800	4.04240	11.53490
B	-10.23840	-2.07660	1.66580
O	-8.87950	-2.18840	1.26020
O	-10.98660	-3.13710	1.08450
C	-8.80380	-3.32100	0.43040
C	-10.07850	-3.89510	0.32420
C	-10.33190	-5.03230	-0.42930
C	-7.68790	-3.84130	-0.20910
C	-9.21580	-5.55280	-1.06860
C	-7.94120	-4.97850	-0.96250
O	-7.03280	-5.73670	-1.72210

B	-7.78100	-6.79860	-2.30210
O	-9.13960	-6.68620	-1.89740
B	6.24220	-7.64490	3.82560
O	5.27140	-6.93630	3.06450
O	6.27830	-8.99810	3.39010
C	4.71730	-7.86350	2.16430
C	5.32630	-9.11100	2.36140
C	4.99300	-10.23690	1.62220
C	3.73040	-7.64920	1.21300
C	4.00570	-10.02270	0.67120
C	3.39720	-8.77510	0.47370
O	2.44520	-8.88780	-0.55490
B	2.48020	-10.24200	-0.98930
O	3.45050	-10.95010	-0.22820
B	2.19130	0.08350	10.34820
O	1.54130	0.27110	9.09680
O	1.29070	0.42050	11.39580
C	0.24220	0.72380	9.38710
C	0.09050	0.81430	10.77800
C	-1.08620	1.23430	11.38150
C	-0.77150	1.04660	8.49650
C	-2.09970	1.55750	10.49080
C	-1.94810	1.46700	9.09990
O	-3.14760	1.86200	8.48190
B	-4.04790	2.20070	9.53010
O	-3.39850	2.01180	10.78090
H	-6.20210	-11.03470	-6.73000
H	-5.75900	-11.97540	-5.31610
H	-1.39270	-10.47150	-9.67710
H	-3.04010	-9.89700	-9.86750
H	-0.20180	-12.98220	-6.02030
H	-1.41670	-13.41370	-4.82960
H	-9.09770	-8.89690	-3.48620
H	-8.17200	-10.65630	-4.96150
H	-4.27550	-8.88940	-4.63640
H	-5.19870	-7.12470	-3.15390
H	-0.43590	-6.15380	-11.65110
H	-1.25560	-8.48130	-11.45190
H	-1.87930	-7.90950	-7.24540
H	-1.06120	-5.57350	-7.44090
H	2.43250	-12.82470	-1.91890
H	1.02060	-13.78790	-3.70980
H	-0.82240	-9.96230	-4.32480
H	0.59100	-8.99190	-2.52870
H	-2.67650	-12.21440	-3.44490
H	-4.35680	-11.67270	-3.64080
H	-4.36660	-8.59600	-8.67650
H	-5.58030	-9.01500	-7.44880
H	0.62990	-11.05640	-6.73020
H	0.17200	-10.07050	-8.13550
H	-5.49270	-16.95830	-0.69210
H	-5.41920	-17.62690	-3.09010
H	-4.67630	-15.98350	-4.81220
H	-4.09110	-13.04190	-1.74970
H	-4.82210	-14.65430	-0.04540
H	-9.29530	-8.83580	-12.36300
H	-8.45770	-11.18120	-12.34480
H	-6.64270	-11.83630	-10.76530
H	-6.51980	-7.82930	-9.24770
H	-8.30770	-7.17400	-10.80100
H	4.65270	-13.37110	-10.78300
H	2.62020	-14.77430	-11.10330
H	0.48960	-14.14570	-9.97040
H	2.42530	-10.74430	-8.22180
H	4.52970	-11.35600	-9.33380
H	-12.83990	1.60000	6.10100
H	-13.28490	2.56390	4.70340
H	-9.65350	5.11100	9.25070
H	-10.24710	3.46680	9.40690
H	-10.80210	7.59280	5.55200
H	-11.78950	6.88630	4.28490
H	-12.82360	-1.73990	2.52430
H	-13.73350	0.01910	4.00990
H	-9.83110	1.77310	4.33560
H	-8.91360	0.00950	2.84830
H	-5.88840	2.88750	11.44820

H	-8.21150	3.68320	11.13440
H	-7.74100	3.40170	6.87890
H	-5.40950	2.60590	7.18850
H	-8.80030	9.68630	1.64330
H	-10.53200	9.10520	3.31430
H	-8.66920	5.28920	3.92890
H	-6.92950	5.86800	2.25480
H	-11.51550	5.16680	2.91080
H	-12.08590	3.49150	3.06860
H	-9.90450	1.61830	8.20990
H	-10.92140	0.90240	6.94130
H	-8.79130	7.10280	6.36100
H	-8.35860	6.14850	7.79510
H	-16.83770	5.75220	-0.13810
H	-17.46540	6.19000	2.23190
H	-15.80250	5.79370	4.04740
H	-12.92260	4.53560	1.13230
H	-14.55440	4.92340	-0.66380
H	-13.23160	-2.18070	11.74550
H	-14.59700	-0.09920	11.67030
H	-13.95330	1.74950	10.12560
H	-10.61200	-0.56040	8.75660
H	-11.23870	-2.38800	10.27520
H	-8.48390	11.71020	10.44210
H	-10.83820	10.91840	10.62410
H	-11.53930	8.83540	9.44590
H	-7.55970	8.35770	7.92640
H	-6.85920	10.40920	9.08400
H	10.63950	-5.52210	7.84610
H	9.40180	-5.91910	9.02560
H	11.52590	0.04370	8.51780
H	12.16450	-1.20020	7.45700
H	8.29090	-0.98310	11.61880
H	7.72490	-2.63780	11.76610
H	8.15120	-8.88280	5.36200
H	9.55050	-7.90710	7.15600
H	7.66810	-4.09740	6.56280
H	6.26290	-5.07130	4.76230
H	12.10970	2.69300	4.49730
H	12.70020	1.32780	6.47620
H	8.93810	-0.71670	6.19760
H	8.34270	0.64820	4.21090
H	3.56620	-0.32110	12.68910
H	5.89560	-1.10440	12.98820
H	6.35670	-1.38300	8.73140
H	4.02180	-0.59540	8.42630
H	6.64590	-3.99580	10.36790
H	7.30160	-5.26810	9.31530
H	11.27280	-2.52130	5.92870
H	10.67380	-4.18760	6.07050
H	8.25290	0.51370	9.98770
H	9.49160	0.90990	8.77760
H	6.07960	-8.75410	14.24460
H	8.38620	-7.91780	14.67210
H	9.36700	-6.17310	13.18470
H	5.75650	-6.11580	10.87470
H	4.78160	-7.83510	12.33480
H	16.43920	-5.45210	4.29660
H	16.60610	-5.30110	6.77700
H	14.65510	-4.49270	8.10300
H	12.40540	-3.99800	4.48800
H	14.32290	-4.79310	3.17250
H	11.01100	5.09310	13.04380
H	12.31040	3.07510	13.70880
H	11.89850	0.90160	12.55800
H	8.91690	2.76670	10.10720
H	9.31550	4.91290	11.23570
H	-11.31270	-5.47430	-0.51080
H	-6.70700	-3.39960	-0.12720
H	5.46130	-11.19690	1.77410
H	3.26190	-6.68930	1.06120
H	-1.20280	1.30420	12.45180
H	-0.65470	0.97730	7.42620

Ta* (unoptimized)

Unoptimized structure.

C	3.99120	-12.50370	2.27750
C	2.67570	-12.90320	1.96580
C	1.66580	-12.85410	2.94900
C	1.98630	-12.45180	4.26100
C	3.29940	-12.04740	4.57860
C	4.30480	-12.09780	3.59180
C	5.07220	-12.50520	1.19660
C	2.34390	-13.41900	0.56980
C	0.22840	-13.22750	2.58940
C	0.91610	-12.49110	5.34650
C	3.63160	-11.55520	5.98670
C	5.74110	-11.73770	3.95660
O	5.73580	-13.74530	1.09690
O	-0.04150	-14.59880	2.77600
O	4.02860	-12.60130	6.84460
C	0.88160	-10.53070	-2.34840
C	0.91240	-11.91380	-2.62760
C	1.37090	-12.81930	-1.68000
C	1.81780	-12.37910	-0.42180
C	1.78690	-11.00890	-0.13690
C	1.32480	-10.09640	-1.08730
C	-1.44810	-8.88990	6.00860
C	-1.71630	-10.08940	6.70090
C	-0.95460	-11.22710	6.47010
C	0.10030	-11.21070	5.54080
C	0.36730	-10.02550	4.84480
C	-0.39560	-8.87950	5.07700
C	6.96410	-7.57700	3.51250
C	5.69760	-7.99040	3.06330
C	5.28310	-9.31530	3.21020
C	6.12530	-10.26310	3.80510
C	7.38980	-9.85240	4.26170
C	7.80330	-8.53430	4.11940
B	0.37300	-9.57410	-3.42300
O	-0.07060	-10.00670	-4.70540
B	-2.29560	-7.65630	6.30270
O	-2.15330	-6.35950	5.74080
B	7.46020	-6.14360	3.35170
O	6.77170	-5.06140	2.74100
O	0.26250	-8.16040	-3.34640
O	8.73480	-5.69340	3.79730
O	-3.37550	-7.64620	7.23040
C	-0.24930	-7.73150	-4.58760
C	-0.44920	-8.85170	-5.40710
C	-0.94410	-8.76190	-6.69930
C	-0.53170	-6.43690	-5.00180
C	-3.88540	-6.33840	7.23400
C	-3.14700	-5.55610	6.33430
C	-3.40750	-4.20970	6.11950
C	-4.93870	-5.83520	7.98250
C	8.82140	-4.33570	3.44980
C	7.63490	-3.95000	2.80930
C	7.41890	-2.66210	2.33840
C	9.87800	-3.46460	3.66750
C	-1.22320	-7.46850	-7.11390
C	-1.02540	-6.34650	-6.29620
O	-1.39860	-5.19060	-7.01070
B	-1.82870	-5.63020	-8.29130
O	-1.72280	-7.04840	-8.35650
C	-4.46020	-3.70510	6.87050
C	-5.19870	-4.49020	7.76760
O	-6.18530	-3.69360	8.37000
B	-6.03690	-2.38640	7.82540
O	-4.96800	-2.39090	6.89030
C	8.47730	-1.79020	2.55470
C	9.66240	-2.17850	3.19610
O	10.53230	-1.07870	3.25780
B	9.85630	0.00700	2.63160
O	8.57720	-0.43100	2.19650
H	1.60180	-14.22000	0.64750

H	3.23640	-13.87900	0.13250
H	1.38290	-12.74150	6.30520
H	0.21750	-13.30660	5.13370
H	5.93810	-12.03460	4.99120
H	6.42910	-12.32790	3.34190
H	5.79950	-11.71530	1.40750
H	4.62260	-12.25370	0.23100
H	-0.46210	-12.63310	3.19560
H	0.03500	-12.95540	1.54750
H	4.42640	-10.80530	5.92970
H	2.76010	-11.04650	6.40940
H	0.57150	-12.26200	-3.59600
H	1.38930	-13.88040	-1.90960
H	2.12380	-10.66780	0.83460
H	1.30710	-9.03750	-0.85370
H	-2.52660	-10.11150	7.42090
H	-1.17130	-12.14240	7.01240
H	1.17600	-10.00920	4.12470
H	-0.17610	-7.96630	4.53470
H	5.03870	-7.26680	2.59590
H	4.30540	-9.62590	2.86290
H	8.04850	-10.57840	4.72830
H	8.78130	-8.22790	4.47290
H	-1.09590	-9.62450	-7.32910
H	-0.37740	-5.57400	-4.37280
H	-2.83850	-3.60610	5.42960
H	-5.50560	-6.43800	8.67470
H	6.50720	-2.36490	1.84400
H	10.79090	-3.76250	4.15910
C	-4.29560	1.83000	-12.42990
C	-3.05010	1.33850	-12.87020
C	-2.83190	-0.05130	-12.97640
C	-3.87920	-0.94800	-12.68690
C	-5.12860	-0.46250	-12.24680
C	-5.34170	0.92690	-12.14580
C	-4.51870	3.33240	-12.26090
C	-1.94270	2.30660	-13.27440
C	-1.46540	-0.57930	-13.41020
C	-3.67350	-2.44580	-12.88220
C	-6.24500	-1.43890	-11.87800
C	-6.71780	1.46110	-11.76420
C	0.99410	3.38000	-10.24000
C	1.22500	3.62760	-11.60940
C	0.28780	3.25700	-12.56550
C	-0.91200	2.62770	-12.19010
C	-1.14040	2.36560	-10.83370
C	-0.20110	2.73820	-9.87130
C	-2.31810	-4.76610	-9.44920
C	-2.68530	-5.38490	-10.66250
C	-3.12240	-4.62810	-11.74170
C	-3.20950	-3.22810	-11.65050
C	-2.85230	-2.60760	-10.44720
C	-2.41100	-3.36610	-9.36120
C	-7.50930	2.17080	-7.53770
C	-8.41290	2.58510	-8.53920
C	-8.13800	2.35680	-9.88110
C	-6.95260	1.71010	-10.27290
C	-6.04920	1.30160	-9.28470
C	-6.32480	1.52670	-7.93470
O	-1.36220	-0.72680	-14.80870
O	-7.05870	-1.77400	-12.97970
C	11.38510	6.89910	0.35210
C	10.63390	8.05790	0.05990
C	9.98010	8.75930	1.09220
C	10.11390	8.32280	2.42800
C	10.85980	7.16540	2.72490
C	11.51660	6.47050	1.68710
C	12.05330	6.12490	-0.78320
C	10.56150	8.55700	-1.37910
C	9.13100	9.99160	0.77950
C	9.47430	9.12850	3.55410
C	10.96710	6.66020	4.16270
C	12.40140	5.27570	2.03020
C	7.57820	6.71540	-3.96640
C	8.68630	7.40760	-4.49760
C	9.63880	7.97330	-3.65880

C	9.51950	7.86810	-2.26240	O	-1.60070	8.22280	7.21650
C	8.42770	7.17180	-1.72880	C	-0.50290	6.98360	5.64100
C	7.46820	6.60600	-2.56890	C	-0.37570	8.03800	6.55690
C	5.30270	8.29590	4.50620	C	0.80360	8.74600	6.73220
C	6.00100	9.36280	5.11090	C	0.54380	6.55710	4.83520
C	7.32790	9.61580	4.78880	C	1.84820	8.32150	5.92520
C	8.00790	8.81140	3.85760	C	1.72490	7.26480	5.01150
C	7.32220	7.74880	3.25700	O	2.95850	7.08400	4.35460
C	5.98660	7.49550	3.57510	B	3.84430	8.05860	4.88620
C	10.46630	1.39600	2.47200	O	3.15520	8.82970	5.86490
C	11.76620	1.63310	2.96570	O	9.88960	11.18010	0.76790
C	12.36110	2.88070	2.82820	O	-4.99100	3.94510	-13.43970
C	11.68200	3.93500	2.19350	H	-1.41260	1.91050	-14.14630
C	10.38820	3.70880	1.70750	H	-2.38840	3.25220	-13.60110
C	9.78880	2.45550	1.84320	H	-4.60550	-2.89560	-13.23880
O	13.36620	6.57020	-1.04000	H	-2.93960	-2.60810	-13.67860
O	12.06180	7.21940	4.85330	H	-7.48590	0.76300	-12.11400
C	-9.75140	5.64050	7.09950	H	-6.90110	2.39890	-12.29820
C	-10.54030	4.50190	6.82450	H	2.14530	4.11670	-11.90790
C	-10.40170	3.33690	7.60240	H	0.47660	3.45930	-13.61530
C	-9.49940	3.32520	8.68950	H	-2.05780	1.86910	-10.54130
C	-8.70880	4.45560	8.96730	H	-0.39380	2.53780	-8.82300
C	-8.85500	5.62270	8.18440	H	-2.61960	-6.46400	-10.74390
C	-9.89340	6.88230	6.21990	H	-3.39900	-5.11900	-12.66980
C	-11.55890	4.55940	5.68940	H	-2.92070	-1.52930	-10.37180
C	-11.21810	2.08390	7.28750	H	-2.13250	-2.87290	-8.43630
C	-9.42120	2.09030	9.58210	H	-9.33000	3.08340	-8.24640
C	-7.69890	4.43960	10.11480	H	-8.84420	2.67830	-10.64070
C	-8.06180	6.87280	8.55240	H	-5.13410	0.80410	-9.58250
C	-10.07740	3.81760	1.65840	H	-5.61920	1.20030	-7.17860
C	-11.29340	4.49550	1.88190	H	-5.23100	3.50380	-11.44850
C	-11.74220	4.74240	3.17400	H	-3.58250	3.80740	-11.95110
C	-10.99620	4.31910	4.28680	H	-1.27670	-1.54220	-12.92520
C	-9.78330	3.65060	4.07150	H	-0.68610	0.10130	-13.05560
C	-9.33060	3.40270	2.77560	H	-6.86280	-1.00190	-11.08740
C	-6.90960	-1.20240	8.22990	H	-5.80670	-2.34830	-11.45610
C	-7.92900	-1.39760	9.18430	H	10.35160	9.63200	-1.38340
C	-8.73510	-0.34040	9.58860	H	11.54170	8.44180	-1.85240
C	-8.55060	0.94750	9.05790	H	10.04780	8.98240	4.47500
C	-7.54430	1.14700	8.10350	H	9.54820	10.19630	3.32260
C	-6.73420	0.08680	7.69580	H	12.94340	5.48450	2.95820
C	-3.94550	7.10990	7.14240	H	13.16770	5.15500	1.25750
C	-4.41840	7.93930	8.18080	H	8.78550	7.49490	-5.57370
C	-5.72970	7.83890	8.62840	H	10.48620	8.50380	-4.08210
C	-6.61610	6.91240	8.05350	H	8.34010	7.07720	-0.65320
C	-6.15330	6.08510	7.02260	H	6.62460	6.07350	-2.14350
C	-4.83580	6.18210	6.57290	H	5.48540	9.98690	5.83180
O	-10.86240	7.78030	6.71250	H	7.85030	10.44380	5.25810
O	-12.46470	2.07510	7.94630	H	7.84230	7.12660	2.53900
O	-8.27010	4.84590	11.33820	H	5.46590	6.67180	3.09900
B	6.53780	6.13180	-4.91710	H	12.29760	0.82570	3.45670
O	5.34700	5.44120	-4.56720	H	13.36220	3.04850	3.21350
O	6.62170	6.22760	-6.33520	H	9.85860	4.52180	1.22610
C	4.69840	5.12550	-5.77700	H	8.78760	2.29190	1.45980
C	5.47240	5.60200	-6.84490	H	12.06920	5.05740	-0.54220
C	5.09840	5.45270	-8.17200	H	11.44980	6.22490	-1.69000
C	3.49150	4.46480	-5.95870	H	8.33060	10.07470	1.52050
C	3.89140	4.79430	-8.35310	H	8.63810	9.86420	-0.18940
C	3.11530	4.31800	-7.28660	H	11.05930	5.57020	4.15360
O	1.95220	3.71390	-7.80310	H	10.04090	6.88620	4.70030
B	2.02800	3.83740	-9.21600	H	-12.34730	3.82090	5.86620
O	3.23870	4.50230	-9.56140	H	-12.05580	5.53510	5.69920
B	-9.62150	3.55320	0.22700	H	-9.04120	2.37770	10.56790
O	-8.43060	2.89930	-0.18760	H	-10.43070	1.70240	9.75280
O	-10.36670	3.93780	-0.92370	H	-8.04920	6.98360	9.64150
C	-8.45430	2.88170	-1.59600	H	-8.57730	7.75870	8.16780
C	-9.62750	3.51000	-2.03810	H	-11.87660	4.82520	1.02950
C	-9.94780	3.64900	-3.38020	H	-12.67960	5.26730	3.33000
C	-7.51320	2.34790	-2.46550	H	-9.19910	3.33290	4.92660
C	-9.00830	3.11400	-4.24870	H	-8.39140	2.88290	2.62170
C	-7.83340	2.48740	-3.80880	H	-8.07750	-2.38760	9.60050
O	-7.09950	2.05470	-4.93090	H	-9.51610	-0.50580	10.32420
B	-7.85580	2.42610	-6.07420	H	-7.40640	2.13610	7.68340
O	-9.04340	3.08880	-5.65190	H	-5.95620	0.25420	6.95900
B	-2.49710	7.25250	6.68480	H	-3.74220	8.65850	8.62880
O	-1.81820	6.48140	5.70410	H	-6.07800	8.48190	9.43090

H	-6.83360	5.36630	6.58250
H	-4.48920	5.53510	5.77460
H	-8.92850	7.39250	6.13800
H	-10.16320	6.57400	5.20610
H	-10.64490	1.19950	7.57830
H	-11.37290	2.00520	6.20670
H	-7.27710	3.43530	10.22310
H	-6.86110	5.09670	9.86570
H	5.69300	5.82160	-8.99310
H	2.89400	4.10110	-5.13730
H	-10.85160	4.13050	-3.71970
H	-6.61070	1.86400	-2.12580
H	0.89990	9.55810	7.43580
H	0.44730	5.74520	4.13130
C	8.63440	-13.53720	-1.89790
C	8.45470	-14.72860	-1.17590
C	7.47920	-14.79860	-0.16800
C	6.68330	-13.67730	0.11800
C	6.86290	-12.48590	-0.60380
C	7.83850	-12.41590	-1.61190
H	9.40080	-13.48220	-2.68970
H	9.08010	-15.60950	-1.40070
H	7.33810	-15.73460	0.39920
H	6.23760	-11.60510	-0.37910
H	7.97960	-11.47990	-2.17900
C	-4.00480	-15.39220	1.73840
C	-3.17570	-16.43180	2.19090
C	-1.84180	-16.16470	2.54020
C	-1.33700	-14.85810	2.43690
C	-2.16600	-13.81850	1.98430
C	-3.50000	-14.08560	1.63510
H	-5.05280	-15.60200	1.46400
H	-3.57250	-17.45820	2.27210
H	-1.19050	-16.98140	2.89570
H	-1.76930	-12.79220	1.90320
H	-4.15130	-13.26890	1.27960
C	4.88740	-11.05370	10.62370
C	5.01760	-12.43130	10.38280
C	4.72860	-12.95220	9.11090
C	4.30930	-12.09550	8.07990
C	4.17910	-10.71790	8.32070
C	4.46820	-10.19700	9.59270
H	5.11450	-10.64450	11.62290
H	5.34700	-13.10430	11.19290
H	4.83090	-14.03450	8.92160
H	3.84980	-10.04490	7.51070
H	4.36590	-9.11470	9.78190
C	-11.10330	11.09310	4.18630
C	-11.92470	11.01550	5.32300
C	-11.84360	9.90050	6.17330
C	-10.94110	8.86320	5.88680
C	-10.11970	8.94070	4.75010
C	-10.20080	10.05570	3.89980
H	-11.16700	11.96900	3.51840
H	-12.63370	11.83060	5.54800
H	-12.48890	9.83960	7.06620
H	-9.41080	8.12570	4.52510
H	-9.55550	10.11670	3.00680
C	-14.53540	-1.41180	6.96290
C	-15.11400	-0.47310	7.83290
C	-14.41710	0.70050	8.16390
C	-13.14150	0.93530	7.62490
C	-12.56290	-0.00330	6.75490
C	-13.25990	-1.17690	6.42390
H	-15.08300	-2.33380	6.70280
H	-16.11620	-0.65780	8.25630
H	-14.87160	1.43790	8.84730
H	-11.56090	0.18130	6.33150
H	-12.80530	-1.91440	5.74040
C	-5.36810	4.73620	14.33500
C	-6.67880	5.13780	14.64090
C	-7.65550	5.17480	13.63230
C	-7.32160	4.81000	12.31770
C	-6.01090	4.40840	12.01170
C	-5.03420	4.37140	13.02040
H	-4.60080	4.70710	15.12730

H	-6.94110	5.42430	15.67380
H	-8.68520	5.49030	13.87270
H	-5.74870	4.12190	10.97900
H	-4.00450	4.05590	12.78000
C	15.00870	4.33870	-4.16040
C	15.76430	5.35220	-3.54820
C	15.21150	6.10330	-2.49800
C	13.90310	5.84080	-2.06000
C	13.14740	4.82740	-2.67210
C	13.70030	4.07630	-3.72240
H	15.44300	3.74870	-4.98540
H	16.79230	5.55830	-3.89240
H	15.80520	6.89940	-2.01710
H	12.11960	4.62140	-2.32790
H	13.10670	3.28020	-4.20330
C	12.17120	5.71340	8.74350
C	13.14340	6.63410	8.31930
C	13.10660	7.14100	7.00990
C	12.09760	6.72720	6.12490
C	11.12540	5.80650	6.54900
C	11.16220	5.29960	7.85850
H	12.20020	5.31520	9.77220
H	13.93610	6.95920	9.01470
H	13.87040	7.86430	6.67670
H	10.33280	5.48150	5.85370
H	10.39850	4.57630	8.19170
C	7.39910	14.41380	-0.10090
C	8.76960	14.62350	0.12430
C	9.60780	13.53510	0.41670
C	9.07560	12.23710	0.48390
C	7.70520	12.02730	0.25870
C	6.86690	13.11580	-0.03370
H	6.74060	15.26880	-0.33060
H	9.18760	15.64330	0.07160
H	10.68440	13.69990	0.59370
H	7.28710	11.00760	0.31150
H	5.79030	12.95100	-0.21070
C	2.42940	-2.17360	-15.78070
C	1.49430	-1.91580	-16.79660
C	0.21820	-1.42890	-16.46950
C	-0.12290	-1.19970	-15.12640
C	0.81210	-1.45750	-14.11040
C	2.08830	-1.94450	-14.43760
H	3.43190	-2.55610	-16.03770
H	1.76240	-2.09580	-17.85180
H	-0.51630	-1.22630	-17.26760
H	0.54400	-1.27750	-13.05540
H	2.82280	-2.14700	-13.63950
C	-10.02930	-4.43810	-11.75820
C	-9.96740	-4.02140	-13.09820
C	-8.96760	-3.12470	-13.50930
C	-8.02970	-2.64480	-12.58040
C	-8.09150	-3.06140	-11.24060
C	-9.09140	-3.95810	-10.82940
H	-10.81480	-5.14250	-11.43530
H	-10.70430	-4.39850	-13.82780
H	-8.91890	-2.79740	-14.56190
H	-7.35470	-2.68430	-10.51100
H	-9.14000	-4.28540	-9.77680
C	-5.53210	8.02780	-12.76720
C	-5.81750	7.48000	-14.02860
C	-5.63540	6.10590	-14.25500
C	-5.16790	5.27960	-13.21990
C	-4.88250	5.82730	-11.95840
C	-5.06460	7.20150	-11.73210
H	-5.67520	9.10740	-12.58930
H	-6.18470	8.12930	-14.84180
H	-5.85950	5.67550	-15.24600
H	-4.51530	5.17810	-11.14540
H	-4.84050	7.63190	-10.74110

Ta’ (optimized)

Sum of Electronic and Thermal Free Energies
(M06-2X-D3/3-21G: -11732.41408 Hartree

No imaginary frequencies found.

C	-16.98600	-6.94340	20.22650
C	-16.84380	-7.88590	19.19480
C	-17.50500	-7.68320	17.97250
C	-18.39360	-6.60590	17.82010
C	-18.53140	-5.66710	18.85560
C	-17.87310	-5.86460	20.08060
C	-16.21050	-7.11350	21.51340
C	-16.08790	-9.18340	19.44800
C	-17.29070	-8.64950	16.82940
C	-19.29980	-6.53260	16.59860
C	-19.42230	-4.46030	18.66500
C	-18.22290	-4.99610	21.28170
O	-17.05180	-7.93760	22.40570
O	-18.30620	-9.71180	16.98170
O	-20.77410	-4.86170	19.10680
C	-11.83670	-9.18640	18.49460
C	-12.46610	-10.32240	19.04390
C	-13.82290	-10.30780	19.34500
C	-14.59860	-9.15940	19.10810
C	-13.97750	-8.02750	18.56310
C	-12.61660	-8.04010	18.26040
C	-17.79880	-4.28410	13.18190
C	-19.05810	-4.91780	13.14720
C	-19.52500	-5.63240	14.24420
C	-18.75180	-5.73920	15.41350
C	-17.49950	-5.11110	15.45290
C	-17.02930	-4.39390	14.35360
C	-15.81290	-1.41540	21.87510
C	-15.54200	-2.28440	20.80350
C	-16.30740	-3.43250	20.60320
C	-17.36510	-3.74560	21.46780
C	-17.64050	-2.87940	22.54030
C	-16.87870	-1.73430	22.74160
B	-10.34830	-9.19890	18.16240
O	-9.46750	-10.29850	18.35570
B	-17.28290	-3.49910	11.98040
O	-16.03100	-2.82690	11.91260
B	-14.97440	-0.16030	22.09370
O	-13.88150	0.25140	21.28160
O	-9.61950	-8.11020	17.60820
O	-15.15850	0.78430	23.14060
O	-17.97400	-3.32160	10.75030
C	-8.29230	-8.55160	17.46470
C	-8.20020	-9.87560	17.91690
C	-7.01380	-10.59490	17.90430
C	-7.20480	-7.84870	16.96640
C	-17.13870	-2.53910	9.93350
C	-15.96320	-2.23990	10.63680
C	-14.93600	-1.47900	10.09740
C	-17.37410	-2.09950	8.63870
C	-14.17370	1.77230	22.96320
C	-13.40120	1.44980	21.83850
C	-12.34420	2.23460	21.40070
C	-13.94650	2.90370	23.73370
C	-5.92630	-9.89200	17.40590
C	-6.01840	-8.56790	16.95380
O	-4.75150	-8.14510	16.51420
B	-3.87080	-9.24550	16.70640
O	-4.59910	-10.33350	17.26140
C	-15.17140	-1.03950	8.80270
C	-16.34690	-1.33860	8.09940
O	-16.27890	-0.75120	6.82360
B	-15.02750	-0.07920	6.75580
O	-14.33590	-0.25700	7.98620
C	-12.11690	3.36600	22.17090
C	-12.88940	3.68860	23.29570
O	-12.40890	4.88710	23.85250
B	-11.31620	5.29860	23.04070
O	-11.13210	4.35380	21.99340
H	-16.58390	-9.97490	18.87290
H	-16.21970	-9.44310	20.50540
H	-20.25580	-6.10130	16.91960
H	-19.51660	-7.56080	16.28410
H	-19.27690	-4.70810	21.18730
H	-18.15510	-5.62500	22.17780
H	-16.00910	-6.14200	21.97490
H	-15.25580	-7.61420	21.32570
H	-17.41490	-8.14350	15.86720
H	-16.28530	-9.07950	16.86910
H	-19.06420	-3.61720	19.26350
H	-19.44300	-4.15730	17.61390
H	-11.87720	-11.21350	19.23050
H	-14.29380	-11.18960	19.76750
H	-14.56950	-7.13880	18.37980
H	-12.14650	-7.15850	17.83860
H	-19.66270	-4.84180	12.25040
H	-20.49610	-6.11510	14.20290
H	-16.89880	-5.18980	16.35110
H	-16.05880	-3.91200	14.39530
H	-14.72570	-2.05230	20.12850
H	-16.09100	-4.09550	19.77410
H	-18.45770	-3.11210	23.21530
H	-17.09950	-1.07320	23.57220
H	-6.94300	-11.61380	18.25200
H	-7.27570	-6.82990	16.61840
H	-14.03150	-1.24880	10.63850
H	-18.27870	-2.32960	8.09750
H	-11.74980	1.98640	20.53520
H	-14.54090	3.15190	24.59900
C	3.86990	-5.82880	13.81660
C	3.79680	-6.02320	15.20570
C	3.04190	-7.09040	15.71910
C	2.45740	-8.02550	14.84900
C	2.53410	-7.82540	13.46100
C	3.28790	-6.76020	12.94130
C	4.61160	-4.63360	13.26140
C	4.63950	-5.16570	16.14050
C	2.89140	-7.25680	17.21460
C	1.86450	-9.31400	15.40340
C	1.83590	-8.78250	12.52130
C	3.58410	-6.69350	11.44900
C	2.76800	-1.55320	17.69910
C	4.09250	-1.89820	18.03860
C	4.67790	-3.05370	17.53420
C	3.96190	-3.90480	16.67420
C	2.64510	-3.56630	16.33430
C	2.05610	-2.40770	16.83870
C	-2.38360	-9.25460	16.36870
C	-1.59560	-10.40160	16.59630
C	-0.24080	-10.40680	16.28550
C	0.37440	-9.26790	15.73680
C	-0.40430	-8.12480	15.51000
C	-1.76340	-8.11780	15.82080
C	0.79600	-4.40990	9.00090
C	1.90510	-5.06750	8.43000
C	2.78670	-5.79320	9.22250
C	2.59220	-5.88790	10.61160
C	1.49120	-5.23630	11.18380
C	0.60570	-4.50770	10.39070
O	4.02570	-8.09250	17.66010
O	2.80120	-9.86130	12.22570
C	-5.67620	9.96090	23.13740
C	-4.60890	10.40550	22.33950
C	-4.86770	11.21510	21.22150
C	-6.17110	11.67270	20.96680
C	-7.23370	11.22400	21.76760
C	-6.98170	10.41410	22.88740
C	-5.41200	9.02230	24.29320
C	-3.17320	10.12450	22.76210
C	-3.73330	11.62760	20.31070
C	-6.40780	12.75130	19.91810
C	-8.65010	11.64570	21.44750
C	-8.09060	10.14250	23.89510

C	-1.34390	6.49970	21.18170
C	-0.63920	7.35850	22.05020
C	-1.23470	8.51170	22.54760
C	-2.55350	8.84810	22.19530
C	-3.25800	7.99790	21.33220
C	-2.66270	6.84050	20.83240
C	-7.32480	11.38980	15.88240
C	-7.22990	12.76280	16.18960
C	-6.93600	13.18360	17.48130
C	-6.72690	12.24930	18.51090
C	-6.82000	10.88350	18.21050
C	-7.11470	10.45930	16.91540
C	-10.47500	6.55240	23.25580
C	-10.74090	7.42830	24.32850
C	-9.97290	8.57070	24.52090
C	-8.91320	8.87860	23.64980
C	-8.64500	8.01130	22.58210
C	-9.41410	6.86480	22.38750
O	-5.13520	9.87240	25.46960
O	-8.88940	12.90550	22.18190
C	-9.20360	4.91640	2.16770
C	-9.30500	3.58820	1.72260
C	-10.53340	2.91450	1.82100
C	-11.68130	3.59780	2.25420
C	-11.57340	4.92630	2.69760
C	-10.34790	5.60550	2.60190
C	-7.86520	5.61970	2.14250
C	-8.12890	2.92910	1.01450
C	-10.62890	1.45930	1.42090
C	-13.05240	2.94940	2.11650
C	-12.79030	5.63940	3.24300
C	-10.28830	7.10900	2.83680
C	-5.37110	0.65580	3.50610
C	-5.18720	0.75640	2.11160
C	-6.07490	1.48430	1.32800
C	-7.17650	2.13710	1.90880
C	-7.36370	2.03990	3.29430
C	-6.47440	1.31020	4.08200
C	-14.50780	0.70750	5.55690
C	-15.27550	0.82500	4.37990
C	-14.79930	1.54310	3.28920
C	-13.54110	2.16850	3.33520
C	-12.77430	2.05440	4.50280
C	-13.25010	1.33470	5.59800
C	-9.36600	8.41840	6.88840
C	-9.57950	9.34640	5.84840
C	-9.87240	8.91530	4.55980
C	-9.96220	7.54380	4.26460
C	-9.75030	6.61750	5.29480
C	-9.45660	7.04770	6.58800
O	-7.75140	6.25910	0.81530
O	-10.95240	1.44090	-0.02080
O	-13.45530	6.27820	2.08850
B	-0.69450	5.23290	20.63460
O	-1.31740	4.30110	19.75870
O	0.62810	4.79350	20.91770
C	-0.36900	3.29330	19.51010
C	0.80810	3.59120	20.21110
C	1.93490	2.78260	20.16900
C	-0.50680	2.16460	18.71510
C	1.79710	1.65390	19.37390
C	0.61980	1.35590	18.67300
O	0.79950	0.15350	17.96660
B	2.12280	-0.28560	18.24920
O	2.74550	0.64610	19.12470
B	-4.39910	-0.14210	4.36910
O	-4.49410	-0.30610	5.77900
O	-3.25460	-0.84030	3.89520
C	-3.40300	-1.10670	6.16090
C	-2.65300	-1.42990	5.02120
C	-1.50850	-2.21260	5.06980
C	-3.06420	-1.54220	7.43380
C	-1.16970	-2.64820	6.34290
C	-1.91970	-2.32500	7.48260
O	-1.31850	-2.91450	8.60870
B	-0.17350	-3.61290	8.13450

O	-0.07860	-3.44880	6.72500
B	-9.04480	8.88800	8.30340
O	-8.81370	8.03800	9.42050
O	-8.92850	10.24340	8.71770
C	-8.55640	8.88150	10.51570
C	-8.62580	10.21570	10.09060
C	-8.41760	11.28980	10.94390
C	-8.27380	8.52220	11.82580
C	-8.13490	10.93060	12.25390
C	-8.06560	9.59630	12.67910
O	-7.76300	9.56830	14.05190
B	-7.64650	10.92420	14.46610
O	-7.87740	11.77380	13.34930
O	-3.19140	12.88850	20.85830
O	6.01630	-5.05610	13.08400
H	4.95310	-5.79850	16.97980
H	5.55570	-4.89150	15.60340
H	2.05330	-10.10890	14.67150
H	2.43430	-9.58190	16.30150
H	3.63090	-7.72310	11.07380
H	4.59140	-6.27670	11.32850
H	4.65380	-1.24890	18.70110
H	5.69820	-3.30620	17.80390
H	2.08810	-4.21810	15.67170
H	1.03660	-2.15550	16.56870
H	-2.06000	-11.28560	17.01880
H	0.35310	-11.29690	16.46610
H	0.06430	-7.24340	15.08930
H	-2.35640	-7.22780	15.64050
H	2.06440	-5.00110	7.35960
H	3.63600	-6.29420	8.76920
H	1.33560	-5.30610	12.25370
H	-0.24290	-4.00740	10.84420
H	4.18550	-4.32580	12.30170
H	4.55580	-3.78830	13.95380
H	1.94330	-7.74720	17.45540
H	2.91810	-6.28500	17.71670
H	1.54410	-8.27310	11.59790
H	0.93740	-9.19730	12.98830
H	-2.56530	10.98970	22.47020
H	-3.15080	10.08870	23.85800
H	-7.22580	13.38880	20.27550
H	-5.51410	13.38620	19.88780
H	-8.75310	11.01660	23.90270
H	-7.63190	10.09580	24.89040
H	0.37860	7.10860	22.32840
H	-0.68020	9.16310	23.21540
H	-4.27470	8.25160	21.05710
H	-3.21730	6.18980	20.16540
H	-7.38920	13.49200	15.40320
H	-6.86570	14.24360	17.70310
H	-6.65920	10.15850	18.99930
H	-7.18410	9.40000	16.69420
H	-11.55610	7.20060	25.00630
H	-10.18930	9.23580	25.35070
H	-7.83030	8.24350	21.90680
H	-9.19770	6.20080	21.55790
H	-6.28260	8.38700	24.48260
H	-4.55220	8.37990	24.08080
H	-4.09500	11.78370	19.28990
H	-2.95440	10.85920	20.29050
H	-9.36290	10.88020	21.76870
H	-8.77290	11.80600	20.37220
H	-8.53580	2.27120	0.23690
H	-7.57270	3.71630	0.49080
H	-13.77140	3.74160	1.87450
H	-13.02020	2.28650	1.24320
H	-11.25460	7.53130	2.53500
H	-9.54310	7.52320	2.14680
H	-4.34100	0.25680	1.65350
H	-5.92090	1.55290	0.25610
H	-8.21110	2.54010	3.74760
H	-6.62890	1.24180	5.15320
H	-16.24740	0.34660	4.33370
H	-15.40130	1.62520	2.38990
H	-11.80390	2.53440	4.54430

H	-12.64820	1.25310	6.49630
H	-9.51250	10.40670	6.06520
H	-10.03420	9.64100	3.76930
H	-9.81750	5.55910	5.07330
H	-9.29460	6.32210	7.37750
H	-7.81070	6.37570	2.93170
H	-7.04980	4.90480	2.28860
H	-11.41330	0.95230	1.99060
H	-9.67990	0.94540	1.60150
H	-13.46970	4.93060	3.72570
H	-12.49830	6.39650	3.97710
H	2.84070	3.01180	20.70830
H	-1.41260	1.93530	18.17580
H	-0.93140	-2.46140	4.19290
H	-3.64130	-1.29350	8.31080
H	-8.47100	12.31650	10.61680
H	-8.22040	7.49550	12.15290
C	-4.35460	8.48390	-0.24280
C	-5.43000	8.31200	-1.12320
C	-6.54020	7.56870	-0.74030
C	-6.59250	6.98360	0.53320
C	-5.52240	7.15200	1.41560
C	-4.40920	7.90150	1.02200
H	-3.49000	9.06340	-0.54240
H	-5.40110	8.75930	-2.11010
H	-7.38310	7.42170	-1.40250
H	-5.54190	6.70960	2.40230
H	-3.58420	8.02600	1.71410
C	-11.42960	-2.17470	-2.09590
C	-11.56590	-0.93400	-2.73080
C	-11.40110	0.24470	-2.01320
C	-11.09660	0.20100	-0.64490
C	-10.95950	-1.03410	-0.00650
C	-11.12700	-2.21530	-0.73630
H	-11.55830	-3.09280	-2.65610
H	-11.80110	-0.88750	-3.78790
H	-11.50140	1.21510	-2.48120
H	-10.72510	-1.09080	1.04780
H	-11.01860	-3.16840	-0.23140
C	-16.96710	8.52340	2.57100
C	-16.37010	8.34450	1.31690
C	-15.20650	7.59450	1.19290
C	-14.62010	7.00970	2.32460
C	-15.21160	7.18480	3.57820
C	-16.38230	7.94110	3.69390
H	-17.87380	9.10810	2.66640
H	-16.81400	8.79150	0.43500
H	-14.72660	7.44210	0.23510
H	-14.77630	6.74260	4.46400
H	-16.83330	8.07100	4.67100
C	-15.75760	-8.98630	26.25710
C	-16.99850	-9.39850	25.75600
C	-17.39630	-9.03310	24.47540
C	-16.55640	-8.24710	23.67320
C	-15.31720	-7.83300	24.16810
C	-14.92500	-8.20530	25.45790
H	-15.44870	-9.27170	27.25520
H	-17.65640	-10.00630	26.36670
H	-18.35000	-9.33920	24.06620
H	-14.65400	-7.22630	23.56660
H	-13.96160	-7.87870	25.83230
C	-18.53830	-12.90400	14.26820
C	-19.41250	-12.79330	15.35660
C	-19.30780	-11.72260	16.23640
C	-18.32400	-10.74240	16.04090
C	-17.44920	-10.84780	14.95660
C	-17.56170	-11.92870	14.07630
H	-18.62080	-13.73890	13.58330
H	-20.17690	-13.54460	15.51820
H	-19.97130	-11.61520	17.08440
H	-16.68330	-10.10320	14.78710
H	-16.87800	-12.00110	13.23820
C	-23.99420	-2.17520	18.96510
C	-24.14560	-3.48740	19.43040
C	-23.05750	-4.35120	19.46390
C	-21.79670	-3.91510	19.03140
C	-21.64000	-2.60700	18.56640
C	-22.74050	-1.74440	18.53600
H	-24.84290	-1.50280	18.93940
H	-25.11500	-3.83590	19.76740
H	-23.14930	-5.36930	19.81890
H	-20.67680	-2.24980	18.22810
H	-22.60820	-0.73150	18.17330
C	-4.27860	8.18790	29.21610
C	-4.31640	9.57400	29.01950
C	-4.60330	10.09850	27.76490
C	-4.85800	9.24250	26.68360
C	-4.82150	7.85890	26.87410
C	-4.53190	7.33960	28.14000
H	-4.05500	7.78030	30.19430
H	-4.12130	10.24570	29.84750
H	-4.63830	11.16550	27.58810
H	-5.01450	7.18060	26.05420
H	-4.50620	6.26460	28.27710
C	-12.57440	14.89760	21.93920
C	-11.51660	15.39070	22.71320
C	-10.30900	14.70530	22.77120
C	-10.13970	13.51230	22.05380
C	-11.19170	13.01570	21.28010
C	-12.40350	13.71190	21.22750
H	-13.51510	15.43270	21.89470
H	-11.63550	16.31180	23.27200
H	-9.47720	15.06600	23.36180
H	-11.08270	12.09750	20.71910
H	-13.21340	13.31810	20.62410
C	0.01980	14.85830	19.00990
C	-0.58690	15.35890	20.16860
C	-1.64880	14.68080	20.75520
C	-2.12230	13.48760	20.19060
C	-1.52080	12.98360	19.03460
C	-0.45270	13.67250	18.45090
H	0.84730	15.38750	18.55360
H	-0.22980	16.28010	20.61430
H	-2.13370	15.04730	21.65040
H	-1.87000	12.06540	18.58240
H	0.00700	13.27290	17.55410
C	4.50270	-9.13740	21.69590
C	5.40450	-9.55930	20.71100
C	5.21650	-9.19470	19.38310
C	4.12020	-8.40000	19.01810
C	3.21740	-7.97640	19.99640
C	3.41410	-8.34790	21.33030
H	4.65020	-9.42200	22.73040
H	6.25560	-10.17370	20.98110
H	5.89930	-9.50820	18.60430
H	2.36510	-7.36310	19.73690
H	2.70780	-8.01390	22.08180
C	1.79720	-13.03620	9.67240
C	3.05450	-12.94720	10.28270
C	3.35400	-11.88170	11.12330
C	2.39790	-10.88530	11.36750
C	1.14190	-10.96910	10.76130
C	0.84900	-12.04470	9.91680
H	1.56490	-13.86700	9.01760
H	3.80170	-13.71120	10.10160
H	4.31810	-11.79090	11.60620
H	0.38990	-10.21180	10.93570
H	-0.12850	-12.10050	9.45140
C	8.90930	-2.41590	11.57830
C	9.22440	-3.73250	11.93660
C	8.24130	-4.58060	12.43240
C	6.92330	-4.12390	12.57810
C	6.60340	-2.81130	12.22250
C	7.59900	-1.96470	11.72430
H	9.67630	-1.75590	11.19200
H	10.23940	-4.09700	11.82840
H	8.46000	-5.60170	12.71580
H	5.59380	-2.43820	12.32720
H	7.34030	-0.94790	11.45130

Tb

Sum of Electronic and Thermal Free Energies
(M06-2X-D3/3-21G: -8544.102161 Hartree

No imaginary frequencies found.

C	3.99120	-12.50370	2.27750
C	2.67570	-12.90320	1.96580
C	1.66580	-12.85410	2.94900
C	1.98630	-12.45180	4.26100
C	3.29940	-12.04740	4.57860
C	4.30480	-12.09780	3.59180
C	5.07220	-12.50520	1.19660
C	2.34390	-13.41900	0.56980
C	0.22840	-13.22750	2.58940
C	0.91610	-12.49110	5.34650
C	3.63160	-11.55520	5.98670
C	5.74110	-11.73770	3.95660
C	5.80270	-13.87020	1.08690
C	-0.06880	-14.73740	2.79490
C	4.06870	-12.70690	6.93120
C	0.88160	-10.53070	-2.34840
C	0.91240	-11.91380	-2.62760
C	1.37090	-12.81930	-1.68000
C	1.81780	-12.37910	-0.42180
C	1.78690	-11.00890	-0.13690
C	1.32480	-10.09640	-1.08730
C	-1.44810	-8.88990	6.00860
C	-1.71630	-10.08940	6.70090
C	-0.95460	-11.22710	6.47010
C	0.10030	-11.21070	5.54080
C	0.36730	-10.02550	4.84480
C	-0.39560	-8.87950	5.07700
C	6.96410	-7.57700	3.51250
C	5.69760	-7.99040	3.06330
C	5.28310	-9.31530	3.21020
C	6.12530	-10.26310	3.80510
C	7.38980	-9.85240	4.26170
C	7.80330	-8.53430	4.11940
B	0.37300	-9.57410	-3.42300
O	-0.07060	-10.00670	-4.70540
B	-2.29560	-7.65630	6.30270
O	-2.15330	-6.35950	5.74080
B	7.46020	-6.14360	3.35170
O	6.77170	-5.06140	2.74100
O	0.26250	-8.16040	-3.34640
O	8.73480	-5.69340	3.79730
O	-3.37550	-7.64620	7.23040
C	-0.24930	-7.73150	-4.58760
C	-0.44920	-8.85170	-5.40710
C	-0.94410	-8.76190	-6.69930
C	-0.53170	-6.43690	-5.00180
C	-3.88540	-6.33840	7.23400
C	-3.14700	-5.55610	6.33430
C	-3.40750	-4.20970	6.11950
C	-4.93870	-5.83520	7.98250
C	8.82140	-4.33570	3.44980
C	7.63490	-3.95000	2.80930
C	7.41890	-2.66210	2.33840
C	9.87800	-3.46460	3.66750
C	-1.22320	-7.46850	-7.11390
C	-1.02540	-6.34650	-6.29620
O	-1.39860	-5.19060	-7.01070
B	-1.82870	-5.63020	-8.29130
O	-1.72280	-7.04840	-8.35650
C	-4.46020	-3.70510	6.87050
C	-5.19870	-4.49020	7.76760
O	-6.18530	-3.69360	8.37000
B	-6.03690	-2.38640	7.82540
O	-4.96800	-2.39090	6.89030
C	8.47730	-1.79020	2.55470
C	9.66240	-2.17850	3.19610
O	10.53230	-1.07870	3.25780
B	9.85630	0.00700	2.63160
O	8.57720	-0.43100	2.19650
H	1.60180	-14.22000	0.64750
H	3.23640	-13.87900	0.13250
H	1.38290	-12.74150	6.30520
H	0.21750	-13.30660	5.13370
H	5.93810	-12.03460	4.99120
H	6.42910	-12.32790	3.34190
H	5.79950	-11.71530	1.40750
H	4.62260	-12.25370	0.23100
H	-0.46210	-12.63310	3.19560
H	0.03500	-12.95540	1.54750
H	4.42640	-10.80530	5.92970
H	2.76010	-11.04650	6.40940
H	0.57150	-12.26200	-3.59600
H	1.38930	-13.88040	-1.90960
H	2.12380	-10.66780	0.83460
H	1.30710	-9.03750	-0.85370
H	-2.52660	-10.11150	7.42090
H	-1.17130	-12.14240	7.01240
H	1.17600	-10.00920	4.12470
H	-0.17610	-7.96630	4.53470
H	5.03870	-7.26680	2.59590
H	4.30540	-9.62590	2.86290
H	8.04850	-10.57840	4.72830
H	8.78130	-8.22790	4.47290
H	-1.09590	-9.62450	-7.32910
H	-0.37740	-5.57400	-4.37280
H	-2.83850	-3.60610	5.42960
H	-5.50560	-6.43800	8.67470
H	6.50720	-2.36490	1.84400
H	10.79090	-3.76250	4.15910
H	6.56860	-13.83630	0.30360
H	4.29640	-12.32080	7.93130
H	-1.10700	-14.96530	2.52770
H	5.09470	-14.66990	0.84590
H	6.28690	-14.12810	2.03440
H	0.09170	-15.02710	3.83850
H	0.59110	-15.35170	2.17350
H	4.95970	-13.21100	6.54280
H	3.27530	-13.45590	7.02050
C	-4.29560	1.83000	-12.42990
C	-3.05010	1.33850	-12.87020
C	-2.83190	-0.05130	-12.97640
C	-3.87920	-0.94800	-12.68690
C	-5.12860	-0.46250	-12.24680
C	-5.34170	0.92690	-12.14580
C	-4.51870	3.33240	-12.26090
C	-1.94270	2.30660	-13.27440
C	-1.46540	-0.57930	-13.41020
C	-3.67350	-2.44580	-12.88220
C	-6.24500	-1.43890	-11.87800
C	-6.71780	1.46110	-11.76420
C	0.99410	3.38000	-10.24000
C	1.22500	3.62760	-11.60940
C	0.28780	3.25700	-12.56550
C	-0.91200	2.62770	-12.19010
C	-1.14040	2.36560	-10.83370
C	-0.20110	2.73820	-9.87130
C	-2.31810	-4.76610	-9.44920
C	-2.68530	-5.38490	-10.66250
C	-3.12240	-4.62810	-11.74170
C	-3.20950	-3.22810	-11.65050
C	-2.85230	-2.60760	-10.44720
C	-2.41100	-3.36610	-9.36120
C	-7.50930	2.17080	-7.53770
C	-8.41290	2.58510	-8.53920
C	-8.13800	2.35680	-9.88110
C	-6.95260	1.71010	-10.27290
C	-6.04920	1.30160	-9.28470
C	-6.32480	1.52670	-7.93470
C	-1.35180	-0.74170	-14.94960
C	-7.14080	-1.80780	-13.09090
C	11.38510	6.89910	0.35210
C	10.63390	8.05790	0.05990
C	9.98010	8.75930	1.09220

C	10.11390	8.32280	2.42800
C	10.85980	7.16540	2.72490
C	11.51660	6.47050	1.68710
C	12.05330	6.12490	-0.78320
C	10.56150	8.55700	-1.37910
C	9.13100	9.99160	0.77950
C	9.47430	9.12850	3.55410
C	10.96710	6.66020	4.16270
C	12.40140	5.27570	2.03020
C	7.57820	6.71540	-3.96640
C	8.68630	7.40760	-4.49760
C	9.63880	7.97330	-3.65880
C	9.51950	7.86810	-2.26240
C	8.42770	7.17180	-1.72880
C	7.46820	6.60600	-2.56890
C	5.30270	8.29590	4.50620
C	6.00100	9.36280	5.11090
C	7.32790	9.61580	4.78880
C	8.00790	8.81140	3.85760
C	7.32220	7.74880	3.25700
C	5.98660	7.49550	3.57510
C	10.46630	1.39600	2.47200
C	11.76620	1.63310	2.96570
C	12.36110	2.88070	2.82820
C	11.68200	3.93500	2.19350
C	10.38820	3.70880	1.70750
C	9.78880	2.45550	1.84320
C	13.49850	6.61510	-1.06590
C	12.17220	7.27580	4.92290
C	-9.75140	5.64050	7.09950
C	-10.54030	4.50190	6.82450
C	-10.40170	3.33690	7.60240
C	-9.49940	3.32520	8.68950
C	-8.70880	4.45560	8.96730
C	-8.85500	5.62270	8.18440
C	-9.89340	6.88230	6.21990
C	-11.55890	4.55940	5.68940
C	-11.21810	2.08390	7.28750
C	-9.42120	2.09030	9.58210
C	-7.69890	4.43960	10.11480
C	-8.06180	6.87280	8.55240
C	-10.07740	3.81760	1.65840
C	-11.29340	4.49550	1.88190
C	-11.74220	4.74240	3.17400
C	-10.99620	4.31910	4.28680
C	-9.78330	3.65060	4.07150
C	-9.33060	3.40270	2.77560
C	-6.90960	-1.20240	8.22990
C	-7.92900	-1.39760	9.18430
C	-8.73510	-0.34040	9.58860
C	-8.55060	0.94750	9.05790
C	-7.54430	1.14700	8.10350
C	-6.73420	0.08680	7.69580
C	-3.94550	7.10990	7.14240
C	-4.41840	7.93930	8.18080
C	-5.72970	7.83890	8.62840
C	-6.61610	6.91240	8.05350
C	-6.15330	6.08510	7.02260
C	-4.83580	6.18210	6.57290
C	-10.96000	7.87080	6.76210
C	-12.59040	2.07420	8.01280
C	-8.32770	4.88690	11.46160
B	6.53780	6.13180	-4.91710
O	5.34700	5.44120	-4.56720
O	6.62170	6.22760	-6.33520
C	4.69840	5.12550	-5.77700
C	5.47240	5.60200	-6.84490
C	5.09840	5.45270	-8.17200
C	3.49150	4.46480	-5.95870
C	3.89140	4.79430	-8.35310
C	3.11530	4.31800	-7.28660
O	1.95220	3.71390	-7.80310
B	2.02800	3.83740	-9.21600
O	3.23870	4.50230	-9.56140
B	-9.62150	3.55320	0.22700
O	-8.43060	2.89930	-0.18760
O	-10.36670	3.93780	-0.92370
C	-8.45430	2.88170	-1.59600
C	-9.62750	3.51000	-2.03810
C	-9.94780	3.64900	-3.38020
C	-7.51320	2.34790	-2.46550
C	-9.00830	3.11400	-4.24870
C	-7.83340	2.48740	-3.80880
O	-7.09950	2.05470	-4.93090
B	-7.85580	2.42610	-6.07420
O	-9.04340	3.08880	-5.65190
B	-2.49710	7.25250	6.68480
O	-1.81820	6.48140	5.70410
O	-1.60070	8.22280	7.21650
C	-0.50290	6.98360	5.64100
C	-0.37570	8.03800	6.55690
C	0.80360	8.74600	6.73220
C	0.54380	6.55710	4.83520
C	1.84820	8.32150	5.92520
C	1.72490	7.26480	5.01150
O	2.95850	7.08400	4.35460
B	3.84430	8.05860	4.88620
O	3.15520	8.82970	5.86490
C	9.96600	11.29980	0.76670
C	-5.03870	4.00690	-13.55870
H	-1.41260	1.91050	-14.14630
H	-2.38840	3.25220	-13.60110
H	-4.60550	-2.89560	-13.23880
H	-2.93960	-2.60810	-13.67860
H	-7.48590	0.76300	-12.11400
H	-6.90110	2.39890	-12.29820
H	2.14530	4.11670	-11.90790
H	0.47660	3.45930	-13.61530
H	-2.05780	1.86910	-10.54130
H	-0.39380	2.53780	-8.82300
H	-2.61960	-6.46400	-10.74390
H	-3.39900	-5.11900	-12.66980
H	-2.92070	-1.52930	-10.37180
H	-2.13250	-2.87290	-8.43630
H	-9.33000	3.08340	-8.24640
H	-8.84420	2.67830	-10.64070
H	-5.13410	0.80410	-9.58250
H	-5.61920	1.20030	-7.17860
H	-5.23100	3.50380	-11.44850
H	-3.58250	3.80740	-11.95110
H	-1.27670	-1.54220	-12.92520
H	-0.68610	0.10130	-13.05560
H	-6.86280	-1.00190	-11.08740
H	-5.80670	-2.34830	-11.45610
H	10.35160	9.63200	-1.38340
H	11.54170	8.44180	-1.85240
H	10.04780	8.98240	4.47500
H	9.54820	10.19630	3.32260
H	12.94340	5.48450	2.95820
H	13.16770	5.15500	1.25750
H	8.78550	7.49490	-5.57370
H	10.48620	8.50380	-4.08210
H	8.34010	7.07720	-0.65320
H	6.62460	6.07350	-2.14350
H	5.48540	9.98690	5.83180
H	7.85030	10.44380	5.25810
H	7.84230	7.12660	2.53900
H	5.46590	6.67180	3.09900
H	12.29760	0.82570	3.45670
H	13.36220	3.04850	3.21350
H	9.85860	4.52180	1.22610
H	8.78760	2.29190	1.45980
H	12.06920	5.05740	-0.54220
H	11.44980	6.22490	-1.69000
H	8.33060	10.07470	1.52050
H	8.63810	9.86420	-0.18940
H	11.05930	5.57020	4.15360
H	10.04090	6.88620	4.70030
H	-12.34730	3.82090	5.86620
H	-12.05580	5.53510	5.69920
H	-9.04120	2.37770	10.56790
H	-10.43070	1.70240	9.75280

H	-8.04920	6.98360	9.64150
H	-8.57730	7.75870	8.16780
H	-11.87660	4.82520	1.02950
H	-12.67960	5.26730	3.33000
H	-9.19910	3.33290	4.92660
H	-8.39140	2.88290	2.62170
H	-8.07750	-2.38760	9.60050
H	-9.51610	-0.50580	10.32420
H	-7.40640	2.13610	7.68340
H	-5.95620	0.25420	6.95900
H	-3.74220	8.65850	8.62880
H	-6.07800	8.48190	9.43090
H	-6.83360	5.36630	6.58250
H	-4.48920	5.53510	5.77460
H	-8.92850	7.39250	6.13800
H	-10.16320	6.57400	5.20610
H	-10.64490	1.19950	7.57830
H	-11.37290	2.00520	6.20670
H	-7.27710	3.43530	10.22310
H	-6.86110	5.09670	9.86570
H	5.69300	5.82160	-8.99310
H	2.89400	4.10110	-5.13730
H	-10.85160	4.13050	-3.71970
H	-6.61070	1.86400	-2.12580
H	0.89990	9.55810	7.43580
H	0.44730	5.74520	4.13130
H	12.22330	6.88500	5.94570
H	13.94560	6.04480	-1.88820
H	14.12910	6.49700	-0.17860
H	13.50150	7.67580	-1.33750
H	12.08520	8.36610	4.97250
H	13.11270	7.03980	4.41450
H	9.32620	12.16420	0.55570
H	10.45470	11.45680	1.73380
H	10.74830	11.25260	0.00190
H	-0.35590	-1.10630	-15.22660
H	-7.92990	-2.50610	-12.78940
H	-1.52420	0.21480	-15.45390
H	-2.09630	-1.45350	-15.32170
H	-6.54830	-2.27590	-13.88380
H	-7.61160	-0.91310	-13.51130
H	-5.18330	5.08220	-13.40380
H	-4.32730	3.86670	-14.37910
H	-5.99390	3.56940	-13.86740
H	-13.14390	1.15720	7.78060
H	-7.57750	4.87590	12.26070
H	-11.04820	8.74270	6.10380
H	-10.69300	8.21900	7.76530
H	-11.93920	7.38520	6.82750
H	-13.19740	2.93270	7.70730
H	-12.45460	2.13240	9.09770
H	-9.14850	4.22070	11.74690
H	-8.73380	5.90070	11.38150

Tb* (unoptimized)

Unoptimized structure.

C	2.97530	9.14410	-8.97280
C	4.03410	8.32720	-9.40030
C	5.25180	8.34380	-8.69930
C	5.44920	9.25300	-7.64790
C	4.38520	10.06710	-7.22420
C	3.16650	10.05600	-7.92090
C	1.63490	9.07160	-9.66920
C	3.91330	7.52770	-10.69070
C	6.36510	7.40540	-9.10730
C	6.83960	9.45580	-7.06060
C	4.56860	10.98550	-6.03710
C	2.10950	11.11290	-7.62990
C	1.67960	10.10040	-10.81420
C	7.18700	8.14190	-10.18140
C	5.11210	12.31560	-6.59120
C	2.49620	3.42450	-10.32070
C	2.91290	3.94740	-11.56220
C	3.36660	5.25720	-11.66590
C	3.41930	6.09040	-10.53480
C	3.00820	5.57460	-9.29820
C	2.55270	4.26120	-9.19200
C	7.85380	7.07110	-3.55830
C	8.73830	8.04640	-4.06290
C	8.40090	8.80260	-5.17930
C	7.16990	8.61130	-5.83100
C	6.28660	7.64430	-5.33210
C	6.62320	6.88450	-4.21260
C	-0.93370	10.07700	-4.68930
C	0.10330	9.17500	-4.98650
C	1.08230	9.49590	-5.92590
C	1.05340	10.72650	-6.59590
C	0.01860	11.63180	-6.30220
C	-0.95790	11.31420	-5.36550
B	1.99680	1.98830	-10.20290
O	1.89660	1.05750	-11.27330
B	8.22120	6.23910	-2.33410
O	7.40400	5.23370	-1.74650
B	-2.00510	9.72390	-3.66300
O	-3.09140	10.55850	-3.28170
O	1.55540	1.36650	-9.00200
O	-2.07710	8.50390	-2.93480
O	9.43350	6.34270	-1.59810
C	1.18580	0.05420	-9.34590
C	1.39220	-0.13280	-10.72010
C	1.11970	-1.32990	-11.36650
C	0.69180	-0.94200	-8.51620
C	9.35210	5.39670	-0.56100
C	8.12420	4.72570	-0.65090
C	7.74480	3.72770	0.23520
C	10.29160	5.11950	0.42160
C	-3.21150	8.59940	-2.10960
C	-3.82490	9.84270	-2.31910
C	-4.97280	10.23880	-1.64780
C	-3.70100	7.65960	-1.21390
C	0.62560	-2.32610	-10.53670
C	0.41930	-2.13910	-9.16250
O	-0.08560	-3.32910	-8.60920
B	-0.18720	-4.25960	-9.68040
O	0.25510	-3.63820	-10.88060
C	8.68450	3.45030	1.21770
C	9.91230	4.12130	1.30750
O	10.63290	3.61220	2.40250
B	9.81700	2.60610	2.98900
O	8.60340	2.50330	2.25380
C	-5.46200	9.29910	-0.75190
C	-4.84910	8.05550	-0.54300
O	-5.58310	7.33910	0.41890
B	-6.66890	8.17500	0.80120
O	-6.59590	9.39490	0.07420
H	4.89830	7.52760	-11.17280
H	3.24220	8.07680	-11.36260
H	6.94190	10.51940	-6.81390
H	7.57000	9.25260	-7.85350
H	2.62850	12.02260	-7.30410
H	1.62030	11.36160	-8.57950
H	0.82560	9.31540	-8.97420
H	1.45910	8.06830	-10.06910
H	6.99850	7.15880	-8.24990
H	5.95550	6.47670	-9.51610
H	3.61610	11.15110	-5.52460
H	5.27790	10.55590	-5.32320
H	2.87640	3.31340	-12.44100
H	3.68490	5.64590	-12.62780
H	3.05060	6.21040	-8.42190
H	2.23650	3.87270	-8.23020
H	9.69030	8.20220	-3.56800
H	9.09140	9.55030	-5.55620
H	5.33480	7.49480	-5.82760
H	5.93220	6.13860	-3.83550
H	0.13530	8.21960	-4.47440
H	1.87700	8.79380	-6.14830
H	-0.01350	12.58730	-6.81570
H	-1.75170	12.01960	-5.14690
H	1.27830	-1.47380	-12.42390
H	0.53280	-0.79800	-7.45890
H	6.80020	3.21120	0.16590
H	11.23650	5.63560	0.49060
H	-5.44470	11.19550	-1.80880
H	-3.22910	6.70290	-1.05300
C	-3.16700	-11.54880	-5.43690
C	-4.14990	-10.88410	-6.18730
C	-3.78790	-10.21050	-7.36620
C	-2.47370	-10.29530	-7.85260
C	-1.49430	-10.96090	-7.09630
C	-1.84980	-11.63670	-5.91830
C	-3.53340	-12.20630	-4.12530
C	-5.61850	-11.00210	-5.80210
C	-4.82890	-9.42430	-8.13100
C	-2.13940	-9.79120	-9.25030
C	-0.06040	-10.98150	-7.57590
C	-0.85290	-12.56770	-5.24100
C	-7.21360	-7.88320	-3.21180
C	-8.04270	-8.89600	-3.73640
C	-7.52180	-9.88340	-4.56460
C	-6.15590	-9.89450	-4.89740
C	-5.32820	-8.88880	-4.38010
C	-5.84860	-7.89780	-3.54890
C	-0.69080	-5.69410	-9.55920
C	-0.75320	-6.53710	-10.68780
C	-1.21360	-7.84370	-10.57470
C	-1.62840	-8.35380	-9.33200
C	-1.56580	-7.52060	-8.20680
C	-1.10460	-6.20960	-8.31810
C	1.61340	-10.83700	-2.09400
C	1.72090	-12.19850	-2.44510
C	0.92760	-12.73810	-3.45090
C	0.00040	-11.93790	-4.14120
C	-0.10960	-10.58400	-3.79640
C	0.68480	-10.04080	-2.78750
C	-3.95920	-13.64750	-4.46190
C	-5.48620	-10.40880	-9.11610
C	0.07890	-12.21510	-8.48720
C	-11.38080	4.36550	4.90970
C	-11.47920	3.17540	5.64970
C	-10.78900	3.05510	6.86640
C	-10.09740	4.15630	7.39860
C	-10.00450	5.34380	6.65510
C	-10.69260	5.47010	5.43650
C	-12.05360	4.46890	3.55940
C	-12.43610	2.07910	5.20060
C	-10.82170	1.74880	7.62770
C	-9.56890	4.10320	8.82580
C	-9.19370	6.50530	7.18460
C	-10.80380	6.83120	4.76270
C	-10.80090	-0.99280	2.57960
C	-12.16880	-0.97230	2.92140
C	-12.67850	0.01580	3.75560

C	-11.84090	1.01680	4.27800
C	-10.48100	1.00200	3.93940
C	-9.96770	0.01160	3.10300
C	-5.47980	2.68980	9.33950
C	-6.29300	3.00090	10.44870
C	-7.59730	3.44760	10.27130
C	-8.13480	3.59980	8.98120
C	-7.33120	3.28990	7.87560
C	-6.02260	2.84200	8.05130
C	-7.73830	7.81800	1.82820
C	-8.77150	8.72450	2.14310
C	-9.74320	8.39720	3.08150
C	-9.71800	7.15400	3.73750
C	-8.69380	6.24910	3.42710
C	-7.71770	6.57570	2.48660
C	-13.48260	4.97990	3.82070
C	-12.04110	1.81880	8.56560
C	-10.15230	7.35510	8.03940
C	8.50170	-3.47720	9.39960
C	9.39590	-3.88450	8.39680
C	10.20770	-2.93060	7.76010
C	10.20790	-1.59720	8.19980
C	9.31120	-1.19610	9.20440
C	8.49870	-2.14480	9.84560
C	7.56570	-4.48330	10.03060
C	9.58310	-5.36550	8.09620
C	11.11200	-3.35130	6.62350
C	11.26080	-0.62300	7.68850
C	9.25130	0.25620	9.62160
C	7.72360	-1.76610	11.10060
C	7.10480	-7.04640	4.93170
C	8.04540	-7.83610	5.62450
C	8.83050	-7.28720	6.63160
C	8.70380	-5.93170	6.98260
C	7.77020	-5.14270	6.29710
C	6.98200	-5.69120	5.28620
C	10.18240	1.76810	4.20970
C	11.41630	1.94660	4.86840
C	11.74680	1.17950	5.97950
C	10.85780	0.20770	6.47120
C	9.62910	0.02830	5.82140
C	9.29580	0.79630	4.70660
C	3.62550	-0.40040	10.53440
C	4.17680	-0.55220	11.82340
C	5.48470	-0.99200	11.99000
C	6.28780	-1.29500	10.87650
C	5.74380	-1.14770	9.59340
C	4.43210	-0.70640	9.42400
C	8.31740	-5.08870	11.23060
C	12.44760	-3.77730	7.26100
C	10.29120	0.43300	10.74360
B	-10.23840	-2.07660	1.66580
O	-8.87950	-2.18840	1.26020
O	-10.98660	-3.13710	1.08450
C	-8.80380	-3.32100	0.43040
C	-10.07850	-3.89510	0.32420
C	-10.33190	-5.03230	-0.42930
C	-7.68790	-3.84130	-0.20910
C	-9.21580	-5.55280	-1.06860
C	-7.94120	-4.97850	-0.96250
O	-7.03280	-5.73670	-1.72210
B	-7.78100	-6.79860	-2.30210
O	-9.13960	-6.68620	-1.89740
B	6.24220	-7.64490	3.82560
O	5.27140	-6.93630	3.06450
O	6.27830	-8.99810	3.39010
C	4.71730	-7.86350	2.16430
C	5.32630	-9.11100	2.36140
C	4.99300	-10.23690	1.62220
C	3.73040	-7.64920	1.21300
C	4.00570	-10.02270	0.67120
C	3.39720	-8.77510	0.47370
O	2.44520	-8.88780	-0.55490
B	2.48020	-10.24200	-0.98930
O	3.45050	-10.95010	-0.22820
B	2.19130	0.08350	10.34820
O	1.54130	0.27110	9.09680
O	1.29070	0.42050	11.39580
C	0.24220	0.72380	9.38710
C	0.09050	0.81430	10.77800
C	-1.08620	1.23430	11.38150
C	-0.77150	1.04660	8.49650
C	-2.09970	1.55750	10.49080
C	-1.94810	1.46700	9.09990
O	-3.14760	1.86200	8.48190
B	-4.04790	2.20070	9.53010
O	-3.39850	2.01180	10.78090
H	-6.20210	-11.03470	-6.73000
H	-5.75900	-11.97540	-5.31610
H	-1.39270	-10.47150	-9.67710
H	-3.04010	-9.89700	-9.86750
H	-0.20180	-12.98220	-6.02030
H	-1.41670	-13.41370	-4.82960
H	-9.09770	-8.89690	-3.48620
H	-8.17200	-10.65630	-4.96150
H	-4.27550	-8.88940	-4.63640
H	-5.19870	-7.12470	-3.15390
H	-0.43590	-6.15380	-11.65110
H	-1.25560	-8.48130	-11.45190
H	-1.87930	-7.90950	-7.24540
H	-1.06120	-5.57350	-7.44090
H	2.43250	-12.82470	-1.91890
H	1.02060	-13.78790	-3.70980
H	-0.82240	-9.96230	-4.32480
H	0.59100	-8.99190	-2.52870
H	-2.67650	-12.21440	-3.44490
H	-4.35680	-11.67270	-3.64080
H	-4.36660	-8.59600	-8.67650
H	-5.58030	-9.01500	-7.44880
H	0.62990	-11.05640	-6.73020
H	0.17200	-10.07050	-8.13550
H	-12.83990	1.60000	6.10100
H	-13.28490	2.56390	4.70340
H	-9.65350	5.11100	9.25070
H	-10.24710	3.46680	9.40690
H	-10.80210	7.59280	5.55200
H	-11.78950	6.88630	4.28490
H	-12.82360	-1.73990	2.52430
H	-13.73350	0.01910	4.00990
H	-9.83110	1.77310	4.33560
H	-8.91360	0.00950	2.84830
H	-5.88840	2.88750	11.44820
H	-8.21150	3.68320	11.13440
H	-7.74100	3.40170	6.87890
H	-5.40950	2.60590	7.18850
H	-8.80030	9.68630	1.64330
H	-10.53200	9.10520	3.31430
H	-8.66920	5.28920	3.92890
H	-6.92950	5.86800	2.25480
H	-11.51550	5.16680	2.91080
H	-12.08590	3.49150	3.06860
H	-9.90450	1.61830	8.20990
H	-10.92140	0.90240	6.94130
H	-8.79130	7.10280	6.36100
H	-8.35860	6.14850	7.79510
H	10.63950	-5.52210	7.84610
H	9.40180	-5.91910	9.02560
H	11.52590	0.04370	8.51780
H	12.16450	-1.20020	7.45700
H	8.29090	-0.98310	11.61880
H	7.72490	-2.63780	11.76610
H	8.15120	-8.88280	5.36200
H	9.55050	-7.90710	7.15600
H	7.66810	-4.09740	6.56280
H	6.26290	-5.07130	4.76230
H	12.10970	2.69300	4.49730
H	12.70020	1.32780	6.47620
H	8.93810	-0.71670	6.19760
H	8.34270	0.64820	4.21090
H	3.56620	-0.32110	12.68910
H	5.89560	-1.10440	12.98820
H	6.35670	-1.38300	8.73140

H	4.02180	-0.59540	8.42630
H	6.64590	-3.99580	10.36790
H	7.30160	-5.26810	9.31530
H	11.27280	-2.52130	5.92870
H	10.67380	-4.18760	6.07050
H	8.25290	0.51370	9.98770
H	9.49160	0.90990	8.77760
H	-11.31270	-5.47430	-0.51080
H	-6.70700	-3.39960	-0.12720
H	5.46130	-11.19690	1.77410
H	3.26190	-6.68930	1.06120
H	-1.20280	1.30420	12.45180
H	-0.65470	0.97730	7.42620
H	13.14600	-4.09470	6.47270
H	10.28850	1.47850	11.08540
H	7.67680	-5.83170	11.72820
H	8.01510	7.49930	-10.51490
H	0.72260	10.08820	-11.35650
H	5.26410	13.02360	-5.76320
H	1.10990	-12.27760	-8.86550
H	-6.25600	-9.88400	-9.70090
H	-4.23780	-14.17360	-3.53690
H	-9.60950	8.21870	8.45110
H	-14.01950	5.07380	2.86520
H	-12.11170	0.88730	9.14630
H	-13.43650	5.95360	4.30840
H	-14.00830	4.27490	4.46470
H	-12.94740	1.94730	7.97380
H	-11.92800	2.66310	9.24580
H	-10.54400	6.74920	8.85640
H	-10.97690	7.70660	7.41930
H	2.48840	9.84620	-11.49940
H	1.85080	11.09490	-10.40230
H	7.58920	9.06400	-9.76190
H	6.54580	8.37790	-11.03070
H	4.39650	12.73340	-7.29930
H	6.06170	12.13770	-7.09580
H	-3.13010	-14.16870	-4.94040
H	-4.81370	-13.62500	-5.13820
H	-5.94650	-11.22560	-8.56020
H	-4.72830	-10.80870	-9.78970
H	-0.61100	-12.12640	-9.32640
H	-0.15420	-13.11510	-7.91810
H	8.57170	-4.29790	11.93630
H	9.22980	-5.57190	10.88130
H	12.27420	-4.60590	7.94760
H	12.87410	-2.93570	7.80680
H	11.28080	0.17730	10.36500
H	10.04030	-0.22230	11.57780

Tb’ (optimized)

Sum of Electronic and Thermal Free Energies
(M06-2X-D3/3-21G: -8546.688316 Hartree

No imaginary frequencies found.

C	-7.80340	8.96150	-5.94980
C	-6.65730	9.42230	-6.62480
C	-5.78130	10.33650	-5.99850
C	-6.07960	10.81720	-4.70960
C	-7.22310	10.35200	-4.02320
C	-8.09390	9.44380	-4.65410
C	-8.74600	7.95370	-6.60680
C	-6.35900	8.96050	-8.04900
C	-4.52460	10.80080	-6.73410
C	-5.19320	11.87210	-4.05200
C	-7.50500	10.84960	-2.60620
C	-9.37640	8.99300	-3.96030
C	-9.84380	8.64330	-7.46000
C	-4.76860	12.08660	-7.56840
C	-8.40850	12.11150	-2.58950
C	-3.45710	5.74450	-8.51540
C	-3.71460	6.65860	-9.55760
C	-4.62860	7.69130	-9.38700
C	-5.31690	7.84800	-8.17250
C	-5.05940	6.94830	-7.12900
C	-4.14440	5.90950	-7.29880
C	-2.26280	10.49740	-1.13680
C	-2.75430	11.81880	-1.11900
C	-3.70390	12.23580	-2.04340
C	-4.19600	11.35070	-3.01690
C	-3.71910	10.03280	-3.03500
C	-2.76360	9.61270	-2.10950
C	-9.32390	5.08930	-2.02480
C	-8.11690	5.73340	-2.35160
C	-8.11750	6.97860	-2.98100
C	-9.32480	7.61280	-3.30250
C	-10.53320	6.97260	-2.97970
C	-10.53510	5.73460	-2.34990
B	-2.45750	4.61570	-8.74420
O	-1.75180	4.41980	-9.96540
B	-1.21010	10.08420	-0.11360
O	-0.58590	8.81360	0.01750
B	-9.36850	3.72600	-1.34260
O	-10.57550	3.07430	-0.96030
O	-2.08140	3.60030	-7.82280
O	-8.26470	2.90210	-0.99010
O	-0.68730	10.96320	0.87760
C	-1.14850	2.78260	-8.48930
C	-0.95140	3.28100	-9.78490
C	-0.08940	2.69090	-10.69680
C	-0.49590	1.65450	-8.01050
C	0.25830	10.22530	1.60610
C	0.32240	8.92400	1.08820
C	1.17370	7.95310	1.59840
C	1.03800	10.65280	2.67020
C	-8.80440	1.74580	-0.39400
C	-10.20210	1.85430	-0.37560
C	-11.02770	0.87380	0.15320
C	-8.12930	0.64290	0.11170
C	0.56090	1.56340	-10.21860
C	0.36610	1.06240	-8.92370
O	1.16390	-0.08580	-8.75380
B	1.85400	-0.28190	-9.98120
O	1.48160	0.74680	-10.89300
C	1.95570	8.38120	2.66280
C	1.88900	9.68320	3.17850
O	2.79970	9.80400	4.23970
B	3.43980	8.53870	4.36930
O	2.91410	7.65040	3.39140
C	-10.35290	-0.22630	0.66050
C	-8.95570	-0.34030	0.63890
O	-8.58900	-1.56880	1.22200
B	-9.79880	-2.20850	1.60760
O	-10.89790	-1.37310	1.25840
H	-6.02280	9.81470	-8.64560
H	-7.28310	8.61080	-8.52010
H	-5.82540	12.62490	-3.56980
H	-4.62900	12.40510	-4.82410
H	-9.65130	9.72240	-3.19210
H	-10.19870	8.99590	-4.68390
H	-9.21600	7.34280	-5.83170
H	-8.17370	7.26330	-7.23440
H	-3.71830	10.97820	-6.01550
H	-4.17690	9.99920	-7.39110
H	-7.97600	10.05330	-2.02120
H	-6.55680	11.07310	-2.11020
H	-3.18810	6.54520	-10.49850
H	-4.81800	8.38630	-10.19910
H	-5.57770	7.07450	-6.18590
H	-3.95640	5.21620	-6.48620
H	-2.37990	12.50890	-0.37130
H	-4.07170	13.25690	-2.01860
H	-4.10730	9.34300	-3.77490
H	-2.39810	8.59200	-2.13570
H	-7.17640	5.25060	-2.10960
H	-7.18400	7.46630	-3.23490
H	-11.47350	7.45520	-3.22780
H	-11.47230	5.24780	-2.10490
H	0.06040	3.07460	-11.69390
H	-0.64790	1.26910	-7.01430
H	1.22520	6.95180	1.19980
H	0.98860	11.65490	3.06680
H	-12.10300	0.95940	0.16860
H	-7.05390	0.55730	0.09630
C	7.78330	-6.70290	-8.42920
C	6.61180	-7.26930	-8.96600
C	5.86150	-6.56810	-9.93590
C	6.31120	-5.31540	-10.39410
C	7.48110	-4.73770	-9.85320
C	8.22640	-5.44300	-8.88990
C	8.59040	-7.44020	-7.36100
C	6.15120	-8.65700	-8.52740
C	4.57500	-7.18480	-10.48400
C	5.56290	-4.58250	-11.50480
C	7.92830	-3.35620	-10.32970
C	9.53630	-4.87140	-8.35470
C	3.01590	-8.83470	-5.50760
C	3.23440	-9.90130	-6.40340
C	4.22530	-9.82180	-7.37430
C	5.03200	-8.67720	-7.48530
C	4.81440	-7.60920	-6.60390
C	3.82220	-7.68780	-5.62700
C	2.83270	-1.40000	-10.32450
C	3.40890	-1.43560	-11.61100
C	4.29400	-2.44630	-11.96480
C	4.63500	-3.45580	-11.04940
C	4.07370	-3.42170	-9.76560
C	3.18280	-2.40920	-9.40890
C	9.40990	-2.91330	-4.46410
C	10.62470	-3.35590	-5.02740
C	10.64490	-3.99120	-6.26240
C	9.45630	-4.20270	-6.98120
C	8.24560	-3.76410	-6.42880
C	8.22250	-3.12900	-5.18670
C	9.64710	-8.39610	-7.97620
C	4.82450	-8.04420	-11.75200
C	8.91090	-3.43210	-11.52880
C	-8.65390	-9.09620	4.24010
C	-7.57900	-9.73800	4.89420
C	-7.17180	-9.31410	6.17320
C	-7.87410	-8.27470	6.82260
C	-8.95180	-7.63670	6.17990
C	-9.35190	-8.06350	4.89400
C	-9.05110	-9.53900	2.83250
C	-6.88930	-10.91760	4.21330
C	-5.98340	-9.97250	6.87330
C	-7.46610	-7.86990	8.23680
C	-9.70380	-6.49410	6.86190

C	-10.56610	-7.41370	4.23630
C	-3.85970	-10.01380	1.21650
C	-4.55320	-11.24150	1.21470
C	-5.53100	-11.50620	2.16540
C	-5.85110	-10.55690	3.15030
C	-5.17240	-9.33060	3.15290
C	-4.18850	-9.06370	2.20080
C	-4.08390	-5.14970	8.61450
C	-4.45120	-6.01550	9.66490
C	-5.52020	-6.89180	9.52220
C	-6.25920	-6.93500	8.32840
C	-5.89390	-6.08360	7.27650
C	-4.82310	-5.20140	7.41840
C	-9.95120	-3.56310	2.29150
C	-11.23950	-4.00890	2.65280
C	-11.41570	-5.23300	3.28520
C	-10.31530	-6.05750	3.57450
C	-9.03250	-5.62200	3.21670
C	-8.85260	-4.39120	2.58480
C	-10.14300	-10.64200	2.84350
C	-6.40260	-11.20620	7.71640
C	-10.87250	-7.00370	7.74690
C	8.39630	3.48200	9.64080
C	9.21820	4.06260	8.65670
C	8.96740	5.37730	8.20430
C	7.91430	6.12050	8.77000
C	7.08800	5.54480	9.76050
C	7.34630	4.23620	10.21000
C	8.63220	2.04650	10.10890
C	10.40680	3.29230	8.08860
C	9.85180	5.97950	7.11290
C	7.66680	7.56420	8.33970
C	5.92850	6.35520	10.33890
C	6.52030	3.62850	11.34110
C	9.86980	1.38710	4.20660
C	11.15380	1.62950	4.73740
C	11.30670	2.25110	5.97020
C	10.18590	2.64760	6.71900
C	8.90700	2.40850	6.19890
C	8.75100	1.78770	4.95920
C	4.52090	8.23830	5.40200
C	4.92670	9.25520	6.29030
C	5.91790	9.01910	7.23510
C	6.53830	7.76210	7.32670
C	6.13370	6.74380	6.45260
C	5.14090	6.97920	5.50180
C	3.29300	0.91850	10.23800
C	3.90230	0.85870	11.50820
C	4.94430	1.71670	11.83710
C	5.41460	2.66350	10.91200
C	4.82010	2.72270	9.64410
C	3.77200	1.86390	9.31260
C	9.64530	1.96400	11.28180
C	11.06210	6.75630	7.69660
C	6.34130	7.16350	11.59800
B	-2.78350	-9.76890	0.16410
O	-1.97170	-8.61160	0.01290
O	-2.43180	-10.71720	-0.83860
C	-1.12110	-8.86110	-1.08150
C	-1.40230	-10.13540	-1.59420
C	-0.72810	-10.67800	-2.67770
C	-0.14230	-8.03470	-1.61680
C	0.25040	-9.85300	-3.21110
C	0.53410	-8.57830	-2.70070
O	1.57520	-8.00550	-3.45660
B	1.92880	-8.96360	-4.44590
O	1.10200	-10.11320	-4.29590
B	9.74380	0.71110	2.84550
O	8.54790	0.43630	2.12740
O	10.85780	0.25400	2.08510
C	8.93800	-0.18760	0.92650
C	10.33550	-0.29830	0.90530
C	11.03040	-0.87500	-0.14710
C	8.13210	-0.64190	-0.10880
C	10.22490	-1.33090	-1.17960
C	8.82760	-1.21700	-1.16390
O	8.31140	-1.76790	-2.35280
B	9.42880	-2.22870	-3.10150
O	10.62100	-1.95710	-2.37150
B	2.14240	-0.03150	9.92300
O	1.39590	-0.11320	8.71570
O	1.63970	-0.99270	10.84590
C	0.43380	-1.12350	8.90920
C	0.58420	-1.65320	10.19860
C	-0.22040	-2.66710	10.69600
C	-0.53530	-1.57060	8.02120
C	-1.18910	-3.11240	9.80930
C	-1.34230	-2.58470	8.51940
O	-2.40990	-3.24440	7.88050
B	-2.91400	-4.19180	8.81320
O	-2.15220	-4.11270	10.01360
H	5.81650	-9.22280	-9.40280
H	7.00210	-9.21160	-8.11910
H	6.28630	-4.16570	-12.21320
H	4.96480	-5.30050	-12.07510
H	9.93110	-4.13760	-9.06430
H	10.28410	-5.67010	-8.30250
H	2.61670	-10.78880	-6.32490
H	4.38320	-10.65190	-8.05590
H	5.42520	-6.71900	-6.69570
H	3.66580	-6.85750	-4.94710
H	3.15100	-0.66090	-12.32410
H	4.72820	-2.46180	-12.95970
H	4.34580	-4.19010	-9.05190
H	2.75070	-2.39530	-8.41420
H	11.54680	-3.19710	-4.47960
H	11.58730	-4.33040	-6.68140
H	7.32660	-3.93190	-6.97740
H	7.27930	-2.79570	-4.76770
H	9.08910	-6.70930	-6.71920
H	7.91660	-8.00770	-6.71150
H	3.85310	-6.39470	-10.71350
H	4.11470	-7.80270	-9.70840
H	8.39890	-2.81410	-9.50340
H	7.04840	-2.77380	-10.61530
H	-6.39510	-11.53550	4.96980
H	-7.64570	-11.55940	3.74970
H	-8.30980	-7.38090	8.73410
H	-7.25100	-8.76810	8.82460
H	-11.35730	-7.28800	4.98330
H	-10.97490	-8.08840	3.47780
H	-4.31240	-11.98010	0.45850
H	-6.05530	-12.45670	2.15280
H	-5.42680	-8.59010	3.90190
H	-3.66640	-8.11320	2.21490
H	-3.88670	-5.98880	10.59010
H	-5.79260	-7.55120	10.34050
H	-6.45250	-6.12410	6.34900
H	-4.55230	-4.54360	6.59970
H	-12.09430	-3.37910	2.43380
H	-12.41310	-5.56090	3.56150
H	-8.18130	-6.25230	3.44480
H	-7.85480	-4.06310	2.31470
H	-9.40670	-8.67650	2.25980
H	-8.16490	-9.90830	2.30990
H	-5.49520	-9.23740	7.51870
H	-5.23600	-10.27240	6.13210
H	-10.09270	-5.81410	6.09950
H	-9.01170	-5.90520	7.47190
H	11.26990	3.96280	8.01530
H	10.69890	2.50380	8.78910
H	7.44800	8.17420	9.22200
H	8.58360	7.97850	7.90840
H	6.05830	4.43090	11.92520
H	7.18660	3.10100	12.03140
H	12.02440	1.32720	4.16630
H	12.30160	2.43520	6.36390
H	8.04100	2.71930	6.77070
H	7.75630	1.60980	4.56530
H	4.45350	10.22860	6.22680
H	6.22130	9.81240	7.91130
H	6.60000	5.76880	6.52950

H	4.83900	6.18540	4.82730
H	3.54550	0.13130	12.22860
H	5.40240	1.66030	12.81970
H	5.18970	3.44170	8.92270
H	3.31620	1.92170	8.33020
H	7.67910	1.60980	10.41870
H	8.98950	1.43830	9.27190
H	9.25870	6.64690	6.47990
H	10.21160	5.18000	6.46010
H	5.09770	5.68790	10.58870
H	5.55140	7.03850	9.57330
H	-0.94460	-11.65930	-3.07030
H	0.07620	-7.05460	-1.22230
H	12.10560	-0.96140	-0.16140
H	7.05670	-0.55540	-0.09450
H	-0.10440	-3.07290	11.68880
H	-0.65330	-1.16270	7.02940
H	-5.53260	-11.64450	8.21840
H	-7.13640	-10.92280	8.47800
H	-6.86000	-11.97270	7.08220
H	-10.39280	-10.94800	1.82130
H	-9.79720	-11.52270	3.39450
H	-11.05510	-10.28160	3.33050
H	-11.40240	-6.16280	8.20860
H	-11.58740	-7.57810	7.14870
H	-10.50090	-7.65860	8.54170
H	-3.85510	12.38390	-8.09570
H	-10.51300	7.89770	-7.90410
H	-8.57710	12.45180	-1.56150
H	-9.39570	9.23240	-8.26690
H	-10.44150	9.32280	-6.84370
H	-5.08210	12.91410	-6.92350
H	-5.55900	11.92320	-8.30820
H	-9.37970	11.90050	-3.04890
H	-7.94390	12.92710	-3.15310
H	9.19920	-2.42660	-11.85570
H	10.21990	-8.89730	-7.18780
H	3.88730	-8.48510	-12.11030
H	10.34440	-7.84190	-8.61300
H	9.16580	-9.16020	-8.59540
H	5.52940	-8.85410	-11.53750
H	5.25140	-7.43540	-12.55580
H	8.44940	-3.95270	-12.37420
H	9.81750	-3.98130	-11.25440
H	5.49430	7.74600	11.97770
H	9.77830	0.92520	11.60460
H	11.68530	7.16170	6.89150
H	9.29500	2.55080	12.13730
H	10.62040	2.36180	10.98220
H	11.68030	6.09850	8.31630
H	10.72410	7.58620	8.32580
H	7.15940	7.85260	11.36410
H	6.68660	6.49440	12.39290

Td

Sum of Electronic and Thermal Free Energies
(M06-2X-D3/3-21G: -9950.726451 Hartree

No imaginary frequencies found.

C	10.07500	-1.94880	8.36860
C	10.05930	-3.29570	7.94330
C	9.18320	-4.22570	8.54780
C	8.30800	-3.79780	9.57080
C	8.31690	-2.45180	10.00220
C	9.19570	-1.52900	9.39260
C	11.04830	-0.95510	7.72850
C	10.96610	-3.75230	6.79870
C	9.18680	-5.69220	8.10720
C	7.31600	-4.78060	10.19630
C	7.38890	-1.99380	11.13060
C	9.17890	-0.05750	9.81130
C	9.01950	-4.65620	3.00670
C	10.21760	-5.29320	3.38840
C	10.81900	-5.00420	4.60740
C	10.24530	-4.07360	5.48910
C	9.05440	-3.43480	5.11580
C	8.45110	-3.72040	3.89120
C	3.11690	-4.24130	9.16580
C	3.50540	-4.79950	10.40060
C	4.84910	-4.95150	10.72040
C	5.85150	-4.54970	9.82250
C	5.47290	-3.99520	8.59170
C	4.12490	-3.84460	8.26720
C	7.48150	2.76540	6.95910
C	7.04760	1.42650	6.97680
C	7.59530	0.50850	7.87260
C	8.58980	0.90370	8.77840
C	9.02240	2.23980	8.76780
C	8.48050	3.15550	7.87390
B	8.39310	-5.01570	1.66340
O	8.95920	-5.96880	0.76890
B	1.62900	-4.08720	8.86800
O	1.02470	-3.54660	7.70030
B	6.92600	3.80540	5.99180
O	5.92880	3.62270	4.99500
O	7.19730	-4.49900	1.09400
O	7.36610	5.15980	5.96530
O	0.60760	-4.48500	9.77770
C	7.03720	-5.14170	-0.14950
C	8.10480	-6.02990	-0.34200
C	8.23610	-6.81740	-1.47550
C	6.02040	-4.97620	-1.08070
C	-0.61560	-4.18380	9.16070
C	-0.36800	-3.61570	7.90310
C	-1.38470	-3.20940	7.04890
C	-1.89350	-4.38810	9.65800
C	6.63590	5.79700	4.95120
C	5.76470	4.87030	4.36130
C	4.90600	5.20260	3.32170
C	6.71470	7.12100	4.54790
C	7.22060	-6.65180	-2.40460
C	6.15150	-5.76450	-2.21640
O	5.29960	-5.83270	-3.33660
B	5.87320	-6.78660	-4.22110
O	7.06940	-7.29760	-3.64070
C	-2.66500	-3.41370	7.54650
C	-2.90800	-3.98220	8.80480
O	-4.29270	-4.05490	9.01650
B	-4.90950	-3.51490	7.85170
O	-3.89890	-3.11460	6.93540
C	4.98440	6.52880	2.91740
C	5.85730	7.45220	3.50990
O	5.70130	8.69830	2.88500
B	4.70310	8.52710	1.88340
O	4.25580	7.17770	1.90080
H	11.53320	-4.63530	7.10520
H	11.70640	-2.97950	6.58190
H	7.40180	-4.75090	11.28590
H	7.57000	-5.80080	9.90190
H	8.60220	0.05960	10.73100
H	10.19410	0.27210	10.04790
H	10.71400	0.06580	7.91470
H	11.02620	-1.07850	6.63950
H	8.15280	-6.03370	7.98080
H	9.64830	-5.78420	7.12370
H	6.91610	-1.04630	10.84690
H	6.57120	-2.70470	11.25220
H	10.66430	-6.01800	2.71730
H	11.74100	-5.50370	4.88790
H	8.60520	-2.72050	5.79570
H	7.53020	-3.21990	3.61250
H	2.73870	-5.10690	11.10260
H	5.13250	-5.38190	11.67570
H	6.24310	-3.67880	7.89810
H	3.84370	-3.41450	7.31200
H	6.27870	1.10980	6.28060
H	7.26280	-0.52290	7.87390
H	9.79180	2.55500	9.46550
H	8.82520	4.18330	7.86880
H	9.05820	-7.50040	-1.62190
H	5.19820	-4.29300	-0.93420
H	-1.19590	-2.77200	6.08090
H	-2.08220	-4.82540	10.62600
H	4.23490	4.49080	2.86670
H	7.38570	7.83280	5.00280
H	13.18510	-0.57780	7.54880
H	7.47450	-1.23070	13.16790
H	9.64890	-7.67550	8.87760
C	1.62550	-5.81260	-11.78230
C	3.01160	-5.60140	-11.60480
C	3.78810	-6.53060	-10.87730
C	3.16820	-7.66790	-10.31150
C	1.78340	-7.88680	-10.48410
C	1.01410	-6.95210	-11.21370
C	0.78780	-4.81920	-12.59250
C	3.65820	-4.33860	-12.17730
C	5.29370	-6.30610	-10.71120
C	4.00430	-8.64230	-9.47970
C	1.12590	-9.13590	-9.89050
C	-0.49440	-7.16590	-11.35190
C	2.66680	-0.57340	-10.22130
C	3.01770	-0.62360	-11.58570
C	3.31740	-1.83430	-12.19830
C	3.27460	-3.03660	-11.47350
C	2.92770	-2.99450	-10.11590
C	2.62980	-1.78010	-9.49790
C	5.34880	-7.23210	-5.58220
C	6.07750	-8.19680	-6.30700
C	5.64280	-8.62600	-7.55510
C	4.46920	-8.10620	-8.12520
C	3.73830	-7.14730	-7.40980
C	4.17060	-6.71770	-6.15530
C	-2.89330	-6.57650	-7.76260
C	-3.30170	-7.45800	-8.78420
C	-2.51790	-7.64590	-9.91600
C	-1.29920	-6.96430	-10.06760
C	-0.88720	-6.08480	-9.05670
C	-1.67370	-5.89220	-7.92100
C	1.37080	12.70740	-3.46040
C	0.04480	12.63360	-3.94400
C	-1.04640	12.83750	-3.07050
C	-0.80690	13.10010	-1.70250
C	0.51520	13.17690	-1.21110
C	1.60130	12.97190	-2.09220
C	2.55440	12.51010	-4.41160
C	-0.20020	12.29630	-5.41600
C	-2.47860	12.78260	-3.60990
C	-1.99310	13.26730	-0.75110
C	0.76590	13.49130	0.26650
C	3.02920	13.00360	-1.54380
C	0.63910	8.20340	-6.65280
C	0.73890	9.28030	-7.55690
C	0.48150	10.58160	-7.14330

C	0.11910	10.85490	-5.81410
C	0.01620	9.78890	-4.90950
C	0.27070	8.48150	-5.32310
C	-4.28420	9.67780	0.17220
C	-4.87660	10.95660	0.20180
C	-4.13590	12.09040	-0.10920
C	-2.78070	11.98850	-0.46380
C	-2.18430	10.72020	-0.49470
C	-2.92480	9.58110	-0.17930
C	4.23160	9.65610	0.97280
C	4.80670	10.93410	1.12450
C	4.40220	11.99500	0.32340
C	3.40870	11.81970	-0.65370
C	2.83290	10.55130	-0.81130
C	3.23990	9.48400	-0.01090
C	-12.37790	-3.33350	3.33850
C	-11.82120	-4.49270	3.92520
C	-11.31840	-4.45430	5.24470
C	-11.35840	-3.24350	5.97280
C	-11.91320	-2.08030	5.39440
C	-12.41490	-2.12790	4.07380
C	-12.94720	-3.38140	1.91770
C	-11.73490	-5.78200	3.10620
C	-10.74280	-5.72200	5.88210
C	-10.76280	-3.19400	7.38100
C	-11.97710	-0.78030	6.20100
C	-12.96770	-0.85280	3.43430
C	-8.83150	-5.83950	-0.14210
C	-10.00660	-6.61660	-0.19230
C	-10.93010	-6.57640	0.84520
C	-10.71600	-5.75830	1.96640
C	-9.54910	-4.98320	2.02420
C	-8.61920	-5.02450	0.98560
C	-6.42150	-3.41470	7.67840
C	-7.25450	-3.87940	8.71640
C	-8.63770	-3.81570	8.60000
C	-9.23810	-3.28960	7.44440
C	-8.41620	-2.82480	6.40810
C	-7.02760	-2.88470	6.52410
C	-10.06980	2.22930	2.39130
C	-11.40400	2.55230	2.71180
C	-12.31250	1.55890	3.05610
C	-11.92210	0.21030	3.09610
C	-10.59710	-0.11880	2.77750
C	-9.68440	0.87620	2.42810
B	0.94180	6.79510	-7.15380
O	0.90780	5.58110	-6.41460
O	1.32070	6.51020	-8.49700
C	1.26830	4.55690	-7.31260
C	1.51720	5.12330	-8.57070
C	1.89570	4.37400	-9.67400
C	1.38050	3.19480	-7.06650
C	2.00770	3.01430	-9.42760
C	1.75970	2.44370	-8.17120
O	1.95990	1.05150	-8.25520
B	2.33800	0.77940	-9.59840
O	2.36830	2.00180	-10.32890
B	-7.85370	-5.91270	-1.31020
O	-6.62510	-5.21390	-1.46330
O	-8.06660	-6.73360	-2.45470
C	-6.09180	-5.61160	-2.70530
C	-6.96680	-6.53070	-3.30140
C	-6.72430	-7.11120	-4.53670
C	-4.90660	-5.20540	-3.30430
C	-5.54100	-6.70530	-5.13420
C	-4.66310	-5.78660	-4.54180
O	-3.56250	-5.58960	-5.39920
B	-3.78560	-6.41300	-6.53650
O	-5.01820	-7.10770	-6.37200
B	-9.10920	3.35720	2.02910
O	-7.73500	3.25300	1.68020
O	-9.49320	4.72860	1.99870
C	-7.28390	4.56580	1.43850
C	-8.35040	5.45490	1.63250
C	-8.22800	6.82650	1.47180
C	-6.01320	4.98610	1.06810
C	-6.95920	7.24550	1.10230
C	-5.89000	6.35990	0.90700
O	-4.74760	7.09830	0.53970
B	-5.14200	8.46390	0.51380
O	-6.51940	8.55610	0.86460
H	4.74550	-4.42570	-12.13170
H	3.40790	-4.24290	-13.23720
H	3.43290	-9.55320	-9.29080
H	4.88580	-8.95140	-10.04770
H	-0.69260	-8.17340	-11.72700
H	-0.89880	-6.48120	-12.10020
H	3.04750	0.29860	-12.15480
H	3.58500	-1.85690	-13.25000
H	2.88790	-3.92040	-9.55420
H	2.36300	-1.75970	-8.44690
H	6.98820	-8.59910	-5.87790
H	6.21530	-9.36900	-8.10140
H	2.83530	-6.73930	-7.84870
H	3.59700	-5.97570	-5.61070
H	-4.23800	-7.99290	-8.67330
H	-2.84540	-8.32870	-10.69360
H	0.05490	-5.56080	-9.16720
H	-1.34500	-5.20900	-7.14540
H	-0.14130	-4.60620	-12.05120
H	1.31010	-3.86500	-12.66610
H	5.66810	-6.89650	-9.87450
H	5.47400	-5.25990	-10.43790
H	0.04800	-8.98970	-9.81570
H	1.47710	-9.27380	-8.86130
H	-1.24640	12.48030	-5.66890
H	0.38580	12.96580	-6.05120
H	-1.64670	13.65860	0.20760
H	-2.68450	14.01450	-1.14970
H	3.18710	13.92480	-0.97670
H	3.74260	13.03260	-2.36980
H	1.02420	9.07980	-8.58340
H	0.56350	11.40050	-7.85100
H	-0.25780	9.99610	-3.88160
H	0.18780	7.66450	-4.61460
H	-5.92370	11.04450	0.46840
H	-4.60640	13.06820	-0.08240
H	-1.14050	10.63670	-0.77360
H	-2.45270	8.60500	-0.20590
H	5.56980	11.08050	1.88050
H	4.85390	12.97360	0.45280
H	2.06150	10.41320	-1.55980
H	2.78770	8.50690	-0.14190
H	3.28630	11.84290	-3.94170
H	2.22460	11.99490	-5.31430
H	-3.17950	12.62200	-2.79020
H	-2.58410	11.90910	-4.26380
H	1.77480	13.18650	0.54590
H	0.09290	12.88490	0.88370
H	-11.47510	-6.61810	3.75840
H	-12.71570	-6.02050	2.68650
H	-11.05130	-2.26290	7.87270
H	-11.18350	-3.99930	7.98890
H	-13.71500	-0.40290	4.09340
H	-13.49210	-1.09980	2.50910
H	-10.18340	-7.24660	-1.05670
H	-11.83090	-7.17980	0.79160
H	-9.38320	-4.34600	2.88500
H	-7.71960	-4.42110	1.04120
H	-6.80000	-4.29260	9.60970
H	-9.26600	-4.17840	9.40740
H	-8.87580	-2.42420	5.51210
H	-6.40150	-2.52160	5.71630
H	-11.71300	3.59120	2.68980
H	-13.33640	1.82330	3.30120
H	-10.29000	-1.15750	2.81320
H	-8.66190	0.60990	2.18310
H	-12.59820	-2.50490	1.35960
H	-12.54840	-4.24580	1.38610
H	-10.11220	-5.45810	6.73170
H	-10.07860	-6.21720	5.16430
H	-11.01430	-0.61770	6.69920

H	-12.10720	0.06870	5.52930
H	2.08700	4.81160	-10.64130
H	1.18930	2.75720	-6.09890
H	-7.39870	-7.81830	-4.99400
H	-4.23210	-4.49820	-2.84700
H	-9.04970	7.50930	1.62170
H	-5.19140	4.30320	0.91840
H	0.49300	15.09720	1.71120
H	4.23940	13.60850	-5.24450
H	-3.79410	13.84440	-4.98230
H	7.11640	-6.20780	-11.89790
H	1.15780	-11.30390	-10.08990
H	-0.37370	-4.71280	-14.43110
H	-11.36400	-7.70500	6.53170
H	-12.92600	0.04660	7.97770
H	-14.83960	-3.18500	0.85940
C	3.26360	13.84590	-4.79810
C	2.47070	14.73220	-5.78700
C	2.52060	14.21490	-7.24260
C	1.65130	15.06590	-8.19350
H	1.43000	14.81560	-5.44860
H	2.89070	15.74710	-5.77090
H	3.56360	14.22840	-7.58540
H	2.18870	13.16990	-7.28050
H	1.72120	14.70420	-9.22540
H	0.59870	15.03050	-7.88730
H	1.97410	16.11350	-8.17490
C	0.55530	14.99750	0.61850
C	1.66550	15.93970	0.09740
C	2.96380	15.87230	0.93330
C	4.08460	16.75320	0.34020
H	1.87730	15.70580	-0.95380
H	1.29630	16.97400	0.12330
H	2.74070	16.19950	1.95740
H	3.31170	14.83410	1.00180
H	4.98740	16.71760	0.96010
H	4.34720	16.41370	-0.66920
H	3.75690	17.79710	0.27160
C	-2.88450	14.06290	-4.40540
C	-3.14330	15.30950	-3.52730
C	-4.50060	15.26330	-2.78930
C	-4.69720	16.47640	-1.85460
H	-2.32280	15.42480	-2.80750
H	-3.13220	16.20320	-4.16590
H	-5.30610	15.23830	-3.53510
H	-4.57920	14.33650	-2.20750
H	-5.67520	16.44290	-1.36170
H	-3.92180	16.49420	-1.07910
H	-4.63080	17.41260	-2.42120
H	3.45630	14.42260	-3.88680
H	-0.40560	15.32700	0.20820
H	-2.09350	14.29950	-5.12550
C	12.51730	-1.12650	8.22760
C	12.76510	-0.63320	9.67220
C	12.85020	0.90610	9.78330
C	13.00740	1.37730	11.24520
H	11.97480	-1.01880	10.32880
H	13.71230	-1.05460	10.03550
H	13.70510	1.25520	9.18940
H	11.95370	1.36310	9.34610
H	13.09490	2.46790	11.30330
H	12.14140	1.06960	11.84380
H	13.90440	0.93710	11.69640
C	9.91150	-6.63570	9.11760
C	11.45240	-6.50310	9.12600
C	12.12970	-7.18920	7.91770
C	13.65740	-6.96620	7.90260
H	11.72590	-5.44080	9.16220
H	11.84480	-6.96260	10.04340
H	11.91450	-8.26510	7.95740
H	11.69590	-6.81410	6.98240
H	14.12550	-7.48300	7.05740
H	13.88940	-5.89720	7.82310
H	14.10960	-7.34280	8.82760
C	8.12630	-1.80310	12.49310
C	8.52880	-3.12160	13.19440
C	7.33950	-3.83520	13.87640
C	7.74770	-5.19150	14.49110
H	9.00650	-3.78990	12.46660
H	9.28150	-2.90120	13.96360
H	6.93910	-3.18030	14.66140
H	6.53100	-3.99060	13.15130
H	6.90030	-5.66990	14.99470
H	8.11380	-5.87190	13.71260
H	8.54990	-5.05440	15.22550
H	12.79220	-2.18520	8.16610
H	9.53610	-6.43160	10.12630
H	9.02820	-1.20340	12.32840
C	6.11110	-6.64020	-11.99840
C	6.25030	-8.15130	-12.29730
C	7.28200	-8.85930	-11.39000
C	7.34010	-10.38000	-11.65060
H	5.26860	-8.63330	-12.20450
H	6.56860	-8.27990	-13.34090
H	8.27070	-8.41660	-11.56910
H	7.04030	-8.67750	-10.33540
H	8.09620	-10.86190	-11.02080
H	6.36930	-10.84420	-11.43820
H	7.58890	-10.58070	-12.69920
C	1.42550	-10.43350	-10.70510
C	0.67620	-10.53150	-12.05440
C	-0.81440	-10.90880	-11.89760
C	-1.55940	-10.91360	-13.25000
H	0.77340	-9.58090	-12.59430
H	1.15880	-11.29880	-12.67510
H	-0.87920	-11.90360	-11.43750
H	-1.30740	-10.20980	-11.21060
H	-2.60660	-11.21080	-13.12510
H	-1.53710	-9.91580	-13.70490
H	-1.08510	-11.61410	-13.94720
C	0.43380	-5.33610	-14.02230
C	1.61700	-5.32940	-15.01820
C	1.95860	-3.91880	-15.54940
C	3.19600	-3.92840	-16.47280
H	2.49700	-5.77920	-14.54100
H	1.36310	-5.96530	-15.87730
H	1.09130	-3.52990	-16.09910
H	2.13380	-3.23450	-14.70980
H	3.40730	-2.92680	-16.86360
H	4.08070	-4.27800	-15.92690
H	3.03520	-4.60130	-17.32330
H	5.63460	-6.15530	-12.85750
H	2.50260	-10.49160	-10.89660
H	0.04630	-6.35790	-13.94480
C	-14.50740	-3.41580	1.88120
C	-15.13030	-4.76430	2.31170
C	-15.02890	-5.85750	1.22370
C	-15.57890	-7.21580	1.71010
H	-14.65350	-5.10570	3.23950
H	-16.19330	-4.60880	2.54080
H	-15.59030	-5.52770	0.33970
H	-13.98480	-5.97710	0.90880
H	-15.52720	-7.97140	0.91840
H	-15.00240	-7.57860	2.56980
H	-16.62530	-7.11700	2.02180
C	-11.84080	-6.73420	6.33640
C	-12.62690	-6.30600	7.59760
C	-11.82980	-6.50040	8.90760
C	-12.60160	-5.98360	10.14090
H	-12.93890	-5.25890	7.49430
H	-13.54550	-6.90470	7.66510
H	-11.61060	-7.56920	9.03100
H	-10.86360	-5.98530	8.83800
H	-12.03620	-6.15550	11.06350
H	-12.79400	-4.90750	10.05100
H	-13.56770	-6.49380	10.23080
C	-13.10950	-0.77860	7.27540
C	-14.53830	-0.63150	6.70160
C	-14.87270	0.81250	6.26370
C	-16.27030	0.91580	5.61550
H	-14.66510	-1.32340	5.85900
H	-15.26350	-0.93200	7.46990

H	-14.82540	1.46720	7.14390	H	-14.89550	-2.62600	2.53380
H	-14.11450	1.17520	5.55850	H	-12.54970	-6.88150	5.51420
H	-16.50320	1.94960	5.33740	H	-13.05630	-1.71010	7.84950
H	-16.32180	0.29670	4.71150				
H	-17.04270	0.56530	6.31010				

12. References

1) Gunther, J. R.; Moore, T. W.; Collins, M. L.; Katzenellenbogen, J. A. *ACS Chem. Biol.* **2008**, *3,* 282-286.

2) Shi, C.; Chien, C.; Ojima, I. *Chem. – Asian J.* **2011**, *6*, 674-680.

3) Dzhevakov, P. B.; Topchiy, M. A.; Zharkova, D. A.; Morozov, O. S.; Asachenko, A. F.; Nechaev, M. S. *Adv. Synth. Catal.* **2016**, *358*, 977-983.

4) Cromwell, O. R.; Chung, J.; Guan, Z. *J. Am. Chem. Soc.* **2015**, *137*, 6492-6495.

5) Weider, P. R.; Hegedus, L. S.; Asada, H. *J. Org. Chem.* **1985**, *50*, 4276-4281.

6) Pauli, G. F.; Chen, S.-N.; Simmler, C.; Lankin, D. C.; Gödecke, T.; Jaki, B. U.; Friesen, J. B.; McAlpine J. B.; Napolitano, J. G. *J. Med. Chem.* **2014**, *57*, 9220-9231.

7) Rigaku Oxford Diffraction, *CrysAlisPro Software system, version 1.171.40.53,* Rigaku Corporation, Wroclaw, Poland, 2019.

8) Sheldrick, G. M. *Acta Crystallogr. Sect. A* **2015**, *A71*, 3-8.

9) Sheldrick, G. M. *Acta Crystallogr. Sect. C* **2015**, *C71*, 3-8.

10) Dolomanov, O. V.; Bourhis, L. J.; Gildea, R. J.; Howard, J. A. K.; Puschmann, H. *J. Appl. Cryst.* **2009**, *42*, 339-341.
